# Associations of *NINJ2* Sequence Variants with Incident Ischemic Stroke in the Cohorts for Heart and Aging in Genomic Epidemiology (CHARGE) Consortium

**DOI:** 10.1371/journal.pone.0099798

**Published:** 2014-06-24

**Authors:** Joshua C. Bis, Anita DeStefano, Xiaoming Liu, Jennifer A. Brody, Seung Hoan Choi, Benjamin F. J. Verhaaren, Stéphanie Debette, M. Arfan Ikram, Eyal Shahar, Kenneth R. Butler, Rebecca F. Gottesman, Donna Muzny, Christie L. Kovar, Bruce M. Psaty, Albert Hofman, Thomas Lumley, Mayetri Gupta, Philip A. Wolf, Cornelia van Duijn, Richard A. Gibbs, Thomas H. Mosley, W. T. Longstreth, Eric Boerwinkle, Sudha Seshadri, Myriam Fornage

**Affiliations:** 1 Cardiovascular Health Research Unit, Department of Medicine, University of Washington, Seattle, Washington, United States of America; 2 Department of Biostatistics, Boston University School of Public Health, Boston, Massachusetts, United States of America; 3 Human Genetics Center, University of Texas Health Science Center at Houston, Houston, Texas, United States of America; 4 Department of Radiology, Erasmus MC, Rotterdam, The Netherlands; 5 Department of Epidemiology, Erasmus MC, Rotterdam, The Netherlands; 6 Institut National de la Santé et de la Recherche Médicale (INSERM), U708, Neuroepidemiology, Paris, France; 7 Department of Epidemiology, University of Versailles Saint-Quentin-en-Yvelines, Paris, France; 8 Department of Neurology, Erasmus MC, Rotterdam, The Netherlands (M.A.I.); Netherlands; 9 Consortium for Healthy Aging, Leiden, The Netherlands; 10 Mel and Enid Zuckerman College of Public Health, University of Arizona, Tucson, Arizona, United States of America; 11 Department of Medicine (Geriatrics), University of Mississippi Medical Center, Jackson, Mississippi, United States of America; 12 Department of Neurology, Johns Hopkins University School of Medicine, Baltimore, Maryland, United States of America; 13 Human Genome Sequencing Center, Baylor College of Medicine, Houston, Texas, United States of America; 14 Department of Epidemiology, University of Washington, Seattle, Washington, United States of America; 15 Group Health Research Institute, Group Health, Seattle, Washington, United States of America; 16 Department of Statistics, University of Auckland, Auckland, New Zealand; 17 Department of Neurology, Boston University School of Medicine, Boston, Massachusetts, United States of America; 18 Department of Neurology, University of Washington, Seattle, Washington, United States of America; 19 Brown Foundation Institute of Molecular Medicine, University of Texas Health Science Center at Houston, Texas, United States of America; Inrca, Italy

## Abstract

**Background:**

Stroke, the leading neurologic cause of death and disability, has a substantial genetic component. We previously conducted a genome-wide association study (GWAS) in four prospective studies from the Cohorts for Heart and Aging Research in Genomic Epidemiology (CHARGE) consortium and demonstrated that sequence variants near the *NINJ2* gene are associated with incident ischemic stroke. Here, we sought to fine-map functional variants in the region and evaluate the contribution of rare variants to ischemic stroke risk.

**Methods and Results:**

We sequenced 196 kb around *NINJ2* on chromosome 12p13 among 3,986 European ancestry participants, including 475 ischemic stroke cases, from the Atherosclerosis Risk in Communities Study, Cardiovascular Health Study, and Framingham Heart Study. Meta-analyses of single-variant tests for 425 common variants (minor allele frequency [MAF] ≥ 1%) confirmed the original GWAS results and identified an independent intronic variant, rs34166160 (MAF = 0.012), most significantly associated with incident ischemic stroke (HR = 1.80, p = 0.0003). Aggregating 278 putatively-functional variants with MAF≤ 1% using count statistics, we observed a nominally statistically significant association, with the burden of rare *NINJ2* variants contributing to decreased ischemic stroke incidence (HR = 0.81; p = 0.026).

**Conclusion:**

Common and rare variants in the *NINJ2* region were nominally associated with incident ischemic stroke among a subset of CHARGE participants. Allelic heterogeneity at this locus, caused by multiple rare, low frequency, and common variants with disparate effects on risk, may explain the difficulties in replicating the original GWAS results. Additional studies that take into account the complex allelic architecture at this locus are needed to confirm these findings.

## Introduction

Stroke is the leading neurologic cause of death and disability.[1] Twin and familial aggregation studies suggest that the risk of stroke has a substantial genetic component[2]–[4], but few genes underlying this risk in the general population have been elucidated. Previously, we conducted a genome-wide association study in four prospective cohorts comprising the Cohorts for Heart and Aging in Genomic Epidemiology (CHARGE) consortium that identified and replicated associations of two common single nucleotide polymorphisms (SNPs) with risk of incident ischemic stroke among 19,602 individuals of European ancestry, who suffered 1164 incident ischemic strokes over an average follow-up of 11 years.[5] Both SNPs were in close proximity to *NINJ2*, which encodes ninjurin2, an adhesion molecule expressed in glia that plays a role in neurite growth, ischemic tolerance, and inflammation response, and that may influence how the brain responds to an ischemic insult.[6] These two SNPs were in linkage disequilibrium (LD) with each other (r^2^ = 0.73 based on HapMap CEU data, NCBI build 36) as well as with other variants in the 5′ untranslated region of *NINJ2*. We observed even stronger associations when the analyses were restricted to ischemic strokes of atherothrombotic origin.

Subsequent reports have been conflicting about the association of the *NINJ2* SNPs with ischemic stroke. Independent attempts to replicate these findings in large case-control samples were unsuccessful [7]–[9], although other smaller studies have observed associations of these variants with risk.[10]–[13]

In order to clarify the role of sequence variation in this region in the etiology of incident ischemic stroke, we sequenced a 196 kb region of chromosome 12 that contains the *NINJ2* gene, part of the *WNK1* gene, and their intergenic sequence, among a subsample from 3 cohorts of the CHARGE consortium. Our aim was to detail the landscape of common and rare variation in this region and to identify novel variants underlying associations with ischemic stroke at this locus.

## Methods

### Participating Studies and Study Design

Our analyses were performed as part of the Cohorts for Heart and Aging Research in Genomic Epidemiology Targeted Sequencing Study (CHARGE-S), which aimed at following up GWAS signals for a wide array of cardiovascular related traits to identify functional variants and to evaluate the contribution of rare variants. The CHARGE consortium is a collaborative program of prospective population-based cohorts seeking to identify susceptibility genes for cardiovascular, lung, and blood diseases and their risk factors.[14]

This project focused on a subset of 3,986 participants of European ancestry from the Atherosclerosis Risk in Communities Study (ARIC), the Cardiovascular Health Study (CHS), and the Framingham Heart Study (FHS), and included 229 individuals selected based on their stroke phenotype and representing a targeted subsample of individuals from the genome-wide association study discovery effort. Information about the 3 cohorts' study design and recruitment is included in File S1.

In each of the 3 cohorts, participants with available DNA and consent who experienced an incident ischemic stroke after age 65 were eligible for selection. This sample was enriched for participants in whom incident stroke was of atherothrombotic origin, preferentially selecting those with the earliest onset, with equal numbers of men and women, and in numbers proportional to the sample size of the participating cohorts. In our epidemiological samples, ‘atherothrombotic brain infarction’ is defined as a clinical ischemic stroke consistent with occlusion of an extra-or intracranial artery, that is, a clinical ischemic stroke not suspected to be either of cardioembolic origin or due to an identified non-atherothrombotic etiology (such as arterial dissection). This category includes the subtypes of large artery atherosclerosis, small-artery occlusion (lacunes) and undetermined origin. The atherothrombotic stroke phenotype was selected because it yielded the strongest association with *NINJ2* variants in our previous GWAS meta-analysis.[5] Seventy-one atherothrombotic stroke cases were sequenced in ARIC; 105 in CHS and 53 in FHS.

The remaining participants in this study were selected as part of a Cohort Random Sample or for extreme values of other cardiovascular phenotypes. Among these, 246 also experienced an incident ischemic stroke during study follow-up.

#### Ethics Statement

All subjects provided written and informed consent to participate in genetic studies, and all study sites received approval to conduct this research from their local respective Institutional Review Boards (IRB), including the Committee for the Protection of Human Subjects at the University of Texas Health Science Center at Houston, University of Mississippi Medical Center IRB (ARIC – Jackson Field Center), Wake Forest University Health Sciences IRB (ARIC – Forsyth County Field Center), University of Minnesota IRB (ARIC – Minnesota Field Center), Johns Hopkins University IRB (ARIC and CHS – Washington County Field Centers), Wake Forest University Health Sciences IRB (CHS – Forsyth County Field Center), University of California, Davis IRB (CHS – Sacramento County Field Center), University of Pittsburgh IRB (CHS – Pittsburgh Field Center), and Boston University IRB (FHS).

### Stroke phenotypes

Stroke was defined as a focal neurologic deficit of presumed vascular cause with a sudden onset and lasting for at least 24 hours or until death if the participant died less than 24 hours after the onset of symptoms. Details of stroke surveillance and diagnostic criteria for stroke and stroke types in the three studies have been published [15]–[21] and are summarized in File S1. Strokes were classified as ischemic, hemorrhagic, or unknown type on the basis of clinical and imaging criteria. For this study, we considered only ischemic strokes.

### Sequencing

The methods of the CHARGE Targeted Sequencing Study have been previously described (Lin H, Wang M, Brody JA, Bis JC, Dupuis J, Lumley T, et al., accepted to *Circ Cardiovasc Genet*). Briefly, the Study sequenced a total of 77 target regions that harbor genetic variants associated with 14 phenotypes implicated by GWAS within the CHARGE consortium. In particular, the Neurology working group selected the *NINJ2* gene region from the University of California at Santa Cruz (UCSC) Genome Browser, with the aim of capturing all sequence variation upstream and downstream of the gene.

Approximately 2 Mb of target regions were captured by a customized NimbleGen Capture array and then sequenced using the ABI SOLiD V4.0 platform. The raw short reads were aligned to the reference human genome (NCBI Genome Build 36, hg18) by BFAST.[22] SAMtools[23] was used to pile up aligned reads and call variants with quality filters. The resulting data were then subjected to quality control (QC) procedures, including variant-level and sample-level QC. Detailed QC methods are described in the accompanying CHARGE Targeted Sequencing Study Design manuscript. Variants were categorized as known or novel by comparison with the dbSNP database and the 1000 Genomes Project. Functional annotations were produced using a combination of ANNOVAR,[24] dbNSFP,[25] and custom internal tools.

### Statistical analysis

Each study independently implemented the predefined analysis plan described next and results from the 3 studies were combined by meta-analytic techniques.

In each study, Cox proportional hazard models were used to assess association of variants with incident ischemic stroke. Participants with prevalent stroke were excluded from the analysis. Participants who experienced a stroke not classified as “ischemic” were censored at time of alternative type of stroke. Although atherothrombotic strokes were enriched in the subset of stroke cases selected for sequencing, our primary analyses included all incident ischemic strokes, including those sequenced as part of the Cohort Random Sample or selected for other Phenotype Groups, to maximize sample size since we did not have adequate power to perform analyses restricted only to atherothrombotic strokes. Models were adjusted for age and sex. Additional adjustments included study site for CHS and ARIC, and familial structure for FHS.

For each variant with a minor allele frequency (MAF) ≥ 1% in the combined population, each study fitted additive genetic models, regressing trait on genotype dosage (0 to 2 copies of the variant allele). Meta-analyses of standard regression coefficients [26] were used to determine significance, but we repeated these analyses weighted by each participant's sampling probability to obtain valid estimates of effect size. (Lumley T, Dupuis J, Rice KM, Barbalic M, Bis JC, Cupples LA, et al. http://stattech.wordpress.fos.auckland.ac.nz/files/2012/05/design-paper.pdf).

Our primary hypothesis focused on descriptive analyses of sequence variants in the NINJ2 regions. Given the prior evidence for this region in this sample, we used a p-value threshold corresponding to one expected false discovery among the total number of SNPs tested to identify variants of potential interest (p = 1/425 = 0.002).

The primary analysis for rare variants was to aggregate variants of MAF <1% and with predicted functional changes on encoded proteins[24] or gene regulation, into a T1 count statistic, defined as the sum of the number of variant sites in the target at which a person has at least one rare allele with MAF <1%. We annotated variants using a heuristic scoring system implemented in RegulomeDB, which represents the confidence that a variant has a functional impact on gene regulation.[27] The burden of variants of MAF <1% with predicted functional impact on proteins (amino-acid change) and on gene regulation (RegulomeDB score ≤3) was evaluated for association with incident ischemic stroke.

To explore the possibility that rare variants within a gene did not have the same direction or magnitude of association, we also implemented the Sequence Kernel Association Test (SKAT)[28], which approximates the score test that would be obtained fitting a model that includes all the variants, using customized R scripts for meta-analyses. (Lumley T, Brody J, Dupuis J, Cupples LA http://stattech.wordpress.fos.auckland.ac.nz/files/2012/11/skat-meta-paper.pdf).

## Results

Our analysis included 3,986 participants who successfully completed targeted sequencing. Characteristics of these participants from the 3 participating cohorts are shown in Table 1 and in the Supplemental Material (Table S1 in File S1). In general, participants selected for sequencing had their stroke event early in the follow-up period, except in ARIC where events were uniformly distributed across the follow-up period. Age at stroke onset was similar across the 3 cohorts.

**Table 1:** Characteristics of study participants.

	ARIC	CHS	FHS
Sample size	1885	1131	970
Ischemic stroke, n	189	217	69
Atherothrombotic stroke, n	153 (71*)	167 (105*)	58 (53*)
Female, %	49% (39%#)	53.7% (54.8%#)	51.6% (51.2%#)
Mean baseline age, y	54.8 (58.5#)	72.5 (72.5#)	62.9 (75.4#)
Mean follow-up, y	18.2 (11.8#)	11.9 (7.4#)	8.6 (3.5#)

*Number of atherothrombotic stroke cases originally selected for resequencing.

# In ischemic stroke cases only.

Resequencing of the *NINJ2* region on chromosome 12p13 between base pairs 543,643 and 740,130 (NCBI Build 36, 2006) on 3,986 individuals identified 4,001 variants, including 3,077 not previously identified in the 1000 Genomes Project (Table 2). Twenty-eight were coding variants. Across all SNPs in the *NINJ2* target region, the average 2.5% coverage percentile was 37.8X and the 97.5% percentile was 45.4X, indicating excellent sequence coverage of the *NINJ2* gene region.

**Table 2:** Characteristics of variants in the *NINJ2* targeted region.

	Common	Rare
	(MAF ≥ 0.01)	(MAF <0.01)
Nonsynonymous	0	18
Synonymous	1	9
Intergenic	135	1,459
Intron	280	2,031
Upstream	5	48
3′ Untranslated Region	3	7
5′ Untranslated Region	1	4
Total	425	3,576

Our primary analyses of the *NINJ2* locus focused on 425 individual common variants and aggregated tests of 278 rare putatively-functional variants. Genomic annotation for these variants is shown in Table S2 in File S1.

### Common Variants Results

**Figure 1:**
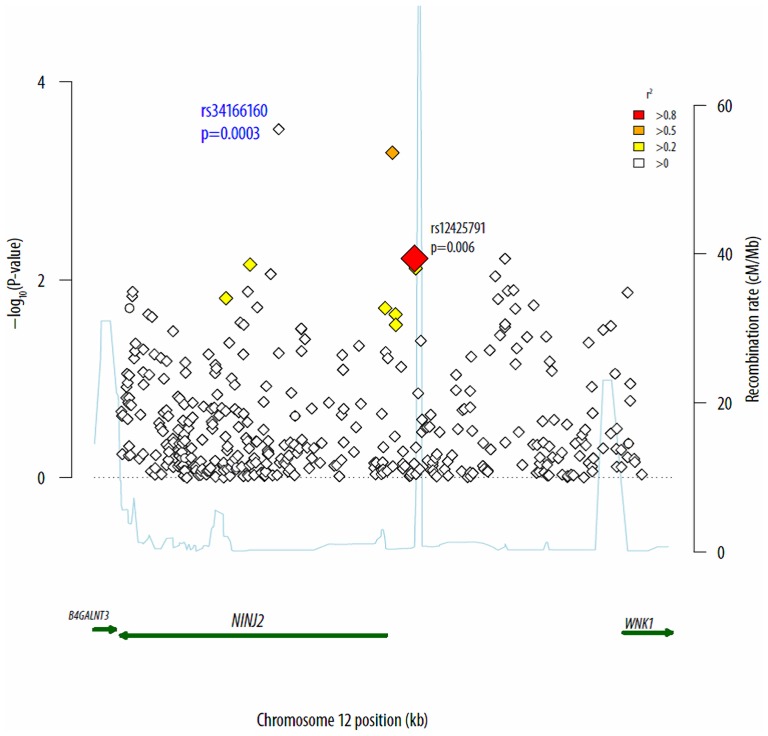
Associations of common variants (MAF≥1%) with incident ischemic stroke in the CHARGE Targeted Sequencing Study. Association p-values are plotted against their genomic position.

Figure 1 displays a regional association plot showing the results for the meta-analysis of common variants. We confirmed the association of the previously-reported sentinel GWAS SNP rs11833579 (Hazard Ratio (HR) = 1.39, p = 0.0005) with incident ischemic stroke in this smaller targeted sample. The second GWAS SNP, rs12425791, was only borderline significant based on our threshold of one expected false discovery (HR = 1.31, p = 0.006). However, the most significant association in the meta-analysis of common *NINJ2* variants was an intronic SNP, rs34166160 (MAF = 0.012, HR = 1.80, p = 0.0003), in the *NINJ2* gene, which was in low LD (r^2^ = 0.02) with the sentinel GWAS SNPs. When we repeated the analysis with adjustment for rs12425791, the association was slightly attenuated (HR = 1.66, p = 0.002).

We annotated variants in LD (r^2^ >0.8) with the sentinel GWAS SNPs for predicted functional impact on gene regulation using a heuristic score metric implemented in RegulomeDB. We identified 2 variants, rs7297967 (MAF = 0.45; RegulomeDB score = 1f) and rs3782851 (MAF = 0.06; RegulomeDB score = 3a), with predicted functional impact on *NINJ2* gene regulation. rs7297967 was classified as likely to affect transcription factor binding and was shown to be associated with *NINJ2* gene expression levels [29], while rs3782851 was less likely to affect transcription factor binding. In the targeted sequencing sample, these 2 SNPs showed nominal associations with incident ischemic stroke (p = 0.022 and 0.019, respectively) and modest effect sizes (HR = 1.18 and 1.45, respectively). As expected, these associations were no longer significant after adjusting for rs12425791 (p = 0.42 and 0.34, respectively).

We next examined the association of these 3 SNPs in an independent sample of 6,066 participants from the Rotterdam Study (mean follow-up: 12 years; 60% females; mean baseline age: 69 years), which included 353 incident atherothrombotic stroke cases. rs3782851 was directly genotyped, while rs34166160 and rs7297967 were imputed from the 1000 Genomes (Phase 1, V3) CEU reference panel. Imputation quality for both SNPs was excellent (Rsq>0.99). Estimated allele frequencies of rs7297967 and rs3782851 in the Rotterdam sample were similar to those in the CHARGE sample (MAF = 0.42 and 0.06, respectively), but rs34166160 was much less frequent (MAF = 0.006). rs7297967 was nominally associated with incident ischemic stroke risk (HR = 1.18; p = 0.03) but rs3782851 and rs34166160 were not (HR = 1.18; p = 0.27 and HR = 2.3; p = 0.20, respectively). However, power to detect effect sizes similar to those observed in CHARGE in this Rotterdam sample was low to moderate for these two SNPs (rs3782851, power = 0.68; rs34166160, power = 0.23).

### Rare Variants Results

**Figure 2:**
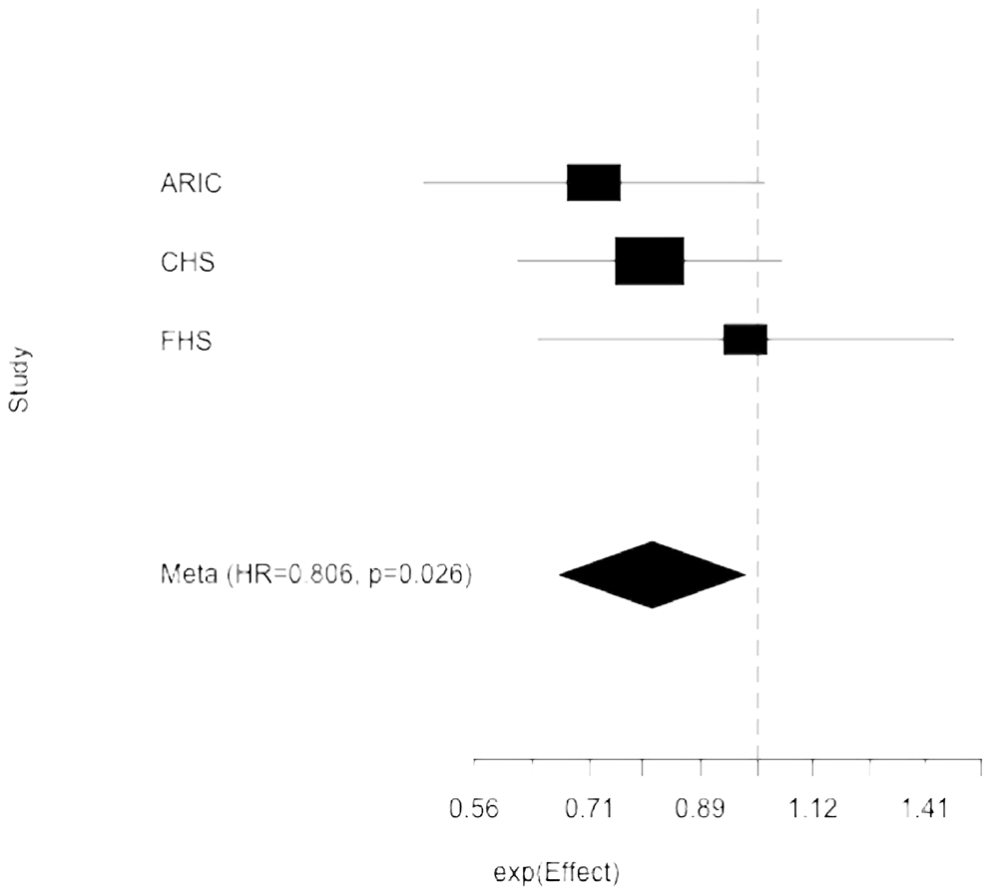
T1 test of association of rare variants with predicted functional impact on protein function or gene regulation in the 3 cohorts. Shown are the hazard ratios (HR) and associated confidence intervals for each cohort and the summary measure (diamond) from the meta-analysis.

We performed a single burden test collapsing 278 variants of MAF ≤1%, which also had a potential impact on protein function or gene regulation. These included 268 variants with RegulomeDB score ≤3 and 10 coding variants within the *NINJ2* exons. These variants, in aggregate, were modestly associated with lower stroke incidence (HR = 0.81; p = 0.026) (Figure 2).

Similar results were obtained in a secondary gene-based analysis using a SKAT test. This test can be more powerful in situations where multiple variants have different directions and/or magnitude of effects within the *NINJ2* region. Jointly modeling the effects of the 278 putatively-functional variants with MAF≤ 1%, we observed a nominally significant association with incident ischemic stroke (p = 0.03). This association was only slightly attenuated when adjusting for rs12425791 (p = 0.04).

## Discussion

We sequenced a 196-kb region around the *NINJ2* gene in 3,986 participants from the CHARGE consortium and demonstrated evidence of association between newly-characterized, low frequency and rare sequence variants and ischemic stroke. In the meta-analysis of variants with MAF≥1%, we confirm an association for the original GWAS SNPs in this smaller targeted sample. Only one novel intronic *NINJ2* variant, rs34166160, showed an independent association with incident ischemic stroke. In addition, burden test-based analysis of rare variants across the *NINJ2* region showed modest evidence that, in aggregate, rare variants in this gene were also associated with ischemic stroke incidence, but they appeared to mitigate stroke risk.

Although theoretical models have demonstrated that GWAS findings can reflect the contributions of one or more uncommon or rare variants, empirical data reporting such synthetic associations remain sparse, especially for complex disorders.[30] The variant rs34166160, located in intron 1 of *NINJ2*, showed the strongest association with incident ischemic stroke, which was slightly attenuated after accounting for the effects of the GWAS sentinel SNP rs12425791, suggesting possible allelic heterogeneity at this locus. This association was however not replicated in an independent sample, likely due to low power. Functional annotation of rs34166160 using the ENCODE data indicated that this variant is located in a region of open chromatin as determined from DNaseI hypersensitivity and Formaldehyde-Assisted Isolation of Regulatory Elements assays.[31] However, there was minimal evidence that this variant disrupts transcription factor binding [27], and no evidence of evolutionary constraint on this variant (GERP score <0). rs34166160 was associated with a modest increase in ischemic stroke incidence (HR = 1.80). The effects of rs11833579 and rs12425791 on ischemic stroke risk were even more modest, with HRs of 1.39 and 1.31, respectively, but were similar to those reported in our genome-wide association study.[5]

Rare variants in this region, in aggregate, also influenced stroke incidence. In particular, cumulative burden of rare alleles that affect *NINJ2* gene regulation or function was associated with a lower stroke incidence. These data highlight the complex relationship between sequence variation in the *NINJ2* gene and ischemic stroke susceptibility.

Allelic heterogeneity at this locus, caused by multiple rare, low frequency, and common variants with disparate effects on risk, was suggested by analyses conditioning on the sentinel GWAS SNP and showing only mild attenuation of effects. If confirmed, this may help explain the conflicting findings of studies seeking to replicate the original GWAS results. Ninjurin 2 is a broadly expressed homophilic adhesion molecule. It is involved in neuronal growth, plays a role in nerve regeneration, and may affect how the brain tolerates cerebral ischemic insults. Thus, variants that affect *NINJ2* regulation or function may influence stroke risk, either favorably or unfavorably.

Despite the large number of novel variants identified at the *NINJ2* locus, the causal set of variants underlying association with stroke incidence remains unclear. Only few variants had a potentially functional impact on the encoded gene product, and none of them was common. Annotation of variants with predicted functional impact on gene regulation using RegulomeDB identified 268 variants with MAF< 1%. Additional studies will be needed to understand the mechanism(s) by which, in aggregate, they influence stroke risk. Among the common variants, we identified 2 novel variants, rs7297967 and rs3782851, with predicted functional impact on *NINJ2* gene regulation and that were in LD with the sentinel GWAS SNPs. rs7297967 has previously been associated with *NINJ2* gene expression[29] and maps to a region binding transcription factors and encompassing a DNAseI footprint.[27] rs3782851 was also predicted to affect transcription factor binding but with lower confidence due to a more incomplete set of evidence. These 2 SNPs were only nominally associated with stroke risk and had modest effect sizes. Attempt at an independent replication of these variants' associations in Rotterdam Study showed that rs7297967 was associated with incident atherothrombotic stroke, with effects of similar magnitude as that observed in the CHARGE sample.

Several limitations of our study must be acknowledged: First, because of our limited sample size, we likely had little power to detect functional variants with weak to moderate effects on ischemic stroke risk and, thus, we may have failed to identify true genetic associations. Second, we only considered single nucleotide variants as a source of DNA sequence variation in our study and did not investigate the role of copy number variants or other structural variants. Third, the enrichment of our ischemic stroke cases with the atherothrombotic stroke subtype did not distinguish between individual subtypes such as those related to large and small vessel disease. Because of the small sample size, we did not perform analyses limited to specific ischemic subtypes. Possible differences in the strength of association between *NINJ2* variation and specific ischemic stroke etiologies may further erode our power to detect true effects. Fourth, although characterization of the regions of transcription, transcription factor association, chromatin structure and histone modification in the human genome sequence is rapidly progressing[32], our ability to predict function of sequence variants in this region remains imperfect and, thus, further limited our ability to identify functional variants and, hence, to fully capture their impact on stroke risk. Finally, independent replication of findings was hampered by the limited availability of sequence data on prospective cohort studies of incident stroke. Replication is especially challenging for gene-based associations and this limitation is magnified in the absence of precise knowledge of functionally important regulatory gene regions, which further erodes power.[33] Thus, definitive evidence of an association of *NINJ2* with risk for ischemic stroke cannot not be firmly established.

## Conclusion

In conclusion, resequencing of a 196-kb region around the *NINJ2* gene in 3,986 European-American participants of 3 prospective cohorts of the CHARGE consortium identified novel associations of both common and rare variants with incident ischemic stroke. While single common variants were associated with increased ischemic stroke incidence, rare variants, in aggregate, were associated with decreased stroke risk. These data highlight the complexity of the genetic architecture underlying the association of *NINJ2* with ischemic stroke risk. Additional studies that take into account the complex allelic architecture at the *NINJ2* locus will be needed to confirm and extend these novel findings.

## Supporting Information

File S1 Supplemental Materials.
(PDF)
