# Supplementary material for: Associations of NINJ2 Sequence Variants with Incident Ischemic Stroke in the Cohorts for Heart and Aging in Genomic Epidemiology (CHARGE) Consortium
Source: PLoS One. 2014 Jun 24;9(6):e99798. doi: 10.1371/journal.pone.0099798 (PMC4069013; doi:10.1371/journal.pone.0099798)
Supplement: File S1 — Supplemental Materials. (PDF) [file pone.0099798.s001.pdf]

SUPPLEMENTAL MATERIALS:

Associations of NINJ2 Sequence Variants with Incident Ischemic Stroke in the Cohorts for Heart and Aging in Genomic Epidemiology (CHARGE) Consortium

Bis et al.

Section 1: Study descriptions

Our analyses were performed within the Cohorts for Heart and Aging Research in Genomic Epidemiology (CHARGE) consortium,[1] the individual studies contributing to this meta-analysis have been described in detail elsewhere. A brief overview follows.

The Atherosclerosis Risk in Communities Study (ARIC):

The ARIC study is a multi-center prospective investigation of atherosclerotic disease in a predominantly bi-racial population[2]. Men and women aged 45-64 years at baseline were recruited from 4 communities: Forsyth County, North Carolina; Jackson, Mississippi; suburban areas of Minneapolis, Minnesota; and Washington County, Maryland. A total of 15,792 individuals participated in the baseline examination in 1987-1989, with follow-up examinations in approximate 3-year intervals, during 1990-1992, 1993-1995, and 1996-1998.

The Cardiovascular Health Study (CHS):

The CHS is a population-based cohort study of risk factors for CHD and stroke in adults ≥65 years conducted across four field centers in the United States. [3] The original predominantly Caucasian cohort of 5201 persons was recruited in 1989-1990 from a random sample of people on Medicare eligibility lists and an additional 687 African-Americans were enrolled subsequently for a total sample of 5,888.
Because the focus of the targeted sequencing project was the follow-up of findings from genomewide studies conducted among European ancestry individuals, the African-American participants were excluded from this analysis.

The Framingham Heart Study (FHS):

The methods of recruitment and data collection have been described previously for the original Framingham Heart Study cohort (5,209 participants ascertained systematically from two-thirds of the households in the town of Framingham, MA, beginning in 1948),[4] the Framingham Heart Study Offspring cohort (5,124 children of the original cohort, and spouses of those children, beginning in 1972[5] and the Third Generation cohort (4,095 children of the Offspring cohort, beginning in 2002).[6] Data from the Original and Offspring cohort participants selected for the targeted sequencing were utilized in this study but data from Gen 3 participants were not included since they are young (mean age 40±9 years) and very few have suffered

strokes. The Framingham Heart Study was approved by the institutional review boards of Boston University and the National Institutes of Health. All participants provided written informed consent.

Section 2: Stroke Surveillance and Ascertainment

ARIC:

For the present study, we included stroke events occurring between ARIC visit 1 and December 31, 2009. TIAs were not ascertained. All participants were contacted annually by phone, and all hospitalizations and deaths in the previous year were identified. Hospital records for any hospitalizations identified were then obtained. In addition, all local hospitals annually provided lists of stroke discharges (International Classification of Diseases, Ninth Revision, Clinical Modification codes 430 to 438), which were scrutinized for ARIC participant discharges. Details on quality assurance for ascertainment and classification of stroke are described elsewhere.[7] Out-of-hospital stroke was not ascertained and validated. Briefly, the stroke diagnosis was assigned according to criteria adapted from the National Survey of Stroke.[8] Strokes secondary to trauma, neoplasm, hematologic abnormality, infection, or vasculitis were excluded, and a focal deficit lasting <24 hours was not considered to be a stroke. Qualifying strokes were further classified into definite or probable hospitalized ischemic (cardioembolic or thrombotic) or hemorrhagic stroke on the basis of neuroimaging studies and autopsy, when available. A stroke was classified as ischemic when a brain CT or MRI revealed acute infarction and showed no evidence of hemorrhage. For this analysis, the hemorrhagic strokes identified by ARIC were censored at the time of their occurrence. Participants with definite (N=41) or probable (N=30) thrombotic strokes after age 65 were selected for sequencing. Data analyses included all definite and probable incident ischemic strokes (N=189), including those sequenced as part of the Cohort Random Sample (N=54) and other case or extreme phenotype groups (N=64). Among these, 153 were classified as definite or probable thrombotic stroke events. Basic characteristics of the sample by sequencing subgroup are shown in the Table below.

CHS:

Participants were examined annually from enrollment to 1999, and since then continue to be under surveillance for stroke.[10, 11] Since baseline, participants have also been contacted twice a year to identify potential cardiovascular events, including stroke. In addition, all hospitalizations were screened for potential stroke events. For suspected events, information was collected from the participant or next of kin, from medical records, and, if needed, from the participant's physician. When available, CT and/or MRI scans or reports were reviewed centrally. Final adjudication of the occurrence of stroke, stroke types, and subtypes was undertaken by vascular neurologists at a consensus conference using all available information.

For this analysis, hemorrhagic strokes and strokes of unknown subtype identified by CHS were censored at the time of their occurrence. Ischemic strokes with atherosclerotic evidence of large vessel disease were considered “definite atherothrombotic”. In the absence of a specific mechanism, an ischemic stroke without cardioembolic or other subtype information were

considered "probable atherothrombotic." Participants with definite (N=26) or probable (N=79) thrombotic strokes were selected for sequencing. Data analyses included all definite and probable incident ischemic strokes (N=217), including those sequenced as part of the Cohort Random Sample (N=34) and other case or extreme phenotype groups (N=78). Among these, 167 were classified as definite or probable thrombotic stroke events. Basic characteristics of the sample by sequencing subgroup are shown in the Table below.

FHS:
At each clinic exam, participants receive questionnaires, physical examinations and laboratory testing; between examinations they remain under surveillance (regardless of whether or not they live in the vicinity) via physician referrals, record linkage and annual telephone health history updates. Incident strokes have been identified since 1948 through this ongoing system of FHS clinic and local hospital surveillance and methods used have been detailed previously; [12-14] they include review of medical records and collaboration with local general practitioners, emergency rooms and imaging facilities. If a participant saw a physician or was admitted to the hospital, visited an emergency room or obtained any brain imaging between biennial examinations for symptoms suggestive of TIA or stroke, a stroke neurologist from the Heart Study attempted to visit the person within 48 hours and recorded a complete history and neurological examination; this was repeated at 1, 3 and 6 months. All medical records from practitioners, hospitals, imaging centers, rehabilitation centers and nursing homes were procured for review. A panel of 3 investigators (at least 2 neurologists) adjudicated the diagnosis of stroke and determined stroke subtype in each case based on the Framingham evaluations and external records. The recruitment of Original and Offspring cohort participants at FHS had occurred long before the DNA collection with the result that a large number of stroke events in the FHS (although ascertained prospectively) were prevalent at the time of DNA collection and were excluded from these analyses. While this reduced the sample size from FHS, the meta-analyses presented here focused on incident events. All participants with available DNA and consent and incident ischemic stroke (excluding cardioembolic events) were eligible for selection. This phenotype, which included both large and small artery atherothrombotic strokes had yielded the largest hazard ratio in the original CHARGE GWAS. We also excluded participants who had been selected for whole exome sequencing as part of an alternative (GO-ESP) project. From among all eligible individuals meeting these criteria we selected the earliest strokes with onset past age 65, and attempted to select equal numbers of men and women (as had been done for the control sample). Since we did not have sufficient events among men age>65, the Framingham sample included 19 men who had their stroke between the ages of 45 and 65 as well as an additional 5 men and 29 women older than 65 years at the time of their initial stroke. Data analyses also included additional ischemic strokes sequenced as part of the Cohort Random Sample (N=16).

Table S1. Characteristics of the study sample

	Subgroup	Sample Size (N)	Stroke (N)	Follow-up (mean)	Female (%)	Baseline Age (mean)
ARIC	Stroke Cases	71	71	13 years	48%	61 years
	Random Cohort	893	54	19 years	49%	54 years
	Other Extremes	921	64	18 years	49%	55 years
CHS	Stroke Cases	105	105	6 years	50%	69 years
	Random Cohort	358	34	13 years	51%	73 years
	Other Extremes	668	78	12 years	55%	73 years
FHS	Stroke Cases	53	53	5 years	55%	75 years
	Random Cohort	494	16	9 years	50%	62 years
	Other Extremes	423	0	9 years	54%	63 years
	Total N	3669				

References

1. Psaty BM, O'Donnell CJ, Gudnason V, Lunetta KL, Folsom AR, Rotter JI, et al. Cohorts for Heart and Aging Research in Genomic Epidemiology (CHARGE) Consortium: Design of Prospective Meta-Analyses of Genome-Wide Association Studies From 5 Cohorts. *Circ Cardiovasc Genet*. 2009;2:73-80

2. The Atherosclerosis Risk in Communities (ARIC) Study: design and objectives. The ARIC investigators. *Am J Epidemiol*. 1989;129:687-702

3. Fried LP, Borhani NO, Enright P, Furberg CD, Gardin JM, Kronmal RA, et al. The Cardiovascular Health Study: design and rationale. *Ann Epidemiol*. 1991;1:263-276

4. Dawber TR, Kannel WB. The Framingham study. An epidemiological approach to coronary heart disease. *Circulation*. 1966;34:553-555

5. Kannel WB, Feinleib M, McNamara PM, Garrison RJ, Castelli WP. An investigation of coronary heart disease in families. The Framingham offspring study. *Am J Epidemiol*. 1979;110:281-290

6. Splansky GL, Corey D, Yang Q, Atwood LD, Cupples LA, Benjamin EJ, et al. The Third Generation Cohort of the National Heart, Lung, and Blood Institute's Framingham Heart Study: design, recruitment, and initial examination. *Am J Epidemiol*. 2007;165:1328-1335

7. Rosamond WD, Folsom AR, Chambless LE, Wang CH, McGovern PG, Howard G, et al. Stroke incidence and survival among middle-aged adults: 9-year follow-up of the Atherosclerosis Risk in Communities (ARIC) cohort. *Stroke; a journal of cerebral circulation*. 1999;30:736-743

8. The National Survey of Stroke. National Institute of Neurological and Communicative Disorders and Stroke. *Stroke; a journal of cerebral circulation*. 1981;12:I1-91

9. Ay H, Furie KL, Singhal A, Smith WS, Sorensen AG, Koroshetz WJ. An evidence-based causative classification system for acute ischemic stroke. *Annals of neurology*. 2005;58:688-697

10. Longstreth WT, Jr., Bernick C, Fitzpatrick A, Cushman M, Knepper L, Lima J, et al. Frequency and predictors of stroke death in 5,888 participants in the Cardiovascular Health Study. *Neurology*. 2001;56:368-375

11. Price TR, Psaty B, O'Leary D, Burke G, Gardin J. Assessment of cerebrovascular disease in the Cardiovascular Health Study. *Ann Epidemiol*. 1993;3:504-507.

12. Carandang R, Seshadri S, Beiser A, Kelly-Hayes M, Kase CS, Kannel WB, et al. Trends in incidence, lifetime risk, severity, and 30-day mortality of stroke over the past 50 years. *JAMA : the journal of the American Medical Association*. 2006;296:2939-2946

13. Seshadri S, Beiser A, Kelly-Hayes M, Kase CS, Au R, Kannel WB, et al. The lifetime risk of stroke: estimates from the Framingham Study. *Stroke; a journal of cerebral circulation*. 2006;37:345-350

14. Wolf PA, Kannel WB, Dawber TR. Prospective investigations: the Framingham study and the epidemiology of stroke. *Advances in neurology*. 1978;19:107-120

Table S2. Sequence variants identified in the NINJ2 region

CHR: chromosome

pos_hg19: SNP position (GRCh37/hg19 reference)

SNP: SNP ID

POS: SNP position (Human Genome NCBI36/hg18 reference)

REF: Reference allele

ALT: Alternative allele

AAF: Alternative allele frequency

RAF: Reference allele frequency

MAF: Minor allele frequency

mean MAPQ: Mean MAP Quality score (Coverage)

mean SNPQ: Mean SNP Quality score

minHWE exact: Hardy Weinberg Equilibrium

mean nREADS.025: average 2.5% coverage percentile

mean nREADS.975: average 97.5% coverage percentile

mean missing rate: mean missing rate

CHR	pos_hg19	snp	POS	REF	ALT	AAF	RAF	MAF	mean MAPQ	mean SNPQ	minHWE exact	mean nREADS.025	mean nREADS.975	mean Missing Rate	Ancestral _allele
12	673515	chr12:543776	543776	T	C	0.07704	0.92296	0.07704	60.00	220.03	0.4984	17.32	60.12	0.0367	C
12	673540	chr12:543801	543801	G	T	0.00039	0.99961	0.00039	60.00	228.00	1.0000	27.00	27.00	0.0000	G
12	673572	chr12:543833	543833	C	T	0.00031	0.99969	0.00031	60.00	228.00	1.0000	31.16	31.16	0.0303	C
12	673573	chr12:543834	543834	G	A	0.00267	0.99733	0.00267	60.00	221.81	1.0000	16.61	42.54	0.0288	G
12	673583	chr12:543844	543844	G	A	0.00027	0.99973	0.00027	60.00	45.00	1.0000	10.00	10.00	0.0609	G
12	673639	chr12:543900	543900	C	T	0.00031	0.99969	0.00031	60.00	228.00	1.0000	46.27	46.27	0.0271	C
12	673652	chr12:543913	543913	C	T	0.00026	0.99974	0.00026	60.00	228.00	1.0000	30.00	30.00	0.0419	C
12	673704	chr12:543965	543965	T	A	0.07217	0.92783	0.07217	60.00	215.58	0.3319	16.86	55.22	0.0373	A
12	673751	chr12:544012	544012	G	T	0.00048	0.99952	0.00048	60.00	228.00	1.0000	27.00	27.00	0.0742	G
12	673788	chr12:544049	544049	A	G	0.05795	0.94205	0.05795	60.00	194.06	0.7120	11.72	41.11	0.0663	A
12	673854	chr12:544115	544115	A	G	0.76567	0.23433	0.23433	60.00	141.71	0.0112	12.30	48.11	0.0673	G
12	673925	chr12:544186	544186	G	A	0.00663	0.99337	0.00663	60.00	224.65	1.0000	12.60	38.60	0.0108	G
12	673969	chr12:544230	544230	G	A	0.00155	0.99845	0.00155	60.00	126.00	1.0000	13.08	19.63	0.0054	G
12	673986	chr12:544247	544247	C	A	0.00047	0.99953	0.00047	60.00	169.00	1.0000	30.00	30.00	0.0601	C
12	674025	chr12:544286	544286	G	T	0.00029	0.99971	0.00029	59.00	86.00	1.0000	11.00	11.00	0.1343	G
12	674038	chr12:544299	544299	C	T	0.00029	0.99971	0.00029	60.00	68.00	1.0000	14.00	14.00	0.1398	C
12	674059	chr12:544320	544320	G	C	0.00027	0.99973	0.00027	60.00	228.00	1.0000	20.00	20.00	0.0844	G
12	674078	chr12:544339	544339	A	G	0.00046	0.99954	0.00046	60.00	152.00	1.0000	10.00	10.00	0.0327	A
12	674099	chr12:544360	544360	C	A	0.15878	0.84122	0.15878	60.00	206.61	0.0913	12.80	47.17	0.0946	C
12	674107	chr12:544368	544368	C	T	0.00039	0.99961	0.00039	60.00	228.00	1.0000	35.00	35.00	0.0000	C
12	674137	chr12:544398	544398	C	T	0.00031	0.99969	0.00031	60.00	228.00	1.0000	21.00	21.00	0.1862	C
12	674164	chr12:544425	544425	A	G	0.00095	0.99905	0.00095	60.00	227.50	1.0000	11.28	21.73	0.0654	A
12	674177	chr12:544438	544438	G	C	0.00063	0.99937	0.00063	60.00	228.00	1.0000	26.68	38.72	0.0933	G
12	674179	chr12:544440	544440	G	A	0.00030	0.99970	0.00030	60.00	144.00	1.0000	12.00	12.00	0.1807	G
12	674208	chr12:544469	544469	G	C	0.00039	0.99961	0.00039	60.00	228.00	1.0000	24.00	24.00	0.0015	G
12	674269	chr12:544530	544530	C	T	0.00039	0.99961	0.00039	60.00	228.00	1.0000	38.00	38.00	0.0000	C
12	674277	chr12:544538	544538	C	G	0.00036	0.99964	0.00036	60.00	191.06	1.0000	20.92	20.92	0.1090	C
12	674312	chr12:544573	544573	C	T	0.00046	0.99954	0.00046	60.00	228.00	1.0000	46.00	46.00	0.0424	C
12	674412	chr12:544673	544673	T	A	0.00048	0.99952	0.00048	60.00	228.00	1.0000	18.00	18.00	0.0795	T
12	674450	chr12:544711	544711	G	T	0.00046	0.99954	0.00046	60.00	228.00	1.0000	30.00	30.00	0.0300	G
12	674486	chr12:544747	544747	G	A	0.00095	0.99905	0.00095	60.00	228.00	1.0000	24.40	46.98	0.0487	G

12	674504	chr12:544765	544765	G	T	0.00045	0.99955	0.00045	60.00	228.00	1.0000	20.00	20.00	0.0177	G
12	674530	chr12:544791	544791	T	C	0.00029	0.99971	0.00029	60.00	47.00	1.0000	10.00	10.00	0.1443	T
12	674547	chr12:544808	544808	C	T	0.00037	0.99963	0.00037	60.00	228.00	1.0000	27.14	27.14	0.0761	C
12	674585	chr12:544846	544846	G	C	0.00039	0.99961	0.00039	60.00	228.00	1.0000	42.00	42.00	0.0046	G
12	674605	chr12:544866	544866	G	A	0.00040	0.99960	0.00040	60.00	228.00	1.0000	31.00	31.00	0.0270	G
12	674607	chr12:544868	544868	G	A	0.00040	0.99960	0.00040	60.00	228.00	1.0000	22.00	22.00	0.0255	G
12	674617	chr12:544878	544878	C	T	0.00039	0.99961	0.00039	60.00	228.00	1.0000	20.00	20.00	0.0154	C
12	674629	chr12:544890	544890	C	G	0.00528	0.99472	0.00528	60.00	211.08	1.0000	13.23	38.00	0.0592	C
12	674706	chr12:544967	544967	C	T	0.01934	0.98066	0.01934	60.00	217.67	0.4898	15.87	47.65	0.0398	C
12	674717	chr12:544978	544978	G	A	0.00053	0.99947	0.00053	60.00	187.00	1.0000	13.00	13.00	0.1617	G
12	674733	chr12:544994	544994	A	G	0.00051	0.99949	0.00051	60.00	228.00	1.0000	23.00	23.00	0.1299	A
12	674738	chr12:544999	544999	T	C	0.00039	0.99961	0.00039	60.00	122.00	1.0000	46.00	46.00	0.0031	T
12	674792	chr12:545053	545053	G	A	0.00028	0.99972	0.00028	60.00	208.00	1.0000	19.00	19.00	0.0959	G
12	674815	chr12:545076	545076	G	A	0.00033	0.99967	0.00033	60.00	228.00	1.0000	35.43	35.43	0.0647	G
12	674835	chr12:545096	545096	A	G	0.00039	0.99961	0.00039	60.00	228.00	1.0000	38.00	38.00	0.0000	A
12	674893	chr12:545154	545154	C	T	0.00047	0.99953	0.00047	60.00	228.00	1.0000	20.00	20.00	0.0574	C
12	674895	chr12:545156	545156	G	A	0.00034	0.99966	0.00034	60.00	223.64	1.0000	23.92	23.92	0.0928	G
12	674944	chr12:545205	545205	C	T	0.00221	0.99779	0.00221	60.00	223.72	1.0000	12.96	28.54	0.0650	C
12	674979	chr12:545240	545240	C	T	0.00112	0.99888	0.00112	60.00	212.25	1.0000	17.89	25.83	0.1394	C
12	675001	chr12:545262	545262	C	A	0.00050	0.99950	0.00050	60.00	228.00	1.0000	19.00	19.00	0.1184	C
12	675044	chr12:545305	545305	G	C	0.00041	0.99959	0.00041	60.00	35.00	1.0000	10.00	10.00	0.0494	G
12	675050	chr12:545311	545311	C	T	0.00041	0.99959	0.00041	60.00	224.00	1.0000	14.00	14.00	0.0633	C
12	675126	chr12:545387	545387	G	A	0.00039	0.99961	0.00039	60.00	228.00	1.0000	20.00	20.00	0.0147	G
12	675134	chr12:545395	545395	C	T	0.00039	0.99961	0.00039	60.00	155.00	1.0000	13.00	13.00	0.0077	C
12	675142	chr12:545403	545403	A	G	0.00039	0.99961	0.00039	60.00	228.00	1.0000	33.00	33.00	0.0046	A
12	675152	chr12:545413	545413	G	A	0.00039	0.99961	0.00039	60.00	228.00	1.0000	45.00	45.00	0.0023	G
12	675222	chr12:545483	545483	C	T	0.00039	0.99961	0.00039	60.00	201.00	1.0000	15.00	15.00	0.0023	C
12	675239	chr12:545500	545500	C	T	0.00033	0.99967	0.00033	60.00	228.00	1.0000	21.23	21.23	0.0763	C
12	675240	chr12:545501	545501	G	A	0.00147	0.99853	0.00147	60.00	220.42	1.0000	17.14	25.50	0.0748	G
12	675249	chr12:545510	545510	G	A	0.00054	0.99946	0.00054	60.00	228.00	1.0000	20.67	31.90	0.1157	G
12	675298	chr12:545559	545559	T	A	0.00031	0.99969	0.00031	60.00	228.00	1.0000	28.47	28.47	0.0259	T
12	675316	chr12:545577	545577	G	A	0.00026	0.99974	0.00026	60.00	228.00	1.0000	14.00	14.00	0.0305	G
12	675339	chr12:545600	545600	T	C	0.01197	0.98803	0.01197	60.00	212.45	1.0000	15.78	54.18	0.0192	T
12	675366	chr12:545627	545627	C	G	0.00046	0.99954	0.00046	60.00	226.79	1.0000	28.02	30.90	0.0048	C

12	675424	chr12:545685	545685	T	A	0.00025	0.99975	0.00025	60.00	228.00	1.0000	30.00	30.00	0.0030	t
12	675426	chr12:545687	545687	T	A	0.00025	0.99975	0.00025	60.00	228.00	1.0000	32.00	32.00	0.0020	T
12	675441	chr12:545702	545702	T	A	0.34883	0.65117	0.34883	60.00	212.62	0.1174	23.81	76.01	0.0072	A
12	675508	chr12:545769	545769	G	A	0.00113	0.99887	0.00113	60.00	228.00	1.0000	41.98	54.05	0.0009	G
12	675515	chr12:545776	545776	C	T	0.00039	0.99961	0.00039	60.00	228.00	1.0000	63.00	63.00	0.0000	C
12	675519	chr12:545780	545780	T	C	0.00030	0.99970	0.00030	60.00	228.00	1.0000	42.50	42.50	0.0009	T
12	675579	chr12:545840	545840	C	G	0.00039	0.99961	0.00039	60.00	228.00	1.0000	93.00	93.00	0.0000	C
12	675586	chr12:545847	545847	C	T	0.00062	0.99938	0.00062	60.00	228.00	1.0000	52.00	65.19	0.0000	C
12	675603	chr12:545864	545864	C	T	0.01490	0.98510	0.01490	60.00	226.55	1.0000	33.27	80.64	0.0005	C
12	675641	chr12:545902	545902	A	G	0.00039	0.99961	0.00039	60.00	228.00	1.0000	59.00	59.00	0.0000	A
12	675666	chr12:545927	545927	A	G	0.00025	0.99975	0.00025	60.00	228.00	1.0000	30.00	30.00	0.0010	A
12	675691	chr12:545952	545952	G	C	0.00030	0.99970	0.00030	60.00	228.00	1.0000	48.00	48.00	0.0012	G
12	675739	chr12:546000	546000	A	T	0.00044	0.99956	0.00044	60.00	228.00	1.0000	43.00	43.00	0.0027	A
12	675790	chr12:546051	546051	G	A	0.00048	0.99952	0.00048	60.00	228.00	1.0000	35.00	35.00	0.0751	G
12	675799	chr12:546060	546060	T	C	0.29446	0.70554	0.29446	60.00	188.58	0.0507	11.00	37.00	0.0108	C
12	675838	chr12:546099	546099	C	G	0.26794	0.73206	0.26794	60.00	187.01	0.0136	10.53	34.61	0.1032	C
12	675889	chr12:546150	546150	G	A	0.00026	0.99974	0.00026	60.00	122.00	1.0000	16.00	16.00	0.0444	G
12	675890	chr12:546151	546151	G	A	0.01393	0.98607	0.01393	60.00	222.20	1.0000	13.20	49.09	0.0569	G
12	675919	chr12:546180	546180	G	A	0.00390	0.99610	0.00390	60.00	203.48	1.0000	10.17	19.88	0.1026	G
12	675920	chr12:546181	546181	C	T	0.18947	0.81053	0.18947	59.98	179.50	0.1641	10.00	23.83	0.0471	C
12	675923	chr12:546184	546184	A	G	0.00030	0.99970	0.00030	60.00	63.00	1.0000	10.00	10.00	0.1658	A
12	675969	chr12:546230	546230	G	A	0.00059	0.99941	0.00059	60.00	219.06	1.0000	15.26	20.93	0.0376	G
12	676025	chr12:546286	546286	A	G	0.00057	0.99943	0.00057	60.00	228.00	1.0000	26.41	27.88	0.0081	A
12	676042	chr12:546303	546303	C	T	0.00039	0.99961	0.00039	60.00	228.00	1.0000	50.00	50.00	0.0000	C
12	676092	chr12:546353	546353	T	A	0.00025	0.99975	0.00025	60.00	105.00	1.0000	14.00	14.00	0.0065	T
12	676116	chr12:546377	546377	T	A	0.30374	0.69626	0.30374	60.00	207.15	0.5362	15.12	61.23	0.0150	A
12	676127	chr12:546388	546388	G	A	0.00068	0.99932	0.00068	60.00	228.00	1.0000	37.92	44.99	0.0036	G
12	676129	chr12:546390	546390	G	A	0.00039	0.99961	0.00039	60.00	228.00	1.0000	55.00	55.00	0.0000	G
12	676209	chr12:546470	546470	C	G	0.19900	0.80100	0.19900	60.00	222.37	0.2828	24.80	76.75	0.0032	C
12	676226	chr12:546487	546487	A	G	0.00044	0.99956	0.00044	60.00	52.00	1.0000	15.00	15.00	0.0009	A
12	676280	chr12:546541	546541	C	T	0.00039	0.99961	0.00039	60.00	228.00	1.0000	81.00	81.00	0.0000	C
12	676333	chr12:546594	546594	C	T	0.00075	0.99925	0.00075	59.33	84.33	1.0000	15.30	27.65	0.0025	C
12	676366	chr12:546627	546627	C	G	0.00897	0.99103	0.00897	59.95	216.40	1.0000	10.54	25.39	0.0061	C
12	676390	chr12:546651	546651	A	C	0.27300	0.72700	0.27300	60.00	195.10	0.0458	13.87	46.50	0.0518	C

12	676409	chr12:546670	546670	G	A	0.01471	0.98529	0.01471	59.92	226.32	1.0000	20.30	58.95	0.0027	G
12	676425	chr12:546686	546686	T	C	0.17397	0.82603	0.17397	59.82	153.60	0.0000	10.00	32.33	0.0745	C
12	676440	chr12:546701	546701	G	T	0.00045	0.99955	0.00045	60.00	47.00	1.0000	10.00	10.00	0.0265	G
12	676445	chr12:546706	546706	T	C	0.00045	0.99955	0.00045	60.00	46.00	1.0000	10.00	10.00	0.0247	T
12	676482	chr12:546743	546743	T	A	0.00075	0.99925	0.00075	60.00	64.67	1.0000	22.30	28.00	0.0040	t
12	676484	chr12:546745	546745	C	A	0.19753	0.80247	0.19753	59.99	182.60	0.0038	11.00	40.20	0.0000	A
12	676515	chr12:546776	546776	G	A	0.00039	0.99961	0.00039	60.00	228.00	1.0000	54.00	54.00	0.0000	g
12	676518	chr12:546779	546779	C	T	0.00025	0.99975	0.00025	60.00	228.00	1.0000	41.00	41.00	0.0000	C
12	676543	chr12:546804	546804	C	T	0.26171	0.73829	0.26171	60.00	193.22	0.0005	15.24	51.05	0.0168	T
12	676560	chr12:546821	546821	T	A	0.01125	0.98875	0.01125	59.98	125.27	1.0000	12.53	28.78	0.0166	T
12	676614	chr12:546875	546875	T	C	0.00044	0.99956	0.00044	60.00	228.00	1.0000	93.00	93.00	0.0000	T
12	676641	chr12:546902	546902	A	G	0.00046	0.99954	0.00046	60.00	81.63	1.0000	41.42	43.15	0.0018	a
12	676651	chr12:546912	546912	G	C	0.00032	0.99968	0.00032	60.00	228.00	1.0000	57.50	57.50	0.0000	G
12	676652	chr12:546913	546913	G	C	0.00025	0.99975	0.00025	60.00	228.00	1.0000	71.00	71.00	0.0000	G
12	676658	chr12:546919	546919	T	G	0.00039	0.99961	0.00039	60.00	228.00	1.0000	41.00	41.00	0.0000	T
12	676688	chr12:546949	546949	G	A	0.00147	0.99853	0.00147	60.00	228.00	1.0000	44.71	82.21	0.0000	G
12	676743	chr12:547004	547004	G	A	0.00041	0.99959	0.00041	60.00	228.00	1.0000	61.02	61.02	0.0000	G
12	676744	chr12:547005	547005	T	C	0.00025	0.99975	0.00025	60.00	228.00	1.0000	76.00	76.00	0.0000	T
12	676749	chr12:547010	547010	C	T	0.00044	0.99956	0.00044	60.00	228.00	1.0000	72.00	72.00	0.0000	C
12	676750	chr12:547011	547011	G	A	0.00039	0.99961	0.00039	60.00	228.00	1.0000	91.00	91.00	0.0000	G
12	676762	chr12:547023	547023	C	G	0.00113	0.99887	0.00113	60.00	227.74	1.0000	45.02	82.61	0.0000	C
12	676781	chr12:547042	547042	G	A	0.00025	0.99975	0.00025	60.00	228.00	1.0000	91.00	91.00	0.0000	G
12	676823	chr12:547084	547084	G	A	0.00045	0.99955	0.00045	60.00	228.00	1.0000	78.55	83.89	0.0000	G
12	676867	chr12:547128	547128	G	A	0.00044	0.99956	0.00044	60.00	228.00	1.0000	92.00	92.00	0.0000	G
12	676883	chr12:547144	547144	G	A	0.12090	0.87910	0.12090	59.99	218.19	0.2423	23.77	57.55	0.0161	G
12	677187	chr12:547448	547448	A	C	0.00025	0.99975	0.00025	60.00	228.00	1.0000	28.00	28.00	0.0045	A
12	677203	chr12:547464	547464	T	C	0.05841	0.94159	0.05841	59.98	225.44	0.1765	22.23	59.76	0.0070	T
12	677228	chr12:547489	547489	T	C	0.00044	0.99956	0.00044	60.00	228.00	1.0000	61.00	61.00	0.0000	T
12	677232	chr12:547493	547493	G	T	0.00025	0.99975	0.00025	60.00	228.00	1.0000	65.00	65.00	0.0000	G
12	677277	chr12:547538	547538	A	G	0.00039	0.99961	0.00039	60.00	228.00	1.0000	46.00	46.00	0.0000	A
12	677327	chr12:547588	547588	A	G	0.00033	0.99967	0.00033	60.00	228.00	1.0000	37.75	37.75	0.0455	A
12	677413	chr12:547674	547674	A	G	0.00039	0.99961	0.00039	60.00	228.00	1.0000	51.00	51.00	0.0000	A
12	677472	chr12:547733	547733	A	C	0.00077	0.99923	0.00077	60.00	86.00	1.0000	38.00	38.00	0.0000	A
12	677481	chr12:547742	547742	C	T	0.00025	0.99975	0.00025	60.00	223.00	1.0000	27.00	27.00	0.0025	C

12	677521	chr12:547782	547782	C	G	0.00039	0.99961	0.00039	60.00	228.00	1.0000	69.00	69.00	0.0000	C
12	677562	chr12:547823	547823	G	C	0.00039	0.99961	0.00039	60.00	228.00	1.0000	55.00	55.00	0.0000	G
12	677601	chr12:547862	547862	G	A	0.38063	0.61937	0.38063	60.00	205.23	0.0178	16.31	62.22	0.0324	G
12	677625	chr12:547886	547886	G	T	0.00039	0.99961	0.00039	60.00	37.00	1.0000	10.00	10.00	0.0008	G
12	677640	chr12:547901	547901	G	A	0.00059	0.99941	0.00059	59.75	228.00	1.0000	20.50	22.75	0.0404	G
12	677683	chr12:547944	547944	C	T	0.00041	0.99959	0.00041	60.00	60.00	1.0000	10.00	10.00	0.0610	C
12	677731	chr12:547992	547992	C	T	0.00051	0.99949	0.00051	60.00	56.00	1.0000	10.00	10.00	0.1352	C
12	677756	chr12:548017	548017	T	C	0.42112	0.57888	0.42112	60.00	195.67	0.0096	15.44	55.83	0.0556	C
12	677862	chr12:548123	548123	A	G	0.00045	0.99955	0.00045	60.00	211.36	1.0000	33.70	39.25	0.0043	A
12	677866	chr12:548127	548127	C	T	0.00039	0.99961	0.00039	60.00	228.00	1.0000	77.00	77.00	0.0000	C
12	677868	chr12:548129	548129	A	C	0.00044	0.99956	0.00044	60.00	228.00	1.0000	42.00	42.00	0.0009	A
12	677887	chr12:548148	548148	C	A	0.00100	0.99900	0.00100	60.00	228.00	1.0000	21.60	56.78	0.0045	C
12	677921	chr12:548182	548182	G	A	0.00100	0.99900	0.00100	60.00	228.00	1.0000	23.15	55.00	0.0060	G
12	677977	chr12:548238	548238	C	A	0.00026	0.99974	0.00026	60.00	170.00	1.0000	33.00	33.00	0.0280	C
12	678015	chr12:548276	548276	C	T	0.00053	0.99947	0.00053	60.00	228.00	1.0000	30.00	30.00	0.1643	C
12	678179	chr12:548440	548440	C	T	0.00039	0.99961	0.00039	60.00	228.00	1.0000	18.00	18.00	0.0069	C
12	678180	chr12:548441	548441	G	A	0.00048	0.99952	0.00048	60.00	87.00	1.0000	10.00	10.00	0.0839	G
12	678188	chr12:548449	548449	T	G	0.00028	0.99972	0.00028	60.00	216.00	1.0000	26.00	26.00	0.1058	T
12	678190	chr12:548451	548451	G	T	0.00064	0.99936	0.00064	60.00	225.47	1.0000	25.39	28.72	0.0578	G
12	678210	chr12:548471	548471	A	G	0.82600	0.17400	0.17400	60.00	165.22	0.1529	20.00	54.70	0.0000	G
12	678215	chr12:548476	548476	C	T	0.00039	0.99961	0.00039	60.00	228.00	1.0000	51.00	51.00	0.0000	C
12	678272	chr12:548533	548533	G	A	0.00025	0.99975	0.00025	60.00	228.00	1.0000	59.00	59.00	0.0165	G
12	678300	chr12:548561	548561	A	G	0.08002	0.91998	0.08002	60.00	222.12	0.4984	18.41	70.29	0.0128	G
12	678337	chr12:548598	548598	G	A	0.00026	0.99974	0.00026	60.00	228.00	1.0000	35.00	35.00	0.0384	G
12	678397	chr12:548658	548658	A	C	0.00530	0.99470	0.00530	60.00	115.67	1.0000	10.42	25.88	0.0601	C
12	678419	chr12:548680	548680	C	T	0.00028	0.99972	0.00028	60.00	228.00	1.0000	28.00	28.00	0.0999	C
12	678425	chr12:548686	548686	G	A	0.00060	0.99940	0.00060	60.00	207.26	1.0000	25.70	27.76	0.0581	G
12	678452	chr12:548713	548713	G	A	0.00041	0.99959	0.00041	60.00	204.36	1.0000	17.83	17.83	0.0057	G
12	678477	chr12:548738	548738	G	A	0.00044	0.99956	0.00044	60.00	228.00	1.0000	51.00	51.00	0.0044	G
12	678499	chr12:548760	548760	A	G	0.00114	0.99886	0.00114	60.00	210.96	1.0000	19.95	44.38	0.0111	A
12	678510	chr12:548771	548771	C	T	0.00077	0.99923	0.00077	60.00	228.00	1.0000	43.48	61.53	0.0000	C
12	678511	chr12:548772	548772	G	A	0.00051	0.99949	0.00051	60.00	228.00	1.0000	37.00	37.00	0.0210	G
12	678564	chr12:548825	548825	C	A	0.00044	0.99956	0.00044	60.00	228.00	1.0000	39.00	39.00	0.0062	C
12	678601	chr12:548862	548862	C	G	0.00046	0.99954	0.00046	60.00	228.00	1.0000	39.00	39.00	0.0309	C

12	678658	chr12:548919	548919	G	C	0.48333	0.51667	0.48333	60.00	192.53	0.0802	15.83	60.53	0.0561	C
12	678659	chr12:548920	548920	C	T	0.00026	0.99974	0.00026	60.00	123.00	1.0000	20.00	20.00	0.0439	C
12	678735	chr12:548996	548996	G	C	0.00077	0.99923	0.00077	60.00	228.00	1.0000	37.35	50.65	0.0000	G
12	678740	chr12:549001	549001	G	A	0.00039	0.99961	0.00039	60.00	228.00	1.0000	43.00	43.00	0.0000	G
12	678780	chr12:549041	549041	C	T	0.00029	0.99971	0.00029	60.00	228.00	1.0000	23.00	23.00	0.1473	T
12	678801	chr12:549062	549062	A	G	0.00039	0.99961	0.00039	60.00	53.00	1.0000	13.00	13.00	0.0023	A
12	678843	chr12:549104	549104	A	T	0.09054	0.90946	0.09054	60.00	214.89	0.0004	15.33	51.93	0.0700	T
12	678867	chr12:549128	549128	A	G	0.00044	0.99956	0.00044	60.00	228.00	1.0000	57.00	57.00	0.0053	A
12	678870	chr12:549131	549131	T	G	0.00044	0.99956	0.00044	60.00	228.00	1.0000	61.00	61.00	0.0053	T
12	678970	chr12:549231	549231	G	C	0.00039	0.99961	0.00039	60.00	228.00	1.0000	38.00	38.00	0.0000	G
12	679257	chr12:549518	549518	C	T	0.00041	0.99959	0.00041	60.00	228.00	1.0000	23.00	23.00	0.0617	C
12	679451	chr12:549712	549712	A	G	0.00032	0.99968	0.00032	60.00	114.71	1.0000	27.06	27.06	0.0598	A
12	679479	chr12:549740	549740	C	T	0.00039	0.99961	0.00039	60.00	228.00	1.0000	46.00	46.00	0.0000	T
12	679516	chr12:549777	549777	A	G	0.00030	0.99970	0.00030	60.00	228.00	1.0000	18.00	18.00	0.1663	A
12	679550	chr12:549811	549811	C	T	0.03524	0.96476	0.03524	60.00	157.93	0.0665	12.84	48.95	0.0395	C
12	679978	chr12:550239	550239	C	T	0.01305	0.98695	0.01305	59.96	178.21	1.0000	10.00	19.00	0.1721	C
12	680009	chr12:550270	550270	C	A	0.00054	0.99946	0.00054	60.00	31.00	1.0000	10.00	10.00	0.1864	C
12	680013	chr12:550274	550274	T	C	0.00102	0.99898	0.00102	60.00	50.00	1.0000	11.05	12.95	0.1325	T
12	680036	chr12:550297	550297	G	A	0.00380	0.99620	0.00380	60.00	225.49	1.0000	22.51	49.00	0.0462	G
12	680061	chr12:550322	550322	A	G	0.00077	0.99923	0.00077	60.00	228.00	1.0000	55.03	55.98	0.0000	A
12	680117	chr12:550378	550378	T	A	0.00028	0.99972	0.00028	60.00	228.00	1.0000	40.00	40.00	0.1013	T
12	680137	chr12:550398	550398	G	A	0.00034	0.99966	0.00034	60.00	228.00	1.0000	28.81	28.81	0.0667	G
12	680173	chr12:550434	550434	A	G	0.00029	0.99971	0.00029	60.00	69.00	1.0000	10.00	10.00	0.1403	A
12	680289	chr12:550550	550550	A	T	0.75901	0.24099	0.24099	60.00	152.65	0.5741	12.67	52.57	0.0386	T
12	680337	chr12:550598	550598	A	C	0.00048	0.99952	0.00048	60.00	38.00	1.0000	10.00	10.00	0.0786	A
12	680383	chr12:550644	550644	G	C	0.00046	0.99954	0.00046	60.00	228.00	1.0000	30.00	30.00	0.0398	G
12	680405	chr12:550666	550666	G	A	0.00031	0.99969	0.00031	60.00	36.00	1.0000	10.00	10.00	0.1977	G
12	680431	chr12:550692	550692	G	C	0.00028	0.99972	0.00028	60.00	228.00	1.0000	25.00	25.00	0.1088	G
12	680435	chr12:550696	550696	G	T	0.00145	0.99855	0.00145	60.00	217.83	1.0000	22.13	43.23	0.0069	G
12	680484	chr12:550745	550745	G	A	0.00045	0.99955	0.00045	60.00	228.00	1.0000	39.00	39.00	0.0097	G
12	680529	chr12:550790	550790	G	A	0.00039	0.99961	0.00039	58.00	119.00	1.0000	10.00	10.00	0.0185	g
12	680643	chr12:550904	550904	T	A	0.00049	0.99951	0.00049	60.00	69.00	1.0000	10.00	10.00	0.1016	T
12	680676	chr12:550937	550937	A	G	0.03477	0.96523	0.03477	59.98	211.25	0.0233	14.53	46.31	0.0957	A
12	680692	chr12:550953	550953	C	T	0.00039	0.99961	0.00039	60.00	228.00	1.0000	39.00	39.00	0.0008	C

12	680693	chr12:550954	550954	G	A	0.00026	0.99974	0.00026	60.00	228.00	1.0000	43.00	43.00	0.0509	G
12	680709	chr12:550970	550970	T	A	0.00044	0.99956	0.00044	60.00	48.00	1.0000	11.00	11.00	0.0027	T
12	680755	chr12:551016	551016	G	A	0.00039	0.99961	0.00039	60.00	228.00	1.0000	64.00	64.00	0.0000	G
12	680786	chr12:551047	551047	A	T	0.00025	0.99975	0.00025	60.00	228.00	1.0000	19.00	19.00	0.0065	A
12	680794	chr12:551055	551055	G	A	0.00025	0.99975	0.00025	60.00	228.00	1.0000	32.00	32.00	0.0065	G
12	680795	chr12:551056	551056	A	T	0.00039	0.99961	0.00039	60.00	228.00	1.0000	67.00	67.00	0.0000	A
12	680823	chr12:551084	551084	T	C	0.00044	0.99956	0.00044	60.00	228.00	1.0000	46.00	46.00	0.0009	T
12	680835	chr12:551096	551096	T	C	0.00044	0.99956	0.00044	60.00	228.00	1.0000	50.00	50.00	0.0009	T
12	680845	chr12:551106	551106	C	T	0.00039	0.99961	0.00039	60.00	228.00	1.0000	83.00	83.00	0.0000	C
12	680851	chr12:551112	551112	T	C	0.00039	0.99961	0.00039	60.00	228.00	1.0000	73.00	73.00	0.0000	T
12	680934	chr12:551195	551195	T	C	0.00039	0.99961	0.00039	60.00	228.00	1.0000	27.00	27.00	0.0000	T
12	681004	chr12:551265	551265	A	G	0.00044	0.99956	0.00044	60.00	228.00	1.0000	37.00	37.00	0.0044	A
12	681044	chr12:551305	551305	C	T	0.00025	0.99975	0.00025	60.00	228.00	1.0000	48.00	48.00	0.0125	C
12	681059	chr12:551320	551320	A	G	0.00025	0.99975	0.00025	60.00	228.00	1.0000	22.00	22.00	0.0075	A
12	681078	chr12:551339	551339	A	C	0.00039	0.99961	0.00039	60.00	228.00	1.0000	57.00	57.00	0.0000	A
12	681086	chr12:551347	551347	C	G	0.00025	0.99975	0.00025	60.00	228.00	1.0000	22.00	22.00	0.0115	C
12	681087	chr12:551348	551348	G	A	0.00039	0.99961	0.00039	60.00	228.00	1.0000	59.00	59.00	0.0000	G
12	681119	chr12:551380	551380	G	A	0.00039	0.99961	0.00039	60.00	228.00	1.0000	60.00	60.00	0.0000	G
12	681141	chr12:551402	551402	G	A	0.00044	0.99956	0.00044	60.00	228.00	1.0000	40.00	40.00	0.0035	G
12	681147	chr12:551408	551408	A	G	0.00025	0.99975	0.00025	60.00	197.00	1.0000	30.00	30.00	0.0170	G
12	681160	chr12:551421	551421	G	T	0.00044	0.99956	0.00044	60.00	167.00	1.0000	19.00	19.00	0.0035	g
12	681223	chr12:551484	551484	T	C	0.00076	0.99924	0.00076	60.00	228.00	1.0000	15.90	52.95	0.0210	T
12	681226	chr12:551487	551487	T	C	0.00062	0.99938	0.00062	60.00	228.00	1.0000	54.29	69.01	0.0012	T
12	681233	chr12:551494	551494	G	A	0.00039	0.99961	0.00039	60.00	228.00	1.0000	55.00	55.00	0.0000	G
12	681239	chr12:551500	551500	C	T	0.00062	0.99938	0.00062	60.00	228.00	1.0000	56.76	56.76	0.0016	C
12	681245	chr12:551506	551506	G	A	0.00039	0.99961	0.00039	60.00	228.00	1.0000	62.00	62.00	0.0000	G
12	681257	chr12:551518	551518	C	T	0.00800	0.99200	0.00800	60.00	220.17	1.0000	20.29	67.03	0.0123	C
12	681283	chr12:551544	551544	G	A	0.00026	0.99974	0.00026	60.00	228.00	1.0000	43.00	43.00	0.0240	G
12	681287	chr12:551548	551548	G	A	0.00039	0.99961	0.00039	60.00	228.00	1.0000	24.00	24.00	0.0000	G
12	681289	chr12:551550	551550	A	G	0.00026	0.99974	0.00026	60.00	107.00	1.0000	13.00	13.00	0.0240	A
12	681324	chr12:551585	551585	C	T	0.00026	0.99974	0.00026	60.00	228.00	1.0000	24.00	24.00	0.0325	C
12	681347	chr12:551608	551608	G	T	0.00026	0.99974	0.00026	60.00	201.00	1.0000	26.00	26.00	0.0494	G
12	681350	chr12:551611	551611	G	A	0.00511	0.99489	0.00511	60.00	227.07	1.0000	18.67	48.43	0.0282	G
12	681372	chr12:551633	551633	G	A	0.00035	0.99965	0.00035	60.00	228.00	1.0000	23.88	23.88	0.0822	G

12	681398	chr12:551659	551659	C	T	0.00030	0.99970	0.00030	60.00	80.00	1.0000	11.00	11.00	0.1772	C
12	681485	chr12:551746	551746	T	A	0.00043	0.99957	0.00043	60.00	185.00	1.0000	13.00	13.00	0.1026	T
12	681599	chr12:551860	551860	G	A	0.00025	0.99975	0.00025	60.00	117.00	1.0000	11.00	11.00	0.0100	G
12	681639	chr12:551900	551900	G	T	0.08793	0.91207	0.08793	60.00	223.38	0.8286	24.66	67.23	0.0105	G
12	681665	chr12:551926	551926	A	G	0.00025	0.99975	0.00025	60.00	228.00	1.0000	77.00	77.00	0.0005	A
12	681686	chr12:551947	551947	T	G	0.00025	0.99975	0.00025	60.00	228.00	1.0000	38.00	38.00	0.0030	T
12	681730	chr12:551991	551991	C	G	0.45886	0.54114	0.45886	60.00	208.45	0.1327	23.83	70.65	0.0070	G
12	681743	chr12:552004	552004	C	T	0.00039	0.99961	0.00039	60.00	228.00	1.0000	35.00	35.00	0.0000	C
12	681755	chr12:552016	552016	A	C	0.31397	0.68603	0.31397	59.99	147.04	0.0002	10.00	30.26	0.0918	C
12	681773	chr12:552034	552034	G	C	0.00131	0.99869	0.00131	60.00	122.00	0.0016	10.35	33.15	0.0439	G
12	681777	chr12:552038	552038	G	A	0.00055	0.99945	0.00055	60.00	82.00	1.0000	13.10	16.90	0.0969	G
12	681783	chr12:552044	552044	A	G	0.00039	0.99961	0.00039	60.00	228.00	1.0000	20.00	20.00	0.0015	A
12	681795	chr12:552056	552056	C	T	0.00050	0.99950	0.00050	60.00	202.00	1.0000	11.13	15.88	0.0030	C
12	681796	chr12:552057	552057	G	C	0.00032	0.99968	0.00032	60.00	228.00	1.0000	27.52	27.52	0.0054	G
12	681819	chr12:552080	552080	G	A	0.00025	0.99975	0.00025	60.00	228.00	1.0000	49.00	49.00	0.0010	G
12	681832	chr12:552093	552093	G	C	0.41033	0.58967	0.41033	60.00	187.05	0.0027	13.86	69.50	0.0981	C
12	681833	chr12:552094	552094	C	G	0.00966	0.99034	0.00966	59.99	102.35	1.0000	12.57	33.74	0.0093	C
12	681892	chr12:552153	552153	C	T	0.00039	0.99961	0.00039	60.00	228.00	1.0000	52.00	52.00	0.0000	C
12	681897	chr12:552158	552158	T	G	0.00039	0.99961	0.00039	60.00	228.00	1.0000	36.00	36.00	0.0000	T
12	681903	chr12:552164	552164	A	T	0.00044	0.99956	0.00044	60.00	228.00	1.0000	37.00	37.00	0.0027	A
12	681906	chr12:552167	552167	G	A	0.00032	0.99968	0.00032	60.00	223.67	1.0000	40.20	40.20	0.0019	G
12	681944	chr12:552205	552205	G	A	0.00041	0.99959	0.00041	59.46	228.00	1.0000	37.15	37.15	0.0057	G
12	682600	chr12:552861	552861	G	C	0.00039	0.99961	0.00039	60.00	228.00	1.0000	37.00	37.00	0.0000	G
12	682834	chr12:553095	553095	G	C	0.00025	0.99975	0.00025	60.00	228.00	1.0000	48.00	48.00	0.0000	G
12	682926	chr12:553187	553187	G	A	0.00056	0.99944	0.00056	60.00	58.71	1.0000	17.68	18.65	0.0009	G
12	682933	chr12:553194	553194	C	T	0.00169	0.99831	0.00169	60.00	223.05	1.0000	23.90	58.15	0.0007	C
12	682946	chr12:553207	553207	C	A	0.00079	0.99921	0.00079	60.00	227.77	1.0000	27.35	41.00	0.0011	C
12	682961	chr12:553222	553222	A	T	0.00025	0.99975	0.00025	60.00	30.00	1.0000	10.00	10.00	0.0030	A
12	682964	chr12:553225	553225	G	A	0.00032	0.99968	0.00032	60.00	228.00	1.0000	53.67	53.67	0.0000	G
12	683066	chr12:553327	553327	G	C	0.00039	0.99961	0.00039	60.00	228.00	1.0000	92.00	92.00	0.0000	G
12	683070	chr12:553331	553331	C	G	0.00025	0.99975	0.00025	60.00	228.00	1.0000	68.00	68.00	0.0000	C
12	683580	chr12:553841	553841	G	A	0.00039	0.99961	0.00039	59.00	228.00	1.0000	32.00	32.00	0.0000	G
12	683606	chr12:553867	553867	T	C	0.43695	0.56305	0.43695	57.94	197.94	0.4208	21.54	65.94	0.0243	T
12	683637	chr12:553898	553898	A	G	0.00025	0.99975	0.00025	58.00	228.00	1.0000	33.00	33.00	0.0030	A

12	683662	chr12:553923	553923	A	G	0.00083	0.99917	0.00083	60.00	62.06	1.0000	14.36	18.96	0.0126	A
12	683677	chr12:553938	553938	A	G	0.00044	0.99956	0.00044	60.00	61.00	1.0000	11.00	11.00	0.0009	A
12	683719	chr12:553980	553980	A	G	0.00025	0.99975	0.00025	59.00	60.00	1.0000	10.00	10.00	0.0190	A
12	683739	chr12:554000	554000	C	T	0.00570	0.99430	0.00570	59.96	192.67	1.0000	11.41	36.64	0.0101	C
12	683755	chr12:554016	554016	C	T	0.00025	0.99975	0.00025	60.00	152.00	1.0000	22.00	22.00	0.0105	C
12	683759	chr12:554020	554020	C	T	0.00025	0.99975	0.00025	60.00	228.00	1.0000	34.00	34.00	0.0110	C
12	683798	chr12:554059	554059	C	T	0.00039	0.99961	0.00039	60.00	228.00	1.0000	34.00	34.00	0.0000	C
12	683898	chr12:554159	554159	T	C	0.00045	0.99955	0.00045	58.00	86.00	1.0000	15.00	15.00	0.0177	T
12	683915	chr12:554176	554176	G	A	0.00025	0.99975	0.00025	60.00	228.00	1.0000	27.00	27.00	0.0085	G
12	683936	chr12:554197	554197	C	T	0.00080	0.99920	0.00080	59.84	228.00	1.0000	27.28	38.88	0.0032	C
12	683946	chr12:554207	554207	G	A	0.45565	0.54435	0.45565	59.90	199.69	0.2068	19.16	58.15	0.0303	A
12	684007	chr12:554268	554268	C	T	0.00064	0.99936	0.00064	60.00	228.00	1.0000	40.73	50.41	0.0000	C
12	684113	chr12:554374	554374	A	G	0.00025	0.99975	0.00025	60.00	228.00	1.0000	67.00	67.00	0.0000	A
12	684166	chr12:554427	554427	G	A	0.00025	0.99975	0.00025	60.00	228.00	1.0000	65.00	65.00	0.0005	G
12	684218	chr12:554479	554479	T	A	0.00045	0.99955	0.00045	60.00	79.00	1.0000	11.00	11.00	0.0194	T
12	684254	chr12:554515	554515	C	G	0.00039	0.99961	0.00039	60.00	228.00	1.0000	38.00	38.00	0.0000	C
12	684276	chr12:554537	554537	C	T	0.00044	0.99956	0.00044	60.00	195.00	1.0000	32.00	32.00	0.0035	C
12	684311	chr12:554572	554572	C	T	0.00046	0.99954	0.00046	60.00	173.71	1.0000	20.80	21.37	0.0106	C
12	684324	chr12:554585	554585	C	T	0.00051	0.99949	0.00051	60.00	228.00	1.0000	35.30	46.70	0.0235	C
12	684340	chr12:554601	554601	C	T	0.00033	0.99967	0.00033	60.00	228.00	1.0000	28.28	28.28	0.0204	C
12	684346	chr12:554607	554607	C	T	0.00039	0.99961	0.00039	60.00	228.00	1.0000	30.00	30.00	0.0015	C
12	684350	chr12:554611	554611	C	T	0.00026	0.99974	0.00026	60.00	210.00	1.0000	31.00	31.00	0.0290	C
12	684352	chr12:554613	554613	G	A	0.00077	0.99923	0.00077	60.00	228.00	1.0000	25.18	31.83	0.0015	G
12	684353	chr12:554614	554614	C	T	0.00045	0.99955	0.00045	60.00	80.00	1.0000	18.00	18.00	0.0080	C
12	684391	chr12:554652	554652	T	C	0.00025	0.99975	0.00025	60.00	46.00	1.0000	17.00	17.00	0.0205	T
12	684394	chr12:554655	554655	G	A	0.00025	0.99975	0.00025	60.00	94.00	1.0000	20.00	20.00	0.0180	G
12	684495	chr12:554756	554756	G	A	0.00063	0.99937	0.00063	60.00	228.00	1.0000	27.17	40.69	0.0167	G
12	684508	chr12:554769	554769	T	G	0.00039	0.99961	0.00039	60.00	206.00	1.0000	23.00	23.00	0.0062	-
12	684526	chr12:554787	554787	G	A	0.00089	0.99911	0.00089	60.00	171.50	1.0000	12.93	48.08	0.0080	G
12	684534	chr12:554795	554795	G	A	0.00033	0.99967	0.00033	60.00	173.09	1.0000	24.78	24.78	0.0187	G
12	684580	chr12:554841	554841	C	G	0.00032	0.99968	0.00032	60.00	228.00	1.0000	32.27	32.27	0.0121	C
12	684618	chr12:554879	554879	G	A	0.00025	0.99975	0.00025	60.00	228.00	1.0000	16.00	16.00	0.0165	G
12	684620	chr12:554881	554881	C	T	0.00159	0.99841	0.00159	60.00	228.00	1.0000	24.92	47.88	0.0087	C
12	684644	chr12:554905	554905	C	T	0.00039	0.99961	0.00039	60.00	228.00	1.0000	42.00	42.00	0.0000	C

12	684651	chr12:554912	554912	G	A	0.66792	0.33208	0.33208	60.00	182.11	0.0723	16.33	66.03	0.0401 A
12	684659	chr12:554920	554920	C	T	0.00025	0.99975	0.00025	60.00	218.00	1.0000	32.00	32.00	0.0180 C
12	684675	chr12:554936	554936	T	G	0.00025	0.99975	0.00025	60.00	228.00	1.0000	63.00	63.00	0.0190 T
12	684701	chr12:554962	554962	C	G	0.00052	0.99948	0.00052	60.00	228.00	1.0000	36.20	43.80	0.0330 C
12	684718	chr12:554979	554979	C	T	0.00027	0.99973	0.00027	60.00	208.00	1.0000	12.00	12.00	0.0644 C
12	684732	chr12:554993	554993	C	T	0.00027	0.99973	0.00027	60.00	154.00	1.0000	11.00	11.00	0.0804 C
12	684738	chr12:554999	554999	C	T	0.00054	0.99946	0.00054	60.00	191.00	1.0000	11.48	29.53	0.0729 C
12	684826	chr12:555087	555087	C	T	0.00039	0.99961	0.00039	60.00	228.00	1.0000	27.00	27.00	0.0000 C
12	684855	chr12:555116	555116	C	T	0.00025	0.99975	0.00025	60.00	228.00	1.0000	47.00	47.00	0.0155 C
12	684859	chr12:555120	555120	T	C	0.00044	0.99956	0.00044	60.00	67.00	1.0000	45.00	45.00	0.0044 T
12	684930	chr12:555191	555191	G	A	0.00031	0.99969	0.00031	60.00	228.00	1.0000	37.35	37.35	0.0105 G
12	684943	chr12:555204	555204	G	T	0.00039	0.99961	0.00039	60.00	228.00	1.0000	60.00	60.00	0.0000 G
12	684971	chr12:555232	555232	G	T	0.00026	0.99974	0.00026	60.00	228.00	1.0000	44.00	44.00	0.0235 G
12	684986	chr12:555247	555247	G	A	0.00080	0.99920	0.00080	60.00	228.00	1.0000	29.08	43.69	0.0185 G
12	685007	chr12:555268	555268	A	T	0.41445	0.58555	0.41445	60.00	185.22	0.1424	13.75	46.32	0.1123 A
12	685037	chr12:555298	555298	T	C	0.00026	0.99974	0.00026	60.00	228.00	1.0000	26.00	26.00	0.0349 T
12	685056	chr12:555317	555317	T	C	0.00026	0.99974	0.00026	60.00	63.00	1.0000	30.00	30.00	0.0290 G
12	685142	chr12:555403	555403	C	T	0.00026	0.99974	0.00026	60.00	171.00	1.0000	19.00	19.00	0.0524 C
12	685169	chr12:555430	555430	C	T	0.00027	0.99973	0.00027	60.00	228.00	1.0000	19.00	19.00	0.0819 C
12	685170	chr12:555431	555431	A	G	0.00048	0.99952	0.00048	60.00	185.54	1.0000	16.85	17.97	0.0500 A
12	685188	chr12:555449	555449	T	C	0.00082	0.99918	0.00082	60.00	191.47	0.0016	18.25	21.79	0.0341 T
12	685198	chr12:555459	555459	C	T	0.00026	0.99974	0.00026	60.00	44.00	1.0000	20.00	20.00	0.0384 C
12	685207	chr12:555468	555468	T	A	0.00093	0.99907	0.00093	60.00	81.15	1.0000	19.17	27.63	0.0288 t
12	685280	chr12:555541	555541	A	T	0.00025	0.99975	0.00025	60.00	228.00	1.0000	23.00	23.00	0.0105 A
12	685288	chr12:555549	555549	T	A	0.00039	0.99961	0.00039	60.00	228.00	1.0000	59.00	59.00	0.0000 T
12	685557	chr12:555818	555818	A	T	0.00025	0.99975	0.00025	60.00	208.00	1.0000	28.00	28.00	0.0155 A
12	685563	chr12:555824	555824	G	A	0.00045	0.99955	0.00045	60.00	228.00	1.0000	35.77	37.71	0.0054 G
12	685606	chr12:555867	555867	G	A	0.00107	0.99893	0.00107	60.00	80.79	1.0000	20.21	24.90	0.0369 G
12	685632	chr12:555893	555893	T	C	0.00680	0.99320	0.00680	60.00	219.48	1.0000	21.74	56.84	0.0049 T
12	685641	chr12:555902	555902	C	T	0.00076	0.99924	0.00076	60.00	228.00	1.0000	36.24	51.92	0.0033 C
12	685878	chr12:556139	556139	C	T	0.44044	0.55956	0.44044	60.00	208.62	0.1624	24.01	68.24	0.0203 T
12	685905	chr12:556166	556166	C	G	0.00044	0.99956	0.00044	60.00	154.00	1.0000	11.00	11.00	0.0000 C
12	685910	chr12:556171	556171	C	T	0.00044	0.99956	0.00044	60.00	228.00	1.0000	46.00	46.00	0.0000 C
12	685932	chr12:556193	556193	A	C	0.00025	0.99975	0.00025	60.00	228.00	1.0000	37.00	37.00	0.0020 A

12	685973	chr12:556234	556234	C	G	0.00025	0.99975	0.00025	60.00	228.00	1.0000	48.00	48.00	0.0010	C
12	686010	chr12:556271	556271	A	C	0.00064	0.99936	0.00064	60.00	228.00	1.0000	38.64	43.74	0.0022	A
12	686024	chr12:556285	556285	C	G	0.00077	0.99923	0.00077	60.00	228.00	1.0000	48.00	48.00	0.0000	C
12	686076	chr12:556337	556337	G	T	0.82442	0.17558	0.17558	60.00	185.77	0.0018	24.24	72.84	0.0038	T
12	686081	chr12:556342	556342	G	A	0.00044	0.99956	0.00044	60.00	169.00	1.0000	21.00	21.00	0.0027	G
12	686103	chr12:556364	556364	C	T	0.00044	0.99956	0.00044	60.00	133.00	1.0000	17.00	17.00	0.0035	C
12	686120	chr12:556381	556381	G	A	0.00039	0.99961	0.00039	60.00	228.00	1.0000	40.00	40.00	0.0000	G
12	686170	chr12:556431	556431	T	C	0.77456	0.22544	0.22544	60.00	166.93	0.0770	15.13	58.08	0.0188	C
12	686254	chr12:556515	556515	C	A	0.00039	0.99961	0.00039	60.00	228.00	1.0000	51.00	51.00	0.0000	C
12	686286	chr12:556547	556547	T	C	0.00044	0.99956	0.00044	60.00	228.00	1.0000	55.00	55.00	0.0009	T
12	686448	chr12:556709	556709	G	C	0.20436	0.79564	0.20436	60.00	216.94	0.0149	26.05	72.09	0.0219	G
12	686705	chr12:556966	556966	C	G	0.00102	0.99898	0.00102	59.89	228.00	1.0000	29.94	38.26	0.0018	C
12	686723	chr12:556984	556984	G	T	0.00192	0.99808	0.00192	60.00	201.86	1.0000	24.69	46.88	0.0029	G
12	686835	chr12:557096	557096	T	C	0.00044	0.99956	0.00044	60.00	228.00	1.0000	40.00	40.00	0.0000	T
12	687587	chr12:557848	557848	G	A	0.00039	0.99961	0.00039	52.00	123.00	1.0000	10.00	10.00	0.0000	G
12	687603	chr12:557864	557864	G	C	0.00025	0.99975	0.00025	58.00	168.00	1.0000	11.00	11.00	0.0085	G
12	687657	chr12:557918	557918	C	T	0.00025	0.99975	0.00025	60.00	139.00	1.0000	29.00	29.00	0.0010	C
12	687673	chr12:557934	557934	G	A	0.28883	0.71117	0.28883	59.46	196.29	0.0487	13.96	38.12	0.0473	g
12	687683	chr12:557944	557944	T	G	0.00025	0.99975	0.00025	59.00	105.00	1.0000	15.00	15.00	0.0015	T
12	687693	chr12:557954	557954	A	G	0.04033	0.95967	0.04033	56.12	133.82	0.2355	10.00	16.47	0.0142	A
12	687718	chr12:557979	557979	A	T	0.00025	0.99975	0.00025	58.00	59.00	1.0000	19.00	19.00	0.0015	A
12	687769	chr12:558030	558030	C	G	0.00045	0.99955	0.00045	59.00	228.00	1.0000	30.00	30.00	0.0088	C
12	687776	chr12:558037	558037	A	T	0.00044	0.99956	0.00044	60.00	71.00	1.0000	15.00	15.00	0.0009	A
12	687780	chr12:558041	558041	G	A	0.00025	0.99975	0.00025	60.00	228.00	1.0000	35.00	35.00	0.0010	G
12	687816	chr12:558077	558077	C	G	0.00064	0.99936	0.00064	59.82	228.00	1.0000	28.40	42.51	0.0013	C
12	687825	chr12:558086	558086	C	T	0.00039	0.99961	0.00039	60.00	228.00	1.0000	51.00	51.00	0.0000	C
12	687828	chr12:558089	558089	G	A	0.00025	0.99975	0.00025	60.00	228.00	1.0000	33.00	33.00	0.0015	G
12	688027	chr12:558288	558288	T	A	0.00025	0.99975	0.00025	60.00	228.00	1.0000	27.00	27.00	0.0005	T
12	688053	chr12:558314	558314	G	C	0.00027	0.99973	0.00027	60.00	177.00	1.0000	37.00	37.00	0.0724	G
12	688065	chr12:558326	558326	T	C	0.00044	0.99956	0.00044	60.00	228.00	1.0000	57.00	57.00	0.0009	T
12	688086	chr12:558347	558347	T	G	0.00044	0.99956	0.00044	60.00	102.00	1.0000	12.00	12.00	0.0009	t
12	688091	chr12:558352	558352	G	T	0.00045	0.99955	0.00045	60.00	228.00	1.0000	40.25	52.27	0.0007	G
12	688160	chr12:558421	558421	C	T	0.00025	0.99975	0.00025	60.00	228.00	1.0000	64.00	64.00	0.0005	C
12	688180	chr12:558441	558441	C	T	0.00025	0.99975	0.00025	60.00	228.00	1.0000	55.00	55.00	0.0005	c

12	688183	chr12:558444	558444	G	A	0.00025	0.99975	0.00025	60.00	228.00	1.0000	62.00	62.00	0.0005	G
12	688240	chr12:558501	558501	A	T	0.00025	0.99975	0.00025	60.00	228.00	1.0000	51.00	51.00	0.0000	A
12	688248	chr12:558509	558509	G	A	0.22250	0.77750	0.22250	60.00	223.23	0.5093	31.51	82.73	0.0029	G
12	688263	chr12:558524	558524	A	C	0.00088	0.99912	0.00088	60.00	228.00	1.0000	14.75	81.25	0.0018	A
12	688275	chr12:558536	558536	G	A	0.00044	0.99956	0.00044	60.00	228.00	1.0000	24.00	24.00	0.0000	G
12	688281	chr12:558542	558542	A	G	0.00056	0.99944	0.00056	60.00	228.00	1.0000	66.95	70.45	0.0000	A
12	688340	chr12:558601	558601	A	C	0.37875	0.62125	0.37875	60.00	207.07	0.4920	20.47	62.93	0.0141	A
12	688364	chr12:558625	558625	G	A	0.00067	0.99933	0.00067	60.00	85.32	1.0000	13.29	26.13	0.0532	G
12	688425	chr12:558686	558686	G	A	0.00039	0.99961	0.00039	60.00	73.00	1.0000	24.00	24.00	0.0000	G
12	688432	chr12:558693	558693	G	A	0.00048	0.99952	0.00048	60.00	44.00	1.0000	10.00	10.00	0.0760	G
12	688444	chr12:558705	558705	A	C	0.00048	0.99952	0.00048	60.00	98.00	1.0000	14.00	14.00	0.0733	A
12	688522	chr12:558783	558783	C	T	0.00154	0.99846	0.00154	60.00	228.00	1.0000	33.68	45.93	0.0000	C
12	688545	chr12:558806	558806	C	T	0.00025	0.99975	0.00025	60.00	228.00	1.0000	48.00	48.00	0.0010	C
12	688564	chr12:558825	558825	G	A	0.06104	0.93896	0.06104	60.00	221.68	0.2989	19.99	61.60	0.0223	G
12	688600	chr12:558861	558861	C	G	0.00025	0.99975	0.00025	60.00	228.00	1.0000	47.00	47.00	0.0005	C
12	688616	chr12:558877	558877	T	C	0.30860	0.69140	0.30860	60.00	207.59	0.2495	18.00	59.00	0.0479	C
12	688631	chr12:558892	558892	G	T	0.00052	0.99948	0.00052	60.00	228.00	1.0000	13.00	13.00	0.1484	G
12	688710	chr12:558971	558971	T	A	0.00025	0.99975	0.00025	59.00	197.00	1.0000	24.00	24.00	0.0005	T
12	688769	chr12:559030	559030	C	T	0.00025	0.99975	0.00025	60.00	228.00	1.0000	63.00	63.00	0.0000	C
12	688771	chr12:559032	559032	G	A	0.00025	0.99975	0.00025	60.00	228.00	1.0000	38.00	38.00	0.0000	G
12	688993	chr12:559254	559254	G	A	0.00076	0.99924	0.00076	59.80	228.00	1.0000	56.52	66.16	0.0000	g
12	689059	chr12:559320	559320	T	C	0.31573	0.68427	0.31573	60.00	225.08	0.1460	36.69	93.04	0.0043	c
12	689302	chr12:559563	559563	G	A	0.00582	0.99418	0.00582	59.40	187.31	1.0000	10.16	19.80	0.0112	G
12	689360	chr12:559621	559621	G	T	0.31553	0.68447	0.31553	60.00	223.28	0.3926	34.23	92.65	0.0047	G
12	689480	chr12:559741	559741	A	G	0.22377	0.77623	0.22377	59.98	214.69	0.4124	23.00	49.22	0.0090	G
12	689809	chr12:560070	560070	G	A	0.00025	0.99975	0.00025	60.00	228.00	1.0000	41.00	41.00	0.0030	G
12	689927	chr12:560188	560188	C	G	0.00025	0.99975	0.00025	60.00	228.00	1.0000	38.00	38.00	0.0000	C
12	689941	chr12:560202	560202	G	A	0.00047	0.99953	0.00047	60.00	228.00	1.0000	16.00	16.00	0.0557	G
12	689959	chr12:560220	560220	T	C	0.00140	0.99860	0.00140	59.80	219.63	1.0000	27.93	46.80	0.0308	T
12	690026	chr12:560287	560287	G	A	0.00026	0.99974	0.00026	60.00	228.00	1.0000	30.00	30.00	0.0235	G
12	690040	chr12:560301	560301	A	C	0.00027	0.99973	0.00027	60.00	228.00	1.0000	23.00	23.00	0.0599	A
12	690368	chr12:560629	560629	C	T	0.00032	0.99968	0.00032	58.65	58.87	1.0000	14.53	14.53	0.0182	c
12	690381	chr12:560642	560642	A	C	0.00025	0.99975	0.00025	59.00	228.00	1.0000	38.00	38.00	0.0015	a
12	690402	chr12:560663	560663	A	G	0.07933	0.92067	0.07933	60.00	216.58	0.0566	14.82	66.80	0.0304	a

12	690409	chr12:560670	560670	G	T	0.00025	0.99975	0.00025	60.00	55.00	1.0000	10.00	10.00	0.0040	g
12	690418	chr12:560679	560679	A	G	0.21895	0.78105	0.21895	60.00	214.52	0.6043	15.99	69.28	0.0162	a
12	690440	chr12:560701	560701	C	A	0.00039	0.99961	0.00039	60.00	228.00	1.0000	40.00	40.00	0.0000	c
12	690452	chr12:560713	560713	C	G	0.20225	0.79775	0.20225	60.00	212.02	0.2059	20.11	79.89	0.0261	c
12	690473	chr12:560734	560734	C	T	0.43299	0.56701	0.43299	60.00	208.79	0.3888	22.61	78.38	0.0325	c
12	690480	chr12:560741	560741	A	G	0.00039	0.99961	0.00039	60.00	228.00	1.0000	50.00	50.00	0.0000	a
12	690484	chr12:560745	560745	C	T	0.00045	0.99955	0.00045	60.00	228.00	1.0000	18.00	18.00	0.0159	c
12	690485	chr12:560746	560746	G	A	0.00025	0.99975	0.00025	60.00	228.00	1.0000	52.00	52.00	0.0025	g
12	690506	chr12:560767	560767	T	C	0.00039	0.99961	0.00039	60.00	228.00	1.0000	32.00	32.00	0.0000	t
12	690515	chr12:560776	560776	C	T	0.00045	0.99955	0.00045	60.00	228.00	1.0000	90.00	90.00	0.0292	c
12	690726	chr12:560987	560987	A	T	0.00046	0.99954	0.00046	60.00	228.00	1.0000	25.00	25.00	0.0353	-
12	690729	chr12:560990	560990	C	T	0.00107	0.99893	0.00107	60.00	228.00	1.0000	32.00	49.92	0.0057	-
12	690820	chr12:561081	561081	A	C	0.10535	0.89465	0.10535	59.99	189.58	0.1677	10.88	32.00	0.0332	-
12	690836	chr12:561097	561097	G	A	0.00030	0.99970	0.00030	60.00	228.00	1.0000	24.00	24.00	0.1692	-
12	690865	chr12:561126	561126	C	T	0.00028	0.99972	0.00028	60.00	228.00	1.0000	31.00	31.00	0.1128	-
12	690879	chr12:561140	561140	C	T	0.00039	0.99961	0.00039	60.00	228.00	1.0000	24.00	24.00	0.0015	-
12	690907	chr12:561168	561168	C	G	0.00025	0.99975	0.00025	60.00	228.00	1.0000	45.00	45.00	0.0035	c
12	690911	chr12:561172	561172	C	T	0.00025	0.99975	0.00025	60.00	228.00	1.0000	35.00	35.00	0.0030	c
12	691000	chr12:561261	561261	A	G	0.00025	0.99975	0.00025	60.00	228.00	1.0000	49.00	49.00	0.0000	a
12	691072	chr12:561333	561333	T	C	0.21980	0.78020	0.21980	60.00	217.89	0.4561	19.81	76.48	0.0081	-
12	691080	chr12:561341	561341	G	A	0.00030	0.99970	0.00030	60.00	228.00	1.0000	66.51	66.51	0.0030	-
12	691103	chr12:561364	561364	C	T	0.00250	0.99750	0.00250	60.00	224.95	1.0000	23.27	53.70	0.0081	-
12	691160	chr12:561421	561421	G	T	0.00026	0.99974	0.00026	60.00	36.00	1.0000	13.00	13.00	0.0265	-
12	691167	chr12:561428	561428	C	G	0.00026	0.99974	0.00026	60.00	228.00	1.0000	29.00	29.00	0.0300	-
12	691177	chr12:561438	561438	G	A	0.02426	0.97574	0.02426	60.00	223.02	0.3817	12.80	51.77	0.0361	-
12	691205	chr12:561466	561466	C	T	0.00039	0.99961	0.00039	60.00	32.00	1.0000	20.00	20.00	0.0008	-
12	691206	chr12:561467	561467	C	T	0.00042	0.99958	0.00042	60.00	45.08	1.0000	18.66	18.66	0.0232	-
12	691208	chr12:561469	561469	T	A	0.00026	0.99974	0.00026	60.00	149.00	1.0000	10.00	10.00	0.0265	-
12	691259	chr12:561520	561520	G	A	0.00025	0.99975	0.00025	60.00	227.00	1.0000	32.00	32.00	0.0115	-
12	691291	chr12:561552	561552	G	C	0.00039	0.99961	0.00039	60.00	228.00	1.0000	44.00	44.00	0.0000	-
12	691320	chr12:561581	561581	C	T	0.21531	0.78469	0.21531	60.00	210.19	0.3813	15.94	63.59	0.0238	-
12	691323	chr12:561584	561584	A	G	0.00025	0.99975	0.00025	60.00	83.00	1.0000	24.00	24.00	0.0175	-
12	691337	chr12:561598	561598	A	C	0.00044	0.99956	0.00044	60.00	228.00	1.0000	20.00	20.00	0.0009	-
12	691339	chr12:561600	561600	T	C	0.20307	0.79693	0.20307	60.00	204.35	0.1159	14.88	59.40	0.0363	-

12	691383	chr12:561644	561644	G	T	0.00026	0.99974	0.00026	60.00	108.00	1.0000	14.00	14.00	0.0270 -
12	691419	chr12:561680	561680	G	A	0.01121	0.98879	0.01121	59.92	193.00	0.0002	14.30	24.43	0.0023 -
12	692440	chr12:562701	562701	C	T	0.00046	0.99954	0.00046	60.00	228.00	1.0000	27.00	27.00	0.0362 c
12	692454	chr12:562715	562715	C	T	0.00044	0.99956	0.00044	60.00	228.00	1.0000	38.00	38.00	0.0009 c
12	692457	chr12:562718	562718	G	A	0.00027	0.99973	0.00027	60.00	140.00	1.0000	16.00	16.00	0.0619 g
12	692487	chr12:562748	562748	G	A	0.00241	0.99759	0.00241	60.00	228.00	1.0000	19.99	51.05	0.0536 g
12	692491	chr12:562752	562752	C	T	0.00039	0.99961	0.00039	60.00	228.00	1.0000	25.00	25.00	0.0008 c
12	692499	chr12:562760	562760	C	T	0.00028	0.99972	0.00028	60.00	151.00	1.0000	20.00	20.00	0.1178 c
12	692504	chr12:562765	562765	A	T	0.00045	0.99955	0.00045	60.00	178.00	1.0000	17.00	17.00	0.0239 a
12	692521	chr12:562782	562782	C	G	0.00039	0.99961	0.00039	60.00	228.00	1.0000	39.00	39.00	0.0000 c
12	692537	chr12:562798	562798	C	T	0.00039	0.99961	0.00039	60.00	228.00	1.0000	57.00	57.00	0.0000 c
12	692561	chr12:562822	562822	G	T	0.00026	0.99974	0.00026	60.00	156.00	1.0000	12.00	12.00	0.0464 g
12	692563	chr12:562824	562824	G	A	0.00062	0.99938	0.00062	60.00	228.00	1.0000	33.28	39.91	0.0012 g
12	692617	chr12:562878	562878	C	T	0.00030	0.99970	0.00030	60.00	58.00	1.0000	13.00	13.00	0.1672 c
12	692648	chr12:562909	562909	G	A	0.19148	0.80852	0.19148	60.00	201.46	0.7795	10.00	34.00	0.0671 g
12	692670	chr12:562931	562931	A	C	0.00118	0.99882	0.00118	60.00	186.67	1.0000	11.10	19.65	0.0224 a
12	692686	chr12:562947	562947	C	T	0.00031	0.99969	0.00031	60.00	228.00	1.0000	18.00	18.00	0.1862 c
12	692687	chr12:562948	562948	G	A	0.00031	0.99969	0.00031	60.00	177.00	1.0000	10.00	10.00	0.1817 g
12	692774	chr12:563035	563035	C	T	0.00046	0.99954	0.00046	60.00	228.00	1.0000	83.00	83.00	0.0309 c
12	692803	chr12:563064	563064	T	C	0.00039	0.99961	0.00039	60.00	228.00	1.0000	38.00	38.00	0.0008 t
12	692823	chr12:563084	563084	G	A	0.21331	0.78669	0.21331	60.00	210.01	0.4664	13.48	52.50	0.0696 g
12	692841	chr12:563102	563102	C	A	0.00045	0.99955	0.00045	60.00	228.00	1.0000	29.00	29.00	0.0106 c
12	692887	chr12:563148	563148	G	A	0.00072	0.99928	0.00072	60.00	213.36	1.0000	19.08	27.34	0.0532 g
12	692897	chr12:563158	563158	T	G	0.00039	0.99961	0.00039	60.00	228.00	1.0000	62.00	62.00	0.0000 t
12	692901	chr12:563162	563162	A	G	0.00039	0.99961	0.00039	60.00	158.00	1.0000	31.00	31.00	0.0000 a
12	692908	chr12:563169	563169	G	A	0.36193	0.63807	0.36193	60.00	193.33	0.0209	12.63	49.10	0.0933 g
12	692940	chr12:563201	563201	G	A	0.22222	0.77778	0.22222	60.00	218.19	0.6043	19.00	60.00	0.0000 g
12	692962	chr12:563223	563223	G	A	0.20875	0.79125	0.20875	60.00	206.15	0.6606	13.17	52.27	0.0162 g
12	692967	chr12:563228	563228	C	T	0.00832	0.99168	0.00832	60.00	225.67	1.0000	18.47	60.78	0.0098 c
12	693000	chr12:563261	563261	G	T	0.00044	0.99956	0.00044	60.00	228.00	1.0000	29.00	29.00	0.0053 g
12	693032	chr12:563293	563293	T	G	0.00026	0.99974	0.00026	60.00	47.00	1.0000	10.00	10.00	0.0215 t
12	693035	chr12:563296	563296	G	T	0.00026	0.99974	0.00026	60.00	94.00	1.0000	11.00	11.00	0.0215 g
12	693080	chr12:563341	563341	G	A	0.21415	0.78585	0.21415	60.00	215.40	0.3932	18.28	69.05	0.0222 a
12	693113	chr12:563374	563374	A	G	0.00044	0.99956	0.00044	60.00	228.00	1.0000	17.00	17.00	0.0027 a

12	693139	chr12:563400	563400	C	T	0.00025	0.99975	0.00025	60.00	228.00	1.0000	27.00	27.00	0.0175	c
12	693149	chr12:563410	563410	C	T	0.00044	0.99956	0.00044	60.00	228.00	1.0000	27.00	27.00	0.0035	c
12	693186	chr12:563447	563447	A	G	0.00028	0.99972	0.00028	60.00	228.00	1.0000	14.00	14.00	0.0964	a
12	693213	chr12:563474	563474	G	A	0.00064	0.99936	0.00064	59.70	215.29	1.0000	19.90	25.00	0.0560	g
12	693224	chr12:563485	563485	G	A	0.00039	0.99961	0.00039	60.00	228.00	1.0000	21.00	21.00	0.0170	g
12	693226	chr12:563487	563487	A	G	0.00046	0.99954	0.00046	60.00	192.00	1.0000	22.00	22.00	0.0442	a
12	693287	chr12:563548	563548	G	A	0.00025	0.99975	0.00025	60.00	228.00	1.0000	56.00	56.00	0.0040	g
12	693327	chr12:563588	563588	T	C	0.00044	0.99956	0.00044	60.00	228.00	1.0000	54.00	54.00	0.0009	t
12	693349	chr12:563610	563610	G	A	0.00039	0.99961	0.00039	60.00	225.00	1.0000	79.00	79.00	0.0000	g
12	694339	chr12:564600	564600	C	T	0.00116	0.99884	0.00116	60.00	228.00	1.0000	40.20	45.90	0.0000	c
12	694428	chr12:564689	564689	G	T	0.00044	0.99956	0.00044	60.00	228.00	1.0000	26.00	26.00	0.0018	G
12	694458	chr12:564719	564719	C	T	0.00039	0.99961	0.00039	59.00	228.00	1.0000	39.00	39.00	0.0000	C
12	694550	chr12:564811	564811	G	A	0.00033	0.99967	0.00033	60.00	228.00	1.0000	27.22	27.22	0.0187	G
12	694554	chr12:564815	564815	A	G	0.00108	0.99892	0.00108	60.00	215.08	1.0000	24.10	30.96	0.0150	A
12	694589	chr12:564850	564850	C	T	0.00032	0.99968	0.00032	60.00	228.00	1.0000	33.58	33.58	0.0181	C
12	694605	chr12:564866	564866	G	A	0.00026	0.99974	0.00026	60.00	175.00	1.0000	17.00	17.00	0.0240	G
12	694636	chr12:564897	564897	T	C	0.00041	0.99959	0.00041	59.54	228.00	1.0000	35.05	35.05	0.0025	T
12	694719	chr12:564980	564980	C	T	0.18002	0.81998	0.18002	59.54	195.39	0.5801	10.01	30.28	0.0801	c
12	694720	chr12:564981	564981	G	C	0.05886	0.94114	0.05886	59.60	200.71	0.3291	10.33	29.28	0.1178	g
12	694749	chr12:565010	565010	C	A	0.00727	0.99273	0.00727	60.00	223.55	1.0000	16.02	54.37	0.0214	C
12	694757	chr12:565018	565018	C	T	0.00861	0.99139	0.00861	60.00	222.06	1.0000	15.73	60.75	0.0170	C
12	694785	chr12:565046	565046	G	A	0.00030	0.99970	0.00030	60.00	228.00	1.0000	54.63	54.63	0.0039	G
12	694820	chr12:565081	565081	G	A	0.00044	0.99956	0.00044	60.00	228.00	1.0000	45.00	45.00	0.0035	G
12	694854	chr12:565115	565115	C	T	0.22099	0.77901	0.22099	60.00	219.95	0.3657	22.65	71.61	0.0099	C
12	694958	chr12:565219	565219	G	C	0.00032	0.99968	0.00032	60.00	228.00	1.0000	35.23	35.23	0.0022	G
12	694976	chr12:565237	565237	G	A	0.00249	0.99751	0.00249	60.00	221.30	1.0000	37.64	56.75	0.0020	G
12	695006	chr12:565267	565267	G	T	0.00044	0.99956	0.00044	60.00	228.00	1.0000	28.00	28.00	0.0053	G
12	695014	chr12:565275	565275	A	G	0.00045	0.99955	0.00045	60.00	228.00	1.0000	20.00	20.00	0.0080	A
12	695019	chr12:565280	565280	G	A	0.00025	0.99975	0.00025	60.00	228.00	1.0000	14.00	14.00	0.0130	G
12	695022	chr12:565283	565283	C	T	0.00039	0.99961	0.00039	60.00	228.00	1.0000	47.00	47.00	0.0000	C
12	695039	chr12:565300	565300	G	A	0.00025	0.99975	0.00025	60.00	228.00	1.0000	15.00	15.00	0.0165	G
12	695089	chr12:565350	565350	A	G	0.21598	0.78402	0.21598	60.00	214.71	0.4913	17.51	64.97	0.0130	A
12	695097	chr12:565358	565358	A	G	0.21662	0.78338	0.21662	60.00	214.15	0.5137	17.08	64.52	0.0128	a
12	695150	chr12:565411	565411	C	G	0.00083	0.99917	0.00083	60.00	217.54	1.0000	28.32	42.03	0.0025	C

12	695215	chr12:565476	565476	T	C	0.00116	0.99884	0.00116	60.00	228.00	1.0000	32.65	49.75	0.0000	T
12	695248	chr12:565509	565509	G	A	0.18572	0.81428	0.18572	60.00	197.83	0.0122	12.64	48.28	0.0566	G
12	695254	chr12:565515	565515	G	A	0.14441	0.85559	0.14441	60.00	193.82	0.0002	13.56	45.94	0.1007	A
12	695264	chr12:565525	565525	G	A	0.15171	0.84829	0.15171	60.00	197.34	0.0001	12.65	46.14	0.0951	A
12	695276	chr12:565537	565537	G	A	0.00766	0.99234	0.00766	60.00	225.25	1.0000	14.05	51.67	0.0132	G
12	695294	chr12:565555	565555	G	C	0.00050	0.99950	0.00050	60.00	228.00	1.0000	25.79	27.89	0.0349	G
12	695301	chr12:565562	565562	G	A	0.00039	0.99961	0.00039	60.00	54.00	1.0000	14.00	14.00	0.0046	G
12	695314	chr12:565575	565575	G	A	0.00050	0.99950	0.00050	60.00	228.00	1.0000	24.32	27.33	0.0474	A
12	695382	chr12:565643	565643	T	A	0.00046	0.99954	0.00046	60.00	228.00	1.0000	15.00	15.00	0.0300	T
12	695396	chr12:565657	565657	G	A	0.00045	0.99955	0.00045	60.00	228.00	1.0000	34.00	34.00	0.0247	G
12	695402	chr12:565663	565663	G	A	0.21214	0.78786	0.21214	60.00	209.42	0.0471	12.48	53.49	0.0749	A
12	695440	chr12:565701	565701	G	A	0.00044	0.99956	0.00044	60.00	228.00	1.0000	39.00	39.00	0.0053	G
12	695466	chr12:565727	565727	A	G	0.00032	0.99968	0.00032	60.00	228.00	1.0000	54.45	54.45	0.0083	A
12	695535	chr12:565796	565796	G	A	0.00098	0.99902	0.00098	60.00	199.51	1.0000	13.24	18.70	0.0741	G
12	695621	chr12:565882	565882	G	A	0.00027	0.99973	0.00027	60.00	228.00	1.0000	43.00	43.00	0.0589	G
12	695655	chr12:565916	565916	G	T	0.00244	0.99756	0.00244	60.00	213.69	1.0000	15.10	48.81	0.0292	G
12	695661	chr12:565922	565922	C	T	0.00026	0.99974	0.00026	60.00	228.00	1.0000	17.00	17.00	0.0554	C
12	695662	chr12:565923	565923	G	A	0.00045	0.99955	0.00045	60.00	228.00	1.0000	20.00	20.00	0.0141	G
12	695675	chr12:565936	565936	G	T	0.00045	0.99955	0.00045	60.00	228.00	1.0000	29.00	29.00	0.0124	G
12	695707	chr12:565968	565968	C	A	0.00039	0.99961	0.00039	60.00	228.00	1.0000	22.00	22.00	0.0139	C
12	695720	chr12:565981	565981	G	C	0.00039	0.99961	0.00039	60.00	63.00	1.0000	12.00	12.00	0.0147	G
12	695726	chr12:565987	565987	G	A	0.00047	0.99953	0.00047	60.00	228.00	1.0000	25.00	25.00	0.0504	G
12	695742	chr12:566003	566003	G	A	0.00027	0.99973	0.00027	60.00	228.00	1.0000	50.00	50.00	0.0759	G
12	695774	chr12:566035	566035	C	T	0.00128	0.99872	0.00128	60.00	225.36	1.0000	19.19	43.79	0.0309	C
12	695800	chr12:566061	566061	T	C	0.00026	0.99974	0.00026	60.00	228.00	1.0000	23.00	23.00	0.0479	T
12	695864	chr12:566125	566125	G	A	0.21638	0.78362	0.21638	60.00	214.70	0.4740	16.19	63.39	0.0278	G
12	695916	chr12:566177	566177	A	G	0.00026	0.99974	0.00026	60.00	228.00	1.0000	36.00	36.00	0.0474	A
12	695957	chr12:566218	566218	C	T	0.00045	0.99955	0.00045	60.00	208.00	1.0000	18.00	18.00	0.0150	C
12	695962	chr12:566223	566223	A	T	0.00045	0.99955	0.00045	60.00	112.00	1.0000	14.00	14.00	0.0177	A
12	695969	chr12:566230	566230	C	T	0.00045	0.99955	0.00045	60.00	228.00	1.0000	31.00	31.00	0.0133	C
12	696015	chr12:566276	566276	C	T	0.00044	0.99956	0.00044	60.00	228.00	1.0000	39.00	39.00	0.0044	C
12	696016	chr12:566277	566277	G	A	0.00077	0.99923	0.00077	60.00	228.00	1.0000	34.53	54.48	0.0000	G
12	696024	chr12:566285	566285	G	A	0.00044	0.99956	0.00044	60.00	91.00	1.0000	14.00	14.00	0.0044	g
12	696038	chr12:566299	566299	C	T	0.00044	0.99956	0.00044	60.00	111.00	1.0000	20.00	20.00	0.0035	c

12	696042	chr12:566303	566303	G	A	0.00025	0.99975	0.00025	60.00	145.00	1.0000	17.00	17.00	0.0135	g
12	696050	chr12:566311	566311	C	T	0.21683	0.78317	0.21683	60.00	218.92	0.3730	22.01	74.30	0.0183	c
12	696062	chr12:566323	566323	T	A	0.00044	0.99956	0.00044	60.00	228.00	1.0000	71.00	71.00	0.0027	t
12	696078	chr12:566339	566339	A	G	0.77966	0.22034	0.22034	60.00	204.37	0.0981	25.94	85.06	0.0125	g
12	696145	chr12:566406	566406	G	A	0.00025	0.99975	0.00025	60.00	228.00	1.0000	41.00	41.00	0.0135	g
12	696146	chr12:566407	566407	T	C	0.00051	0.99949	0.00051	60.00	205.50	1.0000	16.38	30.63	0.0135	t
12	696166	chr12:566427	566427	C	T	0.00026	0.99974	0.00026	60.00	181.00	1.0000	17.00	17.00	0.0459	c
12	696196	chr12:566457	566457	T	C	0.18814	0.81186	0.18814	60.00	193.75	0.0393	11.93	46.08	0.0576	c
12	696241	chr12:566502	566502	C	T	0.21974	0.78026	0.21974	60.00	216.37	0.5479	21.34	64.27	0.0029	c
12	696243	chr12:566504	566504	C	T	0.00044	0.99956	0.00044	60.00	228.00	1.0000	72.00	72.00	0.0027	c
12	696268	chr12:566529	566529	C	T	0.00032	0.99968	0.00032	60.00	228.00	1.0000	46.90	46.90	0.0057	c
12	696305	chr12:566566	566566	A	C	0.00044	0.99956	0.00044	60.00	228.00	1.0000	40.00	40.00	0.0018	a
12	696339	chr12:566600	566600	T	C	0.00025	0.99975	0.00025	60.00	42.00	1.0000	10.00	10.00	0.0095	t
12	696372	chr12:566633	566633	C	A	0.00039	0.99961	0.00039	60.00	228.00	1.0000	40.00	40.00	0.0008	c
12	696383	chr12:566644	566644	A	T	0.15111	0.84889	0.15111	60.00	202.03	0.0119	12.31	41.25	0.0671	a
12	696523	chr12:566784	566784	C	T	0.00041	0.99959	0.00041	60.00	228.00	1.0000	27.00	27.00	0.0494	c
12	696533	chr12:566794	566794	C	T	0.00051	0.99949	0.00051	60.00	228.00	1.0000	26.00	26.00	0.1263	c
12	696575	chr12:566836	566836	A	T	0.00087	0.99913	0.00087	60.00	224.50	1.0000	10.35	23.65	0.1103	a
12	696585	chr12:566846	566846	G	A	0.00040	0.99960	0.00040	60.00	60.00	1.0000	10.00	10.00	0.0401	g
12	696597	chr12:566858	566858	A	T	0.00029	0.99971	0.00029	60.00	141.00	1.0000	14.00	14.00	0.1478	a
12	696659	chr12:566920	566920	C	T	0.18464	0.81536	0.18464	60.00	200.25	0.3131	11.15	42.72	0.0757	t
12	696669	chr12:566930	566930	G	A	0.00028	0.99972	0.00028	60.00	43.00	1.0000	10.00	10.00	0.1213	g
12	696674	chr12:566935	566935	A	G	0.14779	0.85221	0.14779	60.00	185.44	0.6591	10.00	25.57	0.0384	a
12	696683	chr12:566944	566944	A	G	0.00046	0.99954	0.00046	60.00	228.00	1.0000	16.00	16.00	0.0318	a
12	696693	chr12:566954	566954	C	T	0.00080	0.99920	0.00080	60.00	62.00	1.0000	10.15	16.80	0.0594	c
12	696724	chr12:566985	566985	A	T	0.00034	0.99966	0.00034	60.00	228.00	1.0000	38.95	38.95	0.0112	a
12	696731	chr12:566992	566992	C	G	0.21192	0.78808	0.21192	60.00	209.14	0.3272	15.15	55.72	0.0300	g
12	696769	chr12:567030	567030	G	A	0.00027	0.99973	0.00027	60.00	228.00	1.0000	21.00	21.00	0.0694	g
12	696783	chr12:567044	567044	G	C	0.00054	0.99946	0.00054	60.00	208.81	1.0000	13.17	23.15	0.1198	g
12	696886	chr12:567147	567147	G	A	0.44678	0.55322	0.44678	60.00	162.40	0.0030	10.00	29.00	0.1373	g
12	696910	chr12:567171	567171	C	T	0.46850	0.53150	0.46850	60.00	169.96	0.0001	12.28	40.92	0.0768	t
12	696912	chr12:567173	567173	G	C	0.47558	0.52442	0.47558	60.00	169.55	0.0001	12.75	41.93	0.0800	g
12	696960	chr12:567221	567221	G	T	0.00026	0.99974	0.00026	60.00	74.00	1.0000	14.00	14.00	0.0225	g
12	696961	chr12:567222	567222	C	T	0.00045	0.99955	0.00045	60.00	228.00	1.0000	33.00	33.00	0.0097	c

12	696973	chr12:567234	567234	G	A	0.00077	0.99923	0.00077	60.00	228.00	1.0000	46.15	51.85	0.0000	g
12	696993	chr12:567254	567254	C	A	0.00044	0.99956	0.00044	60.00	228.00	1.0000	55.00	55.00	0.0000	c
12	697020	chr12:567281	567281	C	T	0.00062	0.99938	0.00062	60.00	228.00	1.0000	50.03	50.92	0.0000	c
12	697027	chr12:567288	567288	T	C	0.00088	0.99912	0.00088	60.00	228.00	1.0000	36.10	39.90	0.0000	t
12	697074	chr12:567335	567335	T	C	0.00025	0.99975	0.00025	60.00	228.00	1.0000	42.00	42.00	0.0005	t
12	697075	chr12:567336	567336	G	C	0.00025	0.99975	0.00025	60.00	228.00	1.0000	67.00	67.00	0.0005	g
12	697084	chr12:567345	567345	C	G	0.00025	0.99975	0.00025	60.00	228.00	1.0000	36.00	36.00	0.0005	c
12	697095	chr12:567356	567356	C	T	0.28122	0.71878	0.28122	60.00	218.60	0.3317	28.10	80.37	0.0061	c
12	697097	chr12:567358	567358	G	A	0.00048	0.99952	0.00048	60.00	228.00	1.0000	37.88	48.20	0.0003	g
12	697122	chr12:567383	567383	T	C	0.25399	0.74601	0.25399	60.00	213.84	0.3224	24.58	73.29	0.0515	c
12	697131	chr12:567392	567392	G	A	0.00044	0.99956	0.00044	60.00	228.00	1.0000	59.00	59.00	0.0000	g
12	697157	chr12:567418	567418	C	T	0.01174	0.98826	0.01174	60.00	228.00	1.0000	22.75	69.65	0.0005	c
12	697158	chr12:567419	567419	G	A	0.27955	0.72045	0.27955	60.00	210.44	0.6291	18.00	66.00	0.0160	g
12	697175	chr12:567436	567436	A	G	0.00044	0.99956	0.00044	60.00	228.00	1.0000	52.00	52.00	0.0027	a
12	697222	chr12:567483	567483	C	T	0.00044	0.99956	0.00044	60.00	228.00	1.0000	41.00	41.00	0.0000	c
12	697230	chr12:567491	567491	C	T	0.00407	0.99593	0.00407	60.00	228.00	1.0000	24.05	60.26	0.0007	c
12	697315	chr12:567576	567576	C	T	0.20475	0.79525	0.20475	59.97	207.35	0.0120	14.20	44.97	0.0487	c
12	697316	chr12:567577	567577	G	A	0.00025	0.99975	0.00025	60.00	228.00	1.0000	32.00	32.00	0.0075	g
12	697317	chr12:567578	567578	G	A	0.00039	0.99961	0.00039	60.00	228.00	1.0000	32.00	32.00	0.0008	g
12	697329	chr12:567590	567590	G	C	0.09919	0.90081	0.09919	59.84	190.24	0.0055	11.16	28.78	0.0742	g
12	697331	chr12:567592	567592	G	T	0.09534	0.90466	0.09534	59.81	189.69	0.0020	10.58	27.22	0.0729	g
12	697351	chr12:567612	567612	C	G	0.00093	0.99907	0.00093	59.72	143.63	0.0031	10.00	10.00	0.1066	c
12	697382	chr12:567643	567643	G	A	0.00042	0.99958	0.00042	60.00	211.00	1.0000	12.00	12.00	0.0880	A
12	697442	chr12:567703	567703	C	T	0.00050	0.99950	0.00050	60.00	228.00	1.0000	23.35	36.65	0.0030	C
12	697443	chr12:567704	567704	G	A	0.00039	0.99961	0.00039	60.00	162.00	1.0000	27.00	27.00	0.0000	G
12	697485	chr12:567746	567746	T	C	0.48866	0.51134	0.48866	60.00	207.91	0.3993	23.61	75.37	0.0045	C
12	697526	chr12:567787	567787	C	T	0.00025	0.99975	0.00025	60.00	228.00	1.0000	37.00	37.00	0.0000	c
12	697548	chr12:567809	567809	C	G	0.00044	0.99956	0.00044	60.00	30.00	1.0000	10.00	10.00	0.0027	C
12	697562	chr12:567823	567823	A	C	0.00044	0.99956	0.00044	60.00	228.00	1.0000	39.00	39.00	0.0044	A
12	697611	chr12:567872	567872	C	T	0.00039	0.99961	0.00039	60.00	228.00	1.0000	48.00	48.00	0.0000	C
12	697612	chr12:567873	567873	G	A	0.00088	0.99912	0.00088	60.00	228.00	1.0000	43.00	43.00	0.0000	G
12	697633	chr12:567894	567894	G	C	0.48853	0.51147	0.48853	60.00	208.72	0.3988	26.11	73.13	0.0061	G
12	697672	chr12:567933	567933	A	G	0.00025	0.99975	0.00025	60.00	228.00	1.0000	54.00	54.00	0.0000	A
12	697686	chr12:567947	567947	G	C	0.00790	0.99210	0.00790	60.00	227.75	1.0000	26.50	71.55	0.0002	G

12	697693	chr12:567954	567954	A	C	0.00044	0.99956	0.00044	60.00	228.00	1.0000	27.00	27.00	0.0018 A
12	697701	chr12:567962	567962	T	A	0.00039	0.99961	0.00039	60.00	228.00	1.0000	43.00	43.00	0.0000 T
12	697809	chr12:568070	568070	C	T	0.00044	0.99956	0.00044	60.00	228.00	1.0000	28.00	28.00	0.0027 C
12	697811	chr12:568072	568072	A	G	0.00039	0.99961	0.00039	60.00	228.00	1.0000	63.00	63.00	0.0000 A
12	697846	chr12:568107	568107	G	A	0.00044	0.99956	0.00044	60.00	115.00	1.0000	12.00	12.00	0.0027 G
12	697935	chr12:568196	568196	G	A	0.00039	0.99961	0.00039	60.00	228.00	1.0000	54.00	54.00	0.0000 G
12	697937	chr12:568198	568198	G	A	0.00384	0.99616	0.00384	60.00	228.00	1.0000	28.64	68.78	0.0000 G
12	697952	chr12:568213	568213	C	A	0.00025	0.99975	0.00025	60.00	228.00	1.0000	60.00	60.00	0.0000 C
12	697964	chr12:568225	568225	C	T	0.00041	0.99959	0.00041	60.00	228.00	1.0000	47.61	47.61	0.0000 C
12	698008	chr12:568269	568269	G	A	0.00045	0.99955	0.00045	60.00	228.00	1.0000	57.17	63.51	0.0000 G
12	698018	chr12:568279	568279	G	A	0.00039	0.99961	0.00039	60.00	228.00	1.0000	61.00	61.00	0.0000 G
12	698020	chr12:568281	568281	G	A	0.00039	0.99961	0.00039	60.00	228.00	1.0000	57.00	57.00	0.0000 G
12	698043	chr12:568304	568304	G	C	0.06153	0.93847	0.06153	60.00	225.30	0.3097	27.75	74.88	0.0005 G
12	698058	chr12:568319	568319	C	A	0.00044	0.99956	0.00044	60.00	228.00	1.0000	24.00	24.00	0.0000 C
12	698075	chr12:568336	568336	T	A	0.00025	0.99975	0.00025	60.00	61.00	1.0000	15.00	15.00	0.0050 T
12	698114	chr12:568375	568375	G	A	0.00039	0.99961	0.00039	60.00	228.00	1.0000	27.00	27.00	0.0023 G
12	698141	chr12:568402	568402	A	G	0.00039	0.99961	0.00039	60.00	228.00	1.0000	19.00	19.00	0.0023 A
12	698263	chr12:568524	568524	C	T	0.00025	0.99975	0.00025	60.00	228.00	1.0000	51.00	51.00	0.0015 A
12	698275	chr12:568536	568536	T	C	0.00039	0.99961	0.00039	60.00	228.00	1.0000	60.00	60.00	0.0000 T
12	698319	chr12:568580	568580	G	A	0.00025	0.99975	0.00025	60.00	228.00	1.0000	32.00	32.00	0.0035 G
12	698349	chr12:568610	568610	C	T	0.00135	0.99865	0.00135	60.00	228.00	1.0000	35.71	50.17	0.0005 C
12	698351	chr12:568612	568612	C	T	0.00025	0.99975	0.00025	60.00	211.00	1.0000	38.00	38.00	0.0015 C
12	698492	chr12:568753	568753	T	A	0.00044	0.99956	0.00044	60.00	50.00	1.0000	10.00	10.00	0.0035 t
12	698502	chr12:568763	568763	C	A	0.00057	0.99943	0.00057	60.00	228.00	1.0000	33.25	42.41	0.0032 C
12	698511	chr12:568772	568772	A	G	0.00025	0.99975	0.00025	60.00	228.00	1.0000	29.00	29.00	0.0035 A
12	698595	chr12:568856	568856	C	T	0.00025	0.99975	0.00025	60.00	228.00	1.0000	53.00	53.00	0.0000 C
12	698634	chr12:568895	568895	A	G	0.76861	0.23139	0.23139	60.00	210.02	0.1004	31.49	83.21	0.0027 G
12	698680	chr12:568941	568941	T	A	0.00025	0.99975	0.00025	60.00	228.00	1.0000	47.00	47.00	0.0005 T
12	698721	chr12:568982	568982	C	T	0.00025	0.99975	0.00025	60.00	218.00	1.0000	29.00	29.00	0.0005 C
12	698778	chr12:569039	569039	A	C	0.00025	0.99975	0.00025	60.00	54.00	1.0000	10.00	10.00	0.0045 A
12	698785	chr12:569046	569046	C	G	0.00025	0.99975	0.00025	60.00	58.00	1.0000	10.00	10.00	0.0065 C
12	698833	chr12:569094	569094	C	T	0.00138	0.99862	0.00138	60.00	228.00	1.0000	25.50	38.80	0.0406 C
12	698865	chr12:569126	569126	T	C	0.00039	0.99961	0.00039	60.00	228.00	1.0000	32.00	32.00	0.0046 T
12	698879	chr12:569140	569140	A	C	0.19477	0.80523	0.19477	60.00	200.64	0.5333	12.26	40.67	0.0382 A

12	698883	chr12:569144	569144	A	C	0.00042	0.99958	0.00042	60.00	228.00	1.0000	23.37	23.37	0.0182	A
12	698908	chr12:569169	569169	C	T	0.00027	0.99973	0.00027	60.00	228.00	1.0000	31.00	31.00	0.0654	C
12	698952	chr12:569213	569213	A	G	0.00026	0.99974	0.00026	60.00	223.00	1.0000	11.00	11.00	0.0489	A
12	699073	chr12:569334	569334	G	A	0.00041	0.99959	0.00041	60.00	141.00	1.0000	15.00	15.00	0.0702	G
12	699087	chr12:569348	569348	G	A	0.00055	0.99945	0.00055	60.00	108.00	1.0000	14.00	14.00	0.1908	G
12	699112	chr12:569373	569373	G	A	0.00031	0.99969	0.00031	60.00	34.00	1.0000	13.00	13.00	0.1817	G
12	699139	chr12:569400	569400	G	T	0.00027	0.99973	0.00027	60.00	44.00	1.0000	10.00	10.00	0.0824	G
12	699168	chr12:569429	569429	G	A	0.00026	0.99974	0.00026	60.00	228.00	1.0000	51.00	51.00	0.0359	G
12	699169	chr12:569430	569430	A	G	0.00026	0.99974	0.00026	60.00	135.00	1.0000	17.00	17.00	0.0334	A
12	699232	chr12:569493	569493	G	A	0.00025	0.99975	0.00025	60.00	228.00	1.0000	31.00	31.00	0.0005	G
12	699331	chr12:569592	569592	T	C	0.00274	0.99726	0.00274	59.48	208.54	1.0000	12.79	31.38	0.0523	T
12	699430	chr12:569691	569691	C	T	0.00044	0.99956	0.00044	60.00	228.00	1.0000	35.00	35.00	0.1281	C
12	699440	chr12:569701	569701	C	T	0.00045	0.99955	0.00045	60.00	228.00	1.0000	20.00	20.00	0.1335	c
12	699491	chr12:569752	569752	A	G	0.00028	0.99972	0.00028	60.00	218.00	1.0000	18.00	18.00	0.0939	A
12	699510	chr12:569771	569771	A	C	0.00045	0.99955	0.00045	60.00	228.00	1.0000	32.00	32.00	0.0150	A
12	699515	chr12:569776	569776	G	T	0.00027	0.99973	0.00027	60.00	228.00	1.0000	15.00	15.00	0.0749	G
12	699517	chr12:569778	569778	G	A	0.00045	0.99955	0.00045	60.00	180.00	1.0000	18.00	18.00	0.0106	G
12	699548	chr12:569809	569809	G	A	0.00359	0.99641	0.00359	60.00	219.29	1.0000	14.36	51.42	0.0259	G
12	699587	chr12:569848	569848	C	T	0.00025	0.99975	0.00025	60.00	228.00	1.0000	15.00	15.00	0.0210	C
12	699608	chr12:569869	569869	G	C	0.00025	0.99975	0.00025	60.00	228.00	1.0000	40.00	40.00	0.0095	G
12	699677	chr12:569938	569938	T	C	0.00045	0.99955	0.00045	60.00	228.00	1.0000	52.97	60.68	0.0029	T
12	699683	chr12:569944	569944	A	G	0.06192	0.93808	0.06192	60.00	225.29	0.3097	24.48	76.03	0.0049	A
12	699766	chr12:570027	570027	C	G	0.00026	0.99974	0.00026	60.00	139.00	1.0000	19.00	19.00	0.0559	C
12	699768	chr12:570029	570029	G	T	0.00029	0.99971	0.00029	60.00	228.00	1.0000	17.00	17.00	0.1478	G
12	699789	chr12:570050	570050	G	A	0.00028	0.99972	0.00028	60.00	171.00	1.0000	24.00	24.00	0.0964	G
12	699791	chr12:570052	570052	C	T	0.46959	0.53041	0.46959	60.00	182.95	0.3176	10.96	45.80	0.0377	C
12	700235	chr12:570496	570496	C	G	0.15306	0.84694	0.15306	59.46	187.69	0.5142	11.62	34.00	0.0580	C
12	700239	chr12:570500	570500	C	T	0.25821	0.74179	0.25821	59.56	198.06	0.4652	13.66	39.68	0.0442	T
12	700282	chr12:570543	570543	C	T	0.00041	0.99959	0.00041	60.00	228.00	1.0000	63.95	63.95	0.0000	C
12	700297	chr12:570558	570558	T	G	0.00045	0.99955	0.00045	60.00	228.00	1.0000	55.33	66.25	0.0005	T
12	700360	chr12:570621	570621	G	A	0.00734	0.99266	0.00734	60.00	227.97	1.0000	30.43	71.79	0.0011	G
12	700372	chr12:570633	570633	A	G	0.00025	0.99975	0.00025	60.00	219.00	1.0000	26.00	26.00	0.0015	G
12	700415	chr12:570676	570676	C	T	0.00025	0.99975	0.00025	60.00	218.00	1.0000	33.00	33.00	0.0070	C
12	700433	chr12:570694	570694	C	T	0.00039	0.99961	0.00039	60.00	228.00	1.0000	36.00	36.00	0.0000	C

12	700457	chr12:570718	570718	G	A	0.00025	0.99975	0.00025	60.00	228.00	1.0000	26.00	26.00	0.0200 G
12	700459	chr12:570720	570720	G	A	0.00039	0.99961	0.00039	60.00	228.00	1.0000	50.00	50.00	0.0000 G
12	700528	chr12:570789	570789	T	C	0.06105	0.93895	0.06105	60.00	221.25	0.3097	17.31	65.01	0.0147 N
12	700542	chr12:570803	570803	A	T	0.00039	0.99961	0.00039	60.00	228.00	1.0000	30.00	30.00	0.0000 A
12	700566	chr12:570827	570827	C	T	0.00044	0.99956	0.00044	60.00	228.00	1.0000	17.00	17.00	0.0027 C
12	700574	chr12:570835	570835	T	C	0.00044	0.99956	0.00044	60.00	104.00	1.0000	13.00	13.00	0.0053 T
12	700585	chr12:570846	570846	G	T	0.00026	0.99974	0.00026	60.00	48.00	1.0000	10.00	10.00	0.0315 G
12	700628	chr12:570889	570889	G	C	0.00039	0.99961	0.00039	60.00	46.00	1.0000	11.00	11.00	0.0023 G
12	700633	chr12:570894	570894	C	G	0.00028	0.99972	0.00028	60.00	73.00	1.0000	15.00	15.00	0.1073 C
12	700638	chr12:570899	570899	A	T	0.00045	0.99955	0.00045	60.00	47.00	1.0000	14.00	14.00	0.0177 A
12	700675	chr12:570936	570936	G	C	0.77889	0.22111	0.22111	60.00	189.36	0.0137	20.99	73.47	0.0118 C
12	700692	chr12:570953	570953	G	A	0.00025	0.99975	0.00025	60.00	228.00	1.0000	49.00	49.00	0.0065 G
12	700740	chr12:571001	571001	G	A	0.00025	0.99975	0.00025	60.00	228.00	1.0000	65.00	65.00	0.0100 G
12	700751	chr12:571012	571012	C	A	0.00044	0.99956	0.00044	60.00	228.00	1.0000	37.00	37.00	0.0000 C
12	700770	chr12:571031	571031	C	A	0.00317	0.99683	0.00317	60.00	224.34	1.0000	23.72	59.95	0.0043 C
12	700782	chr12:571043	571043	G	A	0.00025	0.99975	0.00025	60.00	228.00	1.0000	60.00	60.00	0.0115 G
12	700787	chr12:571048	571048	G	A	0.00113	0.99887	0.00113	60.00	228.00	1.0000	35.38	54.23	0.0079 G
12	700796	chr12:571057	571057	G	A	0.00039	0.99961	0.00039	60.00	228.00	1.0000	48.00	48.00	0.0000 G
12	700807	chr12:571068	571068	T	C	0.00089	0.99911	0.00089	60.00	228.00	1.0000	33.45	50.55	0.0027 T
12	700815	chr12:571076	571076	G	A	0.00025	0.99975	0.00025	60.00	228.00	1.0000	72.00	72.00	0.0190 G
12	700841	chr12:571102	571102	C	A	0.00026	0.99974	0.00026	60.00	228.00	1.0000	89.00	89.00	0.0240 C
12	700870	chr12:571131	571131	G	A	0.00026	0.99974	0.00026	60.00	38.00	1.0000	10.00	10.00	0.0374 G
12	700880	chr12:571141	571141	G	A	0.00026	0.99974	0.00026	60.00	84.00	1.0000	15.00	15.00	0.0449 G
12	700884	chr12:571145	571145	G	T	0.00026	0.99974	0.00026	60.00	213.00	1.0000	22.00	22.00	0.0444 G
12	700897	chr12:571158	571158	G	A	0.00033	0.99967	0.00033	60.00	228.00	1.0000	42.59	42.59	0.0290 G
12	700900	chr12:571161	571161	C	A	0.00044	0.99956	0.00044	60.00	228.00	1.0000	19.00	19.00	0.0053 C
12	700958	chr12:571219	571219	T	C	0.00025	0.99975	0.00025	60.00	228.00	1.0000	42.00	42.00	0.0085 T
12	700963	chr12:571224	571224	T	G	0.00039	0.99961	0.00039	60.00	228.00	1.0000	83.00	83.00	0.0000 T
12	701017	chr12:571278	571278	G	C	0.00025	0.99975	0.00025	60.00	228.00	1.0000	36.00	36.00	0.0035 G
12	701030	chr12:571291	571291	G	C	0.00044	0.99956	0.00044	60.00	228.00	1.0000	56.00	56.00	0.0009 G
12	701037	chr12:571298	571298	G	A	0.00025	0.99975	0.00025	60.00	228.00	1.0000	23.00	23.00	0.0040 G
12	701086	chr12:571347	571347	A	G	0.00781	0.99219	0.00781	60.00	223.57	1.0000	20.89	61.40	0.0029 A
12	701089	chr12:571350	571350	C	T	0.00025	0.99975	0.00025	60.00	228.00	1.0000	40.00	40.00	0.0075 C
12	701090	chr12:571351	571351	G	A	0.00044	0.99956	0.00044	60.00	228.00	1.0000	45.00	45.00	0.0018 G

12	701099	chr12:571360	571360	G	C	0.00039	0.99961	0.00039	60.00	228.00	1.0000	38.00	38.00	0.0000	G
12	701168	chr12:571429	571429	C	A	0.00025	0.99975	0.00025	60.00	59.00	1.0000	13.00	13.00	0.0105	C
12	701194	chr12:571455	571455	G	A	0.00025	0.99975	0.00025	60.00	228.00	1.0000	32.00	32.00	0.0015	G
12	701215	chr12:571476	571476	G	A	0.00212	0.99788	0.00212	60.00	228.00	1.0000	49.84	67.24	0.0003	G
12	701221	chr12:571482	571482	C	T	0.00025	0.99975	0.00025	60.00	228.00	1.0000	40.00	40.00	0.0005	C
12	701243	chr12:571504	571504	T	C	0.00056	0.99944	0.00056	60.00	228.00	1.0000	45.01	51.41	0.0002	T
12	701250	chr12:571511	571511	T	C	0.00039	0.99961	0.00039	60.00	228.00	1.0000	35.00	35.00	0.0000	T
12	701313	chr12:571574	571574	A	G	0.06254	0.93746	0.06254	60.00	226.02	0.3213	32.38	82.39	0.0005	G
12	701357	chr12:571618	571618	G	A	0.00025	0.99975	0.00025	60.00	228.00	1.0000	68.00	68.00	0.0010	G
12	701380	chr12:571641	571641	C	A	0.06233	0.93767	0.06233	60.00	225.46	0.3097	27.43	79.18	0.0007	C
12	701423	chr12:571684	571684	A	C	0.06298	0.93702	0.06298	60.00	226.71	0.3213	37.11	86.07	0.0002	C
12	701454	chr12:571715	571715	C	T	0.00025	0.99975	0.00025	60.00	228.00	1.0000	88.00	88.00	0.0000	C
12	701477	chr12:571738	571738	G	T	0.00025	0.99975	0.00025	60.00	228.00	1.0000	60.00	60.00	0.0000	G
12	701484	chr12:571745	571745	C	T	0.00116	0.99884	0.00116	60.00	228.00	1.0000	65.25	70.95	0.0000	C
12	701496	chr12:571757	571757	T	G	0.00192	0.99808	0.00192	60.00	228.00	1.0000	47.88	77.20	0.0000	T
12	701517	chr12:571778	571778	C	A	0.05981	0.94019	0.05981	60.00	225.14	0.2799	35.94	76.61	0.0038	A
12	701558	chr12:571819	571819	C	T	0.00088	0.99912	0.00088	60.00	228.00	1.0000	48.20	55.80	0.0000	c
12	701592	chr12:571853	571853	A	C	0.00324	0.99676	0.00324	59.19	211.72	1.0000	14.57	23.25	0.0908	C
12	701937	chr12:572198	572198	G	A	0.00025	0.99975	0.00025	60.00	226.00	1.0000	21.00	21.00	0.0010	G
12	702014	chr12:572275	572275	T	C	0.00025	0.99975	0.00025	60.00	228.00	1.0000	40.00	40.00	0.0005	T
12	702125	chr12:572386	572386	T	C	0.00044	0.99956	0.00044	60.00	228.00	1.0000	25.00	25.00	0.0000	T
12	702130	chr12:572391	572391	G	A	0.00025	0.99975	0.00025	60.00	228.00	1.0000	39.00	39.00	0.0020	G
12	702250	chr12:572511	572511	T	C	0.00039	0.99961	0.00039	60.00	228.00	1.0000	25.00	25.00	0.0201	T
12	702356	chr12:572617	572617	G	A	0.00075	0.99925	0.00075	60.00	228.00	1.0000	65.35	72.95	0.0000	G
12	702435	chr12:572696	572696	G	C	0.00044	0.99956	0.00044	60.00	228.00	1.0000	38.00	38.00	0.0000	G
12	702460	chr12:572721	572721	A	G	0.02100	0.97900	0.02100	60.00	228.03	0.2987	33.62	80.24	0.0005	A
12	702481	chr12:572742	572742	T	G	0.00096	0.99904	0.00096	60.00	228.00	1.0000	50.22	67.04	0.0006	T
12	702565	chr12:572826	572826	G	A	0.00025	0.99975	0.00025	60.00	228.00	1.0000	24.00	24.00	0.0040	G
12	702573	chr12:572834	572834	A	G	0.00044	0.99956	0.00044	60.00	69.00	1.0000	10.00	10.00	0.0062	A
12	702986	chr12:573247	573247	A	C	0.00062	0.99938	0.00062	59.50	201.75	1.0000	20.44	21.98	0.0205	a
12	702989	chr12:573250	573250	C	A	0.00048	0.99952	0.00048	59.68	217.29	1.0000	19.04	23.32	0.0098	C
12	703007	chr12:573268	573268	C	T	0.00025	0.99975	0.00025	60.00	228.00	1.0000	37.00	37.00	0.0005	C
12	703015	chr12:573276	573276	C	T	0.00030	0.99970	0.00030	60.00	228.00	1.0000	39.43	39.43	0.0006	C
12	703054	chr12:573315	573315	A	C	0.00025	0.99975	0.00025	60.00	228.00	1.0000	39.00	39.00	0.0005	A

12	703184	chr12:573445	573445	C	T	0.00025	0.99975	0.00025	60.00	228.00	1.0000	59.00	59.00	0.0005	C
12	703258	chr12:573519	573519	G	A	0.06189	0.93811	0.06189	60.00	225.73	0.3097	31.33	75.08	0.0009	G
12	703280	chr12:573541	573541	T	G	0.00044	0.99956	0.00044	60.00	228.00	1.0000	42.00	42.00	0.0000	T
12	703294	chr12:573555	573555	A	G	0.06168	0.93832	0.06168	60.00	224.03	0.4984	26.04	63.94	0.0029	A
12	703350	chr12:573611	573611	T	C	0.00096	0.99904	0.00096	59.50	201.72	1.0000	19.01	35.20	0.0026	T
12	703406	chr12:573667	573667	A	C	0.00050	0.99950	0.00050	59.00	228.00	1.0000	39.28	49.73	0.0000	A
12	703428	chr12:573689	573689	C	G	0.00025	0.99975	0.00025	59.00	228.00	1.0000	33.00	33.00	0.0000	C
12	703441	chr12:573702	573702	A	C	0.04507	0.95493	0.04507	59.41	198.47	0.0294	13.59	40.08	0.0160	C
12	703453	chr12:573714	573714	G	A	0.00025	0.99975	0.00025	59.00	228.00	1.0000	52.00	52.00	0.0000	G
12	703495	chr12:573756	573756	A	C	0.00039	0.99961	0.00039	60.00	221.00	1.0000	52.00	52.00	0.0039	A
12	703841	chr12:574102	574102	C	G	0.73766	0.26234	0.26234	59.56	139.89	0.2551	11.11	43.49	0.0631	G
12	703871	chr12:574132	574132	T	C	0.06277	0.93723	0.06277	58.49	222.27	0.3213	20.40	58.55	0.0005	C
12	703926	chr12:574187	574187	T	C	0.00032	0.99968	0.00032	58.64	81.02	1.0000	10.00	10.00	0.0006	T
12	704151	chr12:574412	574412	C	G	0.00025	0.99975	0.00025	60.00	228.00	1.0000	23.00	23.00	0.0015	C
12	704338	chr12:574599	574599	C	G	0.00273	0.99727	0.00273	59.50	222.67	1.0000	20.25	26.00	0.0292	c
12	704343	chr12:574604	574604	C	T	0.21370	0.78630	0.21370	59.42	208.69	0.4085	13.12	41.57	0.0221	C
12	704362	chr12:574623	574623	T	A	0.00025	0.99975	0.00025	60.00	228.00	1.0000	19.00	19.00	0.0005	T
12	704378	chr12:574639	574639	A	G	0.00039	0.99961	0.00039	59.00	228.00	1.0000	32.00	32.00	0.0008	A
12	704441	chr12:574702	574702	C	T	0.00044	0.99956	0.00044	60.00	228.00	1.0000	30.00	30.00	0.0000	C
12	704442	chr12:574703	574703	G	A	0.00025	0.99975	0.00025	60.00	228.00	1.0000	66.00	66.00	0.0000	G
12	704500	chr12:574761	574761	A	G	0.00025	0.99975	0.00025	60.00	62.00	1.0000	14.00	14.00	0.0050	A
12	704583	chr12:574844	574844	C	T	0.00025	0.99975	0.00025	60.00	228.00	1.0000	30.00	30.00	0.0125	C
12	704601	chr12:574862	574862	T	C	0.00040	0.99960	0.00040	60.00	228.00	1.0000	15.00	15.00	0.0386	T
12	704612	chr12:574873	574873	T	C	0.00026	0.99974	0.00026	59.00	228.00	1.0000	26.00	26.00	0.0409	T
12	704964	chr12:575225	575225	A	T	0.00040	0.99960	0.00040	60.00	228.00	1.0000	17.00	17.00	0.0324	A
12	704970	chr12:575231	575231	A	C	0.00025	0.99975	0.00025	60.00	228.00	1.0000	31.00	31.00	0.0200	A
12	705011	chr12:575272	575272	C	T	0.00025	0.99975	0.00025	60.00	228.00	1.0000	26.00	26.00	0.0005	C
12	705068	chr12:575329	575329	A	T	0.00102	0.99898	0.00102	60.00	224.42	1.0000	38.49	63.25	0.0005	A
12	705074	chr12:575335	575335	G	T	0.00025	0.99975	0.00025	60.00	228.00	1.0000	33.00	33.00	0.0010	G
12	705079	chr12:575340	575340	T	A	0.00044	0.99956	0.00044	60.00	228.00	1.0000	39.00	39.00	0.0000	T
12	705084	chr12:575345	575345	A	G	0.00025	0.99975	0.00025	60.00	228.00	1.0000	26.00	26.00	0.0010	A
12	705085	chr12:575346	575346	T	C	0.00039	0.99961	0.00039	60.00	228.00	1.0000	43.00	43.00	0.0000	t
12	705087	chr12:575348	575348	T	C	0.00025	0.99975	0.00025	60.00	228.00	1.0000	41.00	41.00	0.0010	T
12	705109	chr12:575370	575370	G	A	0.00260	0.99740	0.00260	60.00	226.75	1.0000	24.89	48.03	0.0002	G

12	705123	chr12:575384	575384	G	T	0.00039	0.99961	0.00039	60.00	228.00	1.0000	42.00	42.00	0.0000	G
12	705126	chr12:575387	575387	T	C	0.00025	0.99975	0.00025	60.00	228.00	1.0000	46.00	46.00	0.0015	T
12	705165	chr12:575426	575426	A	G	0.00046	0.99954	0.00046	59.00	228.00	1.0000	16.00	16.00	0.0415	A
12	705263	chr12:575524	575524	G	A	0.00025	0.99975	0.00025	59.00	228.00	1.0000	35.00	35.00	0.0015	G
12	705338	chr12:575599	575599	A	T	0.06222	0.93778	0.06222	60.00	224.26	0.3097	22.26	61.32	0.0025	A
12	705364	chr12:575625	575625	T	C	0.00260	0.99740	0.00260	58.91	223.69	1.0000	24.85	45.41	0.0032	T
12	705383	chr12:575644	575644	G	A	0.00039	0.99961	0.00039	59.00	211.00	1.0000	23.00	23.00	0.0008	G
12	705516	chr12:575777	575777	C	T	0.00025	0.99975	0.00025	60.00	228.00	1.0000	45.00	45.00	0.0010	C
12	705538	chr12:575799	575799	G	A	0.00025	0.99975	0.00025	60.00	228.00	1.0000	32.00	32.00	0.0005	G
12	705546	chr12:575807	575807	G	A	0.00039	0.99961	0.00039	60.00	228.00	1.0000	53.00	53.00	0.0000	G
12	705557	chr12:575818	575818	G	A	0.00032	0.99968	0.00032	60.00	228.00	1.0000	50.06	50.06	0.0000	G
12	705571	chr12:575832	575832	T	G	0.00030	0.99970	0.00030	60.00	228.00	1.0000	41.68	41.68	0.0000	T
12	705574	chr12:575835	575835	A	C	0.00044	0.99956	0.00044	60.00	228.00	1.0000	33.00	33.00	0.0000	A
12	705588	chr12:575849	575849	T	C	0.00025	0.99975	0.00025	60.00	228.00	1.0000	66.00	66.00	0.0000	T
12	705615	chr12:575876	575876	C	T	0.00044	0.99956	0.00044	60.00	179.00	1.0000	19.00	19.00	0.0000	C
12	705627	chr12:575888	575888	T	C	0.00030	0.99970	0.00030	60.00	61.57	1.0000	49.53	49.53	0.0006	T
12	705643	chr12:575904	575904	A	C	0.00068	0.99932	0.00068	60.00	228.00	1.0000	39.29	47.80	0.0000	A
12	705669	chr12:575930	575930	C	T	0.00044	0.99956	0.00044	60.00	228.00	1.0000	40.00	40.00	0.0000	C
12	705680	chr12:575941	575941	A	G	0.06266	0.93734	0.06266	60.00	226.50	0.3213	37.80	75.86	0.0005	G
12	705734	chr12:575995	575995	C	G	0.79164	0.20836	0.20836	60.00	179.65	0.5543	26.00	57.00	0.0859	C
12	705738	chr12:575999	575999	C	G	0.00025	0.99975	0.00025	60.00	228.00	1.0000	43.00	43.00	0.0000	C
12	705787	chr12:576048	576048	C	T	0.05786	0.94214	0.05786	60.00	220.55	0.4767	15.91	43.94	0.0092	C
12	706150	chr12:576411	576411	G	A	0.05935	0.94065	0.05935	60.00	182.91	0.3175	11.45	36.04	0.0243	G
12	706158	chr12:576419	576419	T	G	0.00769	0.99231	0.00769	59.98	225.33	1.0000	19.01	51.24	0.0020	T
12	706229	chr12:576490	576490	A	G	0.00587	0.99413	0.00587	60.00	227.48	1.0000	24.71	73.31	0.0005	A
12	706302	chr12:576563	576563	G	A	0.00039	0.99961	0.00039	60.00	228.00	1.0000	51.00	51.00	0.0000	G
12	706321	chr12:576582	576582	A	G	0.06275	0.93725	0.06275	60.00	227.02	0.3213	41.11	83.10	0.0002	A
12	706336	chr12:576597	576597	G	A	0.00025	0.99975	0.00025	60.00	228.00	1.0000	52.00	52.00	0.0000	G
12	706378	chr12:576639	576639	C	G	0.00137	0.99863	0.00137	60.00	74.94	1.0000	22.16	37.02	0.0092	C
12	706393	chr12:576654	576654	C	T	0.00025	0.99975	0.00025	60.00	228.00	1.0000	59.00	59.00	0.0000	C
12	706438	chr12:576699	576699	T	C	0.00039	0.99961	0.00039	60.00	228.00	1.0000	46.00	46.00	0.0000	T
12	706444	chr12:576705	576705	T	C	0.00056	0.99944	0.00056	60.00	228.00	1.0000	66.03	71.09	0.0000	T
12	706496	chr12:576757	576757	C	T	0.00025	0.99975	0.00025	60.00	228.00	1.0000	46.00	46.00	0.0005	C
12	706523	chr12:576784	576784	G	A	0.00175	0.99825	0.00175	60.00	228.00	1.0000	40.99	65.76	0.0003	G

12	706557	chr12:576818	576818	C	T	0.00025	0.99975	0.00025	60.00	228.00	1.0000	62.00	62.00	0.0010	c
12	706568	chr12:576829	576829	A	G	0.00045	0.99955	0.00045	60.00	228.00	1.0000	50.49	53.92	0.0005	A
12	706585	chr12:576846	576846	T	G	0.00032	0.99968	0.00032	60.00	228.00	1.0000	48.88	48.88	0.0025	T
12	706610	chr12:576871	576871	G	A	0.00044	0.99956	0.00044	60.00	228.00	1.0000	47.00	47.00	0.0000	G
12	706654	chr12:576915	576915	A	G	0.00181	0.99819	0.00181	60.00	221.88	1.0000	23.55	43.28	0.0027	A
12	706743	chr12:577004	577004	C	G	0.00044	0.99956	0.00044	60.00	228.00	1.0000	22.00	22.00	0.0018	C
12	706744	chr12:577005	577005	A	T	0.00039	0.99961	0.00039	60.00	228.00	1.0000	22.00	22.00	0.0000	A
12	706779	chr12:577040	577040	T	A	0.00077	0.99923	0.00077	60.00	228.00	1.0000	32.33	44.68	0.0000	T
12	706802	chr12:577063	577063	C	A	0.00025	0.99975	0.00025	60.00	228.00	1.0000	41.00	41.00	0.0050	C
12	706814	chr12:577075	577075	A	G	0.00041	0.99959	0.00041	60.00	65.36	1.0000	20.46	20.46	0.0033	A
12	706823	chr12:577084	577084	C	G	0.00025	0.99975	0.00025	60.00	31.00	1.0000	19.00	19.00	0.0090	C
12	706877	chr12:577138	577138	T	C	0.00057	0.99943	0.00057	60.00	228.00	1.0000	47.73	50.10	0.0016	T
12	706933	chr12:577194	577194	A	G	0.06171	0.93829	0.06171	60.00	223.77	0.2989	18.25	59.91	0.0034	A
12	706951	chr12:577212	577212	C	T	0.00025	0.99975	0.00025	60.00	228.00	1.0000	29.00	29.00	0.0090	C
12	706979	chr12:577240	577240	C	G	0.00026	0.99974	0.00026	60.00	228.00	1.0000	23.00	23.00	0.0275	C
12	707023	chr12:577284	577284	T	C	0.00079	0.99921	0.00079	60.00	228.00	1.0000	31.16	46.03	0.0014	T
12	707025	chr12:577286	577286	T	C	0.00041	0.99959	0.00041	60.00	228.00	1.0000	56.86	56.86	0.0000	T
12	707039	chr12:577300	577300	C	A	0.00044	0.99956	0.00044	60.00	228.00	1.0000	32.00	32.00	0.0009	C
12	707066	chr12:577327	577327	C	T	0.00025	0.99975	0.00025	60.00	84.00	1.0000	15.00	15.00	0.0025	C
12	707072	chr12:577333	577333	C	T	0.00050	0.99950	0.00050	60.00	255.00	0.0005	92.00	92.00	0.0030	C
12	707130	chr12:577391	577391	T	A	0.00039	0.99961	0.00039	60.00	228.00	1.0000	48.00	48.00	0.0000	T
12	707158	chr12:577419	577419	T	C	0.00039	0.99961	0.00039	60.00	228.00	1.0000	61.00	61.00	0.0000	T
12	707181	chr12:577442	577442	G	A	0.00039	0.99961	0.00039	60.00	228.00	1.0000	44.00	44.00	0.0000	G
12	707190	chr12:577451	577451	C	T	0.00030	0.99970	0.00030	60.00	228.00	1.0000	58.10	58.10	0.0006	C
12	707202	chr12:577463	577463	G	C	0.00025	0.99975	0.00025	60.00	228.00	1.0000	60.00	60.00	0.0010	G
12	707209	chr12:577470	577470	C	T	0.00077	0.99923	0.00077	60.00	228.00	1.0000	60.00	60.00	0.0000	C
12	707210	chr12:577471	577471	G	A	0.28360	0.71640	0.28360	60.00	220.14	0.3865	28.87	76.08	0.0025	g
12	707273	chr12:577534	577534	G	A	0.00044	0.99956	0.00044	60.00	228.00	1.0000	65.00	65.00	0.0000	G
12	707294	chr12:577555	577555	C	A	0.00044	0.99956	0.00044	60.00	228.00	1.0000	86.00	86.00	0.0000	C
12	707323	chr12:577584	577584	C	T	0.00025	0.99975	0.00025	60.00	228.00	1.0000	69.00	69.00	0.0005	C
12	707324	chr12:577585	577585	G	A	0.00124	0.99876	0.00124	60.00	228.00	1.0000	39.17	49.52	0.0000	G
12	707332	chr12:577593	577593	C	G	0.00025	0.99975	0.00025	59.00	188.00	1.0000	33.00	33.00	0.0035	C
12	707351	chr12:577612	577612	G	C	0.00025	0.99975	0.00025	60.00	80.00	1.0000	17.00	17.00	0.0005	G
12	707377	chr12:577638	577638	C	T	0.02507	0.97493	0.02507	60.00	227.04	1.0000	30.64	76.39	0.0007	C

12	707433	chr12:577694	577694	C	A	0.00039	0.99961	0.00039	60.00	228.00	1.0000	60.00	60.00	0.0000 T
12	707535	chr12:577796	577796	C	T	0.00054	0.99946	0.00054	60.00	69.50	1.0000	15.00	15.00	0.0784 C
12	707551	chr12:577812	577812	G	C	0.00044	0.99956	0.00044	60.00	228.00	1.0000	27.00	27.00	0.0062 G
12	707575	chr12:577836	577836	G	C	0.27528	0.72472	0.27528	60.00	186.10	0.2066	10.43	43.54	0.0495 G
12	707660	chr12:577921	577921	C	T	0.25770	0.74230	0.25770	60.00	192.24	0.3994	14.18	55.30	0.0185 C
12	707690	chr12:577951	577951	C	T	0.00032	0.99968	0.00032	60.00	228.00	1.0000	58.92	58.92	0.0010 C
12	707712	chr12:577973	577973	G	A	0.00666	0.99334	0.00666	60.00	225.40	0.0863	29.35	68.16	0.0007 G
12	707717	chr12:577978	577978	A	T	0.00077	0.99923	0.00077	60.00	228.00	1.0000	62.08	64.93	0.0000 A
12	707747	chr12:578008	578008	C	G	0.00082	0.99918	0.00082	60.00	228.00	1.0000	56.11	60.67	0.0000 C
12	707761	chr12:578022	578022	T	G	0.00044	0.99956	0.00044	60.00	228.00	1.0000	33.00	33.00	0.0000 T
12	707782	chr12:578043	578043	C	T	0.00044	0.99956	0.00044	60.00	228.00	1.0000	35.00	35.00	0.0000 C
12	707785	chr12:578046	578046	A	C	0.00025	0.99975	0.00025	60.00	135.00	1.0000	35.00	35.00	0.0105 A
12	708243	chr12:578504	578504	G	A	0.00167	0.99833	0.00167	60.00	223.65	1.0000	25.02	60.35	0.0006 G
12	708278	chr12:578539	578539	T	C	0.00136	0.99864	0.00136	59.78	227.25	1.0000	25.59	32.56	0.0007 T
12	708285	chr12:578546	578546	T	A	0.45627	0.54373	0.45627	59.65	176.88	0.0001	12.64	32.08	0.0804 T
12	708513	chr12:578774	578774	G	A	0.00163	0.99837	0.00163	59.50	81.00	0.0010	14.18	20.83	0.0532 G
12	708597	chr12:578858	578858	G	A	0.48322	0.51678	0.48322	60.00	212.82	0.4763	30.82	74.28	0.0050 A
12	708600	chr12:578861	578861	G	A	0.00039	0.99961	0.00039	60.00	228.00	1.0000	37.00	37.00	0.0000 G
12	708604	chr12:578865	578865	C	A	0.00025	0.99975	0.00025	60.00	228.00	1.0000	51.00	51.00	0.0005 C
12	708626	chr12:578887	578887	G	A	0.78078	0.21922	0.21922	60.00	209.51	0.0058	32.37	80.32	0.0029 G
12	708633	chr12:578894	578894	G	A	0.00025	0.99975	0.00025	60.00	228.00	1.0000	39.00	39.00	0.0000 G
12	708696	chr12:578957	578957	A	G	0.48417	0.51583	0.48417	60.00	216.83	0.2160	32.65	82.01	0.0090 A
12	708703	chr12:578964	578964	C	T	0.00039	0.99961	0.00039	60.00	228.00	1.0000	57.00	57.00	0.0000 C
12	708716	chr12:578977	578977	C	T	0.00044	0.99956	0.00044	60.00	228.00	1.0000	38.00	38.00	0.0000 C
12	708725	chr12:578986	578986	C	T	0.00025	0.99975	0.00025	60.00	228.00	1.0000	37.00	37.00	0.0005 C
12	708786	chr12:579047	579047	A	T	0.00039	0.99961	0.00039	60.00	228.00	1.0000	50.00	50.00	0.0000 A
12	708792	chr12:579053	579053	C	G	0.05421	0.94579	0.05421	60.00	226.52	0.4767	29.81	80.52	0.0009 C
12	708800	chr12:579061	579061	C	G	0.00497	0.99503	0.00497	60.00	228.00	1.0000	38.23	72.15	0.0007 C
12	708853	chr12:579114	579114	T	C	0.00044	0.99956	0.00044	60.00	228.00	1.0000	40.00	40.00	0.0000 T
12	708862	chr12:579123	579123	T	A	0.00044	0.99956	0.00044	60.00	228.00	1.0000	66.00	66.00	0.0000 T
12	708907	chr12:579168	579168	C	A	0.00026	0.99974	0.00026	60.00	172.00	1.0000	15.00	15.00	0.0285 C
12	708916	chr12:579177	579177	G	A	0.00026	0.99974	0.00026	60.00	228.00	1.0000	23.00	23.00	0.0295 G
12	708922	chr12:579183	579183	G	A	0.00132	0.99868	0.00132	60.00	211.83	1.0000	14.24	41.93	0.0322 G
12	708931	chr12:579192	579192	A	T	0.00093	0.99907	0.00093	60.00	94.50	1.0000	13.10	16.90	0.0486 A

12	708946	chr12:579207	579207	C	T	0.00045	0.99955	0.00045	60.00	228.00	1.0000	18.00	18.00	0.0274	C
12	708974	chr12:579235	579235	G	A	0.00042	0.99958	0.00042	60.00	35.00	1.0000	10.00	10.00	0.0864	G
12	708978	chr12:579239	579239	G	A	0.00053	0.99947	0.00053	60.00	222.50	1.0000	15.30	26.70	0.0609	G
12	709007	chr12:579268	579268	G	A	0.00126	0.99874	0.00126	60.00	227.75	1.0000	14.84	27.63	0.1018	G
12	709049	chr12:579310	579310	G	A	0.00026	0.99974	0.00026	60.00	228.00	1.0000	25.00	25.00	0.0424	G
12	709064	chr12:579325	579325	C	T	0.00057	0.99943	0.00057	60.00	224.64	1.0000	17.25	22.33	0.0179	C
12	709098	chr12:579359	579359	A	C	0.46982	0.53018	0.46982	60.00	185.14	0.2084	12.49	54.83	0.0496	C
12	709107	chr12:579368	579368	G	A	0.00026	0.99974	0.00026	60.00	228.00	1.0000	34.00	34.00	0.0270	G
12	709141	chr12:579402	579402	G	A	0.00025	0.99975	0.00025	60.00	203.00	1.0000	51.00	51.00	0.0115	G
12	709145	chr12:579406	579406	C	T	0.00600	0.99400	0.00600	60.00	221.83	1.0000	19.66	54.14	0.0040	C
12	709207	chr12:579468	579468	C	T	0.00044	0.99956	0.00044	60.00	228.00	1.0000	41.00	41.00	0.0000	C
12	709241	chr12:579502	579502	G	A	0.00237	0.99763	0.00237	60.00	228.72	1.0000	39.70	69.71	0.0000	g
12	709262	chr12:579523	579523	G	T	0.00535	0.99465	0.00535	60.00	228.00	1.0000	60.04	84.56	0.0000	G
12	709270	chr12:579531	579531	T	A	0.00039	0.99961	0.00039	60.00	228.00	1.0000	73.00	73.00	0.0000	T
12	709291	chr12:579552	579552	C	T	0.00044	0.99956	0.00044	60.00	228.00	1.0000	55.00	55.00	0.0000	C
12	709304	chr12:579565	579565	C	T	0.00044	0.99956	0.00044	60.00	228.00	1.0000	58.00	58.00	0.0000	C
12	709333	chr12:579594	579594	G	A	0.00050	0.99950	0.00050	60.00	228.00	1.0000	74.20	81.80	0.0000	G
12	709399	chr12:579660	579660	C	T	0.00039	0.99961	0.00039	60.00	228.00	1.0000	45.00	45.00	0.0000	C
12	709415	chr12:579676	579676	A	C	0.00025	0.99975	0.00025	60.00	228.00	1.0000	50.00	50.00	0.0000	A
12	709425	chr12:579686	579686	C	T	0.00025	0.99975	0.00025	60.00	228.00	1.0000	47.00	47.00	0.0005	C
12	709445	chr12:579706	579706	T	G	0.01809	0.98191	0.01809	59.87	216.10	0.0014	14.86	36.87	0.0508	T
12	709787	chr12:580048	580048	G	A	0.00044	0.99956	0.00044	60.00	228.00	1.0000	52.00	52.00	0.0000	G
12	709799	chr12:580060	580060	G	A	0.00030	0.99970	0.00030	60.00	228.00	1.0000	45.32	45.32	0.0000	G
12	709819	chr12:580080	580080	G	T	0.00044	0.99956	0.00044	59.00	228.00	1.0000	37.00	37.00	0.0000	g
12	709821	chr12:580082	580082	T	G	0.00124	0.99876	0.00124	59.40	216.06	1.0000	25.86	33.19	0.0014	T
12	709834	chr12:580095	580095	G	A	0.00045	0.99955	0.00045	59.00	46.00	1.0000	10.00	10.00	0.0097	G
12	709838	chr12:580099	580099	G	A	0.00042	0.99958	0.00042	59.53	42.13	1.0000	10.00	10.00	0.0165	g
12	709941	chr12:580202	580202	G	A	0.00090	0.99910	0.00090	57.89	187.94	1.0000	15.24	31.16	0.0018	G
12	710075	chr12:580336	580336	A	T	0.29756	0.70244	0.29756	60.00	228.28	0.0911	46.47	86.56	0.0011	A
12	710085	chr12:580346	580346	A	G	0.00231	0.99769	0.00231	60.00	228.00	1.0000	30.38	64.00	0.0000	A
12	710096	chr12:580357	580357	T	C	0.00044	0.99956	0.00044	60.00	68.00	1.0000	40.00	40.00	0.0018	C
12	710110	chr12:580371	580371	C	T	0.00044	0.99956	0.00044	60.00	228.00	1.0000	87.00	87.00	0.0000	C
12	710281	chr12:580542	580542	T	C	0.00039	0.99961	0.00039	60.00	228.00	1.0000	74.00	74.00	0.0000	T
12	710361	chr12:580622	580622	G	A	0.00722	0.99278	0.00722	60.00	221.64	1.0000	32.21	56.79	0.0000	G

12	710369	chr12:580630	580630	T	A	0.00025	0.99975	0.00025	60.00	228.00	1.0000	35.00	35.00	0.0000	T
12	710395	chr12:580656	580656	T	A	0.00026	0.99974	0.00026	60.00	38.00	1.0000	14.00	14.00	0.0574	T
12	711057	chr12:581318	581318	A	C	0.00031	0.99969	0.00031	60.00	39.00	1.0000	10.00	10.00	0.1837	A
12	711070	chr12:581331	581331	G	T	0.00119	0.99881	0.00119	59.87	110.22	1.0000	11.39	18.93	0.0587	G
12	711078	chr12:581339	581339	T	C	0.00032	0.99968	0.00032	60.00	228.00	1.0000	30.72	30.72	0.0003	T
12	711135	chr12:581396	581396	C	T	0.02020	0.97980	0.02020	60.00	227.62	0.1009	32.82	79.88	0.0002	C
12	711149	chr12:581410	581410	G	C	0.00044	0.99956	0.00044	60.00	228.00	1.0000	65.00	65.00	0.0000	G
12	711234	chr12:581495	581495	C	T	0.00025	0.99975	0.00025	60.00	228.00	1.0000	80.00	80.00	0.0000	C
12	711272	chr12:581533	581533	C	G	0.00044	0.99956	0.00044	60.00	228.00	1.0000	85.00	85.00	0.0000	C
12	711295	chr12:581556	581556	G	A	0.00039	0.99961	0.00039	60.00	228.00	1.0000	61.00	61.00	0.0000	G
12	711463	chr12:581724	581724	C	G	0.00077	0.99923	0.00077	60.00	228.00	1.0000	48.15	53.85	0.0000	C
12	711496	chr12:581757	581757	T	C	0.59485	0.40515	0.40515	59.53	208.41	0.0250	30.71	78.28	0.0043	C
12	711501	chr12:581762	581762	A	G	0.00025	0.99975	0.00025	60.00	228.00	1.0000	72.00	72.00	0.0005	A
12	711560	chr12:581821	581821	T	C	0.00039	0.99961	0.00039	60.00	228.00	1.0000	86.00	86.00	0.0000	T
12	711572	chr12:581833	581833	T	C	0.00025	0.99975	0.00025	60.00	228.00	1.0000	73.00	73.00	0.0000	t
12	711631	chr12:581892	581892	C	T	0.00532	0.99468	0.00532	58.68	216.83	1.0000	30.68	64.90	0.0027	C
12	711646	chr12:581907	581907	T	C	0.59514	0.40486	0.40486	59.44	212.38	0.0234	34.03	80.81	0.0025	T
12	711662	chr12:581923	581923	T	C	0.59625	0.40375	0.40375	60.00	220.23	0.0177	38.81	87.24	0.0023	t
12	711671	chr12:581932	581932	C	G	0.00044	0.99956	0.00044	60.00	228.00	1.0000	58.00	58.00	0.0000	C
12	711682	chr12:581943	581943	T	C	0.00025	0.99975	0.00025	60.00	228.00	1.0000	71.00	71.00	0.0000	T
12	711707	chr12:581968	581968	G	T	0.00032	0.99968	0.00032	60.00	228.00	1.0000	58.06	58.06	0.0000	G
12	711753	chr12:582014	582014	A	G	0.00025	0.99975	0.00025	60.00	228.00	1.0000	84.00	84.00	0.0000	A
12	711768	chr12:582029	582029	G	A	0.00044	0.99956	0.00044	60.00	228.00	1.0000	80.00	80.00	0.0000	G
12	711811	chr12:582072	582072	G	A	0.19243	0.80757	0.19243	60.00	227.68	0.1801	43.86	91.37	0.0014	G
12	711819	chr12:582080	582080	G	C	0.00044	0.99956	0.00044	60.00	228.00	1.0000	39.00	39.00	0.0000	G
12	711827	chr12:582088	582088	C	T	0.07151	0.92849	0.07151	59.92	225.09	0.5813	32.27	70.14	0.0011	C
12	711842	chr12:582103	582103	C	T	0.00039	0.99961	0.00039	60.00	228.00	1.0000	43.00	43.00	0.0000	C
12	711891	chr12:582152	582152	A	T	0.83389	0.16611	0.16611	59.92	181.15	0.0302	23.63	61.44	0.0054	T
12	711896	chr12:582157	582157	G	A	0.00025	0.99975	0.00025	60.00	228.00	1.0000	68.00	68.00	0.0000	G
12	711916	chr12:582177	582177	T	A	0.00025	0.99975	0.00025	60.00	228.00	1.0000	57.00	57.00	0.0000	T
12	712019	chr12:582280	582280	G	C	0.00045	0.99955	0.00045	59.00	228.00	1.0000	45.00	45.00	0.0150	G
12	712025	chr12:582286	582286	G	T	0.00046	0.99954	0.00046	59.00	228.00	1.0000	21.00	21.00	0.0389	G
12	712123	chr12:582384	582384	T	A	0.00039	0.99961	0.00039	60.00	89.00	1.0000	32.00	32.00	0.0000	T
12	712129	chr12:582390	582390	G	T	0.00077	0.99923	0.00077	60.00	228.00	1.0000	43.13	47.88	0.0000	G

12	712143	chr12:582404	582404	C	A	0.00077	0.99923	0.00077	60.00	228.00	1.0000	37.03	37.98	0.0000	C
12	712168	chr12:582429	582429	T	G	0.00052	0.99948	0.00052	60.00	123.00	1.0000	10.00	10.00	0.1484	T
12	712265	chr12:582526	582526	A	G	0.00025	0.99975	0.00025	59.00	228.00	1.0000	40.00	40.00	0.0030	A
12	712296	chr12:582557	582557	C	T	0.00025	0.99975	0.00025	59.00	228.00	1.0000	33.00	33.00	0.0000	C
12	712310	chr12:582571	582571	G	C	0.00032	0.99968	0.00032	60.00	228.00	1.0000	63.72	63.72	0.0000	G
12	712311	chr12:582572	582572	G	A	0.00025	0.99975	0.00025	60.00	228.00	1.0000	68.00	68.00	0.0000	G
12	712341	chr12:582602	582602	T	C	0.00039	0.99961	0.00039	60.00	228.00	1.0000	26.00	26.00	0.0000	T
12	712354	chr12:582615	582615	C	A	0.00044	0.99956	0.00044	59.00	216.00	1.0000	14.00	14.00	0.0000	C
12	712385	chr12:582646	582646	T	A	0.00248	0.99752	0.00248	58.32	187.59	1.0000	12.63	23.23	0.0005	T
12	712453	chr12:582714	582714	G	T	0.00025	0.99975	0.00025	60.00	228.00	1.0000	55.00	55.00	0.0000	G
12	712500	chr12:582761	582761	A	G	0.00050	0.99950	0.00050	59.00	228.00	1.0000	34.33	46.68	0.0000	A
12	712517	chr12:582778	582778	C	T	0.65257	0.34743	0.34743	59.28	186.04	0.0001	25.74	74.70	0.0364	T
12	712523	chr12:582784	582784	C	A	0.00025	0.99975	0.00025	60.00	228.00	1.0000	43.00	43.00	0.0000	C
12	712619	chr12:582880	582880	A	T	0.18176	0.81824	0.18176	60.00	228.88	0.0196	47.09	94.55	0.0005	T
12	712701	chr12:582962	582962	T	G	0.00044	0.99956	0.00044	59.00	228.00	1.0000	71.00	71.00	0.0000	T
12	712704	chr12:582965	582965	T	C	0.13484	0.86516	0.13484	59.14	221.27	0.0447	25.83	60.85	0.0025	T
12	712719	chr12:582980	582980	G	A	0.00044	0.99956	0.00044	59.00	223.00	1.0000	41.00	41.00	0.0000	G
12	712746	chr12:583007	583007	T	C	0.00039	0.99961	0.00039	58.00	228.00	1.0000	49.00	49.00	0.0000	T
12	712747	chr12:583008	583008	G	A	0.00039	0.99961	0.00039	59.00	228.00	1.0000	43.00	43.00	0.0000	G
12	712761	chr12:583022	583022	G	A	0.00025	0.99975	0.00025	59.00	228.00	1.0000	42.00	42.00	0.0005	G
12	712766	chr12:583027	583027	A	G	0.00039	0.99961	0.00039	59.00	228.00	1.0000	57.00	57.00	0.0000	A
12	713153	chr12:583414	583414	G	A	0.18744	0.81256	0.18744	60.00	223.42	0.2550	33.47	75.17	0.0045	A
12	713156	chr12:583417	583417	G	A	0.07423	0.92577	0.07423	60.00	226.59	0.6169	36.83	78.00	0.0010	G
12	713246	chr12:583507	583507	C	T	0.00025	0.99975	0.00025	60.00	228.00	1.0000	86.00	86.00	0.0000	c
12	713285	chr12:583546	583546	T	C	0.35630	0.64370	0.35630	60.00	226.32	0.1201	41.02	89.84	0.0027	C
12	713308	chr12:583569	583569	G	A	0.00025	0.99975	0.00025	60.00	228.00	1.0000	55.00	55.00	0.0000	G
12	713309	chr12:583570	583570	C	G	0.00039	0.99961	0.00039	60.00	228.00	1.0000	64.00	64.00	0.0000	c
12	713392	chr12:583653	583653	C	T	0.00032	0.99968	0.00032	60.00	228.00	1.0000	53.53	53.53	0.0000	C
12	713433	chr12:583694	583694	C	A	0.00025	0.99975	0.00025	60.00	174.00	1.0000	33.00	33.00	0.0000	C
12	713436	chr12:583697	583697	C	T	0.00050	0.99950	0.00050	60.00	228.00	1.0000	45.55	66.45	0.0000	C
12	713525	chr12:583786	583786	C	T	0.00026	0.99974	0.00026	60.00	228.00	1.0000	23.00	23.00	0.0325	C
12	713532	chr12:583793	583793	G	A	0.00039	0.99961	0.00039	60.00	228.00	1.0000	21.00	21.00	0.0201	G
12	713804	chr12:584065	584065	A	G	0.00254	0.99746	0.00254	59.89	208.59	1.0000	13.45	29.57	0.0217	a
12	713907	chr12:584168	584168	G	A	0.00039	0.99961	0.00039	60.00	228.00	1.0000	53.00	53.00	0.0000	g

12	713930	chr12:584191	584191	C	T	0.00061	0.99939	0.00061	60.00	228.00	1.0000	33.44	57.64	0.0015	c
12	713948	chr12:584209	584209	C	A	0.05406	0.94594	0.05406	60.00	224.26	0.3553	19.05	57.33	0.0043	t
12	713999	chr12:584260	584260	C	T	0.00025	0.99975	0.00025	60.00	228.00	1.0000	57.00	57.00	0.0005	c
12	714084	chr12:584345	584345	C	T	0.00044	0.99956	0.00044	60.00	228.00	1.0000	69.00	69.00	0.0000	c
12	714094	chr12:584355	584355	G	A	0.00039	0.99961	0.00039	60.00	228.00	1.0000	62.00	62.00	0.0000	g
12	714095	chr12:584356	584356	T	G	0.00452	0.99548	0.00452	60.00	66.61	1.0000	14.65	38.46	0.0016	t
12	714169	chr12:584430	584430	G	A	0.00075	0.99925	0.00075	60.00	228.00	1.0000	46.15	74.65	0.0005	g
12	714224	chr12:584485	584485	A	C	0.72928	0.27072	0.27072	60.00	215.26	0.0001	32.91	87.23	0.0034	a
12	714275	chr12:584536	584536	G	A	0.00039	0.99961	0.00039	60.00	215.00	1.0000	33.00	33.00	0.0000	g
12	714281	chr12:584542	584542	G	C	0.00045	0.99955	0.00045	60.00	63.00	1.0000	41.00	41.00	0.0247	g
12	714312	chr12:584573	584573	G	T	0.00044	0.99956	0.00044	60.00	228.00	1.0000	19.00	19.00	0.0000	g
12	714348	chr12:584609	584609	C	T	0.00025	0.99975	0.00025	60.00	228.00	1.0000	49.00	49.00	0.0070	c
12	714355	chr12:584616	584616	C	T	0.00077	0.99923	0.00077	60.00	228.00	1.0000	36.23	44.78	0.0000	c
12	714416	chr12:584677	584677	G	T	0.00031	0.99969	0.00031	60.00	74.99	1.0000	23.40	23.40	0.0078	g
12	714479	chr12:584740	584740	C	A	0.00044	0.99956	0.00044	60.00	228.00	1.0000	46.00	46.00	0.0000	c
12	714569	chr12:584830	584830	G	T	0.00082	0.99918	0.00082	60.00	228.00	1.0000	40.83	41.85	0.0004	g
12	714576	chr12:584837	584837	T	G	0.13322	0.86678	0.13322	60.00	213.50	0.0647	18.02	50.00	0.0740	t
12	714592	chr12:584853	584853	C	T	0.00025	0.99975	0.00025	60.00	228.00	1.0000	32.00	32.00	0.0105	c
12	714741	chr12:585002	585002	A	G	0.00025	0.99975	0.00025	60.00	228.00	1.0000	17.00	17.00	0.0205	A
12	714792	chr12:585053	585053	C	T	0.00034	0.99966	0.00034	60.00	228.00	1.0000	49.67	49.67	0.0005	C
12	714817	chr12:585078	585078	T	C	0.00050	0.99950	0.00050	60.00	228.00	1.0000	62.15	67.85	0.0010	T
12	714842	chr12:585103	585103	A	G	0.54228	0.45772	0.45772	60.00	224.52	0.0325	38.30	86.80	0.0018	A
12	714909	chr12:585170	585170	T	A	0.00030	0.99970	0.00030	60.00	228.00	1.0000	80.75	80.75	0.0000	T
12	714910	chr12:585171	585171	C	T	0.06149	0.93851	0.06149	60.00	224.07	0.1961	41.71	85.01	0.0090	C
12	714937	chr12:585198	585198	G	A	0.00039	0.99961	0.00039	60.00	228.00	1.0000	77.00	77.00	0.0000	g
12	714980	chr12:585241	585241	G	C	0.00039	0.99961	0.00039	60.00	228.00	1.0000	65.00	65.00	0.0000	G
12	714986	chr12:585247	585247	C	T	0.00025	0.99975	0.00025	60.00	228.00	1.0000	41.00	41.00	0.0005	C
12	715113	chr12:585374	585374	G	A	0.00025	0.99975	0.00025	60.00	228.00	1.0000	31.00	31.00	0.0005	G
12	715133	chr12:585394	585394	A	G	0.00025	0.99975	0.00025	60.00	228.00	1.0000	34.00	34.00	0.0010	A
12	715164	chr12:585425	585425	A	G	0.00025	0.99975	0.00025	60.00	228.00	1.0000	24.00	24.00	0.0020	A
12	715172	chr12:585433	585433	C	T	0.00025	0.99975	0.00025	60.00	228.00	1.0000	23.00	23.00	0.0020	C
12	715364	chr12:585625	585625	C	T	0.00039	0.99961	0.00039	60.00	156.00	1.0000	11.00	11.00	0.0201	C
12	715368	chr12:585629	585629	A	G	0.79614	0.20386	0.20386	60.00	117.77	0.0013	10.00	37.03	0.1767	G
12	715452	chr12:585713	585713	G	A	0.00048	0.99952	0.00048	60.00	161.00	1.0000	19.00	19.00	0.0839	g

12	715467	chr12:585728	585728	T	C	0.00046	0.99954	0.00046	60.00	228.00	1.0000	26.00	26.00	0.0406	T
12	715572	chr12:585833	585833	T	C	0.00075	0.99925	0.00075	60.00	228.00	1.0000	20.45	36.60	0.0035	T
12	715603	chr12:585864	585864	A	T	0.00061	0.99939	0.00061	60.00	228.00	1.0000	24.30	27.70	0.0082	A
12	715628	chr12:585889	585889	T	G	0.00045	0.99955	0.00045	60.00	160.00	1.0000	10.00	10.00	0.0230	T
12	715632	chr12:585893	585893	T	C	0.00078	0.99922	0.00078	60.00	228.00	1.0000	17.03	17.98	0.0123	t
12	715653	chr12:585914	585914	T	C	0.00025	0.99975	0.00025	60.00	225.00	1.0000	30.00	30.00	0.0040	T
12	715659	chr12:585920	585920	G	A	0.00025	0.99975	0.00025	60.00	228.00	1.0000	42.00	42.00	0.0025	G
12	715726	chr12:585987	585987	T	A	0.00032	0.99968	0.00032	60.00	228.00	1.0000	43.19	43.19	0.0010	T
12	715745	chr12:586006	586006	G	A	0.00025	0.99975	0.00025	60.00	228.00	1.0000	43.00	43.00	0.0030	G
12	715803	chr12:586064	586064	G	A	0.00025	0.99975	0.00025	60.00	228.00	1.0000	46.00	46.00	0.0010	G
12	715804	chr12:586065	586065	C	T	0.00048	0.99952	0.00048	60.00	228.00	1.0000	34.67	44.39	0.0006	C
12	715834	chr12:586095	586095	C	T	0.00113	0.99887	0.00113	60.00	228.00	1.0000	47.74	69.22	0.0002	C
12	715900	chr12:586161	586161	A	C	0.00039	0.99961	0.00039	60.00	228.00	1.0000	67.00	67.00	0.0000	A
12	715942	chr12:586203	586203	G	T	0.00039	0.99961	0.00039	60.00	36.00	1.0000	30.00	30.00	0.0000	G
12	715992	chr12:586253	586253	G	A	0.00025	0.99975	0.00025	60.00	57.00	1.0000	12.00	12.00	0.0045	G
12	715995	chr12:586256	586256	C	T	0.01627	0.98373	0.01627	60.00	223.93	0.0758	21.90	51.07	0.0081	C
12	715996	chr12:586257	586257	G	A	0.00044	0.99956	0.00044	60.00	228.00	1.0000	46.00	46.00	0.0000	G
12	716027	chr12:586288	586288	G	A	0.00044	0.99956	0.00044	60.00	205.00	1.0000	37.00	37.00	0.0009	G
12	716040	chr12:586301	586301	A	G	0.00847	0.99153	0.00847	59.99	225.46	1.0000	23.47	63.93	0.0007	A
12	716050	chr12:586311	586311	A	G	0.00025	0.99975	0.00025	60.00	96.00	1.0000	24.00	24.00	0.0090	A
12	716051	chr12:586312	586312	A	G	0.50542	0.49458	0.49458	59.97	184.84	0.0319	15.25	50.26	0.0621	G
12	716070	chr12:586331	586331	C	T	0.00025	0.99975	0.00025	60.00	228.00	1.0000	42.00	42.00	0.0020	C
12	716074	chr12:586335	586335	G	A	0.07196	0.92804	0.07196	60.00	223.76	0.3179	17.10	61.12	0.0011	G
12	716142	chr12:586403	586403	G	T	0.00044	0.99956	0.00044	60.00	180.00	1.0000	17.00	17.00	0.0000	g
12	716155	chr12:586416	586416	C	G	0.00030	0.99970	0.00030	60.00	228.00	1.0000	38.68	38.68	0.0006	C
12	716157	chr12:586418	586418	G	A	0.00039	0.99961	0.00039	60.00	228.00	1.0000	75.00	75.00	0.0000	G
12	716191	chr12:586452	586452	T	C	0.00025	0.99975	0.00025	60.00	228.00	1.0000	45.00	45.00	0.0000	T
12	716288	chr12:586549	586549	T	C	0.00025	0.99975	0.00025	60.00	228.00	1.0000	50.00	50.00	0.0005	T
12	716320	chr12:586581	586581	G	C	0.00050	0.99950	0.00050	60.00	228.00	1.0000	20.58	42.43	0.0025	G
12	716325	chr12:586586	586586	A	T	0.00050	0.99950	0.00050	60.00	228.00	1.0000	28.25	37.75	0.0025	A
12	716449	chr12:586710	586710	A	G	0.00044	0.99956	0.00044	60.00	78.00	1.0000	10.00	10.00	0.0018	A
12	716453	chr12:586714	586714	A	G	0.00046	0.99954	0.00046	60.00	44.00	1.0000	13.00	13.00	0.0406	A
12	716466	chr12:586727	586727	T	G	0.00044	0.99956	0.00044	60.00	228.00	1.0000	44.00	44.00	0.0000	T
12	716509	chr12:586770	586770	T	C	0.21536	0.78464	0.21536	60.00	218.59	0.1462	22.83	59.24	0.0097	T

12	716518	chr12:586779	586779	G	A	0.00034	0.99966	0.00034	60.00	228.00	1.0000	25.59	25.59	0.0005	G
12	716596	chr12:586857	586857	G	A	0.00032	0.99968	0.00032	60.00	228.00	1.0000	35.64	35.64	0.0010	G
12	716663	chr12:586924	586924	C	T	0.00039	0.99961	0.00039	60.00	228.00	1.0000	52.00	52.00	0.0000	C
12	716664	chr12:586925	586925	G	A	0.18848	0.81152	0.18848	60.00	222.51	0.1306	25.82	73.00	0.0025	G
12	716723	chr12:586984	586984	A	T	0.00039	0.99961	0.00039	60.00	228.00	1.0000	58.00	58.00	0.0000	A
12	716773	chr12:587034	587034	T	C	0.00045	0.99955	0.00045	60.00	228.00	1.0000	40.18	42.79	0.0003	T
12	716817	chr12:587078	587078	T	C	0.00025	0.99975	0.00025	60.00	113.00	1.0000	25.00	25.00	0.0020	T
12	716906	chr12:587167	587167	C	T	0.00048	0.99952	0.00048	60.00	228.00	1.0000	29.32	48.72	0.0016	C
12	716924	chr12:587185	587185	A	G	0.00061	0.99939	0.00061	60.00	228.00	1.0000	27.34	34.24	0.0045	A
12	716945	chr12:587206	587206	C	T	0.00039	0.99961	0.00039	60.00	228.00	1.0000	25.00	25.00	0.0054	C
12	716953	chr12:587214	587214	C	T	0.00027	0.99973	0.00027	60.00	36.00	1.0000	11.00	11.00	0.0669	C
12	716956	chr12:587217	587217	C	G	0.00027	0.99973	0.00027	60.00	71.00	1.0000	13.00	13.00	0.0639	C
12	716957	chr12:587218	587218	A	T	0.00046	0.99954	0.00046	60.00	47.00	1.0000	13.00	13.00	0.0380	A
12	716963	chr12:587224	587224	G	C	0.00039	0.99961	0.00039	60.00	228.00	1.0000	24.00	24.00	0.0185	G
12	717001	chr12:587262	587262	G	A	0.00041	0.99959	0.00041	60.00	228.00	1.0000	28.00	28.00	0.0525	G
12	717031	chr12:587292	587292	G	A	0.00044	0.99956	0.00044	60.00	228.00	1.0000	33.00	33.00	0.0009	A
12	717078	chr12:587339	587339	G	T	0.00039	0.99961	0.00039	60.00	228.00	1.0000	38.00	38.00	0.0000	G
12	717138	chr12:587399	587399	G	A	0.83987	0.16013	0.16013	60.00	219.59	0.5414	35.94	87.82	0.0024	A
12	717139	chr12:587400	587400	T	C	0.00078	0.99922	0.00078	60.00	161.67	1.0000	49.30	55.95	0.0359	T
12	717174	chr12:587435	587435	G	A	0.00056	0.99944	0.00056	60.00	228.00	1.0000	66.08	69.97	0.0005	G
12	717182	chr12:587443	587443	C	A	0.00025	0.99975	0.00025	60.00	228.00	1.0000	87.00	87.00	0.0005	C
12	717187	chr12:587448	587448	G	C	0.00039	0.99961	0.00039	60.00	228.00	1.0000	70.00	70.00	0.0000	G
12	717211	chr12:587472	587472	C	G	0.00044	0.99956	0.00044	60.00	228.00	1.0000	60.00	60.00	0.0000	C
12	717238	chr12:587499	587499	G	A	0.00025	0.99975	0.00025	60.00	228.00	1.0000	75.00	75.00	0.0005	G
12	717290	chr12:587551	587551	G	T	0.00030	0.99970	0.00030	60.00	228.00	1.0000	60.64	60.64	0.0003	G
12	717416	chr12:587677	587677	A	G	0.00025	0.99975	0.00025	60.00	228.00	1.0000	37.00	37.00	0.0015	A
12	717421	chr12:587682	587682	G	A	0.00025	0.99975	0.00025	60.00	228.00	1.0000	40.00	40.00	0.0015	G
12	717443	chr12:587704	587704	T	C	0.00025	0.99975	0.00025	60.00	228.00	1.0000	50.00	50.00	0.0010	T
12	717447	chr12:587708	587708	A	G	0.00056	0.99944	0.00056	60.00	216.26	1.0000	31.18	55.46	0.0007	A
12	717475	chr12:587736	587736	G	A	0.00025	0.99975	0.00025	60.00	199.00	1.0000	14.00	14.00	0.0020	G
12	717477	chr12:587738	587738	A	C	0.46908	0.53092	0.46908	60.00	214.17	0.0063	26.73	78.23	0.0036	a
12	717501	chr12:587762	587762	A	G	0.25229	0.74771	0.25229	60.00	216.73	0.0727	25.76	76.05	0.0141	A
12	717561	chr12:587822	587822	C	A	0.00048	0.99952	0.00048	60.00	228.00	1.0000	45.14	49.38	0.0019	C
12	717572	chr12:587833	587833	C	T	0.00025	0.99975	0.00025	60.00	228.00	1.0000	40.00	40.00	0.0045	C

12	717585	chr12:587846	587846	A	G	0.00045	0.99955	0.00045	60.00	228.00	1.0000	33.46	35.65	0.0025	A
12	717588	chr12:587849	587849	T	C	0.00048	0.99952	0.00048	60.00	228.00	1.0000	35.89	46.19	0.0035	T
12	717614	chr12:587875	587875	A	C	0.00039	0.99961	0.00039	60.00	228.00	1.0000	37.00	37.00	0.0000	A
12	717649	chr12:587910	587910	T	C	0.00096	0.99904	0.00096	60.00	117.50	1.0000	18.20	25.80	0.0795	T
12	717698	chr12:587959	587959	C	A	0.00039	0.99961	0.00039	60.00	228.00	1.0000	38.00	38.00	0.0000	C
12	717735	chr12:587996	587996	C	T	0.00041	0.99959	0.00041	60.00	228.00	1.0000	31.64	31.64	0.0053	C
12	717840	chr12:588101	588101	C	T	0.00044	0.99956	0.00044	60.00	228.00	1.0000	47.00	47.00	0.0000	C
12	717864	chr12:588125	588125	G	A	0.00050	0.99950	0.00050	60.00	228.00	1.0000	21.30	32.70	0.0065	G
12	717893	chr12:588154	588154	A	G	0.00058	0.99942	0.00058	60.00	76.20	1.0000	12.82	13.39	0.0201	A
12	717896	chr12:588157	588157	T	C	0.00125	0.99875	0.00125	60.00	85.54	1.0000	10.13	12.66	0.0063	T
12	717956	chr12:588217	588217	G	A	0.00025	0.99975	0.00025	60.00	228.00	1.0000	46.00	46.00	0.0025	G
12	717981	chr12:588242	588242	T	C	0.00025	0.99975	0.00025	60.00	228.00	1.0000	37.00	37.00	0.0055	T
12	718086	chr12:588347	588347	T	C	0.05484	0.94516	0.05484	60.00	227.50	0.3554	40.71	92.83	0.0020	T
12	718124	chr12:588385	588385	T	C	0.00050	0.99950	0.00050	60.00	145.50	1.0000	14.10	17.90	0.0025	T
12	718126	chr12:588387	588387	T	G	0.00050	0.99950	0.00050	60.00	162.00	1.0000	15.08	17.93	0.0045	T
12	718140	chr12:588401	588401	C	A	0.00041	0.99959	0.00041	60.00	228.00	1.0000	49.74	49.74	0.0000	C
12	718508	chr12:588769	588769	A	G	0.00131	0.99869	0.00131	59.70	95.97	1.0000	10.00	10.43	0.0568	A
12	718520	chr12:588781	588781	G	C	0.00706	0.99294	0.00706	59.86	217.99	1.0000	12.18	35.09	0.0544	G
12	718563	chr12:588824	588824	A	C	0.00041	0.99959	0.00041	60.00	228.00	1.0000	39.80	39.80	0.0004	A
12	718578	chr12:588839	588839	A	G	0.00025	0.99975	0.00025	60.00	228.00	1.0000	37.00	37.00	0.0190	A
12	718581	chr12:588842	588842	C	G	0.18554	0.81446	0.18554	60.00	217.29	0.1306	18.44	60.77	0.0151	C
12	718644	chr12:588905	588905	A	G	0.00039	0.99961	0.00039	60.00	228.00	1.0000	45.00	45.00	0.0000	A
12	719196	chr12:589457	589457	C	G	0.00025	0.99975	0.00025	60.00	129.00	1.0000	21.00	21.00	0.0105	C
12	719236	chr12:589497	589497	T	C	0.18733	0.81267	0.18733	60.00	227.19	0.0045	41.31	85.27	0.0007	T
12	719258	chr12:589519	589519	T	G	0.18876	0.81124	0.18876	60.00	227.58	0.1311	45.70	91.26	0.0023	T
12	719325	chr12:589586	589586	C	T	0.00025	0.99975	0.00025	60.00	228.00	1.0000	40.00	40.00	0.0010	C
12	719335	chr12:589596	589596	C	T	0.25811	0.74189	0.25811	60.00	222.41	0.0649	29.68	79.31	0.0050	C
12	719349	chr12:589610	589610	G	C	0.00050	0.99950	0.00050	60.00	228.00	1.0000	37.68	63.33	0.0010	G
12	719380	chr12:589641	589641	C	A	0.00039	0.99961	0.00039	60.00	228.00	1.0000	74.00	74.00	0.0000	C
12	719385	chr12:589646	589646	G	A	0.05508	0.94492	0.05508	60.00	225.90	0.3554	23.94	71.09	0.0023	G
12	719389	chr12:589650	589650	C	A	0.00044	0.99956	0.00044	60.00	228.00	1.0000	40.00	40.00	0.0000	C
12	719434	chr12:589695	589695	C	G	0.00026	0.99974	0.00026	60.00	33.00	1.0000	13.00	13.00	0.0539	C
12	719512	chr12:589773	589773	C	A	0.00044	0.99956	0.00044	60.00	228.00	1.0000	45.00	45.00	0.0009	C
12	719521	chr12:589782	589782	C	T	0.00025	0.99975	0.00025	60.00	228.00	1.0000	33.00	33.00	0.0105	C

12	719571	chr12:589832	589832	C	T	0.00025	0.99975	0.00025	60.00	228.00	1.0000	79.00	79.00	0.0105	C
12	719653	chr12:589914	589914	G	A	0.00048	0.99952	0.00048	60.00	228.00	1.0000	12.00	12.00	0.1898	G
12	719739	chr12:590000	590000	C	G	0.00039	0.99961	0.00039	60.00	228.00	1.0000	23.00	23.00	0.0023	C
12	719763	chr12:590024	590024	C	G	0.00044	0.99956	0.00044	60.00	228.00	1.0000	26.00	26.00	0.0009	C
12	719770	chr12:590031	590031	C	G	0.00025	0.99975	0.00025	60.00	228.00	1.0000	65.00	65.00	0.0005	C
12	719773	chr12:590034	590034	G	C	0.00044	0.99956	0.00044	60.00	228.00	1.0000	37.00	37.00	0.0000	G
12	719791	chr12:590052	590052	G	A	0.00080	0.99920	0.00080	60.00	228.00	1.0000	30.36	40.74	0.0003	G
12	719797	chr12:590058	590058	A	C	0.00044	0.99956	0.00044	60.00	228.00	1.0000	63.00	63.00	0.0000	A
12	719828	chr12:590089	590089	C	G	0.00039	0.99961	0.00039	60.00	228.00	1.0000	70.00	70.00	0.0000	C
12	719845	chr12:590106	590106	T	C	0.00045	0.99955	0.00045	60.00	228.00	1.0000	35.42	44.76	0.0003	T
12	719847	chr12:590108	590108	A	G	0.00045	0.99955	0.00045	60.00	228.00	1.0000	41.93	48.65	0.0003	A
12	720171	chr12:590432	590432	G	T	0.00025	0.99975	0.00025	59.00	228.00	1.0000	15.00	15.00	0.0040	G
12	720189	chr12:590450	590450	C	T	0.00044	0.99956	0.00044	60.00	228.00	1.0000	35.00	35.00	0.0000	C
12	720202	chr12:590463	590463	G	T	0.00044	0.99956	0.00044	60.00	168.00	1.0000	63.00	63.00	0.0000	G
12	720299	chr12:590560	590560	G	A	0.00025	0.99975	0.00025	60.00	228.00	1.0000	68.00	68.00	0.0000	G
12	720308	chr12:590569	590569	C	T	0.00039	0.99961	0.00039	60.00	228.00	1.0000	58.00	58.00	0.0000	C
12	720311	chr12:590572	590572	C	A	0.10061	0.89939	0.10061	59.99	220.97	0.1456	27.24	63.90	0.0029	C
12	720336	chr12:590597	590597	T	C	0.00078	0.99922	0.00078	60.00	111.00	1.0000	14.28	24.73	0.0123	T
12	720782	chr12:591043	591043	A	C	0.01020	0.98980	0.01020	58.82	164.78	0.0000	10.00	22.02	0.1542	A
12	720787	chr12:591048	591048	C	T	0.00252	0.99748	0.00252	59.15	202.40	0.0111	16.82	26.93	0.0132	C
12	720816	chr12:591077	591077	G	C	0.18441	0.81559	0.18441	59.99	219.25	0.1306	24.20	60.78	0.0123	G
12	720859	chr12:591120	591120	C	G	0.10239	0.89761	0.10239	60.00	226.11	0.1100	34.43	81.88	0.0005	C
12	720873	chr12:591134	591134	G	C	0.00044	0.99956	0.00044	60.00	228.00	1.0000	73.00	73.00	0.0000	G
12	720915	chr12:591176	591176	A	G	0.00088	0.99912	0.00088	60.00	228.00	1.0000	52.20	59.80	0.0000	G
12	720922	chr12:591183	591183	A	G	0.00327	0.99673	0.00327	60.00	228.00	1.0000	34.83	79.61	0.0002	A
12	720932	chr12:591193	591193	C	G	0.05808	0.94192	0.05808	60.00	227.25	0.3572	29.98	80.35	0.0013	C
12	720949	chr12:591210	591210	T	G	0.00045	0.99955	0.00045	60.00	228.00	1.0000	51.28	53.51	0.0016	T
12	721001	chr12:591262	591262	C	T	0.00041	0.99959	0.00041	60.00	228.00	1.0000	52.95	52.95	0.0000	C
12	721014	chr12:591275	591275	T	G	0.00048	0.99952	0.00048	60.00	228.00	1.0000	42.33	52.65	0.0000	T
12	721030	chr12:591291	591291	C	A	0.18845	0.81155	0.18845	60.00	223.00	0.1306	31.25	78.31	0.0018	C
12	721081	chr12:591342	591342	A	G	0.00025	0.99975	0.00025	60.00	228.00	1.0000	62.00	62.00	0.0010	A
12	721092	chr12:591353	591353	G	A	0.00610	0.99390	0.00610	60.00	228.00	1.0000	33.64	67.50	0.0007	G
12	721139	chr12:591400	591400	G	T	0.00025	0.99975	0.00025	60.00	228.00	1.0000	98.00	98.00	0.0010	G
12	721208	chr12:591469	591469	C	T	0.00025	0.99975	0.00025	60.00	228.00	1.0000	26.00	26.00	0.0050	C

12	721224	chr12:591485	591485	T	A	0.00079	0.99921	0.00079	60.00	228.00	1.0000	38.51	45.47	0.0025	T
12	721266	chr12:591527	591527	A	G	0.00045	0.99955	0.00045	60.00	228.00	1.0000	52.73	54.45	0.0009	A
12	721311	chr12:591572	591572	A	T	0.00025	0.99975	0.00025	60.00	47.00	1.0000	13.00	13.00	0.0055	A
12	721321	chr12:591582	591582	T	C	0.00025	0.99975	0.00025	60.00	228.00	1.0000	40.00	40.00	0.0055	T
12	721336	chr12:591597	591597	G	A	0.00045	0.99955	0.00045	60.00	228.00	1.0000	47.36	48.20	0.0029	G
12	721338	chr12:591599	591599	G	T	0.00044	0.99956	0.00044	60.00	90.00	1.0000	13.00	13.00	0.0018	G
12	721388	chr12:591649	591649	G	A	0.10041	0.89959	0.10041	60.00	222.03	0.2799	20.71	66.66	0.0043	G
12	721411	chr12:591672	591672	G	A	0.00025	0.99975	0.00025	60.00	228.00	1.0000	69.00	69.00	0.0040	G
12	721441	chr12:591702	591702	G	A	0.00039	0.99961	0.00039	60.00	228.00	1.0000	75.00	75.00	0.0000	G
12	721469	chr12:591730	591730	A	T	0.07044	0.92956	0.07044	60.00	221.85	0.5497	19.31	64.26	0.0083	A
12	721485	chr12:591746	591746	G	T	0.00044	0.99956	0.00044	60.00	46.00	1.0000	10.00	10.00	0.0071	G
12	721487	chr12:591748	591748	G	T	0.00025	0.99975	0.00025	60.00	228.00	1.0000	77.00	77.00	0.0115	G
12	721502	chr12:591763	591763	C	T	0.00025	0.99975	0.00025	60.00	228.00	1.0000	53.00	53.00	0.0080	C
12	721510	chr12:591771	591771	G	A	0.00025	0.99975	0.00025	60.00	228.00	1.0000	73.00	73.00	0.0075	G
12	721540	chr12:591801	591801	G	C	0.00039	0.99961	0.00039	60.00	228.00	1.0000	65.00	65.00	0.0000	G
12	721589	chr12:591850	591850	G	A	0.00039	0.99961	0.00039	60.00	228.00	1.0000	67.00	67.00	0.0000	G
12	721601	chr12:591862	591862	C	T	0.00076	0.99924	0.00076	60.00	228.00	1.0000	44.41	48.24	0.0033	C
12	721602	chr12:591863	591863	G	A	0.00050	0.99950	0.00050	60.00	228.00	1.0000	29.18	35.83	0.0055	A
12	721623	chr12:591884	591884	C	A	0.00091	0.99909	0.00091	60.00	213.93	1.0000	27.13	37.53	0.0032	C
12	721660	chr12:591921	591921	A	C	0.00025	0.99975	0.00025	60.00	228.00	1.0000	44.00	44.00	0.0025	A
12	721696	chr12:591957	591957	A	G	0.00039	0.99961	0.00039	60.00	228.00	1.0000	46.00	46.00	0.0000	A
12	721769	chr12:592030	592030	G	T	0.13490	0.86510	0.13490	60.00	222.37	0.0130	19.26	65.32	0.0079	G
12	721821	chr12:592082	592082	C	T	0.00088	0.99912	0.00088	60.00	255.00	1.0000	96.00	96.00	0.0000	C
12	721862	chr12:592123	592123	T	C	0.00039	0.99961	0.00039	60.00	228.00	1.0000	72.00	72.00	0.0000	T
12	721914	chr12:592175	592175	G	A	0.00050	0.99950	0.00050	60.00	208.00	1.0000	11.78	41.23	0.0005	G
12	721948	chr12:592209	592209	G	A	0.00025	0.99975	0.00025	60.00	228.00	1.0000	43.00	43.00	0.0010	G
12	721980	chr12:592241	592241	C	T	0.00039	0.99961	0.00039	60.00	228.00	1.0000	24.00	24.00	0.0000	C
12	721988	chr12:592249	592249	T	C	0.00043	0.99957	0.00043	60.00	162.60	1.0000	24.38	24.38	0.0333	T
12	722014	chr12:592275	592275	G	A	0.00025	0.99975	0.00025	60.00	228.00	1.0000	28.00	28.00	0.0125	G
12	722021	chr12:592282	592282	C	T	0.00044	0.99956	0.00044	60.00	228.00	1.0000	36.00	36.00	0.0062	C
12	722033	chr12:592294	592294	T	C	0.00044	0.99956	0.00044	60.00	221.00	1.0000	31.00	31.00	0.0009	T
12	722070	chr12:592331	592331	G	C	0.00039	0.99961	0.00039	60.00	228.00	1.0000	61.00	61.00	0.0000	G
12	722088	chr12:592349	592349	C	A	0.00044	0.99956	0.00044	60.00	91.00	1.0000	34.00	34.00	0.0035	C
12	722146	chr12:592407	592407	C	T	0.00044	0.99956	0.00044	60.00	228.00	1.0000	28.00	28.00	0.0009	C

12	722220	chr12:592481	592481	C	T	0.00048	0.99952	0.00048	60.00	228.00	1.0000	28.67	37.18	0.0070	C
12	722265	chr12:592526	592526	T	G	0.00045	0.99955	0.00045	60.00	228.00	1.0000	22.00	22.00	0.0106	T
12	722266	chr12:592527	592527	C	T	0.00051	0.99949	0.00051	60.00	218.50	1.0000	19.48	37.53	0.0150	C
12	722289	chr12:592550	592550	G	T	0.00044	0.99956	0.00044	60.00	228.00	1.0000	50.00	50.00	0.0053	G
12	722314	chr12:592575	592575	G	A	0.00039	0.99961	0.00039	60.00	228.00	1.0000	84.00	84.00	0.0000	G
12	722353	chr12:592614	592614	T	C	0.00039	0.99961	0.00039	60.00	228.00	1.0000	51.00	51.00	0.0000	T
12	722372	chr12:592633	592633	A	G	0.01762	0.98238	0.01762	60.00	227.78	0.3411	30.79	68.74	0.0009	A
12	722427	chr12:592688	592688	T	C	0.00025	0.99975	0.00025	60.00	228.00	1.0000	31.00	31.00	0.0015	T
12	722434	chr12:592695	592695	T	C	0.00032	0.99968	0.00032	60.00	112.53	1.0000	20.75	20.75	0.0038	T
12	722440	chr12:592701	592701	T	A	0.00044	0.99956	0.00044	60.00	200.00	1.0000	23.00	23.00	0.0018	T
12	722473	chr12:592734	592734	G	A	0.00030	0.99970	0.00030	60.00	228.00	1.0000	48.07	48.07	0.0000	G
12	722474	chr12:592735	592735	C	A	0.00025	0.99975	0.00025	60.00	228.00	1.0000	31.00	31.00	0.0000	C
12	723246	chr12:593507	593507	A	G	0.00049	0.99951	0.00049	60.00	66.18	1.0000	17.68	26.14	0.0146	A
12	723252	chr12:593513	593513	G	A	0.00075	0.99925	0.00075	60.00	47.33	1.0000	18.30	24.95	0.0080	G
12	723290	chr12:593551	593551	A	G	0.77902	0.22098	0.22098	60.00	191.17	0.0265	23.92	74.76	0.0123	G
12	723321	chr12:593582	593582	A	G	0.00064	0.99936	0.00064	60.00	44.48	1.0000	12.31	21.40	0.0025	A
12	723322	chr12:593583	593583	A	G	0.00025	0.99975	0.00025	60.00	228.00	1.0000	22.00	22.00	0.0000	A
12	723374	chr12:593635	593635	A	T	0.10994	0.89006	0.10994	60.00	224.75	0.5457	33.41	86.81	0.0014	A
12	723504	chr12:593765	593765	G	A	0.00025	0.99975	0.00025	57.00	228.00	1.0000	20.00	20.00	0.0010	G
12	723506	chr12:593767	593767	C	T	0.00032	0.99968	0.00032	53.64	228.00	1.0000	22.20	22.20	0.0013	C
12	723584	chr12:593845	593845	G	T	0.00026	0.99974	0.00026	60.00	175.00	1.0000	15.00	15.00	0.0325	G
12	723599	chr12:593860	593860	G	A	0.00397	0.99603	0.00397	59.86	185.21	1.0000	10.00	30.35	0.0559	G
12	723658	chr12:593919	593919	C	T	0.00026	0.99974	0.00026	60.00	228.00	1.0000	26.00	26.00	0.0544	C
12	723659	chr12:593920	593920	G	A	0.00039	0.99961	0.00039	60.00	180.00	1.0000	23.00	23.00	0.0077	G
12	723664	chr12:593925	593925	G	A	0.00039	0.99961	0.00039	60.00	228.00	1.0000	33.00	33.00	0.0062	G
12	723672	chr12:593933	593933	G	A	0.00026	0.99974	0.00026	60.00	228.00	1.0000	43.00	43.00	0.0285	G
12	723699	chr12:593960	593960	C	G	0.00057	0.99943	0.00057	60.00	215.85	1.0000	21.76	25.12	0.0126	C
12	723700	chr12:593961	593961	G	C	0.00137	0.99863	0.00137	60.00	193.28	1.0000	20.68	40.71	0.0123	G
12	723735	chr12:593996	593996	C	A	0.00077	0.99923	0.00077	60.00	228.00	1.0000	57.08	59.93	0.0000	C
12	723741	chr12:594002	594002	C	T	0.00725	0.99275	0.00725	60.00	228.00	1.0000	28.42	65.36	0.0045	C
12	723755	chr12:594016	594016	A	G	0.00039	0.99961	0.00039	60.00	228.00	1.0000	48.00	48.00	0.0000	A
12	723801	chr12:594062	594062	C	A	0.00025	0.99975	0.00025	60.00	209.00	1.0000	37.00	37.00	0.0005	C
12	723807	chr12:594068	594068	G	T	0.00044	0.99956	0.00044	60.00	228.00	1.0000	57.00	57.00	0.0018	G
12	723886	chr12:594147	594147	T	C	0.00025	0.99975	0.00025	60.00	228.00	1.0000	27.00	27.00	0.0045	T

12	723902	chr12:594163	594163	G	A	0.36950	0.63050	0.36950	60.00	206.59	0.0390	18.04	61.22	0.0201	A
12	723939	chr12:594200	594200	C	G	0.00045	0.99955	0.00045	60.00	228.00	1.0000	33.00	33.00	0.0133	C
12	723981	chr12:594242	594242	A	G	0.36454	0.63546	0.36454	60.00	201.35	0.0306	15.06	59.20	0.0283	G
12	724000	chr12:594261	594261	C	T	0.00039	0.99961	0.00039	60.00	228.00	1.0000	49.00	49.00	0.0000	C
12	724050	chr12:594311	594311	A	G	0.00044	0.99956	0.00044	60.00	207.00	1.0000	18.00	18.00	0.0027	A
12	724078	chr12:594339	594339	G	A	0.00039	0.99961	0.00039	60.00	228.00	1.0000	30.00	30.00	0.0000	G
12	724087	chr12:594348	594348	C	T	0.00025	0.99975	0.00025	60.00	228.00	1.0000	39.00	39.00	0.0100	C
12	724148	chr12:594409	594409	G	A	0.00039	0.99961	0.00039	60.00	228.00	1.0000	56.00	56.00	0.0000	G
12	724158	chr12:594419	594419	C	G	0.00025	0.99975	0.00025	60.00	228.00	1.0000	42.00	42.00	0.0015	C
12	724259	chr12:594520	594520	T	C	0.00025	0.99975	0.00025	60.00	228.00	1.0000	47.00	47.00	0.0000	T
12	724271	chr12:594532	594532	A	G	0.00025	0.99975	0.00025	60.00	228.00	1.0000	38.00	38.00	0.0005	A
12	724279	chr12:594540	594540	C	T	0.25924	0.74076	0.25924	60.00	220.80	0.0547	29.13	77.98	0.0049	C
12	724315	chr12:594576	594576	C	T	0.00039	0.99961	0.00039	60.00	228.00	1.0000	74.00	74.00	0.0000	C
12	724331	chr12:594592	594592	A	G	0.00039	0.99961	0.00039	60.00	228.00	1.0000	61.00	61.00	0.0000	A
12	724332	chr12:594593	594593	C	T	0.00048	0.99952	0.00048	60.00	228.00	1.0000	49.13	53.99	0.0006	C
12	724338	chr12:594599	594599	A	G	0.00050	0.99950	0.00050	60.00	228.00	1.0000	37.63	61.38	0.0005	A
12	724347	chr12:594608	594608	C	A	0.00937	0.99063	0.00937	60.00	226.74	1.0000	30.28	73.81	0.0009	C
12	724420	chr12:594681	594681	T	A	0.00700	0.99300	0.00700	60.00	228.00	1.0000	31.37	68.39	0.0007	T
12	724461	chr12:594722	594722	G	A	0.05809	0.94191	0.05809	60.00	226.18	0.3557	29.47	76.06	0.0016	A
12	724501	chr12:594762	594762	T	C	0.00039	0.99961	0.00039	59.00	228.00	1.0000	18.00	18.00	0.0008	T
12	724760	chr12:595021	595021	A	C	0.00028	0.99972	0.00028	60.00	51.00	1.0000	10.00	10.00	0.0969	A
12	724812	chr12:595073	595073	G	C	0.00039	0.99961	0.00039	60.00	228.00	1.0000	73.00	73.00	0.0000	G
12	724835	chr12:595096	595096	C	T	0.00039	0.99961	0.00039	60.00	228.00	1.0000	57.00	57.00	0.0000	C
12	724836	chr12:595097	595097	G	A	0.00046	0.99954	0.00046	60.00	228.00	1.0000	51.06	54.43	0.0027	G
12	724889	chr12:595150	595150	T	G	0.00025	0.99975	0.00025	60.00	228.00	1.0000	53.00	53.00	0.0010	T
12	724900	chr12:595161	595161	G	A	0.00025	0.99975	0.00025	60.00	228.00	1.0000	30.00	30.00	0.0040	G
12	724927	chr12:595188	595188	G	A	0.00025	0.99975	0.00025	60.00	228.00	1.0000	33.00	33.00	0.0080	G
12	724929	chr12:595190	595190	G	A	0.00025	0.99975	0.00025	60.00	228.00	1.0000	24.00	24.00	0.0100	G
12	724946	chr12:595207	595207	G	A	0.00026	0.99974	0.00026	60.00	129.00	1.0000	21.00	21.00	0.0344	G
12	724947	chr12:595208	595208	G	A	0.00051	0.99949	0.00051	60.00	40.00	1.0000	10.00	10.00	0.1413	G
12	724971	chr12:595232	595232	A	C	0.00215	0.99785	0.00215	60.00	58.28	1.0000	11.00	16.83	0.1073	A
12	725020	chr12:595281	595281	C	G	0.00080	0.99920	0.00080	60.00	228.00	1.0000	22.24	39.18	0.0057	C
12	725083	chr12:595344	595344	G	A	0.00025	0.99975	0.00025	60.00	228.00	1.0000	28.00	28.00	0.0080	G
12	725105	chr12:595366	595366	C	T	0.00466	0.99534	0.00466	60.00	224.21	1.0000	21.29	47.37	0.0074	C

12	725115	chr12:595376	595376	G	T	0.00039	0.99961	0.00039	60.00	228.00	1.0000	50.00	50.00	0.0000	G
12	725140	chr12:595401	595401	T	C	0.00025	0.99975	0.00025	60.00	226.00	1.0000	21.00	21.00	0.0030	T
12	725230	chr12:595491	595491	A	G	0.00096	0.99904	0.00096	60.00	228.00	1.0000	41.92	49.59	0.0013	A
12	725300	chr12:595561	595561	T	C	0.00044	0.99956	0.00044	60.00	105.00	1.0000	15.00	15.00	0.0062	T
12	725303	chr12:595564	595564	C	A	0.00025	0.99975	0.00025	60.00	228.00	1.0000	51.00	51.00	0.0005	C
12	725313	chr12:595574	595574	G	A	0.00102	0.99898	0.00102	60.00	228.00	1.0000	28.80	47.22	0.0016	G
12	725342	chr12:595603	595603	G	C	0.00045	0.99955	0.00045	60.00	228.00	1.0000	23.00	23.00	0.0106	G
12	725403	chr12:595664	595664	G	A	0.00025	0.99975	0.00025	60.00	152.00	1.0000	14.00	14.00	0.0000	G
12	725596	chr12:595857	595857	G	C	0.00039	0.99961	0.00039	54.00	205.00	1.0000	17.00	17.00	0.0000	G
12	725653	chr12:595914	595914	A	G	0.00025	0.99975	0.00025	60.00	65.00	1.0000	10.00	10.00	0.0005	A
12	725661	chr12:595922	595922	T	G	0.00091	0.99909	0.00091	58.89	226.30	1.0000	16.22	21.90	0.0036	T
12	725678	chr12:595939	595939	G	C	0.00061	0.99939	0.00061	59.60	228.00	1.0000	31.80	42.75	0.0006	G
12	725684	chr12:595945	595945	T	C	0.00025	0.99975	0.00025	60.00	228.00	1.0000	30.00	30.00	0.0005	T
12	725697	chr12:595958	595958	A	C	0.00272	0.99728	0.00272	60.00	212.61	1.0000	28.15	47.85	0.0036	A
12	725736	chr12:595997	595997	A	T	0.00076	0.99924	0.00076	60.00	228.00	1.0000	22.35	32.12	0.0003	A
12	725746	chr12:596007	596007	G	T	0.00045	0.99955	0.00045	60.00	228.00	1.0000	23.00	23.00	0.0141	G
12	725749	chr12:596010	596010	G	A	0.00045	0.99955	0.00045	60.00	228.00	1.0000	68.00	68.00	0.0097	G
12	725757	chr12:596018	596018	C	T	0.00039	0.99961	0.00039	60.00	228.00	1.0000	57.00	57.00	0.0000	C
12	725759	chr12:596020	596020	C	T	0.00025	0.99975	0.00025	60.00	228.00	1.0000	47.00	47.00	0.0015	C
12	725794	chr12:596055	596055	G	A	0.00025	0.99975	0.00025	60.00	228.00	1.0000	27.00	27.00	0.0015	G
12	725815	chr12:596076	596076	T	C	0.00075	0.99925	0.00075	60.00	228.00	1.0000	22.85	60.85	0.0000	T
12	725822	chr12:596083	596083	C	T	0.00044	0.99956	0.00044	60.00	228.00	1.0000	20.00	20.00	0.0035	C
12	725875	chr12:596136	596136	G	A	0.00039	0.99961	0.00039	60.00	228.00	1.0000	82.00	82.00	0.0000	G
12	725876	chr12:596137	596137	T	A	0.00048	0.99952	0.00048	60.00	228.00	1.0000	67.93	72.74	0.0000	T
12	725935	chr12:596196	596196	C	A	0.00090	0.99910	0.00090	60.00	228.00	1.0000	41.70	56.35	0.0002	C
12	725961	chr12:596222	596222	G	C	0.00025	0.99975	0.00025	60.00	228.00	1.0000	45.00	45.00	0.0000	G
12	726015	chr12:596276	596276	G	C	0.77538	0.22462	0.22462	60.00	187.63	0.0020	21.75	76.45	0.0038	C
12	726022	chr12:596283	596283	G	C	0.00351	0.99649	0.00351	60.00	227.02	1.0000	26.69	68.00	0.0006	G
12	726083	chr12:596344	596344	T	C	0.00044	0.99956	0.00044	60.00	228.00	1.0000	34.00	34.00	0.0053	T
12	726094	chr12:596355	596355	G	A	0.00039	0.99961	0.00039	60.00	152.00	1.0000	24.00	24.00	0.0000	g
12	726422	chr12:596683	596683	A	C	0.00133	0.99867	0.00133	60.00	228.00	1.0000	32.40	59.00	0.0000	A
12	726432	chr12:596693	596693	T	C	0.00339	0.99661	0.00339	60.00	228.00	1.0000	39.98	75.95	0.0000	T
12	726448	chr12:596709	596709	G	A	0.00025	0.99975	0.00025	60.00	228.00	1.0000	53.00	53.00	0.0005	G
12	726452	chr12:596713	596713	C	T	0.00025	0.99975	0.00025	60.00	228.00	1.0000	47.00	47.00	0.0005	C

12	726596	chr12:596857	596857	C	T	0.00025	0.99975	0.00025	60.00	228.00	1.0000	81.00	81.00	0.0175	c
12	726619	chr12:596880	596880	C	T	0.00025	0.99975	0.00025	60.00	228.00	1.0000	57.00	57.00	0.0210	C
12	726639	chr12:596900	596900	T	C	0.00039	0.99961	0.00039	60.00	228.00	1.0000	72.00	72.00	0.0000	T
12	726685	chr12:596946	596946	T	A	0.00026	0.99974	0.00026	60.00	120.00	1.0000	19.00	19.00	0.0275	T
12	726698	chr12:596959	596959	C	A	0.00026	0.99974	0.00026	60.00	228.00	1.0000	26.00	26.00	0.0275	C
12	726809	chr12:597070	597070	C	T	0.00156	0.99844	0.00156	60.00	228.00	1.0000	22.00	29.63	0.0131	C
12	726849	chr12:597110	597110	G	A	0.13089	0.86911	0.13089	60.00	212.26	0.1125	12.99	45.19	0.1124	G
12	726876	chr12:597137	597137	T	C	0.00045	0.99955	0.00045	60.00	228.00	1.0000	32.00	32.00	0.0247	T
12	726877	chr12:597138	597138	C	G	0.15629	0.84371	0.15629	60.00	193.99	0.7313	10.92	42.76	0.0769	C
12	726892	chr12:597153	597153	G	C	0.00026	0.99974	0.00026	60.00	228.00	1.0000	26.00	26.00	0.0349	G
12	726947	chr12:597208	597208	G	C	0.00039	0.99961	0.00039	60.00	228.00	1.0000	27.00	27.00	0.0039	G
12	727015	chr12:597276	597276	G	A	0.00028	0.99972	0.00028	60.00	119.00	1.0000	10.00	10.00	0.1088	G
12	727100	chr12:597361	597361	C	T	0.00039	0.99961	0.00039	60.00	228.00	1.0000	30.00	30.00	0.0000	C
12	727137	chr12:597398	597398	T	C	0.00039	0.99961	0.00039	60.00	228.00	1.0000	66.00	66.00	0.0000	T
12	727168	chr12:597429	597429	G	T	0.15356	0.84644	0.15356	60.00	219.49	0.7037	20.51	60.54	0.0152	G
12	727256	chr12:597517	597517	G	A	0.00025	0.99975	0.00025	60.00	200.00	1.0000	32.00	32.00	0.0070	G
12	727266	chr12:597527	597527	A	T	0.00025	0.99975	0.00025	60.00	228.00	1.0000	31.00	31.00	0.0090	A
12	727302	chr12:597563	597563	G	A	0.00039	0.99961	0.00039	60.00	194.00	1.0000	64.00	64.00	0.0000	G
12	727314	chr12:597575	597575	T	A	0.00050	0.99950	0.00050	60.00	64.00	1.0000	11.25	20.75	0.0085	G
12	727335	chr12:597596	597596	G	A	0.07716	0.92284	0.07716	60.00	196.16	0.0005	20.68	57.26	0.0650	G
12	727344	chr12:597605	597605	A	G	0.05408	0.94592	0.05408	60.00	221.85	0.5137	21.62	60.39	0.0294	G
12	727533	chr12:597794	597794	G	A	0.00040	0.99960	0.00040	60.00	228.00	1.0000	20.00	20.00	0.0370	g
12	727547	chr12:597808	597808	G	A	0.00039	0.99961	0.00039	60.00	228.00	1.0000	26.00	26.00	0.0046	g
12	727549	chr12:597810	597810	G	C	0.00032	0.99968	0.00032	60.00	228.00	1.0000	29.34	29.34	0.0538	g
12	727583	chr12:597844	597844	C	A	0.07695	0.92305	0.07695	60.00	216.12	0.0378	18.18	49.02	0.0721	c
12	727586	chr12:597847	597847	C	G	0.00194	0.99806	0.00194	60.00	225.49	1.0000	26.47	42.06	0.0128	c
12	727596	chr12:597857	597857	T	C	0.00025	0.99975	0.00025	60.00	228.00	1.0000	56.00	56.00	0.0140	t
12	727610	chr12:597871	597871	G	A	0.00039	0.99961	0.00039	60.00	228.00	1.0000	59.00	59.00	0.0000	g
12	727620	chr12:597881	597881	G	A	0.15601	0.84399	0.15601	60.00	222.35	0.7071	25.91	68.96	0.0090	g
12	727671	chr12:597932	597932	G	C	0.00025	0.99975	0.00025	60.00	228.00	1.0000	72.00	72.00	0.0050	G
12	727816	chr12:598077	598077	C	T	0.00039	0.99961	0.00039	60.00	228.00	1.0000	50.00	50.00	0.0000	C
12	727825	chr12:598086	598086	G	A	0.00045	0.99955	0.00045	60.00	228.00	1.0000	44.00	44.00	0.0159	G
12	727840	chr12:598101	598101	T	C	0.00039	0.99961	0.00039	60.00	228.00	1.0000	42.00	42.00	0.0000	T
12	727861	chr12:598122	598122	G	C	0.17724	0.82276	0.17724	60.00	209.40	0.0110	15.17	56.58	0.0279	G

12	727879	chr12:598140	598140	G	A	0.00045	0.99955	0.00045	60.00	197.00	1.0000	30.00	30.00	0.0080	G
12	727917	chr12:598178	598178	G	A	0.00026	0.99974	0.00026	60.00	223.00	1.0000	21.00	21.00	0.0434	G
12	727957	chr12:598218	598218	G	A	0.10955	0.89045	0.10955	60.00	221.11	0.5078	17.30	65.50	0.0141	A
12	727992	chr12:598253	598253	G	T	0.00033	0.99967	0.00033	60.00	228.00	1.0000	27.35	27.35	0.0242	G
12	728005	chr12:598266	598266	T	C	0.00045	0.99955	0.00045	60.00	72.00	1.0000	10.00	10.00	0.0239	T
12	728022	chr12:598283	598283	T	C	0.00039	0.99961	0.00039	60.00	69.00	1.0000	15.00	15.00	0.0031	T
12	728099	chr12:598360	598360	C	A	0.00039	0.99961	0.00039	60.00	228.00	1.0000	55.00	55.00	0.0000	C
12	728115	chr12:598376	598376	G	A	0.00061	0.99939	0.00061	60.00	228.00	1.0000	29.25	33.00	0.0048	g
12	728116	chr12:598377	598377	T	C	0.00025	0.99975	0.00025	60.00	228.00	1.0000	30.00	30.00	0.0080	T
12	728157	chr12:598418	598418	A	G	0.00039	0.99961	0.00039	60.00	228.00	1.0000	70.00	70.00	0.0000	A
12	728159	chr12:598420	598420	G	C	0.00045	0.99955	0.00045	60.00	158.00	1.0000	24.00	24.00	0.0097	G
12	728177	chr12:598438	598438	A	G	0.00025	0.99975	0.00025	60.00	228.00	1.0000	22.00	22.00	0.0095	A
12	728180	chr12:598441	598441	G	A	0.00050	0.99950	0.00050	60.00	187.00	0.0005	53.00	53.00	0.0090	G
12	728203	chr12:598464	598464	C	T	0.00032	0.99968	0.00032	60.00	228.00	1.0000	24.00	24.00	0.0064	C
12	728233	chr12:598494	598494	T	C	0.00025	0.99975	0.00025	60.00	228.00	1.0000	56.00	56.00	0.0095	T
12	728305	chr12:598566	598566	G	A	0.00032	0.99968	0.00032	60.00	228.00	1.0000	24.53	24.53	0.0016	G
12	728325	chr12:598586	598586	T	C	0.00039	0.99961	0.00039	60.00	228.00	1.0000	70.00	70.00	0.0000	T
12	728332	chr12:598593	598593	G	T	0.00025	0.99975	0.00025	60.00	104.00	1.0000	15.00	15.00	0.0015	G
12	728336	chr12:598597	598597	C	T	0.00025	0.99975	0.00025	60.00	228.00	1.0000	35.00	35.00	0.0010	C
12	728348	chr12:598609	598609	T	C	0.00203	0.99797	0.00203	60.00	228.00	1.0000	41.75	66.85	0.0009	T
12	728357	chr12:598618	598618	T	A	0.00025	0.99975	0.00025	60.00	228.00	1.0000	61.00	61.00	0.0015	T
12	728360	chr12:598621	598621	A	G	0.10646	0.89354	0.10646	60.00	221.25	0.3991	22.31	77.27	0.0047	A
12	728369	chr12:598630	598630	G	C	0.10323	0.89677	0.10323	60.00	220.76	0.5821	22.22	76.84	0.0085	C
12	728379	chr12:598640	598640	G	A	0.00050	0.99950	0.00050	60.00	228.00	1.0000	50.28	60.73	0.0020	G
12	728391	chr12:598652	598652	G	T	0.00025	0.99975	0.00025	60.00	68.00	1.0000	18.00	18.00	0.0065	G
12	728392	chr12:598653	598653	G	A	0.00032	0.99968	0.00032	60.00	228.00	1.0000	41.38	41.38	0.0035	G
12	728393	chr12:598654	598654	G	A	0.00077	0.99923	0.00077	60.00	228.00	1.0000	40.28	50.73	0.0000	G
12	728426	chr12:598687	598687	C	T	0.00039	0.99961	0.00039	60.00	228.00	1.0000	53.00	53.00	0.0000	C
12	728441	chr12:598702	598702	C	T	0.00025	0.99975	0.00025	60.00	228.00	1.0000	49.00	49.00	0.0035	C
12	728490	chr12:598751	598751	C	T	0.00025	0.99975	0.00025	60.00	228.00	1.0000	27.00	27.00	0.0020	C
12	728508	chr12:598769	598769	C	T	0.00061	0.99939	0.00061	60.00	228.00	1.0000	43.03	63.20	0.0015	C
12	728546	chr12:598807	598807	G	T	0.00025	0.99975	0.00025	60.00	226.00	1.0000	19.00	19.00	0.0035	G
12	728564	chr12:598825	598825	C	T	0.10180	0.89820	0.10180	60.00	222.21	0.1083	19.28	65.06	0.0068	C
12	728588	chr12:598849	598849	G	A	0.10522	0.89478	0.10522	60.00	216.19	0.2673	12.77	46.82	0.0270	A

12	728593	chr12:598854	598854	T	A	0.00025	0.99975	0.00025	60.00	168.00	1.0000	17.00	17.00	0.0135 T
12	728600	chr12:598861	598861	G	A	0.00025	0.99975	0.00025	60.00	189.00	1.0000	23.00	23.00	0.0170 G
12	728604	chr12:598865	598865	C	T	0.00045	0.99955	0.00045	60.00	74.00	1.0000	12.00	12.00	0.0106 C
12	728637	chr12:598898	598898	G	A	0.00025	0.99975	0.00025	60.00	94.00	1.0000	14.00	14.00	0.0020 G
12	728710	chr12:598971	598971	G	A	0.26082	0.73918	0.26082	60.00	224.11	0.0543	31.36	83.82	0.0036 G
12	728718	chr12:598979	598979	C	T	0.00044	0.99956	0.00044	60.00	228.00	1.0000	65.00	65.00	0.0009 C
12	728719	chr12:598980	598980	G	T	0.00039	0.99961	0.00039	60.00	228.00	1.0000	56.00	56.00	0.0000 G
12	728735	chr12:598996	598996	G	A	0.00039	0.99961	0.00039	60.00	228.00	1.0000	69.00	69.00	0.0000 G
12	728789	chr12:599050	599050	C	G	0.00044	0.99956	0.00044	60.00	228.00	1.0000	73.00	73.00	0.0009 C
12	728795	chr12:599056	599056	G	A	0.00025	0.99975	0.00025	60.00	228.00	1.0000	64.00	64.00	0.0010 G
12	728811	chr12:599072	599072	C	T	0.00025	0.99975	0.00025	60.00	228.00	1.0000	75.00	75.00	0.0005 C
12	728822	chr12:599083	599083	G	T	0.00044	0.99956	0.00044	60.00	228.00	1.0000	42.00	42.00	0.0009 G
12	728857	chr12:599118	599118	A	G	0.00847	0.99153	0.00847	60.00	226.31	1.0000	31.38	76.29	0.0007 A
12	728910	chr12:599171	599171	C	A	0.00044	0.99956	0.00044	60.00	111.00	1.0000	14.00	14.00	0.0000 C
12	728948	chr12:599209	599209	A	G	0.00044	0.99956	0.00044	60.00	228.00	1.0000	69.00	69.00	0.0000 A
12	728997	chr12:599258	599258	G	A	0.00025	0.99975	0.00025	60.00	228.00	1.0000	53.00	53.00	0.0010 G
12	729021	chr12:599282	599282	A	C	0.00025	0.99975	0.00025	60.00	228.00	1.0000	45.00	45.00	0.0020 A
12	729141	chr12:599402	599402	G	T	0.00044	0.99956	0.00044	60.00	228.00	1.0000	25.00	25.00	0.0053 G
12	729265	chr12:599526	599526	G	A	0.00050	0.99950	0.00050	60.00	228.00	1.0000	57.10	60.90	0.0005 G
12	729297	chr12:599558	599558	C	T	0.00045	0.99955	0.00045	60.00	228.00	1.0000	46.41	56.49	0.0006 G
12	729298	chr12:599559	599559	G	A	0.00025	0.99975	0.00025	60.00	228.00	1.0000	44.00	44.00	0.0010 G
12	729314	chr12:599575	599575	T	A	0.00044	0.99956	0.00044	60.00	106.00	1.0000	49.00	49.00	0.0035 T
12	729348	chr12:599609	599609	G	A	0.00102	0.99898	0.00102	60.00	228.00	1.0000	44.97	59.03	0.0011 G
12	729386	chr12:599647	599647	C	T	0.00114	0.99886	0.00114	60.00	64.25	1.0000	18.08	21.85	0.1203 c
12	729387	chr12:599648	599648	T	C	0.00025	0.99975	0.00025	60.00	80.00	1.0000	12.00	12.00	0.0065 C
12	729447	chr12:599708	599708	T	C	0.00025	0.99975	0.00025	60.00	228.00	1.0000	49.00	49.00	0.0005 T
12	729505	chr12:599766	599766	G	A	0.05571	0.94429	0.05571	60.00	223.89	0.5171	15.97	58.34	0.0094 G
12	729517	chr12:599778	599778	G	A	0.00025	0.99975	0.00025	60.00	228.00	1.0000	50.00	50.00	0.0045 G
12	729562	chr12:599823	599823	A	G	0.00039	0.99961	0.00039	60.00	228.00	1.0000	35.00	35.00	0.0000 A
12	729568	chr12:599829	599829	A	G	0.00025	0.99975	0.00025	60.00	228.00	1.0000	117.00	117.00	0.0010 A
12	729578	chr12:599839	599839	T	C	0.00039	0.99961	0.00039	60.00	228.00	1.0000	58.00	58.00	0.0000 T
12	729623	chr12:599884	599884	T	C	0.00772	0.99228	0.00772	60.00	228.00	1.0000	39.38	79.00	0.0000 T
12	729627	chr12:599888	599888	G	C	0.00039	0.99961	0.00039	60.00	228.00	1.0000	78.00	78.00	0.0000 G
12	729712	chr12:599973	599973	A	G	0.00530	0.99470	0.00530	60.00	228.00	1.0000	44.61	85.96	0.0000 A

12	729749	chr12:600010	600010	A	G	0.00044	0.99956	0.00044	60.00	228.00	1.0000	48.00	48.00	0.0000	A
12	729803	chr12:600064	600064	T	A	0.00047	0.99953	0.00047	60.00	117.00	1.0000	12.00	12.00	0.0654	-
12	730203	chr12:600464	600464	C	G	0.00045	0.99955	0.00045	60.00	228.00	1.0000	72.34	75.80	0.0000	C
12	730225	chr12:600486	600486	T	A	0.15753	0.84247	0.15753	60.00	227.24	0.6259	40.06	88.07	0.0000	T
12	730254	chr12:600515	600515	T	C	0.00121	0.99879	0.00121	60.00	228.00	1.0000	65.35	75.07	0.0000	T
12	730259	chr12:600520	600520	G	A	0.00025	0.99975	0.00025	60.00	228.00	1.0000	78.00	78.00	0.0000	G
12	730324	chr12:600585	600585	T	C	0.00025	0.99975	0.00025	60.00	228.00	1.0000	42.00	42.00	0.0000	T
12	730336	chr12:600597	600597	A	C	0.00090	0.99910	0.00090	59.70	228.00	1.0000	35.74	44.28	0.0005	A
12	730343	chr12:600604	600604	G	T	0.00045	0.99955	0.00045	59.13	228.00	1.0000	30.19	31.16	0.0023	G
12	731617	chr12:601878	601878	A	T	0.00025	0.99975	0.00025	60.00	184.00	1.0000	18.00	18.00	0.0135	A
12	731618	chr12:601879	601879	G	T	0.00025	0.99975	0.00025	59.00	130.00	1.0000	13.00	13.00	0.0090	G
12	731649	chr12:601910	601910	A	G	0.00025	0.99975	0.00025	60.00	228.00	1.0000	43.00	43.00	0.0000	A
12	731689	chr12:601950	601950	A	G	0.00044	0.99956	0.00044	60.00	228.00	1.0000	55.00	55.00	0.0000	A
12	731692	chr12:601953	601953	G	C	0.00088	0.99912	0.00088	60.00	228.00	1.0000	83.18	89.83	0.0000	G
12	731694	chr12:601955	601955	C	T	0.00025	0.99975	0.00025	60.00	177.00	1.0000	77.00	77.00	0.0000	C
12	731705	chr12:601966	601966	A	C	0.00039	0.99961	0.00039	60.00	228.00	1.0000	62.00	62.00	0.0000	A
12	731751	chr12:602012	602012	G	A	0.00039	0.99961	0.00039	60.00	228.00	1.0000	68.00	68.00	0.0000	G
12	731812	chr12:602073	602073	T	C	0.00039	0.99961	0.00039	60.00	228.00	1.0000	55.00	55.00	0.0000	T
12	731816	chr12:602077	602077	C	T	0.00044	0.99956	0.00044	60.00	228.00	1.0000	63.00	63.00	0.0000	C
12	731818	chr12:602079	602079	C	T	0.00025	0.99975	0.00025	60.00	228.00	1.0000	85.00	85.00	0.0000	C
12	731872	chr12:602133	602133	A	G	0.00039	0.99961	0.00039	60.00	228.00	1.0000	41.00	41.00	0.0000	A
12	731876	chr12:602137	602137	C	T	0.00260	0.99740	0.00260	60.00	227.49	0.0220	22.18	53.58	0.0005	C
12	731883	chr12:602144	602144	G	C	0.00025	0.99975	0.00025	60.00	228.00	1.0000	30.00	30.00	0.0000	G
12	731886	chr12:602147	602147	T	A	0.04111	0.95889	0.04111	60.00	226.22	0.0721	23.48	62.13	0.0009	T
12	731895	chr12:602156	602156	G	A	0.00025	0.99975	0.00025	60.00	228.00	1.0000	52.00	52.00	0.0000	G
12	731981	chr12:602242	602242	A	G	0.00068	0.99932	0.00068	60.00	228.00	1.0000	27.36	33.03	0.0018	A
12	732011	chr12:602272	602272	C	T	0.00044	0.99956	0.00044	60.00	228.00	1.0000	61.00	61.00	0.0000	C
12	732057	chr12:602318	602318	G	A	0.00025	0.99975	0.00025	60.00	228.00	1.0000	46.00	46.00	0.0005	g
12	732200	chr12:602461	602461	A	G	0.00025	0.99975	0.00025	59.00	108.00	1.0000	13.00	13.00	0.0020	A
12	732202	chr12:602463	602463	G	A	0.00025	0.99975	0.00025	60.00	228.00	1.0000	38.00	38.00	0.0015	G
12	732259	chr12:602520	602520	C	A	0.00075	0.99925	0.00075	60.00	228.00	1.0000	28.65	54.30	0.0010	C
12	732343	chr12:602604	602604	G	T	0.00039	0.99961	0.00039	60.00	228.00	1.0000	59.00	59.00	0.0000	G
12	732505	chr12:602766	602766	C	A	0.01215	0.98785	0.01215	60.00	225.55	0.1145	14.39	52.84	0.0058	C
12	732520	chr12:602781	602781	A	C	0.16764	0.83236	0.16764	60.00	210.78	0.0143	12.68	48.79	0.0098	A

12	732524	chr12:602785	602785	C	T	0.00044	0.99956	0.00044	60.00	177.00	1.0000	15.00	15.00	0.0035	C
12	732527	chr12:602788	602788	C	A	0.00039	0.99961	0.00039	60.00	39.00	1.0000	15.00	15.00	0.0023	C
12	732565	chr12:602826	602826	G	C	0.00039	0.99961	0.00039	60.00	228.00	1.0000	41.00	41.00	0.0015	G
12	732788	chr12:603049	603049	G	A	0.00025	0.99975	0.00025	60.00	228.00	1.0000	78.00	78.00	0.0000	G
12	732854	chr12:603115	603115	A	T	0.00025	0.99975	0.00025	60.00	111.00	1.0000	22.00	22.00	0.0085	A
12	732859	chr12:603120	603120	C	T	0.10845	0.89155	0.10845	60.00	225.85	0.4857	31.94	78.13	0.0063	C
12	732896	chr12:603157	603157	T	C	0.00025	0.99975	0.00025	60.00	228.00	1.0000	69.00	69.00	0.0000	T
12	733247	chr12:603508	603508	A	G	0.00032	0.99968	0.00032	60.00	228.00	1.0000	52.19	52.19	0.0000	A
12	733252	chr12:603513	603513	T	A	0.00147	0.99853	0.00147	60.00	228.00	1.0000	50.03	68.48	0.0000	T
12	733289	chr12:603550	603550	C	T	0.00045	0.99955	0.00045	60.00	228.00	1.0000	62.95	73.33	0.0000	c
12	733318	chr12:603579	603579	A	G	0.00044	0.99956	0.00044	60.00	228.00	1.0000	64.00	64.00	0.0000	A
12	733335	chr12:603596	603596	T	G	0.00090	0.99910	0.00090	60.00	228.00	1.0000	60.45	74.61	0.0000	T
12	733346	chr12:603607	603607	G	C	0.00032	0.99968	0.00032	60.00	228.00	1.0000	50.42	50.42	0.0000	G
12	733354	chr12:603615	603615	A	G	0.99921	0.00079	0.00079	60.00	225.15	1.0000	41.67	91.10	0.0000	G
12	733408	chr12:603669	603669	A	G	0.07159	0.92841	0.07159	60.00	225.60	0.3142	30.21	77.34	0.0007	A
12	733410	chr12:603671	603671	G	T	0.00044	0.99956	0.00044	60.00	228.00	1.0000	66.00	66.00	0.0000	G
12	733448	chr12:603709	603709	T	C	0.00044	0.99956	0.00044	60.00	44.00	1.0000	10.00	10.00	0.0018	T
12	733470	chr12:603731	603731	T	A	0.00043	0.99957	0.00043	60.00	45.20	1.0000	16.03	16.03	0.0445	T
12	733531	chr12:603792	603792	T	C	0.00039	0.99961	0.00039	60.00	228.00	1.0000	66.00	66.00	0.0000	T
12	733541	chr12:603802	603802	G	A	0.00090	0.99910	0.00090	60.00	228.00	1.0000	60.46	67.68	0.0000	G
12	733583	chr12:603844	603844	G	A	0.00025	0.99975	0.00025	60.00	228.00	1.0000	74.00	74.00	0.0000	G
12	733672	chr12:603933	603933	C	T	0.00045	0.99955	0.00045	60.00	228.00	1.0000	42.79	45.01	0.0000	C
12	733674	chr12:603935	603935	T	C	0.00025	0.99975	0.00025	60.00	228.00	1.0000	46.00	46.00	0.0030	T
12	733688	chr12:603949	603949	T	C	0.00039	0.99961	0.00039	60.00	120.00	1.0000	27.00	27.00	0.0000	T
12	733696	chr12:603957	603957	C	T	0.00025	0.99975	0.00025	60.00	228.00	1.0000	17.00	17.00	0.0040	C
12	734426	chr12:604687	604687	G	A	0.00025	0.99975	0.00025	60.00	228.00	1.0000	63.00	63.00	0.0005	G
12	734453	chr12:604714	604714	G	T	0.00025	0.99975	0.00025	60.00	228.00	1.0000	46.00	46.00	0.0005	G
12	734480	chr12:604741	604741	G	A	0.07140	0.92860	0.07140	60.00	226.89	0.5759	34.85	81.76	0.0011	G
12	734500	chr12:604761	604761	A	G	0.00025	0.99975	0.00025	60.00	228.00	1.0000	77.00	77.00	0.0000	A
12	734520	chr12:604781	604781	G	A	0.00044	0.99956	0.00044	60.00	228.00	1.0000	63.00	63.00	0.0000	G
12	734581	chr12:604842	604842	G	T	0.00039	0.99961	0.00039	60.00	228.00	1.0000	75.00	75.00	0.0000	G
12	734660	chr12:604921	604921	G	A	0.01096	0.98904	0.01096	60.00	225.90	0.2208	33.81	67.17	0.0009	G
12	734679	chr12:604940	604940	T	C	0.00044	0.99956	0.00044	60.00	228.00	1.0000	74.00	74.00	0.0000	T
12	734786	chr12:605047	605047	T	A	0.00135	0.99865	0.00135	60.00	226.04	1.0000	31.49	55.15	0.0005	T

12	734816	chr12:605077	605077	C	T	0.00044	0.99956	0.00044	60.00	217.00	1.0000	35.00	35.00	0.0000	C
12	734826	chr12:605087	605087	C	T	0.00044	0.99956	0.00044	60.00	228.00	1.0000	55.00	55.00	0.0000	C
12	734910	chr12:605171	605171	T	G	0.00048	0.99952	0.00048	60.00	228.00	1.0000	41.86	44.89	0.0003	T
12	734950	chr12:605211	605211	T	C	0.00039	0.99961	0.00039	60.00	228.00	1.0000	51.00	51.00	0.0000	T
12	734965	chr12:605226	605226	T	A	0.00870	0.99130	0.00870	60.00	226.58	1.0000	18.27	55.91	0.0014	T
12	734979	chr12:605240	605240	C	T	0.00025	0.99975	0.00025	60.00	228.00	1.0000	28.00	28.00	0.0020	c
12	735003	chr12:605264	605264	G	A	0.00065	0.99935	0.00065	60.00	228.00	1.0000	19.77	22.81	0.0137	G
12	735046	chr12:605307	605307	G	A	0.14515	0.85485	0.14515	60.00	209.92	0.1897	13.85	49.01	0.0173	G
12	735147	chr12:605408	605408	G	A	0.00039	0.99961	0.00039	60.00	228.00	1.0000	55.00	55.00	0.0000	G
12	735160	chr12:605421	605421	G	C	0.00025	0.99975	0.00025	60.00	228.00	1.0000	55.00	55.00	0.0000	G
12	735175	chr12:605436	605436	T	C	0.00075	0.99925	0.00075	60.00	228.00	1.0000	32.05	71.00	0.0000	T
12	735201	chr12:605462	605462	G	A	0.00039	0.99961	0.00039	60.00	228.00	1.0000	74.00	74.00	0.0000	G
12	735303	chr12:605564	605564	G	A	0.00025	0.99975	0.00025	60.00	228.00	1.0000	78.00	78.00	0.0000	G
12	735306	chr12:605567	605567	C	T	0.00025	0.99975	0.00025	60.00	228.00	1.0000	70.00	70.00	0.0000	C
12	735323	chr12:605584	605584	G	C	0.00025	0.99975	0.00025	60.00	228.00	1.0000	42.00	42.00	0.0000	G
12	735417	chr12:605678	605678	A	G	0.00196	0.99804	0.00196	60.00	83.53	1.0000	17.20	29.73	0.0778	A
12	735421	chr12:605682	605682	G	A	0.00025	0.99975	0.00025	60.00	54.00	1.0000	14.00	14.00	0.0010	G
12	735460	chr12:605721	605721	T	C	0.00068	0.99932	0.00068	60.00	228.00	1.0000	56.09	64.42	0.0000	T
12	735495	chr12:605756	605756	C	G	0.00039	0.99961	0.00039	60.00	228.00	1.0000	34.00	34.00	0.0000	C
12	735499	chr12:605760	605760	G	T	0.00025	0.99975	0.00025	60.00	228.00	1.0000	61.00	61.00	0.0000	G
12	735504	chr12:605765	605765	T	C	0.99819	0.00181	0.00181	60.00	193.15	1.0000	31.03	79.65	0.0016	C
12	735525	chr12:605786	605786	G	C	0.00135	0.99865	0.00135	60.00	228.00	1.0000	58.02	71.28	0.0000	G
12	735535	chr12:605796	605796	A	G	0.00044	0.99956	0.00044	60.00	228.00	1.0000	52.00	52.00	0.0000	A
12	735603	chr12:605864	605864	T	C	0.00039	0.99961	0.00039	60.00	228.00	1.0000	70.00	70.00	0.0000	T
12	735621	chr12:605882	605882	C	G	0.00025	0.99975	0.00025	60.00	228.00	1.0000	57.00	57.00	0.0005	c
12	735768	chr12:606029	606029	G	A	0.10908	0.89092	0.10908	60.00	224.85	0.5748	25.73	74.46	0.0007	G
12	735881	chr12:606142	606142	A	C	0.99830	0.00170	0.00170	60.00	145.21	1.0000	20.24	59.48	0.0041	C
12	735981	chr12:606242	606242	G	A	0.00042	0.99958	0.00042	59.00	70.68	1.0000	20.78	20.78	0.0225	G
12	735989	chr12:606250	606250	C	A	0.06111	0.93889	0.06111	58.78	201.81	0.0725	10.00	28.66	0.0512	C
12	736102	chr12:606363	606363	G	T	0.97075	0.02925	0.02925	59.04	100.78	1.0000	11.00	42.00	0.0186	T
12	736110	chr12:606371	606371	G	A	0.00116	0.99884	0.00116	58.33	228.00	1.0000	21.20	31.65	0.0000	G
12	736116	chr12:606377	606377	T	C	0.14915	0.85085	0.14915	59.12	211.86	0.5914	14.29	46.93	0.0164	T
12	736156	chr12:606417	606417	C	T	0.00044	0.99956	0.00044	59.00	228.00	1.0000	36.00	36.00	0.0027	C
12	736172	chr12:606433	606433	G	T	0.00076	0.99924	0.00076	59.20	217.93	1.0000	24.17	31.04	0.0069	G

12	736204	chr12:606465	606465	T	C	0.00025	0.99975	0.00025	57.00	95.00	1.0000	13.00	13.00	0.0120 T
12	736209	chr12:606470	606470	C	T	0.00025	0.99975	0.00025	55.00	185.00	1.0000	11.00	11.00	0.0115 C
12	736233	chr12:606494	606494	A	G	0.00046	0.99954	0.00046	56.00	55.00	1.0000	10.00	10.00	0.0380 A
12	736235	chr12:606496	606496	A	G	0.00046	0.99954	0.00046	58.00	61.00	1.0000	10.00	10.00	0.0415 A
12	736367	chr12:606628	606628	A	C	0.00039	0.99961	0.00039	60.00	228.00	1.0000	83.00	83.00	0.0000 A
12	736378	chr12:606639	606639	T	C	0.07112	0.92888	0.07112	60.00	225.76	0.5813	29.40	77.45	0.0020 T
12	736434	chr12:606695	606695	T	A	0.00039	0.99961	0.00039	60.00	228.00	1.0000	33.00	33.00	0.0000 T
12	736439	chr12:606700	606700	G	A	0.00039	0.99961	0.00039	60.00	228.00	1.0000	56.00	56.00	0.0000 G
12	736714	chr12:606975	606975	G	A	0.81304	0.18696	0.18696	60.00	175.17	0.1634	23.36	65.58	0.0117 G
12	736752	chr12:607013	607013	T	C	0.00782	0.99218	0.00782	60.00	224.17	1.0000	19.80	59.42	0.0038 T
12	736757	chr12:607018	607018	G	A	0.00044	0.99956	0.00044	60.00	228.00	1.0000	34.00	34.00	0.0035 G
12	736766	chr12:607027	607027	G	A	0.00025	0.99975	0.00025	60.00	228.00	1.0000	42.00	42.00	0.0055 G
12	736777	chr12:607038	607038	G	A	0.00039	0.99961	0.00039	60.00	228.00	1.0000	44.00	44.00	0.0000 G
12	736792	chr12:607053	607053	G	T	0.00076	0.99924	0.00076	60.00	228.00	1.0000	29.07	45.76	0.0081 G
12	736811	chr12:607072	607072	A	C	0.00025	0.99975	0.00025	60.00	171.00	1.0000	29.00	29.00	0.0205 A
12	736814	chr12:607075	607075	G	C	0.00026	0.99974	0.00026	60.00	228.00	1.0000	36.00	36.00	0.0230 G
12	736851	chr12:607112	607112	C	G	0.00026	0.99974	0.00026	60.00	228.00	1.0000	22.00	22.00	0.0290 C
12	736859	chr12:607120	607120	G	A	0.10847	0.89153	0.10847	60.00	220.92	0.3994	15.43	62.53	0.0256 G
12	736878	chr12:607139	607139	C	T	0.00039	0.99961	0.00039	60.00	228.00	1.0000	37.00	37.00	0.0008 C
12	736882	chr12:607143	607143	G	C	0.00077	0.99923	0.00077	60.00	228.00	1.0000	33.25	42.75	0.0000 G
12	736890	chr12:607151	607151	A	C	0.00045	0.99955	0.00045	60.00	138.00	1.0000	13.00	13.00	0.0088 A
12	736917	chr12:607178	607178	G	A	0.00028	0.99972	0.00028	60.00	228.00	1.0000	14.00	14.00	0.1043 G
12	737161	chr12:607422	607422	T	C	0.00078	0.99922	0.00078	60.00	141.50	1.0000	11.40	26.60	0.0085 T
12	737186	chr12:607447	607447	C	T	0.00039	0.99961	0.00039	60.00	228.00	1.0000	22.00	22.00	0.0170 C
12	737196	chr12:607457	607457	T	G	0.00043	0.99957	0.00043	60.00	37.10	1.0000	13.61	13.61	0.0321 T
12	737230	chr12:607491	607491	G	A	0.18282	0.81718	0.18282	60.00	213.37	0.0260	13.61	55.40	0.0326 G
12	737269	chr12:607530	607530	G	A	0.00026	0.99974	0.00026	60.00	157.00	1.0000	11.00	11.00	0.0369 G
12	737281	chr12:607542	607542	T	C	0.00026	0.99974	0.00026	60.00	228.00	1.0000	32.00	32.00	0.0344 T
12	737307	chr12:607568	607568	A	G	0.00039	0.99961	0.00039	60.00	228.00	1.0000	39.00	39.00	0.0000 A
12	737355	chr12:607616	607616	A	G	0.00026	0.99974	0.00026	60.00	69.00	1.0000	16.00	16.00	0.0374 A
12	737394	chr12:607655	607655	C	A	0.00077	0.99923	0.00077	60.00	78.50	1.0000	10.23	18.78	0.0000 C
12	737430	chr12:607691	607691	C	G	0.00025	0.99975	0.00025	60.00	228.00	1.0000	34.00	34.00	0.0175 C
12	737471	chr12:607732	607732	G	C	0.00027	0.99973	0.00027	60.00	78.00	1.0000	12.00	12.00	0.0649 G
12	737511	chr12:607772	607772	C	G	0.00242	0.99758	0.00242	59.91	62.28	1.0000	12.60	18.87	0.0224 C

12	737521	chr12:607782	607782	A	G	0.00026	0.99974	0.00026	60.00	227.00	1.0000	22.00	22.00	0.0295	A
12	737534	chr12:607795	607795	C	T	0.00103	0.99897	0.00103	60.00	228.00	1.0000	34.10	58.46	0.0025	C
12	737545	chr12:607806	607806	G	A	0.00039	0.99961	0.00039	60.00	228.00	1.0000	42.00	42.00	0.0000	G
12	737557	chr12:607818	607818	C	A	0.00030	0.99970	0.00030	60.00	228.00	1.0000	31.98	31.98	0.0051	C
12	737600	chr12:607861	607861	C	T	0.00050	0.99950	0.00050	60.00	135.00	1.0000	37.00	37.00	0.0040	C
12	737612	chr12:607873	607873	A	G	0.00025	0.99975	0.00025	60.00	228.00	1.0000	48.00	48.00	0.0025	A
12	737620	chr12:607881	607881	C	T	0.00044	0.99956	0.00044	60.00	228.00	1.0000	54.00	54.00	0.0009	C
12	737695	chr12:607956	607956	T	C	0.00025	0.99975	0.00025	60.00	228.00	1.0000	35.00	35.00	0.0140	T
12	738034	chr12:608295	608295	G	T	0.00046	0.99954	0.00046	58.00	58.00	1.0000	10.00	10.00	0.0495	G
12	738035	chr12:608296	608296	G	C	0.00046	0.99954	0.00046	59.00	228.00	1.0000	17.00	17.00	0.0477	G
12	738226	chr12:608487	608487	C	T	0.00025	0.99975	0.00025	60.00	228.00	1.0000	32.00	32.00	0.0060	C
12	738247	chr12:608508	608508	G	A	0.00056	0.99944	0.00056	60.00	228.00	1.0000	49.32	57.66	0.0011	G
12	738279	chr12:608540	608540	T	G	0.00068	0.99932	0.00068	59.85	67.89	1.0000	14.15	18.41	0.0094	T
12	738280	chr12:608541	608541	T	G	0.00025	0.99975	0.00025	60.00	140.00	1.0000	23.00	23.00	0.0160	T
12	738283	chr12:608544	608544	T	C	0.00044	0.99956	0.00044	60.00	228.00	1.0000	45.00	45.00	0.0018	T
12	738295	chr12:608556	608556	G	A	0.00026	0.99974	0.00026	59.00	228.00	1.0000	15.00	15.00	0.0260	G
12	738307	chr12:608568	608568	C	T	0.01078	0.98922	0.01078	59.05	79.95	1.0000	10.81	26.64	0.0457	C
12	738314	chr12:608575	608575	T	C	0.00026	0.99974	0.00026	60.00	228.00	1.0000	17.00	17.00	0.0369	T
12	738345	chr12:608606	608606	C	A	0.00044	0.99956	0.00044	59.00	228.00	1.0000	29.00	29.00	0.0071	C
12	738347	chr12:608608	608608	C	T	0.00039	0.99961	0.00039	60.00	228.00	1.0000	45.00	45.00	0.0000	C
12	738413	chr12:608674	608674	A	G	0.00026	0.99974	0.00026	60.00	108.00	1.0000	14.00	14.00	0.0295	A
12	738421	chr12:608682	608682	T	C	0.00044	0.99956	0.00044	60.00	228.00	1.0000	35.00	35.00	0.0027	T
12	738445	chr12:608706	608706	A	G	0.00025	0.99975	0.00025	60.00	228.00	1.0000	35.00	35.00	0.0180	A
12	738450	chr12:608711	608711	C	G	0.17169	0.82831	0.17169	60.00	220.14	0.0646	21.67	74.37	0.0121	G
12	738468	chr12:608729	608729	G	A	0.00025	0.99975	0.00025	60.00	228.00	1.0000	41.00	41.00	0.0095	C
12	738484	chr12:608745	608745	C	T	0.00044	0.99956	0.00044	60.00	228.00	1.0000	39.00	39.00	0.0018	C
12	738513	chr12:608774	608774	G	A	0.00039	0.99961	0.00039	60.00	228.00	1.0000	68.00	68.00	0.0000	G
12	738555	chr12:608816	608816	C	T	0.00027	0.99973	0.00027	60.00	227.00	1.0000	15.00	15.00	0.0829	C
12	738556	chr12:608817	608817	C	A	0.00045	0.99955	0.00045	60.00	103.00	1.0000	29.00	29.00	0.0106	C
12	738580	chr12:608841	608841	A	G	0.00048	0.99952	0.00048	60.00	66.00	1.0000	13.00	13.00	0.0804	A
12	738657	chr12:608918	608918	T	C	0.09572	0.90428	0.09572	60.00	215.14	0.3616	15.14	52.12	0.0442	T
12	738667	chr12:608928	608928	G	T	0.00045	0.99955	0.00045	60.00	228.00	1.0000	20.00	20.00	0.0212	G
12	738692	chr12:608953	608953	T	G	0.00039	0.99961	0.00039	59.00	34.00	1.0000	16.00	16.00	0.0139	T
12	739150	chr12:609411	609411	T	C	0.00039	0.99961	0.00039	57.00	228.00	1.0000	20.00	20.00	0.0000	T

12	739332	chr12:609593	609593	A	T	0.07164	0.92836	0.07164	60.00	225.92	0.3142	28.12	73.28	0.0045	A
12	739363	chr12:609624	609624	C	T	0.00039	0.99961	0.00039	60.00	228.00	1.0000	32.00	32.00	0.0000	C
12	739375	chr12:609636	609636	A	T	0.00039	0.99961	0.00039	60.00	228.00	1.0000	40.00	40.00	0.0000	A
12	739438	chr12:609699	609699	C	A	0.00077	0.99923	0.00077	60.00	228.00	1.0000	71.08	73.93	0.0000	C
12	739464	chr12:609725	609725	G	A	0.00039	0.99961	0.00039	60.00	228.00	1.0000	53.00	53.00	0.0000	G
12	739480	chr12:609741	609741	T	C	0.00044	0.99956	0.00044	60.00	228.00	1.0000	40.00	40.00	0.0000	T
12	739503	chr12:609764	609764	T	C	0.00026	0.99974	0.00026	59.00	84.00	1.0000	15.00	15.00	0.0414	-
12	740009	chr12:610270	610270	C	G	0.00415	0.99585	0.00415	60.00	111.40	0.0000	10.50	24.60	0.1628	G
12	740066	chr12:610327	610327	C	T	0.00026	0.99974	0.00026	60.00	228.00	1.0000	36.00	36.00	0.0344	C
12	740137	chr12:610398	610398	A	G	0.00044	0.99956	0.00044	60.00	228.00	1.0000	29.00	29.00	0.0000	A
12	740191	chr12:610452	610452	G	A	0.00025	0.99975	0.00025	60.00	102.00	1.0000	14.00	14.00	0.0010	G
12	740240	chr12:610501	610501	C	T	0.01785	0.98215	0.01785	60.00	226.83	1.0000	19.40	67.16	0.0074	C
12	740268	chr12:610529	610529	G	A	0.00039	0.99961	0.00039	60.00	228.00	1.0000	68.00	68.00	0.0000	G
12	740293	chr12:610554	610554	C	G	0.00497	0.99503	0.00497	60.00	225.56	1.0000	20.07	51.27	0.0002	C
12	740298	chr12:610559	610559	C	T	0.00260	0.99740	0.00260	60.00	220.97	1.0000	30.45	44.48	0.0007	C
12	740307	chr12:610568	610568	G	T	0.00032	0.99968	0.00032	60.00	55.30	1.0000	24.99	24.99	0.0438	G
12	740315	chr12:610576	610576	T	C	0.00032	0.99968	0.00032	60.00	228.00	1.0000	41.17	41.17	0.0006	T
12	740442	chr12:610703	610703	A	G	0.00050	0.99950	0.00050	60.00	228.00	1.0000	39.08	41.93	0.0010	A
12	740450	chr12:610711	610711	G	A	0.00056	0.99944	0.00056	60.00	228.00	1.0000	41.75	45.39	0.0005	G
12	740499	chr12:610760	610760	C	G	0.00041	0.99959	0.00041	60.00	228.00	1.0000	32.80	32.80	0.0000	C
12	740502	chr12:610763	610763	C	T	0.00044	0.99956	0.00044	60.00	226.00	1.0000	17.00	17.00	0.0000	C
12	740856	chr12:611117	611117	A	G	0.00371	0.99629	0.00371	60.00	84.46	1.0000	16.73	27.87	0.0550	A
12	740878	chr12:611139	611139	C	T	0.00169	0.99831	0.00169	60.00	228.00	1.0000	54.67	87.43	0.0000	C
12	740897	chr12:611158	611158	G	A	0.00025	0.99975	0.00025	60.00	228.00	1.0000	72.00	72.00	0.0000	G
12	740905	chr12:611166	611166	T	C	0.00025	0.99975	0.00025	60.00	220.00	1.0000	41.00	41.00	0.0000	T
12	740915	chr12:611176	611176	C	T	0.00044	0.99956	0.00044	60.00	228.00	1.0000	93.00	93.00	0.0000	C
12	740938	chr12:611199	611199	C	T	0.33111	0.66889	0.33111	60.00	223.57	0.0172	35.50	82.98	0.0025	C
12	740949	chr12:611210	611210	T	A	0.00039	0.99961	0.00039	60.00	228.00	1.0000	59.00	59.00	0.0000	T
12	740961	chr12:611222	611222	G	A	0.33114	0.66886	0.33114	60.00	215.52	0.0142	25.20	67.31	0.0043	G
12	740964	chr12:611225	611225	G	A	0.00044	0.99956	0.00044	60.00	228.00	1.0000	31.00	31.00	0.0000	G
12	740971	chr12:611232	611232	A	C	0.00025	0.99975	0.00025	60.00	228.00	1.0000	30.00	30.00	0.0010	A
12	740984	chr12:611245	611245	T	G	0.00053	0.99947	0.00053	60.00	54.00	1.0000	11.08	13.93	0.0599	T
12	741058	chr12:611319	611319	C	T	0.00068	0.99932	0.00068	60.00	228.00	1.0000	32.57	58.72	0.0002	C
12	741062	chr12:611323	611323	G	A	0.05439	0.94561	0.05439	60.00	225.71	0.1042	23.47	66.64	0.0020	G

12	741079	chr12:611340	611340	C	A	0.00039	0.99961	0.00039	60.00	228.00	1.0000	38.00	38.00	0.0000	C
12	741219	chr12:611480	611480	C	G	0.10172	0.89828	0.10172	60.00	226.80	0.0797	37.16	88.61	0.0014	C
12	741304	chr12:611565	611565	G	A	0.00835	0.99165	0.00835	60.00	226.69	1.0000	35.49	82.98	0.0002	G
12	741317	chr12:611578	611578	C	T	0.00372	0.99628	0.00372	60.00	228.00	1.0000	37.13	81.08	0.0000	C
12	741366	chr12:611627	611627	C	A	0.00106	0.99894	0.00106	60.00	228.00	1.0000	50.52	76.63	0.0003	C
12	741429	chr12:611690	611690	G	A	0.00025	0.99975	0.00025	60.00	228.00	1.0000	69.00	69.00	0.0005	G
12	741460	chr12:611721	611721	A	G	0.00039	0.99961	0.00039	60.00	228.00	1.0000	51.00	51.00	0.0000	A
12	741476	chr12:611737	611737	T	C	0.00056	0.99944	0.00056	60.00	228.00	1.0000	35.51	41.82	0.0007	T
12	741500	chr12:611761	611761	C	A	0.00039	0.99961	0.00039	60.00	228.00	1.0000	59.00	59.00	0.0000	C
12	741507	chr12:611768	611768	C	T	0.00044	0.99956	0.00044	60.00	228.00	1.0000	25.00	25.00	0.0000	C
12	741508	chr12:611769	611769	G	C	0.00045	0.99955	0.00045	60.00	228.00	1.0000	44.62	46.29	0.0007	g
12	741557	chr12:611818	611818	A	G	0.00044	0.99956	0.00044	60.00	228.00	1.0000	23.00	23.00	0.0000	A
12	741638	chr12:611899	611899	T	G	0.00044	0.99956	0.00044	60.00	228.00	1.0000	50.00	50.00	0.0000	T
12	741643	chr12:611904	611904	A	G	0.00044	0.99956	0.00044	60.00	228.00	1.0000	79.00	79.00	0.0000	A
12	741646	chr12:611907	611907	G	T	0.00050	0.99950	0.00050	60.00	228.00	1.0000	42.53	62.48	0.0010	G
12	741677	chr12:611938	611938	C	T	0.00025	0.99975	0.00025	60.00	228.00	1.0000	52.00	52.00	0.0005	C
12	741773	chr12:612034	612034	T	C	0.00102	0.99898	0.00102	60.00	228.00	1.0000	59.96	71.76	0.0002	T
12	741825	chr12:612086	612086	A	C	0.00044	0.99956	0.00044	60.00	228.00	1.0000	59.00	59.00	0.0000	A
12	741885	chr12:612146	612146	G	C	0.00039	0.99961	0.00039	60.00	228.00	1.0000	57.00	57.00	0.0000	G
12	741952	chr12:612213	612213	C	T	0.00025	0.99975	0.00025	60.00	228.00	1.0000	67.00	67.00	0.0010	C
12	742009	chr12:612270	612270	G	C	0.00079	0.99921	0.00079	60.00	228.00	1.0000	58.94	66.90	0.0005	G
12	742022	chr12:612283	612283	T	C	0.00044	0.99956	0.00044	60.00	228.00	1.0000	84.00	84.00	0.0000	T
12	742026	chr12:612287	612287	G	C	0.00050	0.99950	0.00050	60.00	228.00	1.0000	54.58	76.43	0.0010	G
12	742043	chr12:612304	612304	G	A	0.00077	0.99923	0.00077	60.00	228.00	1.0000	65.38	79.63	0.0000	G
12	742079	chr12:612340	612340	C	T	0.00025	0.99975	0.00025	60.00	228.00	1.0000	94.00	94.00	0.0005	C
12	742080	chr12:612341	612341	A	C	0.00025	0.99975	0.00025	60.00	228.00	1.0000	67.00	67.00	0.0005	A
12	742095	chr12:612356	612356	G	A	0.00039	0.99961	0.00039	60.00	228.00	1.0000	57.00	57.00	0.0000	G
12	742109	chr12:612370	612370	C	T	0.00044	0.99956	0.00044	60.00	228.00	1.0000	54.00	54.00	0.0000	C
12	742123	chr12:612384	612384	G	A	0.00373	0.99627	0.00373	60.00	228.00	1.0000	39.35	84.33	0.0005	G
12	742254	chr12:612515	612515	C	G	0.00077	0.99923	0.00077	60.00	228.00	1.0000	56.53	76.48	0.0000	C
12	742269	chr12:612530	612530	C	T	0.00044	0.99956	0.00044	60.00	123.00	1.0000	31.00	31.00	0.0027	C
12	742310	chr12:612571	612571	A	C	0.00039	0.99961	0.00039	60.00	228.00	1.0000	50.00	50.00	0.0000	A
12	742324	chr12:612585	612585	C	G	0.38925	0.61075	0.38925	60.00	226.41	0.0146	40.26	87.91	0.0025	C
12	743067	chr12:613328	613328	C	T	0.00025	0.99975	0.00025	60.00	228.00	1.0000	59.00	59.00	0.0000	C

12	743125	chr12:613386	613386	A	T	0.00039	0.99961	0.00039	60.00	32.00	1.0000	34.00	34.00	0.0000	A
12	743169	chr12:613430	613430	T	C	0.07056	0.92944	0.07056	60.00	226.88	0.5713	40.50	89.24	0.0005	T
12	743175	chr12:613436	613436	A	G	0.00032	0.99968	0.00032	60.00	228.00	1.0000	79.39	79.39	0.0000	A
12	743239	chr12:613500	613500	A	C	0.00050	0.99950	0.00050	60.00	228.00	1.0000	66.33	78.68	0.0005	A
12	743240	chr12:613501	613501	C	T	0.00044	0.99956	0.00044	60.00	228.00	1.0000	50.00	50.00	0.0000	C
12	743264	chr12:613525	613525	C	T	0.00075	0.99925	0.00075	60.00	228.00	1.0000	47.80	83.90	0.0000	C
12	743278	chr12:613539	613539	A	G	0.00044	0.99956	0.00044	60.00	228.00	1.0000	57.00	57.00	0.0000	A
12	743326	chr12:613587	613587	C	T	0.00044	0.99956	0.00044	60.00	228.00	1.0000	66.00	66.00	0.0000	C
12	743397	chr12:613658	613658	G	C	0.00028	0.99972	0.00028	60.00	228.00	1.0000	12.00	12.00	0.1103	G
12	743744	chr12:614005	614005	T	C	0.00041	0.99959	0.00041	60.00	228.00	1.0000	31.60	31.60	0.0000	T
12	743783	chr12:614044	614044	C	T	0.00025	0.99975	0.00025	60.00	228.00	1.0000	66.00	66.00	0.0005	C
12	743822	chr12:614083	614083	A	C	0.00025	0.99975	0.00025	60.00	228.00	1.0000	45.00	45.00	0.0000	A
12	743832	chr12:614093	614093	T	C	0.00048	0.99952	0.00048	60.00	228.00	1.0000	49.81	59.41	0.0000	T
12	743844	chr12:614105	614105	G	A	0.00048	0.99952	0.00048	60.00	228.00	1.0000	70.22	77.42	0.0000	G
12	743899	chr12:614160	614160	C	T	0.00102	0.99898	0.00102	60.00	207.58	1.0000	18.44	31.79	0.0005	C
12	743919	chr12:614180	614180	A	G	0.00147	0.99853	0.00147	60.00	228.00	1.0000	40.10	64.96	0.0002	A
12	743927	chr12:614188	614188	A	G	0.15654	0.84346	0.15654	60.00	223.14	0.6260	29.81	76.08	0.0009	A
12	744075	chr12:614336	614336	A	G	0.00025	0.99975	0.00025	60.00	228.00	1.0000	55.00	55.00	0.0000	A
12	744129	chr12:614390	614390	A	G	0.05202	0.94798	0.05202	59.95	213.31	0.5247	15.15	40.85	0.1218	A
12	744528	chr12:614789	614789	C	G	0.00039	0.99961	0.00039	60.00	228.00	1.0000	56.00	56.00	0.0000	C
12	744622	chr12:614883	614883	G	T	0.00044	0.99956	0.00044	60.00	228.00	1.0000	48.00	48.00	0.0000	G
12	744624	chr12:614885	614885	T	C	0.00044	0.99956	0.00044	60.00	58.00	1.0000	19.00	19.00	0.0000	T
12	744693	chr12:614954	614954	A	T	0.00025	0.99975	0.00025	58.00	228.00	1.0000	67.00	67.00	0.0000	A
12	744718	chr12:614979	614979	C	T	0.00068	0.99932	0.00068	58.19	184.50	0.0022	29.93	36.82	0.0020	C
12	744723	chr12:614984	614984	C	A	0.00025	0.99975	0.00025	58.00	228.00	1.0000	39.00	39.00	0.0000	C
12	744821	chr12:615082	615082	T	C	0.00039	0.99961	0.00039	60.00	228.00	1.0000	67.00	67.00	0.0000	T
12	744843	chr12:615104	615104	G	A	0.00025	0.99975	0.00025	60.00	228.00	1.0000	74.00	74.00	0.0000	G
12	744887	chr12:615148	615148	A	G	0.02611	0.97389	0.02611	59.99	226.63	0.2147	25.98	57.86	0.0061	A
12	744916	chr12:615177	615177	C	T	0.00044	0.99956	0.00044	60.00	228.00	1.0000	47.00	47.00	0.0027	C
12	744925	chr12:615186	615186	C	T	0.15510	0.84490	0.15510	60.00	219.43	0.4484	22.19	58.81	0.0129	C
12	744934	chr12:615195	615195	G	A	0.00039	0.99961	0.00039	60.00	228.00	1.0000	39.00	39.00	0.0000	G
12	745696	chr12:615957	615957	C	T	0.00050	0.99950	0.00050	60.00	228.00	1.0000	31.18	37.83	0.0005	C
12	745697	chr12:615958	615958	G	A	0.09646	0.90354	0.09646	59.88	217.88	0.0658	17.68	55.88	0.0056	G
12	745764	chr12:616025	616025	A	C	0.00330	0.99670	0.00330	59.82	228.00	1.0000	22.37	44.13	0.0082	A

12	745819	chr12:616080	616080	C	T	0.00058	0.99942	0.00058	59.88	228.00	1.0000	24.01	24.46	0.0262	C
12	745874	chr12:616135	616135	T	C	0.00030	0.99970	0.00030	60.00	228.00	1.0000	19.53	19.53	0.0012	T
12	745909	chr12:616170	616170	G	A	0.00025	0.99975	0.00025	60.00	219.00	1.0000	18.00	18.00	0.0030	G
12	745962	chr12:616223	616223	G	A	0.00088	0.99912	0.00088	60.00	228.00	1.0000	28.03	28.98	0.0018	G
12	745979	chr12:616240	616240	C	T	0.00044	0.99956	0.00044	59.00	228.00	1.0000	26.00	26.00	0.0009	C
12	746059	chr12:616320	616320	G	A	0.00039	0.99961	0.00039	60.00	228.00	1.0000	72.00	72.00	0.0000	G
12	746076	chr12:616337	616337	G	A	0.00025	0.99975	0.00025	60.00	228.00	1.0000	78.00	78.00	0.0005	G
12	746106	chr12:616367	616367	A	G	0.00025	0.99975	0.00025	60.00	228.00	1.0000	68.00	68.00	0.0005	A
12	746131	chr12:616392	616392	G	A	0.00044	0.99956	0.00044	60.00	228.00	1.0000	48.00	48.00	0.0000	g
12	746158	chr12:616419	616419	C	A	0.00039	0.99961	0.00039	60.00	228.00	1.0000	44.00	44.00	0.0000	C
12	746882	chr12:617143	617143	C	T	0.00025	0.99975	0.00025	60.00	228.00	1.0000	43.00	43.00	0.0005	C
12	746929	chr12:617190	617190	C	T	0.00361	0.99639	0.00361	60.00	227.15	0.0187	45.07	92.61	0.0002	C
12	746961	chr12:617222	617222	G	T	0.00039	0.99961	0.00039	60.00	228.00	1.0000	81.00	81.00	0.0000	G
12	746976	chr12:617237	617237	G	C	0.00025	0.99975	0.00025	60.00	228.00	1.0000	48.00	48.00	0.0000	G
12	747038	chr12:617299	617299	C	T	0.00025	0.99975	0.00025	60.00	228.00	1.0000	45.00	45.00	0.0005	T
12	747063	chr12:617324	617324	C	T	0.00044	0.99956	0.00044	60.00	228.00	1.0000	50.00	50.00	0.0000	C
12	747100	chr12:617361	617361	G	A	0.00025	0.99975	0.00025	60.00	228.00	1.0000	26.00	26.00	0.0010	G
12	747115	chr12:617376	617376	A	G	0.00025	0.99975	0.00025	60.00	228.00	1.0000	49.00	49.00	0.0020	A
12	747134	chr12:617395	617395	C	T	0.00039	0.99961	0.00039	60.00	228.00	1.0000	64.00	64.00	0.0000	C
12	747137	chr12:617398	617398	G	T	0.00025	0.99975	0.00025	60.00	228.00	1.0000	43.00	43.00	0.0015	G
12	747139	chr12:617400	617400	T	C	0.00039	0.99961	0.00039	60.00	228.00	1.0000	66.00	66.00	0.0000	T
12	747185	chr12:617446	617446	C	T	0.00025	0.99975	0.00025	60.00	131.00	1.0000	23.00	23.00	0.0025	C
12	747203	chr12:617464	617464	T	C	0.00025	0.99975	0.00025	60.00	128.00	1.0000	20.00	20.00	0.0025	T
12	747270	chr12:617531	617531	G	A	0.00025	0.99975	0.00025	60.00	228.00	1.0000	89.00	89.00	0.0015	G
12	747296	chr12:617557	617557	G	A	0.00025	0.99975	0.00025	60.00	228.00	1.0000	54.00	54.00	0.0015	G
12	747304	chr12:617565	617565	C	T	0.00025	0.99975	0.00025	60.00	228.00	1.0000	70.00	70.00	0.0005	C
12	747312	chr12:617573	617573	C	T	0.00050	0.99950	0.00050	60.00	228.00	1.0000	50.03	50.98	0.0015	C
12	747318	chr12:617579	617579	G	C	0.00025	0.99975	0.00025	60.00	89.00	1.0000	26.00	26.00	0.0020	G
12	747349	chr12:617610	617610	C	A	0.00088	0.99912	0.00088	60.00	228.00	1.0000	35.13	39.88	0.0000	C
12	747350	chr12:617611	617611	C	T	0.00025	0.99975	0.00025	60.00	228.00	1.0000	81.00	81.00	0.0015	C
12	747399	chr12:617660	617660	C	T	0.00025	0.99975	0.00025	60.00	228.00	1.0000	94.00	94.00	0.0000	C
12	747561	chr12:617822	617822	A	T	0.00039	0.99961	0.00039	60.00	228.00	1.0000	55.00	55.00	0.0000	A
12	747575	chr12:617836	617836	A	G	0.00025	0.99975	0.00025	60.00	228.00	1.0000	20.00	20.00	0.0005	A
12	747577	chr12:617838	617838	T	C	0.00025	0.99975	0.00025	60.00	228.00	1.0000	17.00	17.00	0.0035	T

12	747872	chr12:618133	618133	C	T	0.00040	0.99960	0.00040	59.00	202.00	1.0000	14.00	14.00	0.0301 C
12	747877	chr12:618138	618138	G	A	0.00053	0.99947	0.00053	59.00	130.00	1.0000	11.00	11.00	0.1723 G
12	747925	chr12:618186	618186	T	C	0.00497	0.99503	0.00497	60.00	226.99	1.0000	27.20	59.42	0.0011 T
12	747947	chr12:618208	618208	T	C	0.00044	0.99956	0.00044	60.00	228.00	1.0000	41.00	41.00	0.0000 T
12	747997	chr12:618258	618258	G	A	0.14620	0.85380	0.14620	60.00	218.89	0.2377	22.68	65.49	0.0219 G
12	748003	chr12:618264	618264	C	T	0.00044	0.99956	0.00044	60.00	228.00	1.0000	46.00	46.00	0.0018 C
12	748008	chr12:618269	618269	C	T	0.00025	0.99975	0.00025	60.00	228.00	1.0000	34.00	34.00	0.0015 C
12	748020	chr12:618281	618281	T	C	0.00045	0.99955	0.00045	60.00	228.00	1.0000	46.29	50.18	0.0005 T
12	748073	chr12:618334	618334	T	C	0.00124	0.99876	0.00124	60.00	228.00	1.0000	40.80	59.39	0.0005 T
12	748098	chr12:618359	618359	G	C	0.00044	0.99956	0.00044	60.00	228.00	1.0000	36.00	36.00	0.0000 G
12	748128	chr12:618389	618389	T	A	0.00039	0.99961	0.00039	60.00	228.00	1.0000	64.00	64.00	0.0000 A
12	748180	chr12:618441	618441	T	G	0.00025	0.99975	0.00025	60.00	228.00	1.0000	56.00	56.00	0.0045 T
12	748184	chr12:618445	618445	T	C	0.00044	0.99956	0.00044	60.00	228.00	1.0000	59.00	59.00	0.0009 T
12	748190	chr12:618451	618451	T	G	0.00025	0.99975	0.00025	60.00	228.00	1.0000	36.00	36.00	0.0050 T
12	748236	chr12:618497	618497	A	T	0.00050	0.99950	0.00050	60.00	60.00	1.0000	13.20	20.80	0.0085 A
12	748262	chr12:618523	618523	G	T	0.00331	0.99669	0.00331	60.00	226.12	1.0000	18.65	31.98	0.0112 G
12	748277	chr12:618538	618538	T	C	0.00039	0.99961	0.00039	60.00	228.00	1.0000	47.00	47.00	0.0000 T
12	748307	chr12:618568	618568	G	A	0.00044	0.99956	0.00044	60.00	228.00	1.0000	39.00	39.00	0.0000 G
12	748352	chr12:618613	618613	T	A	0.00373	0.99627	0.00373	60.00	228.00	1.0000	33.01	68.96	0.0009 T
12	748391	chr12:618652	618652	A	G	0.00050	0.99950	0.00050	60.00	228.00	1.0000	48.15	53.85	0.0030 A
12	748414	chr12:618675	618675	T	C	0.00170	0.99830	0.00170	60.00	226.06	1.0000	20.64	52.98	0.0025 T
12	748427	chr12:618688	618688	A	C	0.00025	0.99975	0.00025	60.00	160.00	1.0000	12.00	12.00	0.0030 A
12	748431	chr12:618692	618692	C	A	0.98508	0.01492	0.01492	60.00	116.57	0.0001	15.83	47.26	0.0061 A
12	748463	chr12:618724	618724	A	T	0.00044	0.99956	0.00044	60.00	205.00	1.0000	27.00	27.00	0.0000 A
12	748468	chr12:618729	618729	G	A	0.00039	0.99961	0.00039	60.00	228.00	1.0000	71.00	71.00	0.0000 G
12	748504	chr12:618765	618765	G	T	0.00025	0.99975	0.00025	59.00	142.00	1.0000	17.00	17.00	0.0050 G
12	748505	chr12:618766	618766	T	C	0.00025	0.99975	0.00025	60.00	126.00	1.0000	25.00	25.00	0.0065 T
12	748524	chr12:618785	618785	A	G	0.00025	0.99975	0.00025	59.00	199.00	1.0000	13.00	13.00	0.0100 A
12	748571	chr12:618832	618832	G	T	0.00044	0.99956	0.00044	60.00	228.00	1.0000	32.00	32.00	0.0009 G
12	748585	chr12:618846	618846	G	A	0.07086	0.92914	0.07086	60.00	224.10	0.1281	21.52	64.15	0.0047 G
12	748660	chr12:618921	618921	A	C	0.00025	0.99975	0.00025	60.00	53.00	1.0000	23.00	23.00	0.0005 A
12	748684	chr12:618945	618945	A	G	0.00025	0.99975	0.00025	60.00	228.00	1.0000	30.00	30.00	0.0010 -
12	748725	chr12:618986	618986	A	G	0.00025	0.99975	0.00025	60.00	184.00	1.0000	24.00	24.00	0.0010 A
12	748729	chr12:618990	618990	A	T	0.00075	0.99925	0.00075	60.00	228.00	1.0000	22.10	37.30	0.0005 A

12	748740	chr12:619001	619001	C	T	0.00045	0.99955	0.00045	60.00	228.00	1.0000	39.25	47.27	0.0016	C
12	748745	chr12:619006	619006	C	A	0.00025	0.99975	0.00025	60.00	157.00	1.0000	29.00	29.00	0.0005	C
12	748759	chr12:619020	619020	C	T	0.00044	0.99956	0.00044	60.00	228.00	1.0000	48.00	48.00	0.0000	C
12	748777	chr12:619038	619038	T	A	0.00044	0.99956	0.00044	60.00	228.00	1.0000	59.00	59.00	0.0000	T
12	748793	chr12:619054	619054	G	A	0.00206	0.99794	0.00206	60.00	228.00	1.0000	41.40	71.66	0.0000	G
12	748807	chr12:619068	619068	C	T	0.00025	0.99975	0.00025	60.00	228.00	1.0000	60.00	60.00	0.0010	C
12	748892	chr12:619153	619153	C	T	0.00079	0.99921	0.00079	60.00	228.00	1.0000	45.66	65.04	0.0005	C
12	748914	chr12:619175	619175	C	T	0.00044	0.99956	0.00044	60.00	228.00	1.0000	15.00	15.00	0.0044	C
12	748915	chr12:619176	619176	G	A	0.00316	0.99684	0.00316	60.00	228.00	1.0000	28.38	63.76	0.0016	G
12	748970	chr12:619231	619231	C	T	0.00039	0.99961	0.00039	60.00	228.00	1.0000	43.00	43.00	0.0000	C
12	748997	chr12:619258	619258	C	T	0.00090	0.99910	0.00090	60.00	217.82	1.0000	25.56	48.72	0.0023	C
12	749012	chr12:619273	619273	C	T	0.00147	0.99853	0.00147	60.00	224.37	1.0000	31.85	58.17	0.0020	C
12	749025	chr12:619286	619286	C	A	0.00025	0.99975	0.00025	60.00	228.00	1.0000	39.00	39.00	0.0020	C
12	749027	chr12:619288	619288	C	T	0.00032	0.99968	0.00032	60.00	228.00	1.0000	32.70	32.70	0.0026	C
12	749041	chr12:619302	619302	G	A	0.00057	0.99943	0.00057	60.00	212.68	1.0000	25.89	39.62	0.0018	G
12	749159	chr12:619420	619420	C	A	0.00025	0.99975	0.00025	60.00	228.00	1.0000	42.00	42.00	0.0005	C
12	749164	chr12:619425	619425	G	A	0.00025	0.99975	0.00025	60.00	228.00	1.0000	26.00	26.00	0.0005	G
12	749325	chr12:619586	619586	C	T	0.00039	0.99961	0.00039	60.00	228.00	1.0000	59.00	59.00	0.0000	C
12	749391	chr12:619652	619652	G	T	0.00068	0.99932	0.00068	60.00	228.00	1.0000	35.01	51.30	0.0009	G
12	749400	chr12:619661	619661	T	C	0.00077	0.99923	0.00077	60.00	228.00	1.0000	57.48	75.53	0.0000	T
12	749527	chr12:619788	619788	C	A	0.00039	0.99961	0.00039	60.00	228.00	1.0000	69.00	69.00	0.0000	C
12	749528	chr12:619789	619789	G	A	0.00025	0.99975	0.00025	60.00	142.00	1.0000	25.00	25.00	0.0000	G
12	749596	chr12:619857	619857	T	C	0.00025	0.99975	0.00025	60.00	228.00	1.0000	31.00	31.00	0.0000	T
12	749633	chr12:619894	619894	T	C	0.00032	0.99968	0.00032	60.00	228.00	1.0000	68.94	68.94	0.0000	T
12	749670	chr12:619931	619931	C	T	0.00039	0.99961	0.00039	60.00	228.00	1.0000	46.00	46.00	0.0000	C
12	750023	chr12:620284	620284	T	C	0.00039	0.99961	0.00039	60.00	228.00	1.0000	49.00	49.00	0.0000	T
12	750067	chr12:620328	620328	C	G	0.00039	0.99961	0.00039	60.00	228.00	1.0000	67.00	67.00	0.0000	C
12	750182	chr12:620443	620443	G	A	0.00025	0.99975	0.00025	60.00	228.00	1.0000	66.00	66.00	0.0015	G
12	750214	chr12:620475	620475	C	T	0.00039	0.99961	0.00039	60.00	228.00	1.0000	76.00	76.00	0.0000	C
12	750241	chr12:620502	620502	T	C	0.00025	0.99975	0.00025	60.00	228.00	1.0000	44.00	44.00	0.0005	T
12	750296	chr12:620557	620557	A	G	0.00047	0.99953	0.00047	60.00	66.00	1.0000	10.00	10.00	0.0512	A
12	750337	chr12:620598	620598	C	T	0.00025	0.99975	0.00025	60.00	160.00	1.0000	22.00	22.00	0.0065	C
12	750376	chr12:620637	620637	C	T	0.00026	0.99974	0.00026	60.00	228.00	1.0000	31.00	31.00	0.0215	C
12	750401	chr12:620662	620662	A	G	0.48532	0.51468	0.48532	60.00	184.01	0.2455	13.00	45.00	0.0015	A

12	750406	chr12:620667	620667	C	T	0.00028	0.99972	0.00028	60.00	228.00	1.0000	24.00	24.00	0.1193	C
12	750409	chr12:620670	620670	A	T	0.00029	0.99971	0.00029	60.00	216.00	1.0000	22.00	22.00	0.1358	A
12	750418	chr12:620679	620679	G	T	0.00819	0.99181	0.00819	60.00	221.70	0.0880	18.00	46.58	0.0108	G
12	750477	chr12:620738	620738	C	T	0.00044	0.99956	0.00044	60.00	228.00	1.0000	18.00	18.00	0.1219	C
12	750570	chr12:620831	620831	G	A	0.00026	0.99974	0.00026	60.00	202.00	1.0000	18.00	18.00	0.0509	G
12	750601	chr12:620862	620862	G	A	0.00026	0.99974	0.00026	60.00	228.00	1.0000	25.00	25.00	0.0539	G
12	750602	chr12:620863	620863	C	A	0.00039	0.99961	0.00039	60.00	228.00	1.0000	74.00	74.00	0.0000	A
12	750728	chr12:620989	620989	C	T	0.00028	0.99972	0.00028	60.00	130.00	1.0000	23.00	23.00	0.1063	C
12	751037	chr12:621298	621298	T	A	0.00049	0.99951	0.00049	60.00	63.00	1.0000	10.00	10.00	0.1060	T
12	751149	chr12:621410	621410	C	A	0.00039	0.99961	0.00039	60.00	228.00	1.0000	70.00	70.00	0.0000	C
12	751163	chr12:621424	621424	G	C	0.00034	0.99966	0.00034	60.00	228.00	1.0000	35.59	35.59	0.0072	G
12	751254	chr12:621515	621515	T	C	0.00044	0.99956	0.00044	60.00	228.00	1.0000	57.00	57.00	0.0035	T
12	751266	chr12:621527	621527	G	A	0.00025	0.99975	0.00025	60.00	228.00	1.0000	65.00	65.00	0.0020	G
12	751277	chr12:621538	621538	C	T	0.00050	0.99950	0.00050	60.00	255.00	1.0000	96.00	96.00	0.0015	C
12	751308	chr12:621569	621569	T	G	0.34391	0.65609	0.34391	60.00	219.24	0.1273	29.92	79.17	0.0038	T
12	751325	chr12:621586	621586	A	G	0.00025	0.99975	0.00025	60.00	228.00	1.0000	45.00	45.00	0.0030	A
12	751422	chr12:621683	621683	T	C	0.00039	0.99961	0.00039	60.00	228.00	1.0000	41.00	41.00	0.0000	T
12	752665	chr12:622926	622926	C	G	0.00026	0.99974	0.00026	60.00	228.00	1.0000	29.00	29.00	0.0310	C
12	752709	chr12:622970	622970	T	A	0.00044	0.99956	0.00044	60.00	227.00	1.0000	22.00	22.00	0.0018	T
12	752836	chr12:623097	623097	G	A	0.00039	0.99961	0.00039	60.00	228.00	1.0000	26.00	26.00	0.0015	G
12	752856	chr12:623117	623117	C	T	0.00028	0.99972	0.00028	60.00	41.00	1.0000	10.00	10.00	0.1043	C
12	752857	chr12:623118	623118	C	T	0.00039	0.99961	0.00039	60.00	228.00	1.0000	41.00	41.00	0.0031	C
12	752883	chr12:623144	623144	G	A	0.00026	0.99974	0.00026	60.00	228.00	1.0000	17.00	17.00	0.0464	G
12	752934	chr12:623195	623195	G	A	0.00046	0.99954	0.00046	60.00	228.00	1.0000	29.44	32.29	0.0147	G
12	752963	chr12:623224	623224	C	T	0.00045	0.99955	0.00045	60.00	228.00	1.0000	17.00	17.00	0.0177	C
12	752981	chr12:623242	623242	T	C	0.00051	0.99949	0.00051	60.00	65.50	1.0000	13.18	19.83	0.0165	T
12	752997	chr12:623258	623258	C	A	0.00046	0.99954	0.00046	60.00	59.00	1.0000	25.00	25.00	0.0424	C
12	753042	chr12:623303	623303	C	T	0.00044	0.99956	0.00044	60.00	228.00	1.0000	31.00	31.00	0.0053	C
12	753045	chr12:623306	623306	C	T	0.00068	0.99932	0.00068	60.00	228.00	1.0000	39.54	41.26	0.0045	C
12	753057	chr12:623318	623318	C	T	0.00025	0.99975	0.00025	60.00	228.00	1.0000	38.00	38.00	0.0025	C
12	753098	chr12:623359	623359	C	T	0.00025	0.99975	0.00025	60.00	228.00	1.0000	42.00	42.00	0.0095	C
12	753116	chr12:623377	623377	C	A	0.00104	0.99896	0.00104	60.00	65.18	1.0000	13.27	16.96	0.0241	C
12	753121	chr12:623382	623382	G	A	0.00092	0.99908	0.00092	60.00	135.50	1.0000	11.50	30.50	0.0389	G
12	753140	chr12:623401	623401	C	T	0.00026	0.99974	0.00026	60.00	40.00	1.0000	10.00	10.00	0.0464	C

12	753161	chr12:623422	623422	T	A	0.00039	0.99961	0.00039	60.00	228.00	1.0000	18.00	18.00	0.0000	T
12	753204	chr12:623465	623465	C	A	0.00039	0.99961	0.00039	60.00	228.00	1.0000	47.00	47.00	0.0008	C
12	753274	chr12:623535	623535	G	T	0.00080	0.99920	0.00080	60.00	226.56	1.0000	20.23	30.79	0.0064	G
12	753331	chr12:623592	623592	G	A	0.17685	0.82315	0.17685	60.00	222.34	0.6081	26.37	72.97	0.0027	G
12	753349	chr12:623610	623610	G	A	0.00025	0.99975	0.00025	60.00	228.00	1.0000	63.00	63.00	0.0010	G
12	753357	chr12:623618	623618	T	G	0.00025	0.99975	0.00025	60.00	228.00	1.0000	12.00	12.00	0.0010	T
12	753365	chr12:623626	623626	A	G	0.00025	0.99975	0.00025	60.00	228.00	1.0000	53.00	53.00	0.0015	A
12	753466	chr12:623727	623727	G	A	0.00025	0.99975	0.00025	60.00	228.00	1.0000	22.00	22.00	0.0085	G
12	753577	chr12:623838	623838	G	T	0.00025	0.99975	0.00025	60.00	228.00	1.0000	35.00	35.00	0.0000	G
12	753629	chr12:623890	623890	C	A	0.00025	0.99975	0.00025	60.00	228.00	1.0000	30.00	30.00	0.0015	C
12	753666	chr12:623927	623927	G	A	0.00046	0.99954	0.00046	60.00	228.00	1.0000	23.00	23.00	0.0327	G
12	753668	chr12:623929	623929	G	T	0.00039	0.99961	0.00039	60.00	228.00	1.0000	40.00	40.00	0.0000	G
12	753688	chr12:623949	623949	A	G	0.00113	0.99887	0.00113	60.00	228.00	1.0000	18.57	36.35	0.0052	A
12	753721	chr12:623982	623982	G	A	0.00025	0.99975	0.00025	60.00	228.00	1.0000	40.00	40.00	0.0045	G
12	753745	chr12:624006	624006	A	G	0.00049	0.99951	0.00049	60.00	228.00	1.0000	30.57	32.25	0.0183	A
12	753757	chr12:624018	624018	C	T	0.00093	0.99907	0.00093	60.00	192.50	1.0000	11.25	20.75	0.0512	C
12	753770	chr12:624031	624031	C	G	0.00025	0.99975	0.00025	60.00	228.00	1.0000	41.00	41.00	0.0065	C
12	753788	chr12:624049	624049	A	G	0.00039	0.99961	0.00039	60.00	228.00	1.0000	46.00	46.00	0.0000	A
12	753840	chr12:624101	624101	A	C	0.00025	0.99975	0.00025	60.00	59.00	1.0000	10.00	10.00	0.0200	A
12	753920	chr12:624181	624181	C	T	0.00039	0.99961	0.00039	60.00	114.00	1.0000	20.00	20.00	0.0023	C
12	753941	chr12:624202	624202	C	T	0.00042	0.99958	0.00042	60.00	104.60	1.0000	19.46	19.46	0.0202	C
12	753993	chr12:624254	624254	A	G	0.00061	0.99939	0.00061	60.00	228.00	1.0000	33.95	40.30	0.0018	A
12	754003	chr12:624264	624264	C	T	0.00032	0.99968	0.00032	60.00	228.00	1.0000	24.70	24.70	0.0070	C
12	754006	chr12:624267	624267	G	C	0.00025	0.99975	0.00025	60.00	228.00	1.0000	31.00	31.00	0.0055	G
12	754044	chr12:624305	624305	G	C	0.00039	0.99961	0.00039	60.00	228.00	1.0000	46.00	46.00	0.0000	G
12	754074	chr12:624335	624335	G	C	0.00039	0.99961	0.00039	60.00	228.00	1.0000	49.00	49.00	0.0000	G
12	754087	chr12:624348	624348	A	G	0.00025	0.99975	0.00025	60.00	228.00	1.0000	59.00	59.00	0.0005	A
12	754126	chr12:624387	624387	A	G	0.00032	0.99968	0.00032	60.00	228.00	1.0000	45.70	45.70	0.0006	A
12	754147	chr12:624408	624408	C	A	0.00044	0.99956	0.00044	60.00	154.00	1.0000	33.00	33.00	0.0009	C
12	754184	chr12:624445	624445	C	T	0.00025	0.99975	0.00025	60.00	228.00	1.0000	37.00	37.00	0.0005	C
12	754200	chr12:624461	624461	G	A	0.00030	0.99970	0.00030	60.00	228.00	1.0000	62.46	62.46	0.0003	G
12	754206	chr12:624467	624467	A	G	0.00025	0.99975	0.00025	60.00	228.00	1.0000	45.00	45.00	0.0005	A
12	754276	chr12:624537	624537	A	G	0.18476	0.81524	0.18476	60.00	224.78	0.3973	33.65	83.58	0.0020	G
12	754286	chr12:624547	624547	T	C	0.00025	0.99975	0.00025	60.00	228.00	1.0000	49.00	49.00	0.0000	T

12	754316	chr12:624577	624577	T	C	0.00835	0.99165	0.00835	60.00	227.32	1.0000	32.03	83.30	0.0000	T
12	754367	chr12:624628	624628	A	C	0.00025	0.99975	0.00025	60.00	228.00	1.0000	82.00	82.00	0.0000	A
12	754446	chr12:624707	624707	G	A	0.00044	0.99956	0.00044	60.00	228.00	1.0000	46.00	46.00	0.0000	G
12	754460	chr12:624721	624721	C	G	0.00025	0.99975	0.00025	60.00	228.00	1.0000	72.00	72.00	0.0000	C
12	754475	chr12:624736	624736	C	T	0.00077	0.99923	0.00077	60.00	228.00	1.0000	51.28	61.73	0.0000	C
12	754551	chr12:624812	624812	G	A	0.00025	0.99975	0.00025	60.00	228.00	1.0000	58.00	58.00	0.0000	G
12	754577	chr12:624838	624838	C	T	0.00039	0.99961	0.00039	60.00	228.00	1.0000	51.00	51.00	0.0000	c
12	754601	chr12:624862	624862	C	G	0.00025	0.99975	0.00025	60.00	228.00	1.0000	43.00	43.00	0.0000	C
12	754735	chr12:624996	624996	C	G	0.00050	0.99950	0.00050	60.00	58.00	1.0000	30.13	34.88	0.0090	C
12	754751	chr12:625012	625012	G	A	0.00044	0.99956	0.00044	60.00	161.00	1.0000	13.00	13.00	0.0009	G
12	754764	chr12:625025	625025	T	C	0.00039	0.99961	0.00039	60.00	228.00	1.0000	32.00	32.00	0.0000	T
12	754813	chr12:625074	625074	C	T	0.00061	0.99939	0.00061	60.00	228.00	1.0000	40.69	52.23	0.0003	C
12	754814	chr12:625075	625075	T	A	0.00030	0.99970	0.00030	60.00	228.00	1.0000	53.54	53.54	0.0003	T
12	754824	chr12:625085	625085	A	T	0.00039	0.99961	0.00039	60.00	228.00	1.0000	52.00	52.00	0.0000	A
12	754827	chr12:625088	625088	T	C	0.00050	0.99950	0.00050	60.00	82.00	1.0000	25.00	25.00	0.0030	T
12	754873	chr12:625134	625134	A	T	0.00068	0.99932	0.00068	60.00	228.00	1.0000	51.14	68.37	0.0002	A
12	754921	chr12:625182	625182	G	A	0.00098	0.99902	0.00098	60.00	75.26	1.0000	12.51	20.87	0.0682	G
12	754924	chr12:625185	625185	G	C	0.00077	0.99923	0.00077	60.00	34.50	1.0000	10.20	17.80	0.0000	G
12	754932	chr12:625193	625193	A	G	0.00039	0.99961	0.00039	60.00	30.00	1.0000	13.00	13.00	0.0000	A
12	754936	chr12:625197	625197	G	A	0.00126	0.99874	0.00126	60.00	91.80	1.0000	10.30	18.90	0.0080	G
12	754960	chr12:625221	625221	C	T	0.00025	0.99975	0.00025	60.00	228.00	1.0000	42.00	42.00	0.0005	C
12	754969	chr12:625230	625230	G	A	0.00025	0.99975	0.00025	60.00	228.00	1.0000	58.00	58.00	0.0000	G
12	754981	chr12:625242	625242	C	T	0.00025	0.99975	0.00025	60.00	224.00	1.0000	20.00	20.00	0.0005	C
12	755006	chr12:625267	625267	T	C	0.00025	0.99975	0.00025	60.00	228.00	1.0000	56.00	56.00	0.0005	T
12	755033	chr12:625294	625294	T	C	0.00025	0.99975	0.00025	60.00	90.00	1.0000	11.00	11.00	0.0125	T
12	755037	chr12:625298	625298	A	G	0.00039	0.99961	0.00039	60.00	51.00	1.0000	11.00	11.00	0.0008	A
12	755054	chr12:625315	625315	C	A	0.99305	0.00695	0.00695	60.00	97.40	0.0000	10.00	41.00	0.0659	A
12	755251	chr12:625512	625512	C	T	0.00044	0.99956	0.00044	60.00	228.00	1.0000	17.00	17.00	0.0027	C
12	755252	chr12:625513	625513	A	G	0.18361	0.81639	0.18361	60.00	220.79	0.3973	23.69	70.46	0.0056	G
12	755290	chr12:625551	625551	A	T	0.00039	0.99961	0.00039	60.00	228.00	1.0000	54.00	54.00	0.0000	A
12	755325	chr12:625586	625586	G	A	0.00025	0.99975	0.00025	60.00	228.00	1.0000	30.00	30.00	0.0005	G
12	755383	chr12:625644	625644	C	A	0.00025	0.99975	0.00025	60.00	228.00	1.0000	30.00	30.00	0.0075	C
12	755412	chr12:625673	625673	T	G	0.00025	0.99975	0.00025	60.00	158.00	1.0000	28.00	28.00	0.0075	T
12	755430	chr12:625691	625691	A	G	0.00025	0.99975	0.00025	60.00	46.00	1.0000	10.00	10.00	0.0080	G

12	755438	chr12:625699	625699	G	A	0.00039	0.99961	0.00039	60.00	228.00	1.0000	30.00	30.00	0.0000 G
12	755484	chr12:625745	625745	G	A	0.00080	0.99920	0.00080	60.00	120.50	1.0000	11.05	12.95	0.0340 G
12	755537	chr12:625798	625798	C	T	0.00030	0.99970	0.00030	60.00	199.00	1.0000	11.00	11.00	0.1727 C
12	755542	chr12:625803	625803	C	T	0.00039	0.99961	0.00039	60.00	61.00	1.0000	14.00	14.00	0.0154 c
12	755546	chr12:625807	625807	A	T	0.00027	0.99973	0.00027	60.00	69.00	1.0000	13.00	13.00	0.0764 A
12	755597	chr12:625858	625858	C	T	0.00025	0.99975	0.00025	60.00	228.00	1.0000	36.00	36.00	0.0090 C
12	755701	chr12:625962	625962	C	T	0.00025	0.99975	0.00025	60.00	228.00	1.0000	40.00	40.00	0.0035 C
12	755793	chr12:626054	626054	C	T	0.00030	0.99970	0.00030	60.00	228.00	1.0000	51.58	51.58	0.0033 C
12	755828	chr12:626089	626089	C	T	0.00044	0.99956	0.00044	60.00	228.00	1.0000	21.00	21.00	0.0000 C
12	755866	chr12:626127	626127	G	A	0.00025	0.99975	0.00025	60.00	228.00	1.0000	17.00	17.00	0.0015 G
12	755923	chr12:626184	626184	A	G	0.00814	0.99186	0.00814	60.00	223.77	1.0000	21.09	71.74	0.0014 A
12	755955	chr12:626216	626216	G	A	0.00025	0.99975	0.00025	60.00	228.00	1.0000	42.00	42.00	0.0020 G
12	755959	chr12:626220	626220	A	G	0.00044	0.99956	0.00044	60.00	228.00	1.0000	38.00	38.00	0.0018 A
12	755995	chr12:626256	626256	A	C	0.00025	0.99975	0.00025	60.00	228.00	1.0000	47.00	47.00	0.0020 A
12	756034	chr12:626295	626295	T	G	0.00025	0.99975	0.00025	60.00	228.00	1.0000	57.00	57.00	0.0050 T
12	756044	chr12:626305	626305	A	G	0.01290	0.98710	0.01290	60.00	226.79	1.0000	22.53	67.01	0.0025 A
12	756054	chr12:626315	626315	C	A	0.00050	0.99950	0.00050	60.00	228.00	1.0000	41.38	55.63	0.0040 C
12	756079	chr12:626340	626340	C	T	0.00025	0.99975	0.00025	60.00	87.00	1.0000	20.00	20.00	0.0050 C
12	756095	chr12:626356	626356	A	T	0.00050	0.99950	0.00050	60.00	132.09	1.0000	12.69	16.28	0.0350 A
12	756124	chr12:626385	626385	G	A	0.00039	0.99961	0.00039	60.00	228.00	1.0000	57.00	57.00	0.0000 g
12	756138	chr12:626399	626399	C	G	0.00025	0.99975	0.00025	60.00	228.00	1.0000	67.00	67.00	0.0050 C
12	756140	chr12:626401	626401	G	A	0.01312	0.98688	0.01312	60.00	227.95	1.0000	28.33	68.46	0.0025 G
12	756170	chr12:626431	626431	T	C	0.00062	0.99938	0.00062	60.00	228.00	1.0000	43.99	51.10	0.0008 T
12	756182	chr12:626443	626443	T	C	0.00025	0.99975	0.00025	60.00	228.00	1.0000	48.00	48.00	0.0110 T
12	756208	chr12:626469	626469	T	C	0.00025	0.99975	0.00025	60.00	228.00	1.0000	47.00	47.00	0.0130 T
12	756224	chr12:626485	626485	G	C	0.00025	0.99975	0.00025	60.00	228.00	1.0000	23.00	23.00	0.0145 G
12	756229	chr12:626490	626490	C	G	0.00025	0.99975	0.00025	60.00	228.00	1.0000	23.00	23.00	0.0140 C
12	756276	chr12:626537	626537	C	T	0.00025	0.99975	0.00025	60.00	154.00	1.0000	14.00	14.00	0.0150 C
12	756325	chr12:626586	626586	C	T	0.00044	0.99956	0.00044	60.00	228.00	1.0000	30.00	30.00	0.0009 C
12	756330	chr12:626591	626591	G	C	0.00077	0.99923	0.00077	60.00	228.00	1.0000	63.20	70.80	0.0000 G
12	756351	chr12:626612	626612	G	A	0.01867	0.98133	0.01867	60.00	223.73	1.0000	19.77	67.01	0.0088 G
12	756399	chr12:626660	626660	A	C	0.00025	0.99975	0.00025	60.00	222.00	1.0000	24.00	24.00	0.0145 A
12	756401	chr12:626662	626662	G	A	0.00387	0.99613	0.00387	60.00	228.00	1.0000	24.25	56.46	0.0083 G
12	756417	chr12:626678	626678	G	A	0.00046	0.99954	0.00046	60.00	228.00	1.0000	44.48	47.49	0.0087 G

12	756425	chr12:626686	626686	A	G	0.00039	0.99961	0.00039	60.00	228.00	1.0000	67.00	67.00	0.0000	A
12	756441	chr12:626702	626702	G	C	0.00044	0.99956	0.00044	60.00	228.00	1.0000	31.00	31.00	0.0062	G
12	756486	chr12:626747	626747	A	G	0.00039	0.99961	0.00039	60.00	228.00	1.0000	48.00	48.00	0.0000	A
12	756550	chr12:626811	626811	C	T	0.00025	0.99975	0.00025	60.00	228.00	1.0000	67.00	67.00	0.0080	C
12	756598	chr12:626859	626859	C	A	0.00056	0.99944	0.00056	60.00	69.50	1.0000	10.03	10.98	0.1123	-
12	756600	chr12:626861	626861	T	C	0.00193	0.99807	0.00193	60.00	163.57	1.0000	10.15	21.00	0.0959	-
12	756608	chr12:626869	626869	C	A	0.00086	0.99914	0.00086	59.67	79.00	1.0000	13.10	17.85	0.1278	-
12	756613	chr12:626874	626874	T	A	0.00082	0.99918	0.00082	60.00	87.54	1.0000	13.83	17.42	0.0713	-
12	756621	chr12:626882	626882	C	T	0.00027	0.99973	0.00027	60.00	166.00	1.0000	14.00	14.00	0.0684	C
12	756625	chr12:626886	626886	A	G	0.00837	0.99163	0.00837	60.00	199.87	0.0006	12.30	43.60	0.0289	A
12	756650	chr12:626911	626911	G	C	0.02649	0.97351	0.02649	60.00	225.67	0.2277	23.08	67.39	0.0032	G
12	756663	chr12:626924	626924	C	T	0.00039	0.99961	0.00039	60.00	228.00	1.0000	70.00	70.00	0.0000	C
12	756667	chr12:626928	626928	T	G	0.00025	0.99975	0.00025	60.00	163.00	1.0000	25.00	25.00	0.0060	T
12	756697	chr12:626958	626958	C	T	0.00025	0.99975	0.00025	60.00	35.00	1.0000	19.00	19.00	0.0145	C
12	756714	chr12:626975	626975	C	T	0.02514	0.97486	0.02514	60.00	226.35	0.2099	17.06	60.22	0.0123	C
12	756719	chr12:626980	626980	C	A	0.00032	0.99968	0.00032	60.00	228.00	1.0000	33.10	33.10	0.0165	C
12	756748	chr12:627009	627009	C	T	0.00026	0.99974	0.00026	60.00	228.00	1.0000	21.00	21.00	0.0280	C
12	756775	chr12:627036	627036	A	G	0.00045	0.99955	0.00045	60.00	31.00	1.0000	10.00	10.00	0.0265	A
12	756782	chr12:627043	627043	G	C	0.00039	0.99961	0.00039	60.00	198.00	1.0000	19.00	19.00	0.0000	G
12	756784	chr12:627045	627045	G	T	0.00026	0.99974	0.00026	60.00	104.00	1.0000	10.00	10.00	0.0574	G
12	756810	chr12:627071	627071	A	G	0.00039	0.99961	0.00039	60.00	48.00	1.0000	10.00	10.00	0.0031	A
12	756854	chr12:627115	627115	A	G	0.00039	0.99961	0.00039	60.00	228.00	1.0000	44.00	44.00	0.0000	A
12	756860	chr12:627121	627121	C	T	0.00741	0.99259	0.00741	60.00	225.95	1.0000	20.35	51.64	0.0096	T
12	756889	chr12:627150	627150	G	A	0.01080	0.98920	0.01080	60.00	227.92	1.0000	26.75	60.14	0.0070	G
12	756901	chr12:627162	627162	G	A	0.31534	0.68466	0.31534	60.00	207.44	0.0644	16.50	56.73	0.0256	G
12	756909	chr12:627170	627170	G	A	0.00039	0.99961	0.00039	60.00	228.00	1.0000	45.00	45.00	0.0000	G
12	756913	chr12:627174	627174	A	C	0.00026	0.99974	0.00026	60.00	228.00	1.0000	35.00	35.00	0.0334	A
12	756930	chr12:627191	627191	C	T	0.00032	0.99968	0.00032	60.00	228.00	1.0000	30.35	30.35	0.0447	C
12	757014	chr12:627275	627275	A	G	0.00025	0.99975	0.00025	60.00	228.00	1.0000	59.00	59.00	0.0050	A
12	757061	chr12:627322	627322	T	C	0.00338	0.99662	0.00338	60.00	56.40	1.0000	12.02	36.05	0.0302	T
12	757063	chr12:627324	627324	A	T	0.00060	0.99940	0.00060	60.00	73.84	1.0000	14.18	18.54	0.0546	A
12	757076	chr12:627337	627337	C	T	0.00025	0.99975	0.00025	60.00	227.00	1.0000	36.00	36.00	0.0030	C
12	757121	chr12:627382	627382	C	A	0.00044	0.99956	0.00044	60.00	228.00	1.0000	65.00	65.00	0.0000	C
12	757127	chr12:627388	627388	C	T	0.00025	0.99975	0.00025	60.00	228.00	1.0000	51.00	51.00	0.0020	C

12	757169	chr12:627430	627430	G	A	0.00025	0.99975	0.00025	60.00	161.00	1.0000	22.00	22.00	0.0060	G
12	757206	chr12:627467	627467	G	T	0.99884	0.00116	0.00116	60.00	124.49	1.0000	18.00	48.00	0.0000	T
12	757225	chr12:627486	627486	A	C	0.00025	0.99975	0.00025	60.00	147.00	1.0000	27.00	27.00	0.0105	A
12	757231	chr12:627492	627492	A	G	0.00025	0.99975	0.00025	60.00	102.00	1.0000	11.00	11.00	0.0115	A
12	757670	chr12:627931	627931	G	A	0.00025	0.99975	0.00025	60.00	228.00	1.0000	52.00	52.00	0.0045	G
12	757685	chr12:627946	627946	C	T	0.00025	0.99975	0.00025	60.00	228.00	1.0000	57.00	57.00	0.0045	C
12	757713	chr12:627974	627974	G	A	0.00147	0.99853	0.00147	60.00	228.00	1.0000	32.99	58.14	0.0014	g
12	757790	chr12:628051	628051	T	G	0.00044	0.99956	0.00044	60.00	228.00	1.0000	21.00	21.00	0.0035	T
12	757842	chr12:628103	628103	G	T	0.00050	0.99950	0.00050	60.00	228.00	1.0000	18.63	42.38	0.0020	G
12	758033	chr12:628294	628294	C	T	0.00025	0.99975	0.00025	60.00	228.00	1.0000	40.00	40.00	0.0005	C
12	758043	chr12:628304	628304	G	A	0.00077	0.99923	0.00077	60.00	228.00	1.0000	57.20	64.80	0.0000	G
12	758057	chr12:628318	628318	C	T	0.00091	0.99909	0.00091	60.00	228.00	1.0000	62.09	76.28	0.0003	C
12	758058	chr12:628319	628319	G	A	0.00039	0.99961	0.00039	60.00	228.00	1.0000	54.00	54.00	0.0000	G
12	758077	chr12:628338	628338	A	G	0.00088	0.99912	0.00088	60.00	228.00	1.0000	46.30	57.70	0.0000	A
12	758078	chr12:628339	628339	C	G	0.00050	0.99950	0.00050	60.00	228.00	1.0000	43.63	67.38	0.0005	C
12	758090	chr12:628351	628351	C	T	0.00169	0.99831	0.00169	60.00	228.00	1.0000	47.91	66.27	0.0002	C
12	758092	chr12:628353	628353	A	G	0.00025	0.99975	0.00025	60.00	228.00	1.0000	68.00	68.00	0.0005	a
12	758121	chr12:628382	628382	A	G	0.00044	0.99956	0.00044	60.00	209.00	1.0000	23.00	23.00	0.0035	A
12	758166	chr12:628427	628427	A	G	0.00501	0.99499	0.00501	58.38	131.25	0.0000	10.18	15.65	0.0756	-
12	758584	chr12:628845	628845	A	G	0.00050	0.99950	0.00050	60.00	171.75	1.0000	14.76	16.08	0.0383	A
12	758595	chr12:628856	628856	C	G	0.00127	0.99873	0.00127	60.00	196.98	1.0000	18.96	30.88	0.0216	C
12	758609	chr12:628870	628870	G	T	0.00025	0.99975	0.00025	60.00	103.00	1.0000	19.00	19.00	0.0130	G
12	758839	chr12:629100	629100	A	T	0.00026	0.99974	0.00026	60.00	81.00	1.0000	10.00	10.00	0.0399	A
12	758844	chr12:629105	629105	A	G	0.00052	0.99948	0.00052	60.00	114.00	1.0000	12.00	12.00	0.1546	A
12	758872	chr12:629133	629133	G	A	0.00051	0.99949	0.00051	60.00	171.00	1.0000	23.13	27.88	0.0130	G
12	758887	chr12:629148	629148	A	G	0.00263	0.99737	0.00263	60.00	227.20	1.0000	18.48	45.91	0.0148	A
12	758892	chr12:629153	629153	C	T	0.00045	0.99955	0.00045	60.00	228.00	1.0000	27.00	27.00	0.0203	C
12	758967	chr12:629228	629228	A	G	0.00025	0.99975	0.00025	60.00	228.00	1.0000	68.00	68.00	0.0010	A
12	758979	chr12:629240	629240	A	G	0.00030	0.99970	0.00030	60.00	228.00	1.0000	45.44	45.44	0.0027	A
12	759039	chr12:629300	629300	G	T	0.00025	0.99975	0.00025	60.00	228.00	1.0000	49.00	49.00	0.0005	G
12	759097	chr12:629358	629358	T	A	0.00044	0.99956	0.00044	60.00	112.00	1.0000	12.00	12.00	0.0062	T
12	759175	chr12:629436	629436	T	C	0.00048	0.99952	0.00048	60.00	228.00	1.0000	25.05	26.88	0.0063	T
12	759198	chr12:629459	629459	A	G	0.00039	0.99961	0.00039	60.00	228.00	1.0000	51.00	51.00	0.0000	A
12	759848	chr12:630109	630109	C	A	0.00026	0.99974	0.00026	60.00	94.00	1.0000	10.00	10.00	0.0270	C

12	759874	chr12:630135	630135	A	G	0.00039	0.99961	0.00039	60.00	228.00	1.0000	46.00	46.00	0.0000	A
12	759976	chr12:630237	630237	T	C	0.00044	0.99956	0.00044	60.00	228.00	1.0000	43.00	43.00	0.0053	T
12	759994	chr12:630255	630255	C	T	0.00025	0.99975	0.00025	60.00	228.00	1.0000	45.00	45.00	0.0035	C
12	759995	chr12:630256	630256	G	A	0.00045	0.99955	0.00045	60.00	228.00	1.0000	33.00	33.00	0.0124	G
12	760005	chr12:630266	630266	C	T	0.00025	0.99975	0.00025	60.00	186.00	1.0000	26.00	26.00	0.0050	C
12	760044	chr12:630305	630305	A	G	0.00026	0.99974	0.00026	60.00	50.00	1.0000	13.00	13.00	0.0509	A
12	760046	chr12:630307	630307	A	G	0.00039	0.99961	0.00039	60.00	82.00	1.0000	14.00	14.00	0.0023	A
12	760078	chr12:630339	630339	C	T	0.00045	0.99955	0.00045	60.00	66.00	1.0000	10.00	10.00	0.0177	C
12	760094	chr12:630355	630355	C	T	0.00045	0.99955	0.00045	60.00	228.00	1.0000	23.00	23.00	0.0247	C
12	760143	chr12:630404	630404	G	A	0.00025	0.99975	0.00025	60.00	228.00	1.0000	38.00	38.00	0.0025	G
12	760156	chr12:630417	630417	G	A	0.00025	0.99975	0.00025	60.00	228.00	1.0000	39.00	39.00	0.0025	G
12	760164	chr12:630425	630425	G	C	0.00284	0.99716	0.00284	60.00	224.87	1.0000	24.15	47.59	0.0051	G
12	760166	chr12:630427	630427	G	C	0.00025	0.99975	0.00025	60.00	187.00	1.0000	33.00	33.00	0.0025	G
12	760177	chr12:630438	630438	A	G	0.00025	0.99975	0.00025	60.00	228.00	1.0000	29.00	29.00	0.0035	A
12	760210	chr12:630471	630471	G	A	0.00025	0.99975	0.00025	60.00	228.00	1.0000	29.00	29.00	0.0020	G
12	760322	chr12:630583	630583	A	G	0.00039	0.99961	0.00039	60.00	228.00	1.0000	55.00	55.00	0.0000	A
12	760334	chr12:630595	630595	A	G	0.00025	0.99975	0.00025	60.00	228.00	1.0000	28.00	28.00	0.0020	A
12	760340	chr12:630601	630601	A	C	0.00044	0.99956	0.00044	60.00	168.00	1.0000	12.00	12.00	0.0035	A
12	760361	chr12:630622	630622	A	G	0.00030	0.99970	0.00030	60.00	228.00	1.0000	40.93	40.93	0.0012	A
12	760399	chr12:630660	630660	A	G	0.00045	0.99955	0.00045	60.00	58.00	1.0000	10.00	10.00	0.0265	A
12	760516	chr12:630777	630777	T	C	0.00083	0.99917	0.00083	60.00	228.00	1.0000	39.40	43.37	0.0021	T
12	760522	chr12:630783	630783	C	T	0.00077	0.99923	0.00077	60.00	228.00	1.0000	55.05	56.95	0.0000	C
12	760565	chr12:630826	630826	C	T	0.00025	0.99975	0.00025	60.00	228.00	1.0000	42.00	42.00	0.0015	A
12	760566	chr12:630827	630827	G	A	0.00113	0.99887	0.00113	60.00	228.00	1.0000	28.34	52.05	0.0016	G
12	760570	chr12:630831	630831	T	G	0.00039	0.99961	0.00039	60.00	228.00	1.0000	56.00	56.00	0.0000	T
12	760577	chr12:630838	630838	G	A	0.00039	0.99961	0.00039	60.00	228.00	1.0000	48.00	48.00	0.0000	G
12	760668	chr12:630929	630929	T	C	0.00025	0.99975	0.00025	60.00	228.00	1.0000	61.00	61.00	0.0020	T
12	760676	chr12:630937	630937	C	G	0.00044	0.99956	0.00044	60.00	228.00	1.0000	49.00	49.00	0.0035	C
12	760694	chr12:630955	630955	T	A	0.00025	0.99975	0.00025	60.00	156.00	1.0000	33.00	33.00	0.0020	T
12	760718	chr12:630979	630979	G	T	0.00047	0.99953	0.00047	60.00	51.00	1.0000	15.00	15.00	0.0512	G
12	760725	chr12:630986	630986	T	G	0.00025	0.99975	0.00025	60.00	113.00	1.0000	17.00	17.00	0.0125	T
12	760728	chr12:630989	630989	C	A	0.00025	0.99975	0.00025	60.00	87.00	1.0000	14.00	14.00	0.0140	C
12	760732	chr12:630993	630993	G	C	0.00039	0.99961	0.00039	59.00	228.00	1.0000	26.00	26.00	0.0000	G
12	760757	chr12:631018	631018	T	C	0.01120	0.98880	0.01120	59.16	217.99	1.0000	13.63	37.82	0.0219	T

12	760773	chr12:631034	631034	A	C	0.00039	0.99961	0.00039	60.00	228.00	1.0000	49.00	49.00	0.0000	A
12	760818	chr12:631079	631079	A	C	0.00125	0.99875	0.00125	60.00	217.25	1.0000	28.64	38.95	0.0098	A
12	760826	chr12:631087	631087	A	G	0.00025	0.99975	0.00025	60.00	228.00	1.0000	45.00	45.00	0.0050	A
12	760835	chr12:631096	631096	A	G	0.00057	0.99943	0.00057	60.00	228.00	1.0000	25.18	31.36	0.0056	A
12	760843	chr12:631104	631104	T	C	0.00045	0.99955	0.00045	60.00	228.00	1.0000	29.00	29.00	0.0097	T
12	760879	chr12:631140	631140	G	T	0.00025	0.99975	0.00025	60.00	228.00	1.0000	34.00	34.00	0.0035	G
12	760927	chr12:631188	631188	G	A	0.00046	0.99954	0.00046	60.00	228.00	1.0000	16.00	16.00	0.0389	G
12	760941	chr12:631202	631202	C	T	0.00105	0.99895	0.00105	60.00	228.00	1.0000	25.03	30.90	0.0193	C
12	760942	chr12:631203	631203	G	A	0.00039	0.99961	0.00039	60.00	228.00	1.0000	25.00	25.00	0.0000	G
12	760946	chr12:631207	631207	G	C	0.00046	0.99954	0.00046	60.00	228.00	1.0000	19.00	19.00	0.0477	G
12	760983	chr12:631244	631244	A	G	0.00047	0.99953	0.00047	59.00	52.00	1.0000	10.00	10.00	0.0539	A
12	761000	chr12:631261	631261	C	T	0.00050	0.99950	0.00050	59.50	226.00	1.0000	16.25	25.75	0.0070	C
12	761001	chr12:631262	631262	G	A	0.00025	0.99975	0.00025	58.00	228.00	1.0000	25.00	25.00	0.0080	G
12	761035	chr12:631296	631296	G	A	0.00807	0.99193	0.00807	60.00	224.19	1.0000	19.52	56.50	0.0069	G
12	761237	chr12:631498	631498	T	A	0.00055	0.99945	0.00055	60.00	110.00	1.0000	13.00	13.00	0.1943	T
12	761249	chr12:631510	631510	G	A	0.00027	0.99973	0.00027	60.00	228.00	1.0000	18.00	18.00	0.0684	G
12	761309	chr12:631570	631570	C	T	0.00040	0.99960	0.00040	60.00	207.00	1.0000	17.00	17.00	0.0301	C
12	761341	chr12:631602	631602	C	T	0.00042	0.99958	0.00042	60.00	228.00	1.0000	23.00	23.00	0.0733	C
12	761405	chr12:631666	631666	C	T	0.00045	0.99955	0.00045	60.00	89.00	1.0000	10.00	10.00	0.0124	C
12	761410	chr12:631671	631671	G	A	0.00026	0.99974	0.00026	60.00	228.00	1.0000	34.00	34.00	0.0320	G
12	761431	chr12:631692	631692	A	G	0.00042	0.99958	0.00042	60.00	101.85	1.0000	22.85	22.85	0.0094	A
12	761432	chr12:631693	631693	A	G	0.00044	0.99956	0.00044	60.00	47.00	1.0000	15.00	15.00	0.0053	A
12	761457	chr12:631718	631718	A	T	0.00044	0.99956	0.00044	60.00	226.00	1.0000	15.00	15.00	0.0027	A
12	761479	chr12:631740	631740	A	G	0.00039	0.99961	0.00039	60.00	228.00	1.0000	56.00	56.00	0.0000	a
12	761485	chr12:631746	631746	C	T	0.00050	0.99950	0.00050	60.00	228.00	1.0000	38.20	45.80	0.0035	C
12	761498	chr12:631759	631759	C	T	0.00045	0.99955	0.00045	59.23	190.32	1.0000	23.65	27.07	0.0047	C
12	761545	chr12:631806	631806	A	T	0.00307	0.99693	0.00307	59.35	219.60	1.0000	16.46	35.48	0.0088	A
12	761560	chr12:631821	631821	T	A	0.25705	0.74295	0.25705	59.98	202.92	0.0877	14.56	50.67	0.0622	T
12	761623	chr12:631884	631884	A	C	0.00049	0.99951	0.00049	59.00	228.00	1.0000	14.00	14.00	0.0981	A
12	762494	chr12:632755	632755	T	C	0.00048	0.99952	0.00048	60.00	228.00	1.0000	16.00	16.00	0.0804	T
12	762516	chr12:632777	632777	A	C	0.00048	0.99952	0.00048	60.00	228.00	1.0000	20.00	20.00	0.0786	A
12	762523	chr12:632784	632784	A	C	0.00028	0.99972	0.00028	60.00	217.00	1.0000	15.00	15.00	0.1118	A
12	762532	chr12:632793	632793	A	G	0.00048	0.99952	0.00048	60.00	228.00	1.0000	25.00	25.00	0.0795	A
12	762618	chr12:632879	632879	G	A	0.00025	0.99975	0.00025	60.00	175.00	1.0000	26.00	26.00	0.0105	G

12	762639	chr12:632900	632900	G	A	0.01913	0.98087	0.01913	59.96	227.18	1.0000	26.84	73.61	0.0029	G
12	762702	chr12:632963	632963	G	T	0.00046	0.99954	0.00046	60.00	228.00	1.0000	29.65	42.74	0.0021	G
12	762720	chr12:632981	632981	G	A	0.00025	0.99975	0.00025	60.00	228.00	1.0000	28.00	28.00	0.0010	G
12	762867	chr12:633128	633128	A	G	0.00026	0.99974	0.00026	60.00	38.00	1.0000	14.00	14.00	0.0399	a
12	762922	chr12:633183	633183	A	G	0.00032	0.99968	0.00032	60.00	162.13	1.0000	22.00	22.00	0.0019	a
12	762983	chr12:633244	633244	T	C	0.00044	0.99956	0.00044	60.00	228.00	1.0000	30.00	30.00	0.0000	t
12	762986	chr12:633247	633247	T	C	0.00039	0.99961	0.00039	60.00	228.00	1.0000	59.00	59.00	0.0000	t
12	763021	chr12:633282	633282	G	A	0.00039	0.99961	0.00039	60.00	228.00	1.0000	44.00	44.00	0.0000	g
12	763344	chr12:633605	633605	G	A	0.00025	0.99975	0.00025	60.00	228.00	1.0000	56.00	56.00	0.0000	g
12	763361	chr12:633622	633622	T	C	0.00050	0.99950	0.00050	60.00	228.00	1.0000	60.18	66.83	0.0000	t
12	763370	chr12:633631	633631	A	T	0.28141	0.71859	0.28141	60.00	221.98	0.0069	35.10	79.86	0.0011	A
12	763387	chr12:633648	633648	C	T	0.00045	0.99955	0.00045	60.00	228.00	1.0000	68.32	72.36	0.0000	c
12	764241	chr12:634502	634502	A	G	0.00098	0.99902	0.00098	59.23	175.93	1.0000	13.52	17.41	0.0771	A
12	764295	chr12:634556	634556	G	A	0.00025	0.99975	0.00025	60.00	228.00	1.0000	37.00	37.00	0.0015	G
12	764302	chr12:634563	634563	C	A	0.00025	0.99975	0.00025	60.00	228.00	1.0000	57.00	57.00	0.0010	C
12	764360	chr12:634621	634621	T	C	0.00044	0.99956	0.00044	60.00	228.00	1.0000	47.00	47.00	0.0009	T
12	764387	chr12:634648	634648	C	A	0.00032	0.99968	0.00032	60.00	228.00	1.0000	53.64	53.64	0.0006	C
12	764392	chr12:634653	634653	C	G	0.00025	0.99975	0.00025	60.00	228.00	1.0000	51.00	51.00	0.0005	C
12	764460	chr12:634721	634721	G	A	0.00025	0.99975	0.00025	60.00	228.00	1.0000	22.00	22.00	0.0015	G
12	764527	chr12:634788	634788	G	C	0.00431	0.99569	0.00431	59.52	223.12	1.0000	14.63	34.88	0.0043	G
12	764552	chr12:634813	634813	A	G	0.00030	0.99970	0.00030	59.61	228.00	1.0000	37.61	37.61	0.0021	A
12	764568	chr12:634829	634829	A	C	0.00025	0.99975	0.00025	59.00	228.00	1.0000	46.00	46.00	0.0030	A
12	765175	chr12:635436	635436	A	C	0.00025	0.99975	0.00025	60.00	192.00	1.0000	28.00	28.00	0.0080	a
12	765207	chr12:635468	635468	A	G	0.00025	0.99975	0.00025	60.00	94.00	1.0000	23.00	23.00	0.0100	a
12	765211	chr12:635472	635472	T	C	0.00039	0.99961	0.00039	60.00	228.00	1.0000	51.00	51.00	0.0000	t
12	765255	chr12:635516	635516	G	T	0.00039	0.99961	0.00039	60.00	228.00	1.0000	33.00	33.00	0.0000	g
12	765266	chr12:635527	635527	C	T	0.00039	0.99961	0.00039	60.00	228.00	1.0000	25.00	25.00	0.0000	c
12	765557	chr12:635818	635818	A	G	0.00492	0.99508	0.00492	59.98	198.89	0.0131	10.49	30.49	0.0796	A
12	765597	chr12:635858	635858	G	A	0.00039	0.99961	0.00039	60.00	228.00	1.0000	17.00	17.00	0.0000	G
12	765612	chr12:635873	635873	G	A	0.00039	0.99961	0.00039	59.00	228.00	1.0000	39.00	39.00	0.0000	G
12	765615	chr12:635876	635876	T	C	0.00044	0.99956	0.00044	59.00	228.00	1.0000	22.00	22.00	0.0009	T
12	765634	chr12:635895	635895	C	T	0.00025	0.99975	0.00025	60.00	228.00	1.0000	26.00	26.00	0.0110	C
12	766084	chr12:636345	636345	G	A	0.00025	0.99975	0.00025	60.00	228.00	1.0000	48.00	48.00	0.0005	G
12	766102	chr12:636363	636363	C	T	0.00044	0.99956	0.00044	60.00	228.00	1.0000	35.00	35.00	0.0009	C

12	766106	chr12:636367	636367	C	T	0.00100	0.99900	0.00100	60.00	228.00	1.0000	27.35	73.65	0.0010	C
12	766107	chr12:636368	636368	A	G	0.00025	0.99975	0.00025	60.00	228.00	1.0000	51.00	51.00	0.0010	A
12	766120	chr12:636381	636381	A	C	0.00025	0.99975	0.00025	60.00	228.00	1.0000	59.00	59.00	0.0010	A
12	766315	chr12:636576	636576	G	A	0.00025	0.99975	0.00025	60.00	228.00	1.0000	32.00	32.00	0.0020	G
12	766326	chr12:636587	636587	C	G	0.00032	0.99968	0.00032	60.00	228.00	1.0000	37.83	37.83	0.0010	C
12	766355	chr12:636616	636616	A	G	0.00133	0.99867	0.00133	60.00	228.00	1.0000	22.05	29.65	0.0018	A
12	766399	chr12:636660	636660	G	C	0.00032	0.99968	0.00032	59.00	210.32	1.0000	16.20	16.20	0.0048	G
12	766427	chr12:636688	636688	T	C	0.00136	0.99864	0.00136	60.00	228.00	1.0000	27.57	43.82	0.0014	T
12	766439	chr12:636700	636700	A	G	0.00050	0.99950	0.00050	60.00	228.00	1.0000	20.43	36.58	0.0020	A
12	766477	chr12:636738	636738	C	T	0.00090	0.99910	0.00090	59.50	99.00	1.0000	10.13	14.88	0.0203	C
12	766481	chr12:636742	636742	C	T	0.00198	0.99802	0.00198	59.61	195.37	1.0000	12.34	22.75	0.0310	C
12	766831	chr12:637092	637092	G	A	0.00044	0.99956	0.00044	60.00	228.00	1.0000	72.00	72.00	0.0009	g
12	766866	chr12:637127	637127	C	T	0.00039	0.99961	0.00039	59.00	228.00	1.0000	42.00	42.00	0.0000	c
12	766919	chr12:637180	637180	G	A	0.00025	0.99975	0.00025	60.00	228.00	1.0000	33.00	33.00	0.0005	g
12	766960	chr12:637221	637221	A	T	0.00025	0.99975	0.00025	60.00	125.00	1.0000	22.00	22.00	0.0020	a
12	766961	chr12:637222	637222	C	G	0.00025	0.99975	0.00025	60.00	228.00	1.0000	45.00	45.00	0.0020	c
12	766985	chr12:637246	637246	C	T	0.00077	0.99923	0.00077	60.00	228.00	1.0000	53.65	78.35	0.0000	c
12	767051	chr12:637312	637312	T	C	0.00044	0.99956	0.00044	58.00	56.00	1.0000	14.00	14.00	0.0044	t
12	767054	chr12:637315	637315	C	T	0.00039	0.99961	0.00039	59.00	47.00	1.0000	10.00	10.00	0.0000	c
12	767057	chr12:637318	637318	A	G	0.00025	0.99975	0.00025	59.00	58.00	1.0000	10.00	10.00	0.0015	a
12	767101	chr12:637362	637362	G	A	0.00025	0.99975	0.00025	60.00	228.00	1.0000	42.00	42.00	0.0010	g
12	767121	chr12:637382	637382	C	A	0.00025	0.99975	0.00025	60.00	228.00	1.0000	59.00	59.00	0.0005	c
12	767153	chr12:637414	637414	G	T	0.00080	0.99920	0.00080	60.00	130.50	1.0000	10.08	12.93	0.0301	g
12	767896	chr12:638157	638157	T	C	0.00025	0.99975	0.00025	60.00	87.00	1.0000	11.00	11.00	0.0065	T
12	767901	chr12:638162	638162	T	C	0.00771	0.99229	0.00771	59.38	216.58	1.0000	11.15	43.10	0.0047	T
12	767912	chr12:638173	638173	A	G	0.00045	0.99955	0.00045	60.00	228.00	1.0000	32.00	32.00	0.0088	A
12	767914	chr12:638175	638175	T	C	0.00025	0.99975	0.00025	60.00	112.00	1.0000	12.00	12.00	0.0050	T
12	767917	chr12:638178	638178	A	G	0.00045	0.99955	0.00045	60.00	228.00	1.0000	37.00	37.00	0.0088	A
12	767930	chr12:638191	638191	G	A	0.00039	0.99961	0.00039	60.00	220.00	1.0000	31.00	31.00	0.0000	G
12	767994	chr12:638255	638255	G	T	0.00025	0.99975	0.00025	59.00	228.00	1.0000	30.00	30.00	0.0010	G
12	768010	chr12:638271	638271	G	T	0.00039	0.99961	0.00039	59.00	228.00	1.0000	46.00	46.00	0.0000	G
12	768033	chr12:638294	638294	G	A	0.99796	0.00204	0.00204	59.21	181.23	1.0000	26.48	73.57	0.0034	A
12	768039	chr12:638300	638300	A	G	0.00039	0.99961	0.00039	60.00	228.00	1.0000	53.00	53.00	0.0000	A
12	768069	chr12:638330	638330	A	G	0.00102	0.99898	0.00102	60.00	228.00	1.0000	56.79	61.50	0.0005	A

12	768137	chr12:638398	638398	C	T	0.00025	0.99975	0.00025	60.00	165.00	1.0000	21.00	21.00	0.0040	C
12	768190	chr12:638451	638451	A	C	0.00047	0.99953	0.00047	60.00	158.00	1.0000	15.00	15.00	0.0636	A
12	768349	chr12:638610	638610	C	T	0.00039	0.99961	0.00039	60.00	228.00	1.0000	63.00	63.00	0.0000	C
12	768367	chr12:638628	638628	A	G	0.18402	0.81598	0.18402	60.00	223.23	0.4769	28.15	79.17	0.0072	A
12	768377	chr12:638638	638638	C	T	0.18446	0.81554	0.18446	60.00	223.47	0.3182	28.75	81.27	0.0065	T
12	768429	chr12:638690	638690	T	C	0.00025	0.99975	0.00025	60.00	228.00	1.0000	49.00	49.00	0.0005	T
12	768477	chr12:638738	638738	A	G	0.00044	0.99956	0.00044	60.00	65.00	1.0000	10.00	10.00	0.0071	A
12	768487	chr12:638748	638748	A	C	0.00031	0.99969	0.00031	60.00	61.99	1.0000	26.61	26.61	0.0215	A
12	768492	chr12:638753	638753	G	T	0.00125	0.99875	0.00125	60.00	228.00	1.0000	33.93	53.28	0.0047	G
12	768506	chr12:638767	638767	G	A	0.00045	0.99955	0.00045	60.00	228.00	1.0000	42.00	42.00	0.0080	G
12	768512	chr12:638773	638773	C	T	0.00081	0.99919	0.00081	60.00	228.00	1.0000	15.07	44.29	0.0099	C
12	768575	chr12:638836	638836	A	G	0.00160	0.99840	0.00160	60.00	201.73	1.0000	25.19	39.15	0.0121	A
12	768577	chr12:638838	638838	T	C	0.00045	0.99955	0.00045	60.00	201.00	1.0000	23.00	23.00	0.0150	T
12	768604	chr12:638865	638865	C	T	0.00045	0.99955	0.00045	60.00	39.00	1.0000	15.00	15.00	0.0230	C
12	768667	chr12:638928	638928	C	T	0.00027	0.99973	0.00027	60.00	155.00	1.0000	22.00	22.00	0.0834	C
12	768668	chr12:638929	638929	G	T	0.00039	0.99961	0.00039	60.00	228.00	1.0000	36.00	36.00	0.0000	G
12	768715	chr12:638976	638976	G	T	0.00030	0.99970	0.00030	60.00	228.00	1.0000	24.00	24.00	0.1623	G
12	768745	chr12:639006	639006	A	G	0.16009	0.83991	0.16009	60.00	203.09	0.6140	11.00	36.70	0.0216	G
12	768838	chr12:639099	639099	C	T	0.00047	0.99953	0.00047	60.00	228.00	1.0000	28.00	28.00	0.0574	C
12	768840	chr12:639101	639101	A	G	0.00047	0.99953	0.00047	60.00	228.00	1.0000	22.00	22.00	0.0565	A
12	768852	chr12:639113	639113	C	T	0.00051	0.99949	0.00051	60.00	228.00	1.0000	19.22	21.31	0.0681	C
12	768874	chr12:639135	639135	T	C	0.12862	0.87138	0.12862	60.00	192.36	0.0075	13.94	47.23	0.0986	C
12	768904	chr12:639165	639165	A	G	0.00025	0.99975	0.00025	60.00	228.00	1.0000	49.00	49.00	0.0080	A
12	768926	chr12:639187	639187	A	G	0.00045	0.99955	0.00045	60.00	228.00	1.0000	19.00	19.00	0.0159	A
12	768966	chr12:639227	639227	C	T	0.18098	0.81902	0.18098	60.00	215.71	0.3182	18.70	63.50	0.0172	T
12	768988	chr12:639249	639249	C	T	0.00025	0.99975	0.00025	60.00	57.00	1.0000	14.00	14.00	0.0150	C
12	768989	chr12:639250	639250	G	A	0.00077	0.99923	0.00077	60.00	228.00	1.0000	57.20	64.80	0.0000	g
12	769020	chr12:639281	639281	G	C	0.00039	0.99961	0.00039	60.00	45.00	1.0000	30.00	30.00	0.0000	G
12	769122	chr12:639383	639383	C	T	0.16946	0.83054	0.16946	60.00	209.80	0.1525	18.81	60.90	0.0287	T
12	769845	chr12:640106	640106	G	A	0.00049	0.99951	0.00049	60.00	210.22	1.0000	20.51	25.95	0.0242	g
12	770236	chr12:640497	640497	C	T	0.00025	0.99975	0.00025	60.00	217.00	1.0000	12.00	12.00	0.0050	c
12	770242	chr12:640503	640503	A	G	0.00031	0.99969	0.00031	60.00	152.03	1.0000	14.24	14.24	0.0282	a
12	770536	chr12:640797	640797	C	T	0.00169	0.99831	0.00169	60.00	80.33	1.0000	10.00	11.90	0.0872	c
12	770541	chr12:640802	640802	C	T	0.00611	0.99389	0.00611	59.54	117.00	0.0103	10.00	16.00	0.0532	C

12	770628	chr12:640889	640889	A	G	0.00044	0.99956	0.00044	60.00	228.00	1.0000	62.00	62.00	0.0027	A
12	770657	chr12:640918	640918	G	C	0.00025	0.99975	0.00025	60.00	228.00	1.0000	53.00	53.00	0.0005	G
12	770707	chr12:640968	640968	G	A	0.00039	0.99961	0.00039	60.00	228.00	1.0000	79.00	79.00	0.0000	G
12	770772	chr12:641033	641033	T	G	0.00025	0.99975	0.00025	60.00	59.00	1.0000	11.00	11.00	0.0055	T
12	770807	chr12:641068	641068	A	G	0.00039	0.99961	0.00039	60.00	228.00	1.0000	62.00	62.00	0.0000	A
12	770818	chr12:641079	641079	C	G	0.00025	0.99975	0.00025	60.00	94.00	1.0000	30.00	30.00	0.0020	C
12	770929	chr12:641190	641190	G	T	0.17343	0.82657	0.17343	60.00	209.62	0.2385	14.44	44.54	0.0421	G
12	770973	chr12:641234	641234	T	C	0.00045	0.99955	0.00045	60.00	228.00	1.0000	26.00	26.00	0.0221	T
12	771016	chr12:641277	641277	C	T	0.07537	0.92463	0.07537	60.00	222.75	0.4552	16.80	55.33	0.0103	C
12	771020	chr12:641281	641281	C	T	0.00025	0.99975	0.00025	60.00	228.00	1.0000	20.00	20.00	0.0100	C
12	771045	chr12:641306	641306	A	C	0.17503	0.82497	0.17503	60.00	211.11	0.3775	14.35	48.24	0.0237	C
12	771073	chr12:641334	641334	C	T	0.00046	0.99954	0.00046	60.00	146.00	1.0000	14.00	14.00	0.0415	C
12	771079	chr12:641340	641340	G	T	0.00063	0.99937	0.00063	60.00	53.01	1.0000	14.17	18.67	0.0378	G
12	771103	chr12:641364	641364	A	G	0.17576	0.82424	0.17576	60.00	205.85	0.0627	12.05	40.78	0.0438	G
12	771109	chr12:641370	641370	C	A	0.00046	0.99954	0.00046	60.00	228.00	1.0000	14.00	14.00	0.0406	C
12	771114	chr12:641375	641375	C	A	0.00045	0.99955	0.00045	60.00	114.00	1.0000	12.00	12.00	0.0283	C
12	771191	chr12:641452	641452	A	G	0.00025	0.99975	0.00025	60.00	111.00	1.0000	13.00	13.00	0.0145	A
12	771229	chr12:641490	641490	A	G	0.59572	0.40428	0.40428	60.00	178.15	0.1459	14.42	48.94	0.0339	G
12	771281	chr12:641542	641542	G	A	0.00851	0.99149	0.00851	60.00	226.98	1.0000	20.65	57.41	0.0050	G
12	771294	chr12:641555	641555	T	G	0.00025	0.99975	0.00025	60.00	228.00	1.0000	21.00	21.00	0.0070	T
12	771313	chr12:641574	641574	A	G	0.00050	0.99950	0.00050	60.00	228.00	1.0000	33.03	33.98	0.0040	A
12	771335	chr12:641596	641596	A	C	0.00061	0.99939	0.00061	60.00	228.00	1.0000	32.58	38.91	0.0021	A
12	771390	chr12:641651	641651	C	T	0.00031	0.99969	0.00031	60.00	228.00	1.0000	33.40	33.40	0.0090	C
12	771391	chr12:641652	641652	C	T	0.00025	0.99975	0.00025	60.00	228.00	1.0000	19.00	19.00	0.0145	C
12	771398	chr12:641659	641659	G	A	0.00039	0.99961	0.00039	60.00	228.00	1.0000	45.00	45.00	0.0000	G
12	771494	chr12:641755	641755	G	A	0.00044	0.99956	0.00044	60.00	228.00	1.0000	23.00	23.00	0.0018	G
12	771546	chr12:641807	641807	G	A	0.00025	0.99975	0.00025	60.00	210.00	1.0000	17.00	17.00	0.0120	G
12	771552	chr12:641813	641813	C	G	0.15813	0.84187	0.15813	59.96	207.76	0.2870	13.11	46.20	0.0388	C
12	771596	chr12:641857	641857	G	C	0.00041	0.99959	0.00041	60.00	191.67	1.0000	23.59	23.59	0.0008	G
12	771600	chr12:641861	641861	C	G	0.00044	0.99956	0.00044	59.00	165.00	1.0000	32.00	32.00	0.0009	C
12	771610	chr12:641871	641871	T	C	0.00025	0.99975	0.00025	60.00	118.00	1.0000	17.00	17.00	0.0005	T
12	771677	chr12:641938	641938	G	C	0.18886	0.81114	0.18886	60.00	224.54	0.1570	36.36	81.59	0.0016	G
12	771743	chr12:642004	642004	A	T	0.00025	0.99975	0.00025	60.00	228.00	1.0000	45.00	45.00	0.0005	A
12	771785	chr12:642046	642046	G	A	0.24550	0.75450	0.24550	60.00	215.09	0.1876	18.47	61.75	0.0073	G

12	771790	chr12:642051	642051	G	A	0.17981	0.82019	0.17981	60.00	215.10	0.6639	17.05	59.07	0.0048 A
12	771800	chr12:642061	642061	A	G	0.00077	0.99923	0.00077	60.00	228.00	1.0000	40.83	72.18	0.0000 A
12	771805	chr12:642066	642066	T	C	0.00039	0.99961	0.00039	60.00	228.00	1.0000	57.00	57.00	0.0000 T
12	771855	chr12:642116	642116	C	A	0.00044	0.99956	0.00044	60.00	104.00	1.0000	20.00	20.00	0.0009 C
12	771877	chr12:642138	642138	C	T	0.00050	0.99950	0.00050	60.00	228.00	1.0000	27.30	38.70	0.0020 C
12	771901	chr12:642162	642162	G	T	0.00025	0.99975	0.00025	60.00	228.00	1.0000	45.00	45.00	0.0035 G
12	771928	chr12:642189	642189	A	G	0.00044	0.99956	0.00044	60.00	228.00	1.0000	38.00	38.00	0.0000 A
12	771933	chr12:642194	642194	G	A	0.00025	0.99975	0.00025	60.00	228.00	1.0000	42.00	42.00	0.0015 G
12	771948	chr12:642209	642209	C	G	0.00039	0.99961	0.00039	60.00	228.00	1.0000	67.00	67.00	0.0000 C
12	772001	chr12:642262	642262	G	A	0.00025	0.99975	0.00025	60.00	228.00	1.0000	36.00	36.00	0.0005 G
12	772039	chr12:642300	642300	C	T	0.00044	0.99956	0.00044	60.00	228.00	1.0000	59.00	59.00	0.0000 C
12	772042	chr12:642303	642303	G	A	0.00090	0.99910	0.00090	60.00	217.78	1.0000	40.77	45.57	0.0011 G
12	772067	chr12:642328	642328	T	C	0.00030	0.99970	0.00030	60.00	228.00	1.0000	58.36	58.36	0.0006 T
12	772106	chr12:642367	642367	C	T	0.00039	0.99961	0.00039	60.00	228.00	1.0000	31.00	31.00	0.0000 C
12	772131	chr12:642392	642392	C	G	0.12998	0.87002	0.12998	59.99	129.51	0.0025	10.00	18.00	0.0689 C
12	772137	chr12:642398	642398	C	A	0.00207	0.99793	0.00207	60.00	56.46	1.0000	10.00	10.00	0.0029 C
12	772211	chr12:642472	642472	A	G	0.00025	0.99975	0.00025	60.00	228.00	1.0000	55.00	55.00	0.0010 A
12	772241	chr12:642502	642502	A	G	0.00048	0.99952	0.00048	60.00	228.00	1.0000	38.32	40.75	0.0025 A
12	772292	chr12:642553	642553	T	G	0.00025	0.99975	0.00025	60.00	133.00	1.0000	11.00	11.00	0.0050 T
12	772302	chr12:642563	642563	T	C	0.00025	0.99975	0.00025	60.00	228.00	1.0000	32.00	32.00	0.0055 T
12	772378	chr12:642639	642639	G	A	0.00050	0.99950	0.00050	60.00	228.00	1.0000	17.35	30.65	0.0050 G
12	772393	chr12:642654	642654	G	C	0.00350	0.99650	0.00350	60.00	222.71	1.0000	23.38	48.78	0.0000 G
12	772413	chr12:642674	642674	G	T	0.00025	0.99975	0.00025	60.00	228.00	1.0000	57.00	57.00	0.0005 G
12	772457	chr12:642718	642718	C	T	0.00050	0.99950	0.00050	60.00	117.00	1.0000	32.53	52.48	0.0040 C
12	772458	chr12:642719	642719	C	T	0.06066	0.93934	0.06066	60.00	224.63	0.2193	24.51	61.87	0.0085 C
12	772531	chr12:642792	642792	C	T	0.00025	0.99975	0.00025	60.00	228.00	1.0000	32.00	32.00	0.0050 C
12	772546	chr12:642807	642807	T	G	0.00077	0.99923	0.00077	60.00	228.00	1.0000	30.08	32.93	0.0008 T
12	772553	chr12:642814	642814	G	A	0.00045	0.99955	0.00045	60.00	228.00	1.0000	16.00	16.00	0.0115 G
12	772570	chr12:642831	642831	C	T	0.00026	0.99974	0.00026	60.00	180.00	1.0000	31.00	31.00	0.0315 C
12	772572	chr12:642833	642833	G	A	0.00026	0.99974	0.00026	60.00	228.00	1.0000	55.00	55.00	0.0339 G
12	772606	chr12:642867	642867	G	A	0.00026	0.99974	0.00026	60.00	74.00	1.0000	20.00	20.00	0.0250 G
12	772619	chr12:642880	642880	T	C	0.00044	0.99956	0.00044	60.00	228.00	1.0000	15.00	15.00	0.0027 T
12	772624	chr12:642885	642885	G	T	0.00044	0.99956	0.00044	60.00	228.00	1.0000	33.00	33.00	0.0035 G
12	772648	chr12:642909	642909	C	T	0.00026	0.99974	0.00026	60.00	63.00	1.0000	10.00	10.00	0.0379 C

12	772656	chr12:642917	642917	A	T	0.00717	0.99283	0.00717	60.00	194.17	0.0540	14.09	44.28	0.0271	A
12	772670	chr12:642931	642931	C	T	0.00026	0.99974	0.00026	60.00	228.00	1.0000	24.00	24.00	0.0494	C
12	772673	chr12:642934	642934	C	A	0.36457	0.63543	0.36457	60.00	196.38	0.1294	11.92	49.87	0.0497	C
12	772695	chr12:642956	642956	G	T	0.00170	0.99830	0.00170	60.00	60.24	1.0000	11.01	14.75	0.0319	G
12	772703	chr12:642964	642964	C	A	0.00039	0.99961	0.00039	60.00	81.00	1.0000	17.00	17.00	0.0162	C
12	772710	chr12:642971	642971	G	T	0.00028	0.99972	0.00028	59.00	41.00	1.0000	10.00	10.00	0.1058	G
12	772819	chr12:643080	643080	G	C	0.00261	0.99739	0.00261	60.00	223.44	1.0000	21.89	34.28	0.0065	G
12	772824	chr12:643085	643085	A	G	0.00039	0.99961	0.00039	60.00	228.00	1.0000	40.00	40.00	0.0000	A
12	772848	chr12:643109	643109	C	T	0.00025	0.99975	0.00025	60.00	210.00	1.0000	26.00	26.00	0.0180	C
12	772865	chr12:643126	643126	G	A	0.00045	0.99955	0.00045	60.00	228.00	1.0000	30.00	30.00	0.0088	G
12	772928	chr12:643189	643189	A	G	0.00837	0.99163	0.00837	60.00	224.70	1.0000	16.53	50.72	0.0023	G
12	772960	chr12:643221	643221	G	A	0.00077	0.99923	0.00077	60.00	228.00	1.0000	35.53	55.48	0.0000	G
12	772982	chr12:643243	643243	C	T	0.00039	0.99961	0.00039	60.00	214.00	1.0000	26.00	26.00	0.0000	C
12	773051	chr12:643312	643312	G	A	0.01442	0.98558	0.01442	60.00	226.85	1.0000	36.62	69.67	0.0004	G
12	773056	chr12:643317	643317	C	T	0.00039	0.99961	0.00039	60.00	228.00	1.0000	70.00	70.00	0.0000	C
12	773079	chr12:643340	643340	T	C	0.00044	0.99956	0.00044	60.00	215.00	1.0000	35.00	35.00	0.0000	T
12	773084	chr12:643345	643345	C	A	0.00032	0.99968	0.00032	60.00	228.00	1.0000	58.00	58.00	0.0006	C
12	773097	chr12:643358	643358	T	A	0.00350	0.99650	0.00350	60.00	226.39	1.0000	21.24	50.94	0.0014	t
12	773098	chr12:643359	643359	A	T	0.00375	0.99625	0.00375	60.00	176.50	1.0000	18.34	46.23	0.0074	a
12	773166	chr12:643427	643427	C	G	0.00025	0.99975	0.00025	60.00	228.00	1.0000	43.00	43.00	0.0020	C
12	773174	chr12:643435	643435	C	T	0.00025	0.99975	0.00025	60.00	228.00	1.0000	16.00	16.00	0.0020	c
12	773215	chr12:643476	643476	C	T	0.00025	0.99975	0.00025	60.00	228.00	1.0000	80.00	80.00	0.0015	C
12	773247	chr12:643508	643508	G	C	0.00025	0.99975	0.00025	60.00	228.00	1.0000	64.00	64.00	0.0010	G
12	773262	chr12:643523	643523	A	C	0.19003	0.80997	0.19003	60.00	223.37	0.2313	29.91	76.40	0.0018	A
12	773326	chr12:643587	643587	T	G	0.00025	0.99975	0.00025	60.00	228.00	1.0000	25.00	25.00	0.0035	T
12	773396	chr12:643657	643657	C	A	0.00080	0.99920	0.00080	60.00	81.93	1.0000	15.56	21.66	0.0545	C
12	773402	chr12:643663	643663	C	G	0.00044	0.99956	0.00044	60.00	169.00	1.0000	17.00	17.00	0.0000	C
12	773403	chr12:643664	643664	T	G	0.00025	0.99975	0.00025	60.00	31.00	1.0000	13.00	13.00	0.0040	T
12	773404	chr12:643665	643665	T	A	0.00044	0.99956	0.00044	60.00	159.00	1.0000	15.00	15.00	0.0000	T
12	773419	chr12:643680	643680	G	A	0.00025	0.99975	0.00025	60.00	228.00	1.0000	30.00	30.00	0.0045	G
12	773437	chr12:643698	643698	G	A	0.00039	0.99961	0.00039	60.00	228.00	1.0000	62.00	62.00	0.0000	G
12	773440	chr12:643701	643701	C	A	0.00044	0.99956	0.00044	60.00	228.00	1.0000	31.00	31.00	0.0000	C
12	773456	chr12:643717	643717	C	T	0.37381	0.62619	0.37381	60.00	218.58	0.0006	30.86	76.41	0.0038	C
12	773467	chr12:643728	643728	A	T	0.00025	0.99975	0.00025	60.00	228.00	1.0000	55.00	55.00	0.0010	A

12	773489	chr12:643750	643750	G	T	0.00044	0.99956	0.00044	60.00	228.00	1.0000	26.00	26.00	0.0009 G
12	773510	chr12:643771	643771	G	A	0.00025	0.99975	0.00025	60.00	228.00	1.0000	18.00	18.00	0.0040 G
12	773518	chr12:643779	643779	G	A	0.00039	0.99961	0.00039	60.00	218.00	1.0000	35.00	35.00	0.0000 G
12	773525	chr12:643786	643786	C	T	0.00039	0.99961	0.00039	60.00	228.00	1.0000	31.00	31.00	0.0000 C
12	773528	chr12:643789	643789	A	G	0.00045	0.99955	0.00045	60.00	63.00	1.0000	10.00	10.00	0.0088 A
12	773554	chr12:643815	643815	A	T	0.00027	0.99973	0.00027	58.00	41.00	1.0000	10.00	10.00	0.0909 A
12	773692	chr12:643953	643953	A	C	0.00044	0.99956	0.00044	60.00	228.00	1.0000	30.00	30.00	0.0000 A
12	773733	chr12:643994	643994	G	A	0.00077	0.99923	0.00077	60.00	228.00	1.0000	48.00	48.00	0.0000 G
12	773758	chr12:644019	644019	T	G	0.00051	0.99949	0.00051	60.00	52.00	1.0000	13.25	22.75	0.0160 T
12	773761	chr12:644022	644022	A	T	0.00025	0.99975	0.00025	60.00	33.00	1.0000	14.00	14.00	0.0165 A
12	773786	chr12:644047	644047	C	T	0.00041	0.99959	0.00041	60.00	228.00	1.0000	37.81	37.81	0.0000 C
12	773795	chr12:644056	644056	A	T	0.00044	0.99956	0.00044	60.00	228.00	1.0000	32.00	32.00	0.0000 A
12	773815	chr12:644076	644076	C	T	0.00044	0.99956	0.00044	60.00	228.00	1.0000	24.00	24.00	0.0000 C
12	773859	chr12:644120	644120	G	A	0.00039	0.99961	0.00039	60.00	198.00	1.0000	21.00	21.00	0.0000 G
12	773882	chr12:644143	644143	A	G	0.00102	0.99898	0.00102	60.00	228.00	1.0000	34.87	63.44	0.0032 A
12	773891	chr12:644152	644152	G	A	0.00044	0.99956	0.00044	60.00	206.00	1.0000	38.00	38.00	0.0009 G
12	773921	chr12:644182	644182	T	A	0.00039	0.99961	0.00039	60.00	228.00	1.0000	68.00	68.00	0.0000 T
12	773930	chr12:644191	644191	A	T	0.00025	0.99975	0.00025	60.00	228.00	1.0000	76.00	76.00	0.0005 A
12	773946	chr12:644207	644207	A	T	0.00044	0.99956	0.00044	60.00	40.00	1.0000	15.00	15.00	0.0000 A
12	774002	chr12:644263	644263	C	T	0.00050	0.99950	0.00050	60.00	228.00	1.0000	59.05	60.95	0.0000 C
12	774013	chr12:644274	644274	C	T	0.00039	0.99961	0.00039	60.00	228.00	1.0000	67.00	67.00	0.0000 C
12	774016	chr12:644277	644277	G	A	0.00025	0.99975	0.00025	60.00	228.00	1.0000	79.00	79.00	0.0005 G
12	774044	chr12:644305	644305	G	A	0.00039	0.99961	0.00039	60.00	228.00	1.0000	89.00	89.00	0.0000 G
12	774056	chr12:644317	644317	A	G	0.00316	0.99684	0.00316	60.00	228.00	1.0000	39.01	66.93	0.0000 A
12	774079	chr12:644340	644340	C	T	0.00025	0.99975	0.00025	60.00	228.00	1.0000	61.00	61.00	0.0010 C
12	774190	chr12:644451	644451	G	A	0.00077	0.99923	0.00077	60.00	228.00	1.0000	25.05	26.95	0.0000 G
12	774238	chr12:644499	644499	G	C	0.00044	0.99956	0.00044	60.00	228.00	1.0000	28.00	28.00	0.0009 G
12	774259	chr12:644520	644520	A	G	0.00136	0.99864	0.00136	60.00	228.00	1.0000	32.17	49.61	0.0009 A
12	774265	chr12:644526	644526	C	T	0.00025	0.99975	0.00025	60.00	228.00	1.0000	38.00	38.00	0.0015 C
12	774276	chr12:644537	644537	G	A	0.00045	0.99955	0.00045	60.00	228.00	1.0000	34.58	49.89	0.0006 G
12	774311	chr12:644572	644572	C	T	0.00102	0.99898	0.00102	60.00	226.85	1.0000	32.26	51.45	0.0007 C
12	774391	chr12:644652	644652	A	G	0.00039	0.99961	0.00039	60.00	228.00	1.0000	36.00	36.00	0.0000 A
12	774404	chr12:644665	644665	T	C	0.00039	0.99961	0.00039	59.00	221.00	1.0000	16.00	16.00	0.0062 T
12	774405	chr12:644666	644666	A	T	0.00051	0.99949	0.00051	59.00	76.00	1.0000	13.00	13.00	0.1254 A

12	774604	chr12:644865	644865	T	C	0.00025	0.99975	0.00025	60.00	51.00	1.0000	10.00	10.00	0.0175	T
12	774605	chr12:644866	644866	T	C	0.00122	0.99878	0.00122	59.93	221.53	1.0000	15.45	22.25	0.0051	T
12	774619	chr12:644880	644880	T	C	0.00039	0.99961	0.00039	60.00	31.00	1.0000	14.00	14.00	0.0000	T
12	775180	chr12:645441	645441	G	A	0.00049	0.99951	0.00049	60.00	142.00	1.0000	17.00	17.00	0.1060	G
12	775199	chr12:645460	645460	G	A	0.24130	0.75870	0.24130	60.00	206.47	0.0844	15.04	44.69	0.0519	G
12	775261	chr12:645522	645522	T	C	0.00030	0.99970	0.00030	60.00	228.00	1.0000	54.96	54.96	0.0003	T
12	775336	chr12:645597	645597	C	T	0.15643	0.84357	0.15643	60.00	216.20	0.0787	15.88	57.75	0.0038	T
12	775354	chr12:645615	645615	T	A	0.00226	0.99774	0.00226	60.00	222.13	1.0000	19.15	49.20	0.0009	T
12	775373	chr12:645634	645634	T	C	0.00041	0.99959	0.00041	60.00	228.00	1.0000	48.75	48.75	0.0000	T
12	775431	chr12:645692	645692	T	C	0.00080	0.99920	0.00080	60.00	228.00	1.0000	65.01	76.62	0.0000	T
12	775453	chr12:645714	645714	C	G	0.00025	0.99975	0.00025	60.00	228.00	1.0000	71.00	71.00	0.0000	C
12	775509	chr12:645770	645770	T	C	0.00030	0.99970	0.00030	60.00	228.00	1.0000	38.36	38.36	0.0000	T
12	775524	chr12:645785	645785	G	A	0.00048	0.99952	0.00048	60.00	228.00	1.0000	31.79	34.54	0.0010	G
12	775903	chr12:646164	646164	C	T	0.00032	0.99968	0.00032	58.36	228.00	1.0000	23.11	23.11	0.0134	C
12	775904	chr12:646165	646165	G	C	0.00030	0.99970	0.00030	58.60	216.51	1.0000	25.93	25.93	0.0054	g
12	775928	chr12:646189	646189	A	G	0.00747	0.99253	0.00747	59.95	226.25	1.0000	26.21	53.89	0.0025	A
12	775939	chr12:646200	646200	T	C	0.00025	0.99975	0.00025	60.00	228.00	1.0000	54.00	54.00	0.0000	T
12	775954	chr12:646215	646215	C	T	0.00025	0.99975	0.00025	60.00	136.00	1.0000	18.00	18.00	0.0000	C
12	775956	chr12:646217	646217	A	G	0.00039	0.99961	0.00039	60.00	228.00	1.0000	64.00	64.00	0.0000	A
12	775994	chr12:646255	646255	G	A	0.00044	0.99956	0.00044	60.00	228.00	1.0000	52.00	52.00	0.0000	G
12	776031	chr12:646292	646292	T	G	0.00062	0.99938	0.00062	60.00	228.00	1.0000	51.74	53.95	0.0004	T
12	776075	chr12:646336	646336	C	T	0.00051	0.99949	0.00051	60.00	112.00	1.0000	19.03	19.98	0.0205	C
12	776085	chr12:646346	646346	A	C	0.00025	0.99975	0.00025	59.00	228.00	1.0000	70.00	70.00	0.0080	A
12	776097	chr12:646358	646358	A	C	0.13185	0.86815	0.13185	59.99	207.92	0.0109	15.49	51.74	0.0283	A
12	776136	chr12:646397	646397	T	C	0.00039	0.99961	0.00039	60.00	228.00	1.0000	48.00	48.00	0.0000	T
12	776192	chr12:646453	646453	C	T	0.00050	0.99950	0.00050	60.00	228.00	1.0000	54.38	68.63	0.0005	C
12	776204	chr12:646465	646465	C	G	0.00050	0.99950	0.00050	60.00	228.00	1.0000	50.40	65.60	0.0000	C
12	776240	chr12:646501	646501	A	G	0.00025	0.99975	0.00025	60.00	228.00	1.0000	53.00	53.00	0.0000	A
12	776243	chr12:646504	646504	G	A	0.00039	0.99961	0.00039	60.00	228.00	1.0000	74.00	74.00	0.0000	G
12	776267	chr12:646528	646528	C	T	0.00039	0.99961	0.00039	60.00	228.00	1.0000	75.00	75.00	0.0000	C
12	776270	chr12:646531	646531	G	A	0.00048	0.99952	0.00048	60.00	228.00	1.0000	58.43	74.82	0.0000	G
12	776293	chr12:646554	646554	A	G	0.00090	0.99910	0.00090	60.00	228.00	1.0000	53.76	68.29	0.0005	A
12	776300	chr12:646561	646561	G	C	0.00044	0.99956	0.00044	60.00	228.00	1.0000	58.00	58.00	0.0000	G
12	776371	chr12:646632	646632	G	A	0.54446	0.45554	0.45554	60.00	227.90	0.0278	43.77	90.12	0.0025	A

12	776375	chr12:646636	646636	A	C	0.00044	0.99956	0.00044	60.00	228.00	1.0000	59.00	59.00	0.0000	A
12	776436	chr12:646697	646697	T	A	0.00044	0.99956	0.00044	60.00	228.00	1.0000	72.00	72.00	0.0000	T
12	776471	chr12:646732	646732	G	A	0.54501	0.45499	0.45499	60.00	208.61	0.0473	27.33	72.55	0.0023	A
12	776483	chr12:646744	646744	A	C	0.00045	0.99955	0.00045	60.00	228.00	1.0000	50.05	51.99	0.0000	A
12	776485	chr12:646746	646746	C	T	0.00824	0.99176	0.00824	60.00	225.82	1.0000	26.93	72.31	0.0000	C
12	776519	chr12:646780	646780	A	G	0.00064	0.99936	0.00064	60.00	122.91	1.0000	24.45	26.51	0.0045	A
12	776552	chr12:646813	646813	G	A	0.00032	0.99968	0.00032	60.00	228.00	1.0000	53.03	53.03	0.0010	G
12	776622	chr12:646883	646883	G	A	0.00181	0.99819	0.00181	60.00	228.00	1.0000	65.33	78.40	0.0000	G
12	776629	chr12:646890	646890	C	A	0.00090	0.99910	0.00090	60.00	228.00	1.0000	64.26	70.18	0.0000	C
12	776662	chr12:646923	646923	A	G	0.00025	0.99975	0.00025	60.00	228.00	1.0000	46.00	46.00	0.0000	A
12	776703	chr12:646964	646964	G	A	0.00025	0.99975	0.00025	60.00	228.00	1.0000	75.00	75.00	0.0000	G
12	776851	chr12:647112	647112	C	T	0.00025	0.99975	0.00025	59.00	228.00	1.0000	25.00	25.00	0.0015	C
12	776852	chr12:647113	647113	G	A	0.00039	0.99961	0.00039	60.00	228.00	1.0000	44.00	44.00	0.0000	G
12	777246	chr12:647507	647507	C	G	0.00039	0.99961	0.00039	60.00	228.00	1.0000	63.00	63.00	0.0000	C
12	777253	chr12:647514	647514	A	G	0.00025	0.99975	0.00025	60.00	228.00	1.0000	31.00	31.00	0.0010	A
12	777351	chr12:647612	647612	T	C	0.00249	0.99751	0.00249	60.00	224.14	1.0000	17.64	52.14	0.0016	T
12	777385	chr12:647646	647646	A	C	0.00025	0.99975	0.00025	60.00	228.00	1.0000	45.00	45.00	0.0015	A
12	777391	chr12:647652	647652	G	A	0.00025	0.99975	0.00025	60.00	228.00	1.0000	24.00	24.00	0.0015	G
12	777449	chr12:647710	647710	G	T	0.00102	0.99898	0.00102	60.00	228.00	1.0000	37.22	51.90	0.0005	g
12	777466	chr12:647727	647727	C	T	0.00025	0.99975	0.00025	60.00	228.00	1.0000	31.00	31.00	0.0010	C
12	777487	chr12:647748	647748	A	G	0.00039	0.99961	0.00039	60.00	228.00	1.0000	60.00	60.00	0.0000	A
12	777499	chr12:647760	647760	A	G	0.00050	0.99950	0.00050	60.00	228.00	1.0000	44.40	59.60	0.0005	A
12	777512	chr12:647773	647773	C	T	0.00025	0.99975	0.00025	60.00	228.00	1.0000	31.00	31.00	0.0005	C
12	777615	chr12:647876	647876	C	T	0.00025	0.99975	0.00025	60.00	228.00	1.0000	86.00	86.00	0.0000	C
12	777700	chr12:647961	647961	A	C	0.00044	0.99956	0.00044	60.00	228.00	1.0000	43.00	43.00	0.0000	A
12	777720	chr12:647981	647981	A	T	0.00026	0.99974	0.00026	60.00	203.00	1.0000	16.00	16.00	0.0285	A
12	778217	chr12:648478	648478	T	A	0.00077	0.99923	0.00077	60.00	228.00	1.0000	34.13	38.88	0.0000	T
12	778224	chr12:648485	648485	T	C	0.00025	0.99975	0.00025	60.00	181.00	1.0000	30.00	30.00	0.0035	T
12	778228	chr12:648489	648489	G	A	0.00048	0.99952	0.00048	60.00	228.00	1.0000	37.29	45.17	0.0022	G
12	778262	chr12:648523	648523	C	T	0.00044	0.99956	0.00044	60.00	228.00	1.0000	48.00	48.00	0.0000	C
12	778341	chr12:648602	648602	C	T	0.00048	0.99952	0.00048	60.00	228.00	1.0000	54.19	56.61	0.0025	C
12	778354	chr12:648615	648615	G	A	0.00025	0.99975	0.00025	60.00	228.00	1.0000	61.00	61.00	0.0050	G
12	778361	chr12:648622	648622	G	A	0.00025	0.99975	0.00025	60.00	92.00	1.0000	16.00	16.00	0.0080	G
12	778367	chr12:648628	648628	G	A	0.00041	0.99959	0.00041	60.00	228.00	1.0000	36.69	36.69	0.0004	G

12	778380	chr12:648641	648641	T	C	0.00025	0.99975	0.00025	60.00	160.00	1.0000	10.00	10.00	0.0080	T
12	778397	chr12:648658	648658	C	T	0.00039	0.99961	0.00039	60.00	228.00	1.0000	31.00	31.00	0.0015	C
12	778402	chr12:648663	648663	C	T	0.00045	0.99955	0.00045	60.00	150.00	1.0000	10.00	10.00	0.0133	C
12	778475	chr12:648736	648736	A	G	0.37585	0.62415	0.37585	60.00	207.94	0.0026	22.65	59.23	0.0092	A
12	778482	chr12:648743	648743	G	C	0.00025	0.99975	0.00025	60.00	217.00	1.0000	64.00	64.00	0.0025	G
12	778528	chr12:648789	648789	A	G	0.00044	0.99956	0.00044	60.00	228.00	1.0000	36.00	36.00	0.0009	A
12	778537	chr12:648798	648798	C	T	0.00025	0.99975	0.00025	60.00	228.00	1.0000	30.00	30.00	0.0075	C
12	778544	chr12:648805	648805	G	T	0.00025	0.99975	0.00025	60.00	228.00	1.0000	23.00	23.00	0.0205	G
12	778547	chr12:648808	648808	G	C	0.00026	0.99974	0.00026	60.00	228.00	1.0000	21.00	21.00	0.0354	G
12	778842	chr12:649103	649103	G	T	0.00039	0.99961	0.00039	60.00	62.00	1.0000	13.00	13.00	0.0008	G
12	778877	chr12:649138	649138	G	T	0.00044	0.99956	0.00044	60.00	228.00	1.0000	48.00	48.00	0.0000	G
12	778888	chr12:649149	649149	A	G	0.00062	0.99938	0.00062	60.00	228.00	1.0000	61.47	69.00	0.0000	A
12	778890	chr12:649151	649151	T	A	0.00051	0.99949	0.00051	60.00	77.00	1.0000	34.05	35.95	0.0275	T
12	778929	chr12:649190	649190	T	C	0.00067	0.99933	0.00067	60.00	73.51	1.0000	13.55	16.50	0.0531	T
12	778957	chr12:649218	649218	G	A	0.00039	0.99961	0.00039	60.00	228.00	1.0000	64.00	64.00	0.0000	G
12	778978	chr12:649239	649239	C	G	0.00050	0.99950	0.00050	60.00	228.00	1.0000	33.50	52.50	0.0000	C
12	779024	chr12:649285	649285	C	T	0.00068	0.99932	0.00068	60.00	228.00	1.0000	36.46	46.59	0.0002	C
12	779051	chr12:649312	649312	T	C	0.00044	0.99956	0.00044	60.00	228.00	1.0000	29.00	29.00	0.0009	C
12	779074	chr12:649335	649335	C	T	0.00025	0.99975	0.00025	60.00	228.00	1.0000	64.00	64.00	0.0100	C
12	779107	chr12:649368	649368	G	A	0.00039	0.99961	0.00039	60.00	228.00	1.0000	26.00	26.00	0.0000	G
12	779120	chr12:649381	649381	T	G	0.15616	0.84384	0.15616	60.00	217.95	0.1217	18.47	56.74	0.0050	T
12	779138	chr12:649399	649399	T	C	0.00025	0.99975	0.00025	60.00	228.00	1.0000	43.00	43.00	0.0045	-
12	779142	chr12:649403	649403	A	G	0.15193	0.84807	0.15193	60.00	210.68	0.1217	12.47	48.91	0.0143	G
12	779151	chr12:649412	649412	C	A	0.00044	0.99956	0.00044	60.00	60.00	1.0000	10.00	10.00	0.0062	C
12	779158	chr12:649419	649419	C	T	0.00123	0.99877	0.00123	60.00	206.78	1.0000	16.04	27.07	0.0147	C
12	779168	chr12:649429	649429	G	T	0.11340	0.88660	0.11340	60.00	205.25	0.0002	12.11	42.32	0.0730	T
12	779183	chr12:649444	649444	C	T	0.00025	0.99975	0.00025	60.00	228.00	1.0000	21.00	21.00	0.0205	C
12	779724	chr12:649985	649985	G	C	0.00039	0.99961	0.00039	60.00	228.00	1.0000	32.00	32.00	0.0000	G
12	779765	chr12:650026	650026	A	G	0.00044	0.99956	0.00044	60.00	228.00	1.0000	56.00	56.00	0.0009	A
12	779781	chr12:650042	650042	A	G	0.00025	0.99975	0.00025	60.00	228.00	1.0000	62.00	62.00	0.0005	A
12	779823	chr12:650084	650084	G	C	0.00025	0.99975	0.00025	60.00	228.00	1.0000	64.00	64.00	0.0000	G
12	779844	chr12:650105	650105	T	C	0.00039	0.99961	0.00039	60.00	228.00	1.0000	40.00	40.00	0.0000	T
12	779899	chr12:650160	650160	G	A	0.00025	0.99975	0.00025	60.00	228.00	1.0000	75.00	75.00	0.0010	G
12	779906	chr12:650167	650167	G	A	0.15793	0.84207	0.15793	59.93	224.14	0.1217	35.01	77.51	0.0011	A

12	779993	chr12:650254	650254	T	C	0.00159	0.99841	0.00159	60.00	226.38	1.0000	25.33	37.14	0.0074	T
12	780011	chr12:650272	650272	G	A	0.00025	0.99975	0.00025	59.00	193.00	1.0000	16.00	16.00	0.0165	G
12	780623	chr12:650884	650884	C	A	0.00039	0.99961	0.00039	60.00	228.00	1.0000	32.00	32.00	0.0000	C
12	780633	chr12:650894	650894	G	A	0.00050	0.99950	0.00050	60.00	228.00	1.0000	48.28	58.73	0.0000	G
12	780644	chr12:650905	650905	G	A	0.00044	0.99956	0.00044	60.00	228.00	1.0000	63.00	63.00	0.0000	G
12	780673	chr12:650934	650934	G	A	0.00025	0.99975	0.00025	60.00	228.00	1.0000	74.00	74.00	0.0000	G
12	780686	chr12:650947	650947	T	C	0.00039	0.99961	0.00039	60.00	228.00	1.0000	55.00	55.00	0.0000	T
12	780733	chr12:650994	650994	T	A	0.00068	0.99932	0.00068	60.00	223.98	1.0000	33.35	35.60	0.0009	T
12	781062	chr12:651323	651323	G	T	0.00082	0.99918	0.00082	59.32	128.80	1.0000	16.27	19.31	0.0223	G
12	781072	chr12:651333	651333	G	A	0.00039	0.99961	0.00039	60.00	228.00	1.0000	25.00	25.00	0.0008	G
12	781202	chr12:651463	651463	A	G	0.99932	0.00068	0.00068	59.98	247.64	1.0000	56.59	96.42	0.0000	G
12	781228	chr12:651489	651489	A	G	0.00044	0.99956	0.00044	60.00	228.00	1.0000	91.00	91.00	0.0000	A
12	781285	chr12:651546	651546	T	C	0.00030	0.99970	0.00030	60.00	228.00	1.0000	84.64	84.64	0.0000	T
12	781291	chr12:651552	651552	T	C	0.00088	0.99912	0.00088	60.00	228.00	1.0000	46.23	92.78	0.0000	T
12	781415	chr12:651676	651676	G	T	0.00124	0.99876	0.00124	60.00	228.00	1.0000	35.17	70.07	0.0000	G
12	781427	chr12:651688	651688	G	A	0.00025	0.99975	0.00025	60.00	228.00	1.0000	85.00	85.00	0.0000	G
12	781469	chr12:651730	651730	C	G	0.00039	0.99961	0.00039	60.00	228.00	1.0000	76.00	76.00	0.0000	C
12	781479	chr12:651740	651740	G	A	0.00025	0.99975	0.00025	60.00	228.00	1.0000	64.00	64.00	0.0000	G
12	781557	chr12:651818	651818	C	T	0.00044	0.99956	0.00044	60.00	228.00	1.0000	75.00	75.00	0.0000	C
12	781654	chr12:651915	651915	A	G	0.16678	0.83322	0.16678	60.00	230.00	0.1008	57.92	95.70	0.0007	G
12	781682	chr12:651943	651943	A	G	0.00025	0.99975	0.00025	60.00	228.00	1.0000	83.00	83.00	0.0000	A
12	781701	chr12:651962	651962	T	A	0.00025	0.99975	0.00025	59.00	228.00	1.0000	43.00	43.00	0.0000	T
12	781722	chr12:651983	651983	T	A	0.09954	0.90046	0.09954	59.57	226.90	0.0021	29.00	67.58	0.0000	T
12	781724	chr12:651985	651985	A	T	0.06242	0.93758	0.06242	59.70	226.48	0.0422	34.37	70.24	0.0534	a
12	781811	chr12:652072	652072	G	A	0.00025	0.99975	0.00025	60.00	228.00	1.0000	84.00	84.00	0.0000	G
12	781828	chr12:652089	652089	C	T	0.00079	0.99921	0.00079	60.00	228.00	1.0000	77.94	84.24	0.0000	C
12	781871	chr12:652132	652132	G	A	0.00044	0.99956	0.00044	60.00	228.00	1.0000	67.00	67.00	0.0000	G
12	781958	chr12:652219	652219	C	G	0.00048	0.99952	0.00048	60.00	228.00	1.0000	59.09	74.19	0.0000	C
12	781959	chr12:652220	652220	A	T	0.00124	0.99876	0.00124	60.00	228.00	1.0000	54.18	83.30	0.0000	A
12	781987	chr12:652248	652248	C	G	0.00044	0.99956	0.00044	60.00	228.00	1.0000	37.00	37.00	0.0000	C
12	781993	chr12:652254	652254	C	T	0.43043	0.56957	0.43043	59.75	218.82	0.0002	31.24	83.01	0.0025	C
12	781998	chr12:652259	652259	G	A	0.00412	0.99588	0.00412	59.50	228.00	1.0000	43.05	71.46	0.0000	G
12	782122	chr12:652383	652383	T	G	0.00214	0.99786	0.00214	59.08	220.69	1.0000	23.58	35.02	0.0002	T
12	782137	chr12:652398	652398	G	A	0.00025	0.99975	0.00025	58.00	228.00	1.0000	38.00	38.00	0.0000	G

12	782191	chr12:652452	652452	C	T	0.00039	0.99961	0.00039	60.00	228.00	1.0000	48.00	48.00	0.0000	C
12	782213	chr12:652474	652474	C	T	0.00044	0.99956	0.00044	59.00	228.00	1.0000	69.00	69.00	0.0000	C
12	782233	chr12:652494	652494	C	T	0.00044	0.99956	0.00044	60.00	228.00	1.0000	48.00	48.00	0.0000	C
12	782270	chr12:652531	652531	A	T	0.00025	0.99975	0.00025	58.00	142.00	1.0000	21.00	21.00	0.0030	A
12	782335	chr12:652596	652596	A	G	0.00025	0.99975	0.00025	59.00	32.00	1.0000	10.00	10.00	0.0005	A
12	782346	chr12:652607	652607	T	C	0.00102	0.99898	0.00102	59.63	224.91	1.0000	17.71	21.32	0.0000	T
12	782366	chr12:652627	652627	A	G	0.16456	0.83544	0.16456	59.99	221.79	0.0477	33.83	65.88	0.0023	G
12	782370	chr12:652631	652631	A	C	0.00050	0.99950	0.00050	60.00	228.00	1.0000	47.53	67.48	0.0000	A
12	782379	chr12:652640	652640	G	A	0.00050	0.99950	0.00050	60.00	228.00	1.0000	68.58	90.43	0.0000	G
12	782399	chr12:652660	652660	T	G	0.00025	0.99975	0.00025	60.00	228.00	1.0000	69.00	69.00	0.0000	T
12	782475	chr12:652736	652736	A	G	0.00025	0.99975	0.00025	60.00	228.00	1.0000	56.00	56.00	0.0000	A
12	782540	chr12:652801	652801	G	A	0.00025	0.99975	0.00025	60.00	228.00	1.0000	80.00	80.00	0.0000	G
12	782601	chr12:652862	652862	T	G	0.00039	0.99961	0.00039	60.00	228.00	1.0000	70.00	70.00	0.0000	T
12	782615	chr12:652876	652876	C	T	0.00025	0.99975	0.00025	60.00	228.00	1.0000	76.00	76.00	0.0000	C
12	782647	chr12:652908	652908	C	T	0.00044	0.99956	0.00044	60.00	228.00	1.0000	86.00	86.00	0.0000	C
12	782648	chr12:652909	652909	G	A	0.00044	0.99956	0.00044	60.00	228.00	1.0000	64.00	64.00	0.0000	G
12	782713	chr12:652974	652974	G	A	0.00025	0.99975	0.00025	60.00	209.00	1.0000	25.00	25.00	0.0045	G
12	782998	chr12:653259	653259	A	G	0.00055	0.99945	0.00055	60.00	75.52	1.0000	10.67	12.46	0.1337	A
12	782999	chr12:653260	653260	A	G	0.00028	0.99972	0.00028	59.00	220.00	1.0000	14.00	14.00	0.1218	A
12	783029	chr12:653290	653290	T	G	0.00039	0.99961	0.00039	60.00	228.00	1.0000	61.00	61.00	0.0000	T
12	783039	chr12:653300	653300	G	A	0.00044	0.99956	0.00044	60.00	228.00	1.0000	84.00	84.00	0.0000	G
12	783074	chr12:653335	653335	G	A	0.00025	0.99975	0.00025	60.00	228.00	1.0000	80.00	80.00	0.0000	G
12	783124	chr12:653385	653385	C	T	0.00025	0.99975	0.00025	60.00	228.00	1.0000	45.00	45.00	0.0000	C
12	783128	chr12:653389	653389	A	G	0.00025	0.99975	0.00025	60.00	49.00	1.0000	24.00	24.00	0.0000	A
12	783162	chr12:653423	653423	G	A	0.00025	0.99975	0.00025	60.00	228.00	1.0000	63.00	63.00	0.0000	G
12	783189	chr12:653450	653450	G	A	0.00025	0.99975	0.00025	60.00	228.00	1.0000	75.00	75.00	0.0000	G
12	783251	chr12:653512	653512	G	C	0.00025	0.99975	0.00025	60.00	228.00	1.0000	70.00	70.00	0.0000	G
12	783259	chr12:653520	653520	G	A	0.00068	0.99932	0.00068	60.00	228.00	1.0000	62.81	69.30	0.0000	G
12	783350	chr12:653611	653611	A	G	0.15782	0.84218	0.15782	60.00	228.30	0.1768	45.11	88.61	0.0005	A
12	783484	chr12:653745	653745	G	A	0.20108	0.79892	0.20108	60.00	225.82	0.2110	36.66	84.71	0.0009	G
12	783557	chr12:653818	653818	C	T	0.00044	0.99956	0.00044	60.00	228.00	1.0000	45.00	45.00	0.0000	C
12	783625	chr12:653886	653886	G	A	0.00039	0.99961	0.00039	60.00	228.00	1.0000	57.00	57.00	0.0000	G
12	783633	chr12:653894	653894	T	A	0.00025	0.99975	0.00025	60.00	228.00	1.0000	75.00	75.00	0.0005	T
12	783654	chr12:653915	653915	G	T	0.00044	0.99956	0.00044	60.00	228.00	1.0000	73.00	73.00	0.0000	G

12	783700	chr12:653961	653961	G	A	0.00039	0.99961	0.00039	60.00	42.00	1.0000	28.00	28.00	0.0000	G
12	783706	chr12:653967	653967	C	G	0.00025	0.99975	0.00025	60.00	228.00	1.0000	38.00	38.00	0.0005	C
12	783807	chr12:654068	654068	G	A	0.00712	0.99288	0.00712	60.00	228.00	1.0000	35.32	67.82	0.0009	G
12	783840	chr12:654101	654101	G	C	0.00044	0.99956	0.00044	60.00	228.00	1.0000	43.00	43.00	0.0000	G
12	783872	chr12:654133	654133	T	G	0.00025	0.99975	0.00025	60.00	228.00	1.0000	56.00	56.00	0.0000	T
12	783875	chr12:654136	654136	G	A	0.00045	0.99955	0.00045	60.00	228.00	1.0000	75.54	79.18	0.0000	G
12	783956	chr12:654217	654217	A	C	0.07433	0.92567	0.07433	60.00	225.40	0.5875	25.08	69.93	0.0011	A
12	783987	chr12:654248	654248	A	G	0.14366	0.85634	0.14366	60.00	217.60	0.0100	16.52	57.82	0.0333	A
12	784041	chr12:654302	654302	G	A	0.00030	0.99970	0.00030	60.00	228.00	1.0000	35.65	35.65	0.0003	G
12	784043	chr12:654304	654304	T	C	0.16591	0.83409	0.16591	60.00	221.88	0.0482	23.53	67.97	0.0023	C
12	784083	chr12:654344	654344	C	T	0.00025	0.99975	0.00025	59.00	228.00	1.0000	21.00	21.00	0.0040	C
12	784319	chr12:654580	654580	C	T	0.00044	0.99956	0.00044	59.00	228.00	1.0000	20.00	20.00	0.0018	C
12	784362	chr12:654623	654623	T	C	0.00025	0.99975	0.00025	60.00	228.00	1.0000	33.00	33.00	0.0010	T
12	784745	chr12:655006	655006	A	G	0.00026	0.99974	0.00026	60.00	53.00	1.0000	10.00	10.00	0.0559	A
12	784783	chr12:655044	655044	C	G	0.00051	0.99949	0.00051	60.00	119.50	1.0000	56.10	59.90	0.0295	C
12	784785	chr12:655046	655046	G	C	0.00025	0.99975	0.00025	60.00	193.00	1.0000	34.00	34.00	0.0015	G
12	784812	chr12:655073	655073	C	T	0.00044	0.99956	0.00044	60.00	228.00	1.0000	81.00	81.00	0.0000	C
12	784813	chr12:655074	655074	G	A	0.00025	0.99975	0.00025	60.00	228.00	1.0000	40.00	40.00	0.0000	G
12	784840	chr12:655101	655101	G	A	0.00039	0.99961	0.00039	60.00	228.00	1.0000	55.00	55.00	0.0000	G
12	784848	chr12:655109	655109	T	A	0.46088	0.53912	0.46088	60.00	211.83	0.0006	33.18	83.22	0.0076	T
12	784859	chr12:655120	655120	T	G	0.00051	0.99949	0.00051	60.00	112.50	1.0000	23.63	47.38	0.0135	T
12	784883	chr12:655144	655144	C	A	0.00429	0.99571	0.00429	60.00	228.00	1.0000	54.46	89.09	0.0000	C
12	784907	chr12:655168	655168	T	C	0.00044	0.99956	0.00044	60.00	228.00	1.0000	54.00	54.00	0.0000	T
12	784920	chr12:655181	655181	C	T	0.00025	0.99975	0.00025	60.00	228.00	1.0000	51.00	51.00	0.0000	C
12	784930	chr12:655191	655191	T	C	0.00050	0.99950	0.00050	60.00	228.00	1.0000	47.15	52.85	0.0000	T
12	784958	chr12:655219	655219	C	T	0.00025	0.99975	0.00025	60.00	228.00	1.0000	61.00	61.00	0.0005	C
12	784989	chr12:655250	655250	G	A	0.00039	0.99961	0.00039	60.00	228.00	1.0000	52.00	52.00	0.0000	G
12	785057	chr12:655318	655318	A	G	0.00025	0.99975	0.00025	60.00	228.00	1.0000	69.00	69.00	0.0005	A
12	785100	chr12:655361	655361	C	T	0.00032	0.99968	0.00032	60.00	228.00	1.0000	66.86	66.86	0.0003	C
12	785137	chr12:655398	655398	A	C	0.00075	0.99925	0.00075	60.00	228.00	1.0000	46.35	57.75	0.0010	A
12	785162	chr12:655423	655423	C	T	0.00044	0.99956	0.00044	60.00	228.00	1.0000	54.00	54.00	0.0000	C
12	785182	chr12:655443	655443	G	A	0.00025	0.99975	0.00025	60.00	228.00	1.0000	40.00	40.00	0.0005	G
12	785185	chr12:655446	655446	C	T	0.00039	0.99961	0.00039	60.00	228.00	1.0000	26.00	26.00	0.0000	C
12	785198	chr12:655459	655459	A	G	0.00039	0.99961	0.00039	60.00	228.00	1.0000	31.00	31.00	0.0000	A

12	785468	chr12:655729	655729	T	G	0.79211	0.20789	0.20789	60.00	165.70	0.0021	14.86	60.90	0.0242	T
12	785489	chr12:655750	655750	G	A	0.00032	0.99968	0.00032	60.00	128.71	1.0000	16.15	16.15	0.0183	G
12	785492	chr12:655753	655753	T	C	0.00026	0.99974	0.00026	60.00	99.00	1.0000	12.00	12.00	0.0344	T
12	785511	chr12:655772	655772	A	T	0.00028	0.99972	0.00028	60.00	31.00	1.0000	15.00	15.00	0.1233	A
12	785517	chr12:655778	655778	C	A	0.00028	0.99972	0.00028	60.00	114.00	1.0000	10.00	10.00	0.1088	C
12	785523	chr12:655784	655784	A	G	0.00027	0.99973	0.00027	60.00	46.00	1.0000	10.00	10.00	0.0614	A
12	785587	chr12:655848	655848	C	A	0.00039	0.99961	0.00039	60.00	228.00	1.0000	29.00	29.00	0.0000	C
12	785595	chr12:655856	655856	G	A	0.00025	0.99975	0.00025	60.00	228.00	1.0000	56.00	56.00	0.0015	G
12	785600	chr12:655861	655861	G	C	0.00050	0.99950	0.00050	60.00	228.00	1.0000	45.25	54.75	0.0015	G
12	785619	chr12:655880	655880	A	G	0.00025	0.99975	0.00025	60.00	228.00	1.0000	49.00	49.00	0.0015	A
12	785630	chr12:655891	655891	C	T	0.00248	0.99752	0.00248	60.00	228.00	1.0000	29.92	58.47	0.0002	C
12	785642	chr12:655903	655903	G	A	0.00474	0.99526	0.00474	60.00	222.26	1.0000	20.84	52.63	0.0005	G
12	785644	chr12:655905	655905	C	T	0.00025	0.99975	0.00025	60.00	228.00	1.0000	41.00	41.00	0.0005	C
12	785659	chr12:655920	655920	A	G	0.00039	0.99961	0.00039	60.00	228.00	1.0000	42.00	42.00	0.0000	A
12	785748	chr12:656009	656009	A	G	0.00050	0.99950	0.00050	60.00	228.00	1.0000	38.38	52.63	0.0005	A
12	785763	chr12:656024	656024	T	C	0.00025	0.99975	0.00025	60.00	228.00	1.0000	33.00	33.00	0.0005	T
12	785782	chr12:656043	656043	C	G	0.00039	0.99961	0.00039	60.00	53.00	1.0000	10.00	10.00	0.0000	C
12	785798	chr12:656059	656059	T	C	0.00088	0.99912	0.00088	60.00	58.00	1.0000	13.00	13.00	0.0018	T
12	785809	chr12:656070	656070	C	T	0.00444	0.99556	0.00444	60.00	214.80	1.0000	13.14	40.59	0.0083	C
12	785846	chr12:656107	656107	G	T	0.06399	0.93601	0.06399	60.00	222.87	0.0973	13.55	50.08	0.0072	G
12	785922	chr12:656183	656183	A	T	0.00045	0.99955	0.00045	60.00	228.00	1.0000	30.00	30.00	0.0097	A
12	785929	chr12:656190	656190	C	T	0.00044	0.99956	0.00044	59.00	111.00	1.0000	18.00	18.00	0.0044	C
12	785934	chr12:656195	656195	G	A	0.00034	0.99966	0.00034	60.00	222.19	1.0000	16.42	16.42	0.0146	G
12	785948	chr12:656209	656209	G	A	0.00025	0.99975	0.00025	60.00	228.00	1.0000	18.00	18.00	0.0200	G
12	785960	chr12:656221	656221	C	T	0.00039	0.99961	0.00039	60.00	228.00	1.0000	27.00	27.00	0.0015	C
12	785973	chr12:656234	656234	C	T	0.00044	0.99956	0.00044	60.00	162.00	1.0000	23.00	23.00	0.0000	C
12	786007	chr12:656268	656268	C	G	0.00025	0.99975	0.00025	60.00	228.00	1.0000	37.00	37.00	0.0015	C
12	786051	chr12:656312	656312	T	C	0.00102	0.99898	0.00102	60.00	228.00	1.0000	37.37	49.28	0.0007	T
12	786059	chr12:656320	656320	T	C	0.00044	0.99956	0.00044	60.00	228.00	1.0000	35.00	35.00	0.0000	T
12	786072	chr12:656333	656333	C	T	0.00025	0.99975	0.00025	60.00	228.00	1.0000	59.00	59.00	0.0010	C
12	786074	chr12:656335	656335	A	G	0.00039	0.99961	0.00039	60.00	228.00	1.0000	48.00	48.00	0.0000	A
12	786094	chr12:656355	656355	G	C	0.00025	0.99975	0.00025	60.00	228.00	1.0000	31.00	31.00	0.0015	G
12	786170	chr12:656431	656431	A	G	0.00113	0.99887	0.00113	60.00	228.00	1.0000	45.35	58.92	0.0002	A
12	786182	chr12:656443	656443	A	G	0.00044	0.99956	0.00044	60.00	228.00	1.0000	50.00	50.00	0.0000	A

12	786191	chr12:656452	656452	C	T	0.00025	0.99975	0.00025	60.00	228.00	1.0000	71.00	71.00	0.0005	C
12	786204	chr12:656465	656465	G	A	0.00350	0.99650	0.00350	60.00	228.00	1.0000	41.84	78.28	0.0002	G
12	786225	chr12:656486	656486	C	T	0.00044	0.99956	0.00044	60.00	228.00	1.0000	53.00	53.00	0.0000	C
12	786238	chr12:656499	656499	A	G	0.55036	0.44964	0.44964	60.00	203.85	0.0760	21.95	70.02	0.0097	A
12	786250	chr12:656511	656511	A	T	0.00044	0.99956	0.00044	60.00	228.00	1.0000	61.00	61.00	0.0000	A
12	786260	chr12:656521	656521	G	C	0.00045	0.99955	0.00045	60.00	225.51	1.0000	52.91	57.08	0.0005	G
12	786271	chr12:656532	656532	A	G	0.00044	0.99956	0.00044	60.00	228.00	1.0000	64.00	64.00	0.0000	A
12	786312	chr12:656573	656573	A	G	0.00767	0.99233	0.00767	60.00	228.00	1.0000	25.24	67.09	0.0002	a
12	786323	chr12:656584	656584	C	T	0.00039	0.99961	0.00039	60.00	228.00	1.0000	35.00	35.00	0.0000	C
12	786325	chr12:656586	656586	T	C	0.55120	0.44880	0.44880	60.00	201.71	0.1685	24.61	73.19	0.0038	T
12	786379	chr12:656640	656640	G	A	0.00025	0.99975	0.00025	60.00	228.00	1.0000	74.00	74.00	0.0000	G
12	786398	chr12:656659	656659	T	G	0.00025	0.99975	0.00025	60.00	228.00	1.0000	42.00	42.00	0.0000	T
12	786415	chr12:656676	656676	C	A	0.00048	0.99952	0.00048	60.00	228.00	1.0000	61.47	69.97	0.0003	C
12	786534	chr12:656795	656795	C	T	0.00226	0.99774	0.00226	60.00	228.00	1.0000	54.98	75.19	0.0000	C
12	786544	chr12:656805	656805	T	C	0.00044	0.99956	0.00044	60.00	228.00	1.0000	51.00	51.00	0.0000	T
12	786588	chr12:656849	656849	T	G	0.00025	0.99975	0.00025	60.00	228.00	1.0000	61.00	61.00	0.0000	T
12	786612	chr12:656873	656873	G	A	0.00158	0.99842	0.00158	60.00	228.00	1.0000	68.88	84.15	0.0000	A
12	786615	chr12:656876	656876	T	C	0.00136	0.99864	0.00136	60.00	228.00	1.0000	51.18	82.70	0.0000	T
12	787607	chr12:657868	657868	A	G	0.01073	0.98927	0.01073	60.00	226.23	1.0000	28.88	56.61	0.0007	A
12	787638	chr12:657899	657899	C	G	0.00032	0.99968	0.00032	60.00	228.00	1.0000	77.11	77.11	0.0000	C
12	787672	chr12:657933	657933	G	A	0.00032	0.99968	0.00032	60.00	228.00	1.0000	80.83	80.83	0.0000	G
12	787692	chr12:657953	657953	G	A	0.00025	0.99975	0.00025	60.00	228.00	1.0000	77.00	77.00	0.0000	G
12	787704	chr12:657965	657965	A	G	0.00039	0.99961	0.00039	60.00	228.00	1.0000	38.00	38.00	0.0000	A
12	787716	chr12:657977	657977	C	T	0.00025	0.99975	0.00025	60.00	228.00	1.0000	84.00	84.00	0.0000	C
12	787725	chr12:657986	657986	G	A	0.00068	0.99932	0.00068	60.00	228.00	1.0000	66.72	74.20	0.0000	G
12	787766	chr12:658027	658027	C	T	0.00108	0.99892	0.00108	60.00	56.84	1.0000	20.86	45.20	0.0177	C
12	787774	chr12:658035	658035	G	A	0.00025	0.99975	0.00025	60.00	197.00	1.0000	68.00	68.00	0.0000	G
12	787832	chr12:658093	658093	C	T	0.00039	0.99961	0.00039	60.00	228.00	1.0000	64.00	64.00	0.0000	C
12	787923	chr12:658184	658184	T	C	0.00384	0.99616	0.00384	60.00	228.00	1.0000	49.04	75.77	0.0000	T
12	787939	chr12:658200	658200	C	T	0.00079	0.99921	0.00079	60.00	228.00	1.0000	41.72	64.54	0.0000	C
12	787949	chr12:658210	658210	G	A	0.00025	0.99975	0.00025	60.00	228.00	1.0000	47.00	47.00	0.0000	G
12	787950	chr12:658211	658211	G	T	0.00050	0.99950	0.00050	60.00	228.00	1.0000	70.28	80.73	0.0000	G
12	787969	chr12:658230	658230	G	T	0.00050	0.99950	0.00050	60.00	228.00	1.0000	60.40	75.60	0.0000	G
12	788024	chr12:658285	658285	C	A	0.00025	0.99975	0.00025	60.00	228.00	1.0000	56.00	56.00	0.0000	C

12	788047	chr12:658308	658308	A	G	0.00039	0.99961	0.00039	60.00	228.00	1.0000	32.00	32.00	0.0000	A
12	788176	chr12:658437	658437	G	A	0.00025	0.99975	0.00025	60.00	228.00	1.0000	74.00	74.00	0.0000	G
12	788210	chr12:658471	658471	T	C	0.51814	0.48186	0.48186	60.00	215.20	0.1532	34.95	82.69	0.0050	T
12	788246	chr12:658507	658507	C	T	0.00044	0.99956	0.00044	60.00	228.00	1.0000	61.00	61.00	0.0000	C
12	788247	chr12:658508	658508	G	A	0.00025	0.99975	0.00025	60.00	228.00	1.0000	34.00	34.00	0.0000	G
12	788253	chr12:658514	658514	C	T	0.00025	0.99975	0.00025	60.00	228.00	1.0000	79.00	79.00	0.0000	C
12	788257	chr12:658518	658518	A	G	0.00068	0.99932	0.00068	60.00	228.00	1.0000	58.20	65.20	0.0000	G
12	788265	chr12:658526	658526	C	T	0.00025	0.99975	0.00025	60.00	228.00	1.0000	45.00	45.00	0.0000	C
12	788327	chr12:658588	658588	C	T	0.00045	0.99955	0.00045	60.00	228.00	1.0000	56.84	64.30	0.0000	C
12	788358	chr12:658619	658619	C	T	0.00039	0.99961	0.00039	60.00	228.00	1.0000	49.00	49.00	0.0000	C
12	788359	chr12:658620	658620	G	A	0.04684	0.95316	0.04684	60.00	226.82	0.2595	38.53	83.52	0.0002	G
12	788393	chr12:658654	658654	A	G	0.00025	0.99975	0.00025	60.00	228.00	1.0000	60.00	60.00	0.0000	A
12	788414	chr12:658675	658675	T	C	0.00025	0.99975	0.00025	60.00	228.00	1.0000	70.00	70.00	0.0000	T
12	788491	chr12:658752	658752	T	C	0.00068	0.99932	0.00068	60.00	228.00	1.0000	54.92	63.69	0.0000	T
12	788531	chr12:658792	658792	G	A	0.00044	0.99956	0.00044	60.00	228.00	1.0000	49.00	49.00	0.0000	G
12	788572	chr12:658833	658833	C	T	0.00032	0.99968	0.00032	60.00	228.00	1.0000	26.39	26.39	0.0003	C
12	788636	chr12:658897	658897	C	T	0.00044	0.99956	0.00044	60.00	228.00	1.0000	33.00	33.00	0.0000	C
12	788647	chr12:658908	658908	C	G	0.00025	0.99975	0.00025	60.00	228.00	1.0000	80.00	80.00	0.0005	C
12	788684	chr12:658945	658945	C	T	0.00025	0.99975	0.00025	60.00	228.00	1.0000	53.00	53.00	0.0005	C
12	788711	chr12:658972	658972	C	T	0.00032	0.99968	0.00032	60.00	228.00	1.0000	57.00	57.00	0.0000	C
12	788776	chr12:659037	659037	A	G	0.00079	0.99921	0.00079	60.00	228.00	1.0000	47.11	53.54	0.0000	A
12	788807	chr12:659068	659068	A	C	0.00025	0.99975	0.00025	60.00	228.00	1.0000	46.00	46.00	0.0000	A
12	788828	chr12:659089	659089	G	T	0.00039	0.99961	0.00039	60.00	228.00	1.0000	70.00	70.00	0.0000	G
12	788839	chr12:659100	659100	G	A	0.00045	0.99955	0.00045	60.00	228.00	1.0000	48.61	55.53	0.0000	G
12	788889	chr12:659150	659150	G	T	0.00492	0.99508	0.00492	60.00	157.16	1.0000	13.90	33.95	0.0369	G
12	789107	chr12:659368	659368	G	A	0.00044	0.99956	0.00044	60.00	228.00	1.0000	69.00	69.00	0.0000	G
12	789127	chr12:659388	659388	A	G	0.00418	0.99582	0.00418	60.00	228.00	1.0000	41.58	81.78	0.0000	A
12	789145	chr12:659406	659406	A	G	0.00044	0.99956	0.00044	60.00	228.00	1.0000	51.00	51.00	0.0000	A
12	789152	chr12:659413	659413	A	C	0.60747	0.39253	0.39253	60.00	187.77	0.1427	23.19	54.73	0.0294	C
12	789429	chr12:659690	659690	G	T	0.00039	0.99961	0.00039	60.00	104.00	1.0000	13.00	13.00	0.0023	G
12	789449	chr12:659710	659710	A	T	0.00039	0.99961	0.00039	60.00	228.00	1.0000	37.00	37.00	0.0000	A
12	789475	chr12:659736	659736	T	C	0.00025	0.99975	0.00025	60.00	228.00	1.0000	53.00	53.00	0.0000	T
12	789484	chr12:659745	659745	A	G	0.00039	0.99961	0.00039	60.00	228.00	1.0000	60.00	60.00	0.0000	A
12	789499	chr12:659760	659760	A	C	0.00044	0.99956	0.00044	60.00	228.00	1.0000	51.00	51.00	0.0000	A

12	789522	chr12:659783	659783	C	T	0.00056	0.99944	0.00056	60.00	228.00	1.0000	52.58	55.64	0.0000	C
12	789538	chr12:659799	659799	C	A	0.60871	0.39129	0.39129	60.00	200.33	0.4522	25.84	69.06	0.0025	A
12	789630	chr12:659891	659891	C	A	0.13195	0.86805	0.13195	60.00	223.09	0.0117	26.87	68.73	0.0011	C
12	789636	chr12:659897	659897	C	T	0.00372	0.99628	0.00372	60.00	228.00	1.0000	31.84	58.76	0.0000	C
12	789651	chr12:659912	659912	T	A	0.00050	0.99950	0.00050	60.00	227.50	1.0000	29.65	54.35	0.0000	T
12	789727	chr12:659988	659988	C	T	0.00047	0.99953	0.00047	59.77	228.00	1.0000	19.18	20.04	0.0295	C
12	790201	chr12:660462	660462	T	C	0.00040	0.99960	0.00040	60.00	195.00	1.0000	12.00	12.00	0.0394	T
12	790246	chr12:660507	660507	G	A	0.00025	0.99975	0.00025	60.00	193.00	1.0000	45.00	45.00	0.0040	G
12	790249	chr12:660510	660510	G	A	0.00044	0.99956	0.00044	60.00	228.00	1.0000	42.00	42.00	0.0000	G
12	790282	chr12:660543	660543	G	A	0.11347	0.88653	0.11347	60.00	218.74	0.0606	19.76	72.41	0.0115	G
12	790303	chr12:660564	660564	A	G	0.00039	0.99961	0.00039	60.00	228.00	1.0000	61.00	61.00	0.0023	A
12	790323	chr12:660584	660584	A	G	0.03191	0.96809	0.03191	60.00	224.70	0.0006	21.86	70.29	0.0027	A
12	790346	chr12:660607	660607	G	C	0.02512	0.97488	0.02512	60.00	225.73	0.1083	17.98	61.06	0.0029	G
12	790361	chr12:660622	660622	C	T	0.00107	0.99893	0.00107	60.00	212.74	1.0000	21.09	32.76	0.0061	C
12	790445	chr12:660706	660706	C	T	0.00025	0.99975	0.00025	60.00	228.00	1.0000	64.00	64.00	0.0010	C
12	790446	chr12:660707	660707	G	A	0.00025	0.99975	0.00025	60.00	228.00	1.0000	60.00	60.00	0.0010	A
12	790471	chr12:660732	660732	C	T	0.00025	0.99975	0.00025	60.00	228.00	1.0000	51.00	51.00	0.0040	C
12	790491	chr12:660752	660752	G	C	0.00025	0.99975	0.00025	60.00	228.00	1.0000	60.00	60.00	0.0040	g
12	790888	chr12:661149	661149	T	C	0.00077	0.99923	0.00077	60.00	228.00	1.0000	71.18	77.83	0.0000	T
12	790892	chr12:661153	661153	A	G	0.00044	0.99956	0.00044	60.00	228.00	1.0000	64.00	64.00	0.0009	A
12	790911	chr12:661172	661172	G	A	0.01276	0.98724	0.01276	60.00	227.55	1.0000	41.75	85.53	0.0005	G
12	790988	chr12:661249	661249	C	T	0.00048	0.99952	0.00048	60.00	228.00	1.0000	49.54	52.97	0.0003	C
12	791001	chr12:661262	661262	T	G	0.00025	0.99975	0.00025	60.00	137.00	1.0000	42.00	42.00	0.0130	T
12	791024	chr12:661285	661285	T	G	0.00025	0.99975	0.00025	60.00	228.00	1.0000	60.00	60.00	0.0000	T
12	791053	chr12:661314	661314	C	T	0.00045	0.99955	0.00045	60.00	178.05	1.0000	51.82	52.97	0.0003	C
12	791054	chr12:661315	661315	G	A	0.00025	0.99975	0.00025	60.00	228.00	1.0000	78.00	78.00	0.0000	G
12	791117	chr12:661378	661378	A	G	0.00025	0.99975	0.00025	60.00	228.00	1.0000	59.00	59.00	0.0005	A
12	791119	chr12:661380	661380	G	C	0.00025	0.99975	0.00025	60.00	228.00	1.0000	91.00	91.00	0.0005	G
12	791134	chr12:661395	661395	C	T	0.00030	0.99970	0.00030	60.00	228.00	1.0000	80.43	80.43	0.0003	C
12	791179	chr12:661440	661440	A	G	0.00970	0.99030	0.00970	60.00	228.00	1.0000	49.76	89.48	0.0000	A
12	791210	chr12:661471	661471	C	G	0.12424	0.87576	0.12424	60.00	228.09	0.0428	40.33	90.27	0.0009	C
12	791275	chr12:661536	661536	A	G	0.00044	0.99956	0.00044	60.00	228.00	1.0000	82.00	82.00	0.0000	A
12	791276	chr12:661537	661537	T	C	0.00418	0.99582	0.00418	60.00	228.00	1.0000	51.82	90.68	0.0000	T
12	791346	chr12:661607	661607	G	A	0.00039	0.99961	0.00039	60.00	228.00	1.0000	69.00	69.00	0.0000	G

12	791356	chr12:661617	661617	A	G	0.00068	0.99932	0.00068	60.00	228.00	1.0000	54.43	61.48	0.0000	A
12	791389	chr12:661650	661650	G	T	0.00044	0.99956	0.00044	60.00	228.00	1.0000	20.00	20.00	0.0027	G
12	791732	chr12:661993	661993	G	A	0.00068	0.99932	0.00068	60.00	228.00	1.0000	43.04	49.33	0.0000	G
12	791784	chr12:662045	662045	A	G	0.00039	0.99961	0.00039	60.00	228.00	1.0000	53.00	53.00	0.0000	A
12	791793	chr12:662054	662054	A	C	0.00025	0.99975	0.00025	60.00	228.00	1.0000	58.00	58.00	0.0005	A
12	791804	chr12:662065	662065	C	T	0.00039	0.99961	0.00039	60.00	228.00	1.0000	58.00	58.00	0.0000	C
12	791805	chr12:662066	662066	G	A	0.13150	0.86850	0.13150	60.00	220.10	0.0231	20.55	56.88	0.0020	g
12	791816	chr12:662077	662077	T	C	0.00178	0.99822	0.00178	60.00	67.25	1.0000	15.38	26.85	0.0062	T
12	791817	chr12:662078	662078	T	C	0.00245	0.99755	0.00245	60.00	66.40	1.0000	10.10	17.70	0.0989	T
12	791832	chr12:662093	662093	T	C	0.00041	0.99959	0.00041	60.00	85.97	1.0000	14.74	14.74	0.0000	T
12	791902	chr12:662163	662163	C	T	0.00051	0.99949	0.00051	60.00	79.00	1.0000	31.05	32.95	0.0300	C
12	791903	chr12:662164	662164	A	G	0.46581	0.53419	0.46581	60.00	189.26	0.1900	16.44	54.00	0.0200	A
12	791970	chr12:662231	662231	C	A	0.00025	0.99975	0.00025	60.00	228.00	1.0000	31.00	31.00	0.0010	C
12	791993	chr12:662254	662254	G	A	0.00039	0.99961	0.00039	60.00	228.00	1.0000	54.00	54.00	0.0000	G
12	792001	chr12:662262	662262	T	C	0.00025	0.99975	0.00025	60.00	88.00	1.0000	28.00	28.00	0.0025	T
12	792016	chr12:662277	662277	C	G	0.00025	0.99975	0.00025	60.00	228.00	1.0000	45.00	45.00	0.0035	C
12	792030	chr12:662291	662291	C	T	0.00030	0.99970	0.00030	60.00	228.00	1.0000	38.79	38.79	0.0003	C
12	792034	chr12:662295	662295	T	C	0.00025	0.99975	0.00025	60.00	228.00	1.0000	58.00	58.00	0.0000	T
12	792055	chr12:662316	662316	T	C	0.00032	0.99968	0.00032	60.00	228.00	1.0000	47.61	47.61	0.0003	T
12	792077	chr12:662338	662338	A	G	0.00048	0.99952	0.00048	60.00	228.00	1.0000	40.89	50.60	0.0006	A
12	792122	chr12:662383	662383	C	T	0.00025	0.99975	0.00025	60.00	228.00	1.0000	33.00	33.00	0.0005	C
12	792141	chr12:662402	662402	T	C	0.00039	0.99961	0.00039	60.00	228.00	1.0000	32.00	32.00	0.0000	T
12	792148	chr12:662409	662409	C	T	0.00039	0.99961	0.00039	60.00	228.00	1.0000	39.00	39.00	0.0000	C
12	792154	chr12:662415	662415	T	C	0.00039	0.99961	0.00039	60.00	228.00	1.0000	41.00	41.00	0.0000	T
12	792239	chr12:662500	662500	A	G	0.00044	0.99956	0.00044	60.00	228.00	1.0000	41.00	41.00	0.0000	A
12	792274	chr12:662535	662535	T	C	0.00025	0.99975	0.00025	60.00	228.00	1.0000	67.00	67.00	0.0000	T
12	792277	chr12:662538	662538	C	T	0.00041	0.99959	0.00041	60.00	228.00	1.0000	57.14	57.14	0.0000	C
12	792777	chr12:663038	663038	A	T	0.00102	0.99898	0.00102	60.00	228.00	1.0000	41.68	58.19	0.0000	A
12	792783	chr12:663044	663044	G	T	0.00044	0.99956	0.00044	60.00	228.00	1.0000	37.00	37.00	0.0000	G
12	792801	chr12:663062	663062	C	G	0.00088	0.99912	0.00088	60.00	228.00	1.0000	31.75	60.25	0.0000	C
12	792854	chr12:663115	663115	G	A	0.00158	0.99842	0.00158	60.00	228.00	1.0000	34.34	56.62	0.0002	G
12	792855	chr12:663116	663116	G	C	0.00084	0.99916	0.00084	60.00	73.66	1.0000	24.79	27.00	0.0928	G
12	792880	chr12:663141	663141	G	A	0.00025	0.99975	0.00025	60.00	228.00	1.0000	60.00	60.00	0.0000	g
12	792956	chr12:663217	663217	C	T	0.60671	0.39329	0.39329	60.00	207.92	0.4136	28.28	76.54	0.0018	T

12	793054	chr12:663315	663315	T	C	0.00025	0.99975	0.00025	60.00	203.00	1.0000	38.00	38.00	0.0000	T
12	793062	chr12:663323	663323	C	T	0.00039	0.99961	0.00039	60.00	228.00	1.0000	60.00	60.00	0.0000	C
12	793089	chr12:663350	663350	T	C	0.00102	0.99898	0.00102	60.00	228.00	1.0000	56.29	69.20	0.0000	T
12	793112	chr12:663373	663373	A	G	0.00135	0.99865	0.00135	60.00	228.00	1.0000	44.70	62.22	0.0000	A
12	793132	chr12:663393	663393	G	A	0.00039	0.99961	0.00039	60.00	228.00	1.0000	40.00	40.00	0.0000	G
12	793182	chr12:663443	663443	C	T	0.00025	0.99975	0.00025	60.00	228.00	1.0000	42.00	42.00	0.0000	C
12	793185	chr12:663446	663446	C	G	0.00025	0.99975	0.00025	60.00	228.00	1.0000	47.00	47.00	0.0000	C
12	793253	chr12:663514	663514	C	T	0.00044	0.99956	0.00044	60.00	228.00	1.0000	33.00	33.00	0.0000	C
12	793255	chr12:663516	663516	G	A	0.00025	0.99975	0.00025	60.00	228.00	1.0000	42.00	42.00	0.0010	G
12	793277	chr12:663538	663538	G	A	0.00116	0.99884	0.00116	60.00	228.00	1.0000	19.30	45.90	0.0000	G
12	793434	chr12:663695	663695	A	T	0.00135	0.99865	0.00135	60.00	228.00	1.0000	57.12	76.59	0.0000	A
12	793455	chr12:663716	663716	C	G	0.00666	0.99334	0.00666	60.00	228.00	1.0000	38.19	77.17	0.0000	C
12	793470	chr12:663731	663731	T	A	0.00025	0.99975	0.00025	60.00	228.00	1.0000	48.00	48.00	0.0000	T
12	793480	chr12:663741	663741	A	G	0.00039	0.99961	0.00039	60.00	228.00	1.0000	27.00	27.00	0.0000	A
12	793549	chr12:663810	663810	C	A	0.00025	0.99975	0.00025	60.00	228.00	1.0000	71.00	71.00	0.0000	C
12	793558	chr12:663819	663819	G	A	0.00062	0.99938	0.00062	60.00	228.00	1.0000	55.55	60.87	0.0000	G
12	793599	chr12:663860	663860	G	A	0.00508	0.99492	0.00508	60.00	227.00	1.0000	32.63	64.18	0.0000	G
12	793737	chr12:663998	663998	G	A	0.00044	0.99956	0.00044	60.00	228.00	1.0000	44.00	44.00	0.0000	G
12	793907	chr12:664168	664168	G	A	0.00025	0.99975	0.00025	59.00	228.00	1.0000	17.00	17.00	0.0045	G
12	793935	chr12:664196	664196	C	T	0.00025	0.99975	0.00025	60.00	228.00	1.0000	60.00	60.00	0.0045	C
12	793945	chr12:664206	664206	C	T	0.00039	0.99961	0.00039	59.00	228.00	1.0000	36.00	36.00	0.0000	c
12	794017	chr12:664278	664278	C	G	0.00025	0.99975	0.00025	60.00	228.00	1.0000	80.00	80.00	0.0000	C
12	794096	chr12:664357	664357	T	C	0.00144	0.99856	0.00144	60.00	228.00	1.0000	37.03	63.56	0.0000	T
12	794104	chr12:664365	664365	C	T	0.00044	0.99956	0.00044	60.00	228.00	1.0000	96.00	96.00	0.0000	C
12	794153	chr12:664414	664414	G	A	0.00035	0.99965	0.00035	60.00	226.31	1.0000	17.07	17.07	0.0340	G
12	794212	chr12:664473	664473	T	C	0.00025	0.99975	0.00025	60.00	228.00	1.0000	16.00	16.00	0.0170	T
12	794250	chr12:664511	664511	T	C	0.00045	0.99955	0.00045	60.00	211.00	1.0000	15.00	15.00	0.0177	T
12	794270	chr12:664531	664531	A	G	0.00039	0.99961	0.00039	60.00	228.00	1.0000	49.00	49.00	0.0039	A
12	794286	chr12:664547	664547	A	G	0.00025	0.99975	0.00025	60.00	228.00	1.0000	40.00	40.00	0.0010	A
12	794315	chr12:664576	664576	C	T	0.00044	0.99956	0.00044	60.00	228.00	1.0000	25.00	25.00	0.0000	C
12	794332	chr12:664593	664593	G	A	0.00025	0.99975	0.00025	60.00	228.00	1.0000	62.00	62.00	0.0000	G
12	794386	chr12:664647	664647	C	T	0.00025	0.99975	0.00025	60.00	228.00	1.0000	37.00	37.00	0.0000	C
12	794406	chr12:664667	664667	A	G	0.00030	0.99970	0.00030	60.00	228.00	1.0000	57.36	57.36	0.0000	A
12	794407	chr12:664668	664668	A	G	0.00025	0.99975	0.00025	60.00	109.00	1.0000	42.00	42.00	0.0095	A

12	794443	chr12:664704	664704	C	T	0.00044	0.99956	0.00044	60.00	228.00	1.0000	27.00	27.00	0.0018 C
12	794489	chr12:664750	664750	T	C	0.00025	0.99975	0.00025	57.00	147.00	1.0000	10.00	10.00	0.0070 T
12	794609	chr12:664870	664870	A	G	0.00043	0.99957	0.00043	60.00	183.00	1.0000	11.00	11.00	0.0965 A
12	794711	chr12:664972	664972	G	A	0.00025	0.99975	0.00025	60.00	228.00	1.0000	44.00	44.00	0.0000 G
12	794718	chr12:664979	664979	A	G	0.00039	0.99961	0.00039	60.00	228.00	1.0000	52.00	52.00	0.0000 A
12	794791	chr12:665052	665052	A	G	0.00032	0.99968	0.00032	60.00	221.17	1.0000	14.20	14.20	0.0038 A
12	794796	chr12:665057	665057	A	C	0.00089	0.99911	0.00089	60.00	78.00	1.0000	20.03	20.98	0.0080 A
12	794861	chr12:665122	665122	C	A	0.01491	0.98509	0.01491	60.00	226.53	0.0645	30.85	64.94	0.0007 C
12	794917	chr12:665178	665178	A	G	0.00025	0.99975	0.00025	60.00	228.00	1.0000	48.00	48.00	0.0000 A
12	795056	chr12:665317	665317	G	A	0.00044	0.99956	0.00044	60.00	228.00	1.0000	25.00	25.00	0.0000 G
12	795105	chr12:665366	665366	T	C	0.00025	0.99975	0.00025	60.00	228.00	1.0000	63.00	63.00	0.0000 T
12	795114	chr12:665375	665375	C	T	0.00044	0.99956	0.00044	60.00	228.00	1.0000	67.00	67.00	0.0000 C
12	795156	chr12:665417	665417	A	C	0.00044	0.99956	0.00044	60.00	228.00	1.0000	42.00	42.00	0.0009 A
12	795174	chr12:665435	665435	C	A	0.00047	0.99953	0.00047	60.00	63.00	1.0000	10.00	10.00	0.1728 C
12	795512	chr12:665773	665773	C	T	0.00045	0.99955	0.00045	60.00	228.00	1.0000	21.00	21.00	0.0239 C
12	795518	chr12:665779	665779	G	A	0.00039	0.99961	0.00039	60.00	228.00	1.0000	36.00	36.00	0.0000 G
12	795634	chr12:665895	665895	G	T	0.00103	0.99897	0.00103	60.00	228.00	1.0000	49.51	64.66	0.0000 G
12	795642	chr12:665903	665903	C	A	0.00039	0.99961	0.00039	60.00	228.00	1.0000	60.00	60.00	0.0000 C
12	795683	chr12:665944	665944	A	G	0.00025	0.99975	0.00025	60.00	228.00	1.0000	80.00	80.00	0.0000 A
12	795686	chr12:665947	665947	G	A	0.00025	0.99975	0.00025	60.00	228.00	1.0000	72.00	72.00	0.0005 G
12	795703	chr12:665964	665964	T	C	0.00039	0.99961	0.00039	60.00	228.00	1.0000	70.00	70.00	0.0000 T
12	795738	chr12:665999	665999	G	A	0.00044	0.99956	0.00044	60.00	156.00	1.0000	39.00	39.00	0.0009 G
12	795746	chr12:666007	666007	A	T	0.00127	0.99873	0.00127	60.00	68.30	1.0000	21.76	34.40	0.0194 A
12	795753	chr12:666014	666014	G	T	0.00044	0.99956	0.00044	60.00	228.00	1.0000	31.00	31.00	0.0009 G
12	795762	chr12:666023	666023	C	A	0.00044	0.99956	0.00044	60.00	228.00	1.0000	45.00	45.00	0.0000 C
12	795792	chr12:666053	666053	A	T	0.00041	0.99959	0.00041	60.00	228.00	1.0000	42.07	42.07	0.0000 A
12	795852	chr12:666113	666113	T	A	0.00044	0.99956	0.00044	60.00	228.00	1.0000	64.00	64.00	0.0000 T
12	795895	chr12:666156	666156	G	A	0.00025	0.99975	0.00025	60.00	228.00	1.0000	69.00	69.00	0.0010 G
12	795983	chr12:666244	666244	A	G	0.13067	0.86933	0.13067	60.00	218.67	0.0094	21.04	65.77	0.0052 G
12	796011	chr12:666272	666272	T	C	0.00609	0.99391	0.00609	60.00	228.00	1.0000	33.60	67.88	0.0002 T
12	796028	chr12:666289	666289	C	T	0.00080	0.99920	0.00080	60.00	228.00	1.0000	36.85	54.68	0.0003 C
12	796116	chr12:666377	666377	A	C	0.00044	0.99956	0.00044	60.00	228.00	1.0000	34.00	34.00	0.0027 A
12	796131	chr12:666392	666392	C	T	0.52548	0.47452	0.47452	60.00	197.78	0.1183	19.46	59.99	0.0081 T
12	796221	chr12:666482	666482	G	A	0.00039	0.99961	0.00039	60.00	224.00	1.0000	41.00	41.00	0.0000 G

12	796239	chr12:666500	666500	T	A	0.00048	0.99952	0.00048	60.00	228.00	1.0000	34.32	46.46	0.0003	T
12	796275	chr12:666536	666536	G	T	0.00039	0.99961	0.00039	60.00	228.00	1.0000	39.00	39.00	0.0000	G
12	796293	chr12:666554	666554	A	G	0.00721	0.99279	0.00721	60.00	72.88	1.0000	10.00	16.63	0.0203	A
12	796302	chr12:666563	666563	T	C	0.00044	0.99956	0.00044	60.00	46.00	1.0000	10.00	10.00	0.0035	T
12	796313	chr12:666574	666574	G	C	0.00044	0.99956	0.00044	60.00	228.00	1.0000	22.00	22.00	0.0000	G
12	796379	chr12:666640	666640	G	A	0.00048	0.99952	0.00048	60.00	228.00	1.0000	50.57	56.40	0.0003	G
12	796502	chr12:666763	666763	G	A	0.00025	0.99975	0.00025	60.00	215.00	1.0000	27.00	27.00	0.0065	G
12	796832	chr12:667093	667093	G	T	0.00044	0.99956	0.00044	60.00	228.00	1.0000	19.00	19.00	0.0000	G
12	796933	chr12:667194	667194	C	T	0.00039	0.99961	0.00039	59.00	228.00	1.0000	37.00	37.00	0.0000	C
12	796934	chr12:667195	667195	G	A	0.00044	0.99956	0.00044	59.00	228.00	1.0000	71.00	71.00	0.0000	G
12	796942	chr12:667203	667203	C	G	0.00025	0.99975	0.00025	60.00	228.00	1.0000	39.00	39.00	0.0000	C
12	796968	chr12:667229	667229	G	A	0.00025	0.99975	0.00025	60.00	228.00	1.0000	42.00	42.00	0.0005	G
12	797002	chr12:667263	667263	T	A	0.00044	0.99956	0.00044	59.00	114.00	1.0000	12.00	12.00	0.0027	T
12	797089	chr12:667350	667350	C	T	0.00061	0.99939	0.00061	59.80	228.00	1.0000	41.03	46.80	0.0003	C
12	797188	chr12:667449	667449	C	G	0.00077	0.99923	0.00077	58.00	228.00	1.0000	27.15	32.85	0.0000	C
12	797193	chr12:667454	667454	A	G	0.00102	0.99898	0.00102	58.77	228.00	1.0000	21.70	33.08	0.0005	A
12	797275	chr12:667536	667536	T	C	0.00663	0.99337	0.00663	57.62	139.15	1.0000	10.00	12.70	0.0009	T
12	797347	chr12:667608	667608	T	A	0.00032	0.99968	0.00032	60.00	228.00	1.0000	47.44	47.44	0.0003	T
12	797398	chr12:667659	667659	T	A	0.00064	0.99936	0.00064	60.00	228.00	1.0000	58.65	65.32	0.0000	T
12	797568	chr12:667829	667829	C	T	0.00039	0.99961	0.00039	59.00	228.00	1.0000	28.00	28.00	0.0000	C
12	797607	chr12:667868	667868	G	T	0.01152	0.98848	0.01152	60.00	227.72	1.0000	27.56	58.73	0.0011	G
12	797613	chr12:667874	667874	A	G	0.00050	0.99950	0.00050	60.00	228.00	1.0000	37.20	44.80	0.0010	A
12	797620	chr12:667881	667881	C	A	0.00133	0.99867	0.00133	59.67	228.00	1.0000	28.80	56.35	0.0000	C
12	797672	chr12:667933	667933	T	C	0.00801	0.99199	0.00801	59.62	227.41	1.0000	21.13	49.97	0.0002	T
12	798022	chr12:668283	668283	A	T	0.00053	0.99947	0.00053	60.00	196.00	1.0000	12.00	12.00	0.1661	a
12	798031	chr12:668292	668292	A	G	0.00039	0.99961	0.00039	60.00	228.00	1.0000	18.00	18.00	0.0031	A
12	798092	chr12:668353	668353	C	T	0.00044	0.99956	0.00044	60.00	228.00	1.0000	84.00	84.00	0.0000	C
12	798129	chr12:668390	668390	T	C	0.00113	0.99887	0.00113	59.74	228.00	1.0000	33.82	45.76	0.0000	T
12	798148	chr12:668409	668409	T	G	0.00025	0.99975	0.00025	60.00	228.00	1.0000	29.00	29.00	0.0000	T
12	798569	chr12:668830	668830	C	T	0.00025	0.99975	0.00025	60.00	228.00	1.0000	54.00	54.00	0.0005	C
12	798592	chr12:668853	668853	G	C	0.00048	0.99952	0.00048	60.00	228.00	1.0000	39.55	57.15	0.0000	G
12	798600	chr12:668861	668861	G	A	0.00075	0.99925	0.00075	60.00	228.00	1.0000	30.80	65.95	0.0000	G
12	798613	chr12:668874	668874	G	C	0.00039	0.99961	0.00039	60.00	228.00	1.0000	69.00	69.00	0.0000	G
12	798645	chr12:668906	668906	C	T	0.00025	0.99975	0.00025	60.00	228.00	1.0000	66.00	66.00	0.0005	C

12	798784	chr12:669045	669045	G	C	0.00045	0.99955	0.00045	59.00	228.00	1.0000	23.00	23.00	0.0274	G
12	798798	chr12:669059	669059	T	A	0.00040	0.99960	0.00040	59.00	228.00	1.0000	19.00	19.00	0.0386	T
12	798853	chr12:669114	669114	G	A	0.00025	0.99975	0.00025	56.00	161.00	1.0000	11.00	11.00	0.0030	G
12	798980	chr12:669241	669241	T	C	0.00025	0.99975	0.00025	60.00	228.00	1.0000	79.00	79.00	0.0000	T
12	798996	chr12:669257	669257	C	T	0.00044	0.99956	0.00044	60.00	228.00	1.0000	76.00	76.00	0.0000	C
12	799012	chr12:669273	669273	C	T	0.00039	0.99961	0.00039	60.00	228.00	1.0000	84.00	84.00	0.0000	C
12	799079	chr12:669340	669340	C	A	0.22482	0.77518	0.22482	59.96	221.45	0.5083	41.07	81.24	0.0027	T
12	799086	chr12:669347	669347	G	A	0.00044	0.99956	0.00044	60.00	228.00	1.0000	64.00	64.00	0.0000	G
12	799090	chr12:669351	669351	T	C	0.00039	0.99961	0.00039	60.00	228.00	1.0000	51.00	51.00	0.0000	T
12	799147	chr12:669408	669408	C	T	0.12901	0.87099	0.12901	59.08	217.26	0.0712	16.95	57.29	0.0020	C
12	799768	chr12:670029	670029	T	G	0.00025	0.99975	0.00025	59.00	228.00	1.0000	27.00	27.00	0.0000	T
12	799769	chr12:670030	670030	T	G	0.00025	0.99975	0.00025	59.00	228.00	1.0000	55.00	55.00	0.0000	T
12	799811	chr12:670072	670072	C	T	0.12755	0.87245	0.12755	59.74	223.23	0.2024	27.41	69.63	0.0029	C
12	799840	chr12:670101	670101	C	T	0.00079	0.99921	0.00079	60.00	228.00	1.0000	41.60	53.02	0.0000	C
12	799849	chr12:670110	670110	T	C	0.00044	0.99956	0.00044	60.00	197.00	1.0000	28.00	28.00	0.0000	T
12	800526	chr12:670787	670787	T	A	0.00025	0.99975	0.00025	60.00	212.00	1.0000	35.00	35.00	0.0000	T
12	800557	chr12:670818	670818	A	G	0.00039	0.99961	0.00039	60.00	137.00	1.0000	16.00	16.00	0.0000	A
12	800575	chr12:670836	670836	A	T	0.00025	0.99975	0.00025	60.00	228.00	1.0000	39.00	39.00	0.0000	A
12	800585	chr12:670846	670846	C	T	0.00216	0.99784	0.00216	59.82	217.68	1.0000	21.04	34.79	0.0063	C
12	801260	chr12:671521	671521	G	C	0.80183	0.19817	0.19817	59.71	195.15	0.0148	28.88	76.51	0.0029	C
12	801316	chr12:671577	671577	G	A	0.00136	0.99864	0.00136	60.00	228.00	1.0000	33.51	49.21	0.0007	G
12	801325	chr12:671586	671586	C	T	0.00039	0.99961	0.00039	59.00	228.00	1.0000	32.00	32.00	0.0000	C
12	801343	chr12:671604	671604	T	G	0.00044	0.99956	0.00044	60.00	228.00	1.0000	27.00	27.00	0.0027	T
12	801349	chr12:671610	671610	C	T	0.00076	0.99924	0.00076	59.87	228.00	1.0000	31.29	47.65	0.0000	C
12	801351	chr12:671612	671612	A	G	0.00025	0.99975	0.00025	60.00	228.00	1.0000	31.00	31.00	0.0000	A
12	801387	chr12:671648	671648	T	C	0.00045	0.99955	0.00045	60.00	154.00	1.0000	15.00	15.00	0.0080	T
12	801398	chr12:671659	671659	G	A	0.00045	0.99955	0.00045	60.00	228.00	1.0000	32.00	32.00	0.0186	G
12	801433	chr12:671694	671694	C	T	0.00048	0.99952	0.00048	59.43	228.00	1.0000	27.70	27.70	0.1337	C
12	801438	chr12:671699	671699	C	T	0.00029	0.99971	0.00029	59.00	200.00	1.0000	15.00	15.00	0.1253	C
12	801478	chr12:671739	671739	G	C	0.11676	0.88324	0.11676	60.00	215.70	0.0098	14.05	50.64	0.0406	G
12	801515	chr12:671776	671776	C	T	0.00145	0.99855	0.00145	59.73	226.93	1.0000	14.39	27.82	0.0045	C
12	801656	chr12:671917	671917	G	A	0.00025	0.99975	0.00025	60.00	228.00	1.0000	62.00	62.00	0.0000	G
12	802006	chr12:672267	672267	C	T	0.00050	0.99950	0.00050	60.00	228.00	1.0000	29.08	31.93	0.0000	C
12	802007	chr12:672268	672268	G	A	0.00039	0.99961	0.00039	60.00	228.00	1.0000	46.00	46.00	0.0000	G

12	802010	chr12:672271	672271	A	C	0.00044	0.99956	0.00044	60.00	79.45	1.0000	35.21	35.21	0.0515	A
12	802065	chr12:672326	672326	T	C	0.00039	0.99961	0.00039	60.00	228.00	1.0000	41.00	41.00	0.0015	T
12	802104	chr12:672365	672365	C	T	0.00044	0.99956	0.00044	60.00	93.00	1.0000	19.00	19.00	0.0000	C
12	802189	chr12:672450	672450	A	C	0.00045	0.99955	0.00045	60.00	202.53	1.0000	32.56	33.42	0.0011	A
12	802222	chr12:672483	672483	T	C	0.10388	0.89612	0.10388	60.00	212.15	0.0021	15.24	41.11	0.0526	T
12	802894	chr12:673155	673155	T	C	0.00093	0.99907	0.00093	59.68	228.00	1.0000	24.28	27.29	0.0264	T
12	803005	chr12:673266	673266	G	C	0.00025	0.99975	0.00025	60.00	228.00	1.0000	51.00	51.00	0.0005	G
12	803031	chr12:673292	673292	G	C	0.00041	0.99959	0.00041	60.00	228.00	1.0000	65.48	65.48	0.0000	G
12	803040	chr12:673301	673301	C	T	0.00044	0.99956	0.00044	60.00	228.00	1.0000	27.00	27.00	0.0000	C
12	803067	chr12:673328	673328	G	A	0.00039	0.99961	0.00039	60.00	228.00	1.0000	83.00	83.00	0.0000	G
12	803133	chr12:673394	673394	G	A	0.00096	0.99904	0.00096	60.00	228.00	1.0000	34.14	44.18	0.0016	A
12	803154	chr12:673415	673415	C	A	0.00044	0.99956	0.00044	60.00	228.00	1.0000	31.00	31.00	0.0035	C
12	803256	chr12:673517	673517	G	A	0.00026	0.99974	0.00026	59.00	228.00	1.0000	22.00	22.00	0.0310	G
12	803312	chr12:673573	673573	G	A	0.00028	0.99972	0.00028	59.00	191.00	1.0000	12.00	12.00	0.1083	G
12	803321	chr12:673582	673582	A	T	0.00091	0.99909	0.00091	59.50	164.50	1.0000	15.03	15.98	0.0327	A
12	803330	chr12:673591	673591	C	T	0.00026	0.99974	0.00026	60.00	228.00	1.0000	22.00	22.00	0.0334	C
12	803340	chr12:673601	673601	C	T	0.00039	0.99961	0.00039	60.00	228.00	1.0000	41.00	41.00	0.0000	C
12	803358	chr12:673619	673619	C	T	0.00044	0.99956	0.00044	60.00	228.00	1.0000	21.00	21.00	0.0035	C
12	803361	chr12:673622	673622	A	T	0.00044	0.99956	0.00044	60.00	228.00	1.0000	23.00	23.00	0.0027	A
12	803486	chr12:673747	673747	T	G	0.00092	0.99908	0.00092	60.00	220.16	1.0000	27.82	38.34	0.0223	T
12	803488	chr12:673749	673749	A	G	0.19887	0.80113	0.19887	60.00	201.35	0.0393	11.85	41.87	0.0433	G
12	803517	chr12:673778	673778	C	G	0.04657	0.95343	0.04657	59.86	159.20	0.0017	10.00	22.03	0.1051	G
12	803545	chr12:673806	673806	G	A	0.00048	0.99952	0.00048	59.71	168.03	1.0000	14.01	14.57	0.0570	G
12	803602	chr12:673863	673863	G	A	0.00046	0.99954	0.00046	58.00	31.00	1.0000	10.00	10.00	0.0353	G
12	803632	chr12:673893	673893	C	G	0.00045	0.99955	0.00045	60.00	228.00	1.0000	26.00	26.00	0.0159	C
12	803633	chr12:673894	673894	C	T	0.00026	0.99974	0.00026	60.00	200.00	1.0000	18.00	18.00	0.0290	C
12	803635	chr12:673896	673896	G	A	0.00077	0.99923	0.00077	60.00	228.00	1.0000	24.08	26.93	0.0000	G
12	803693	chr12:673954	673954	A	C	0.00025	0.99975	0.00025	60.00	228.00	1.0000	46.00	46.00	0.0005	A
12	803799	chr12:674060	674060	T	A	0.00159	0.99841	0.00159	59.94	225.94	1.0000	26.22	52.09	0.0034	G
12	803802	chr12:674063	674063	T	C	0.00044	0.99956	0.00044	60.00	228.00	1.0000	52.00	52.00	0.0018	T
12	803854	chr12:674115	674115	A	G	0.00136	0.99864	0.00136	60.00	219.13	1.0000	19.68	38.78	0.0009	A
12	803957	chr12:674218	674218	T	C	0.00044	0.99956	0.00044	60.00	228.00	1.0000	36.00	36.00	0.0000	T
12	804035	chr12:674296	674296	C	T	0.00025	0.99975	0.00025	60.00	228.00	1.0000	34.00	34.00	0.0000	C
12	804041	chr12:674302	674302	C	A	0.00025	0.99975	0.00025	60.00	228.00	1.0000	52.00	52.00	0.0000	C

12	804051	chr12:674312	674312	T	C	0.00080	0.99920	0.00080	60.00	228.00	1.0000	44.86	58.95	0.0003	T
12	804075	chr12:674336	674336	A	T	0.00068	0.99932	0.00068	60.00	228.00	1.0000	45.35	49.91	0.0023	A
12	804116	chr12:674377	674377	C	T	0.00257	0.99743	0.00257	60.00	216.06	1.0000	14.20	49.33	0.0064	C
12	804117	chr12:674378	674378	G	A	0.00025	0.99975	0.00025	60.00	228.00	1.0000	20.00	20.00	0.0100	g
12	804148	chr12:674409	674409	G	C	0.00045	0.99955	0.00045	60.00	143.00	1.0000	21.00	21.00	0.0106	G
12	804230	chr12:674491	674491	C	A	0.00039	0.99961	0.00039	60.00	228.00	1.0000	39.00	39.00	0.0000	C
12	804255	chr12:674516	674516	A	G	0.00039	0.99961	0.00039	60.00	228.00	1.0000	37.00	37.00	0.0000	A
12	804267	chr12:674528	674528	G	A	0.00045	0.99955	0.00045	60.00	228.00	1.0000	25.00	25.00	0.0124	G
12	804332	chr12:674593	674593	C	T	0.00025	0.99975	0.00025	60.00	145.00	1.0000	18.00	18.00	0.0090	C
12	804357	chr12:674618	674618	C	T	0.00077	0.99923	0.00077	59.50	175.00	1.0000	15.05	16.95	0.0008	C
12	804387	chr12:674648	674648	C	T	0.00076	0.99924	0.00076	57.69	106.07	0.0024	10.00	10.00	0.1552	c
12	804397	chr12:674658	674658	C	T	0.00093	0.99907	0.00093	59.00	92.50	1.0000	10.00	10.00	0.0451	C
12	804406	chr12:674667	674667	G	A	0.00041	0.99959	0.00041	59.54	228.00	1.0000	26.87	26.87	0.0053	G
12	804408	chr12:674669	674669	G	T	0.00062	0.99938	0.00062	59.23	228.00	1.0000	30.09	35.82	0.0049	G
12	804417	chr12:674678	674678	C	A	0.00039	0.99961	0.00039	59.00	228.00	1.0000	44.00	44.00	0.0000	c
12	804418	chr12:674679	674679	G	A	0.00025	0.99975	0.00025	59.00	228.00	1.0000	37.00	37.00	0.0100	G
12	804421	chr12:674682	674682	T	G	0.00124	0.99876	0.00124	59.49	227.64	1.0000	22.64	37.16	0.0000	T
12	804428	chr12:674689	674689	G	C	0.20315	0.79685	0.20315	59.81	210.12	0.0188	15.36	64.73	0.0265	-
12	804431	chr12:674692	674692	C	A	0.00025	0.99975	0.00025	60.00	228.00	1.0000	49.00	49.00	0.0080	C
12	804441	chr12:674702	674702	T	C	0.07645	0.92355	0.07645	59.79	213.86	0.4989	15.39	59.17	0.0110	T
12	804455	chr12:674716	674716	C	T	0.00088	0.99912	0.00088	60.00	228.00	1.0000	56.15	61.85	0.0000	C
12	804463	chr12:674724	674724	C	T	0.03235	0.96765	0.03235	59.35	226.46	0.0661	19.82	62.55	0.0058	C
12	804477	chr12:674738	674738	C	T	0.00025	0.99975	0.00025	59.00	63.00	1.0000	25.00	25.00	0.0165	C
12	804488	chr12:674749	674749	G	A	0.00139	0.99861	0.00139	59.78	104.35	1.0000	13.60	20.59	0.0177	G
12	804499	chr12:674760	674760	C	T	0.00133	0.99867	0.00133	59.67	225.67	1.0000	11.45	43.75	0.0009	C
12	804500	chr12:674761	674761	G	A	0.00221	0.99779	0.00221	59.60	203.20	1.0000	10.60	48.90	0.0018	g
12	804531	chr12:674792	674792	C	T	0.00064	0.99936	0.00064	59.36	228.00	1.0000	26.10	30.23	0.0045	C
12	804552	chr12:674813	674813	G	A	0.00768	0.99232	0.00768	59.47	224.33	1.0000	19.85	65.79	0.0005	G
12	804557	chr12:674818	674818	T	C	0.43037	0.56963	0.43037	59.95	210.60	0.5843	25.25	75.58	0.0114	t
12	804576	chr12:674837	674837	C	T	0.00025	0.99975	0.00025	60.00	228.00	1.0000	48.00	48.00	0.0005	C
12	804590	chr12:674851	674851	A	C	0.00044	0.99956	0.00044	60.00	228.00	1.0000	47.00	47.00	0.0000	A
12	804624	chr12:674885	674885	C	T	0.00282	0.99718	0.00282	59.06	228.00	1.0000	37.55	66.69	0.0002	C
12	804627	chr12:674888	674888	G	A	0.00056	0.99944	0.00056	60.00	228.00	1.0000	51.90	59.63	0.0002	G
12	804656	chr12:674917	674917	G	A	0.00030	0.99970	0.00030	60.00	228.00	1.0000	49.61	49.61	0.0006	G

12	804658	chr12:674919	674919	G	A	0.00133	0.99867	0.00133	59.67	228.00	1.0000	26.85	44.90	0.0000	G
12	804691	chr12:674952	674952	C	T	0.00080	0.99920	0.00080	60.00	228.00	1.0000	40.43	53.67	0.0000	C
12	804692	chr12:674953	674953	G	A	0.00068	0.99932	0.00068	60.00	228.00	1.0000	57.90	67.32	0.0000	G
12	804812	chr12:675073	675073	A	G	0.01245	0.98755	0.01245	59.87	198.12	1.0000	10.79	29.10	0.0121	A
12	804836	chr12:675097	675097	G	A	0.00030	0.99970	0.00030	60.00	142.55	1.0000	18.03	18.03	0.0018	G
12	804861	chr12:675122	675122	G	A	0.00144	0.99856	0.00144	60.00	228.00	1.0000	29.30	38.81	0.0000	G
12	804874	chr12:675135	675135	A	C	0.00044	0.99956	0.00044	60.00	212.00	1.0000	14.00	14.00	0.0000	A
12	804884	chr12:675145	675145	T	G	0.00025	0.99975	0.00025	60.00	228.00	1.0000	34.00	34.00	0.0020	T
12	804911	chr12:675172	675172	T	C	0.00044	0.99956	0.00044	60.00	49.00	1.0000	10.00	10.00	0.0009	T
12	804979	chr12:675240	675240	G	A	0.00025	0.99975	0.00025	60.00	228.00	1.0000	38.00	38.00	0.0035	G
12	805019	chr12:675280	675280	G	A	0.00039	0.99961	0.00039	60.00	228.00	1.0000	36.00	36.00	0.0008	G
12	805037	chr12:675298	675298	T	A	0.00026	0.99974	0.00026	60.00	46.00	1.0000	10.00	10.00	0.0315	T
12	805047	chr12:675308	675308	T	C	0.00026	0.99974	0.00026	60.00	228.00	1.0000	26.00	26.00	0.0394	T
12	805049	chr12:675310	675310	T	C	0.00039	0.99961	0.00039	59.00	84.00	1.0000	13.00	13.00	0.0015	T
12	805119	chr12:675380	675380	T	C	0.00396	0.99604	0.00396	60.00	228.00	1.0000	21.80	49.89	0.0018	T
12	805120	chr12:675381	675381	T	G	0.00039	0.99961	0.00039	60.00	67.00	1.0000	38.00	38.00	0.0000	T
12	805146	chr12:675407	675407	C	T	0.00032	0.99968	0.00032	60.00	228.00	1.0000	58.61	58.61	0.0003	C
12	805193	chr12:675454	675454	C	T	0.00044	0.99956	0.00044	60.00	228.00	1.0000	47.00	47.00	0.0000	C
12	805196	chr12:675457	675457	C	T	0.06778	0.93222	0.06778	60.00	226.61	0.7290	34.30	73.81	0.0004	C
12	805212	chr12:675473	675473	G	C	0.00025	0.99975	0.00025	60.00	228.00	1.0000	47.00	47.00	0.0005	G
12	805348	chr12:675609	675609	T	G	0.00049	0.99951	0.00049	60.00	129.36	1.0000	43.10	49.11	0.0256	T
12	805349	chr12:675610	675610	T	G	0.00064	0.99936	0.00064	60.00	122.37	1.0000	32.33	45.68	0.0003	T
12	805384	chr12:675645	675645	A	G	0.00260	0.99740	0.00260	60.00	225.16	1.0000	37.02	65.57	0.0002	A
12	805397	chr12:675658	675658	G	A	0.00039	0.99961	0.00039	60.00	228.00	1.0000	55.00	55.00	0.0000	G
12	805398	chr12:675659	675659	A	G	0.00030	0.99970	0.00030	60.00	228.00	1.0000	40.72	40.72	0.0003	A
12	805444	chr12:675705	675705	G	A	0.00025	0.99975	0.00025	60.00	228.00	1.0000	88.00	88.00	0.0005	G
12	805451	chr12:675712	675712	C	T	0.15413	0.84587	0.15413	60.00	226.42	0.0722	36.46	81.97	0.0007	T
12	805513	chr12:675774	675774	C	T	0.00077	0.99923	0.00077	60.00	228.00	1.0000	46.10	49.90	0.0000	C
12	805568	chr12:675829	675829	T	C	0.00025	0.99975	0.00025	60.00	228.00	1.0000	56.00	56.00	0.0010	T
12	805604	chr12:675865	675865	G	A	0.00050	0.99950	0.00050	59.00	228.00	1.0000	48.20	55.80	0.0010	G
12	805619	chr12:675880	675880	T	C	0.00025	0.99975	0.00025	60.00	228.00	1.0000	63.00	63.00	0.0010	T
12	805680	chr12:675941	675941	C	T	0.00025	0.99975	0.00025	60.00	228.00	1.0000	54.00	54.00	0.0005	C
12	805802	chr12:676063	676063	G	A	0.00025	0.99975	0.00025	60.00	228.00	1.0000	43.00	43.00	0.0005	G
12	805860	chr12:676121	676121	G	A	0.00088	0.99912	0.00088	60.00	228.00	1.0000	46.53	66.48	0.0000	G

12	806014	chr12:676275	676275	A	G	0.00025	0.99975	0.00025	60.00	228.00	1.0000	66.00	66.00	0.0000	A
12	806280	chr12:676541	676541	G	A	0.00027	0.99973	0.00027	60.00	228.00	1.0000	16.00	16.00	0.0704	G
12	806347	chr12:676608	676608	T	C	0.00088	0.99912	0.00088	60.00	215.50	1.0000	16.58	38.43	0.0000	T
12	806414	chr12:676675	676675	A	G	0.00039	0.99961	0.00039	60.00	228.00	1.0000	42.00	42.00	0.0015	A
12	806482	chr12:676743	676743	T	C	0.00044	0.99956	0.00044	60.00	108.00	1.0000	13.00	13.00	0.0009	T
12	806491	chr12:676752	676752	C	T	0.00044	0.99956	0.00044	58.00	56.00	1.0000	10.00	10.00	0.0000	C
12	806573	chr12:676834	676834	T	C	0.00080	0.99920	0.00080	60.00	228.00	1.0000	54.92	60.70	0.0000	T
12	806605	chr12:676866	676866	G	A	0.00039	0.99961	0.00039	60.00	228.00	1.0000	49.00	49.00	0.0000	G
12	806626	chr12:676887	676887	G	C	0.00044	0.99956	0.00044	60.00	228.00	1.0000	37.00	37.00	0.0000	G
12	806702	chr12:676963	676963	T	G	0.00025	0.99975	0.00025	60.00	228.00	1.0000	90.00	90.00	0.0000	t
12	806704	chr12:676965	676965	G	A	0.00025	0.99975	0.00025	60.00	228.00	1.0000	70.00	70.00	0.0000	G
12	806743	chr12:677004	677004	C	T	0.00025	0.99975	0.00025	60.00	228.00	1.0000	61.00	61.00	0.0000	C
12	806761	chr12:677022	677022	C	A	0.00025	0.99975	0.00025	60.00	228.00	1.0000	57.00	57.00	0.0000	C
12	806810	chr12:677071	677071	G	A	0.00025	0.99975	0.00025	60.00	228.00	1.0000	75.00	75.00	0.0000	G
12	806844	chr12:677105	677105	A	T	0.00039	0.99961	0.00039	60.00	228.00	1.0000	49.00	49.00	0.0000	A
12	806869	chr12:677130	677130	G	A	0.00039	0.99961	0.00039	60.00	228.00	1.0000	45.00	45.00	0.0000	G
12	806891	chr12:677152	677152	G	A	0.00044	0.99956	0.00044	60.00	228.00	1.0000	92.00	92.00	0.0000	G
12	806913	chr12:677174	677174	G	A	0.00025	0.99975	0.00025	60.00	228.00	1.0000	94.00	94.00	0.0000	G
12	806971	chr12:677232	677232	T	C	0.00135	0.99865	0.00135	60.00	228.00	1.0000	62.46	83.66	0.0000	T
12	807683	chr12:677944	677944	T	C	0.00044	0.99956	0.00044	60.00	228.00	1.0000	58.00	58.00	0.0000	T
12	807688	chr12:677949	677949	A	C	0.00039	0.99961	0.00039	60.00	209.00	1.0000	47.00	47.00	0.0000	A
12	807730	chr12:677991	677991	A	G	0.00508	0.99492	0.00508	60.00	228.00	1.0000	33.87	70.21	0.0002	A
12	807778	chr12:678039	678039	G	A	0.00025	0.99975	0.00025	60.00	226.00	1.0000	38.00	38.00	0.0000	G
12	807785	chr12:678046	678046	G	A	0.00044	0.99956	0.00044	60.00	228.00	1.0000	47.00	47.00	0.0000	G
12	807863	chr12:678124	678124	A	G	0.00030	0.99970	0.00030	60.00	228.00	1.0000	75.36	75.36	0.0000	A
12	807959	chr12:678220	678220	G	A	0.00025	0.99975	0.00025	60.00	228.00	1.0000	34.00	34.00	0.0000	G
12	808000	chr12:678261	678261	C	T	0.00025	0.99975	0.00025	60.00	228.00	1.0000	49.00	49.00	0.0000	C
12	808053	chr12:678314	678314	A	G	0.00039	0.99961	0.00039	60.00	228.00	1.0000	85.00	85.00	0.0000	A
12	808055	chr12:678316	678316	A	G	0.00025	0.99975	0.00025	60.00	228.00	1.0000	76.00	76.00	0.0000	A
12	808056	chr12:678317	678317	G	T	0.00044	0.99956	0.00044	60.00	228.00	1.0000	76.00	76.00	0.0000	G
12	808096	chr12:678357	678357	C	T	0.00044	0.99956	0.00044	60.00	228.00	1.0000	30.00	30.00	0.0000	C
12	808102	chr12:678363	678363	T	C	0.00039	0.99961	0.00039	60.00	228.00	1.0000	21.00	21.00	0.0000	T
12	808877	chr12:679138	679138	T	G	0.00039	0.99961	0.00039	60.00	228.00	1.0000	15.00	15.00	0.0008	T
12	808888	chr12:679149	679149	A	G	0.00045	0.99955	0.00045	60.00	228.00	1.0000	30.28	32.42	0.0007	A

12	808947	chr12:679208	679208	T	C	0.00039	0.99961	0.00039	60.00	228.00	1.0000	75.00	75.00	0.0000	T
12	808954	chr12:679215	679215	A	T	0.00025	0.99975	0.00025	60.00	228.00	1.0000	68.00	68.00	0.0005	A
12	808981	chr12:679242	679242	A	G	0.00135	0.99865	0.00135	60.00	228.00	1.0000	63.14	88.23	0.0000	A
12	809005	chr12:679266	679266	C	T	0.00025	0.99975	0.00025	60.00	228.00	1.0000	34.00	34.00	0.0000	C
12	809014	chr12:679275	679275	C	T	0.00050	0.99950	0.00050	60.00	228.00	1.0000	67.43	83.58	0.0000	C
12	809047	chr12:679308	679308	G	C	0.00039	0.99961	0.00039	60.00	228.00	1.0000	34.00	34.00	0.0000	G
12	809132	chr12:679393	679393	G	C	0.00044	0.99956	0.00044	60.00	228.00	1.0000	76.00	76.00	0.0000	G
12	809181	chr12:679442	679442	A	G	0.00158	0.99842	0.00158	60.00	228.00	1.0000	45.51	59.47	0.0005	A
12	809183	chr12:679444	679444	C	T	0.00025	0.99975	0.00025	60.00	228.00	1.0000	57.00	57.00	0.0000	C
12	809224	chr12:679485	679485	G	A	0.65213	0.34787	0.34787	60.00	214.71	0.0447	32.53	79.73	0.0038	G
12	809246	chr12:679507	679507	T	C	0.00044	0.99956	0.00044	60.00	228.00	1.0000	62.00	62.00	0.0000	T
12	809254	chr12:679515	679515	T	C	0.00032	0.99968	0.00032	60.00	228.00	1.0000	73.28	73.28	0.0000	T
12	809258	chr12:679519	679519	G	A	0.00044	0.99956	0.00044	60.00	228.00	1.0000	83.00	83.00	0.0000	G
12	809321	chr12:679582	679582	C	G	0.00025	0.99975	0.00025	60.00	228.00	1.0000	92.00	92.00	0.0000	C
12	809334	chr12:679595	679595	G	A	0.00025	0.99975	0.00025	60.00	228.00	1.0000	95.00	95.00	0.0000	G
12	809348	chr12:679609	679609	A	G	0.01749	0.98251	0.01749	60.00	227.79	1.0000	38.18	79.62	0.0002	A
12	809371	chr12:679632	679632	G	A	0.00025	0.99975	0.00025	60.00	228.00	1.0000	58.00	58.00	0.0005	G
12	809384	chr12:679645	679645	T	C	0.00025	0.99975	0.00025	60.00	228.00	1.0000	67.00	67.00	0.0010	T
12	809385	chr12:679646	679646	C	T	0.00044	0.99956	0.00044	60.00	228.00	1.0000	63.00	63.00	0.0000	C
12	809423	chr12:679684	679684	A	G	0.00039	0.99961	0.00039	60.00	228.00	1.0000	42.00	42.00	0.0000	A
12	809462	chr12:679723	679723	G	A	0.00044	0.99956	0.00044	60.00	228.00	1.0000	56.00	56.00	0.0000	G
12	809487	chr12:679748	679748	T	C	0.00056	0.99944	0.00056	60.00	228.00	1.0000	51.41	56.02	0.0002	T
12	809573	chr12:679834	679834	C	A	0.00039	0.99961	0.00039	60.00	228.00	1.0000	56.00	56.00	0.0000	C
12	809578	chr12:679839	679839	T	C	0.00044	0.99956	0.00044	60.00	228.00	1.0000	77.00	77.00	0.0000	T
12	809695	chr12:679956	679956	G	T	0.00025	0.99975	0.00025	60.00	228.00	1.0000	77.00	77.00	0.0000	G
12	809704	chr12:679965	679965	G	T	0.07269	0.92731	0.07269	60.00	226.70	0.0373	37.41	82.42	0.0002	G
12	809728	chr12:679989	679989	G	A	0.00064	0.99936	0.00064	60.00	228.00	1.0000	41.88	46.73	0.0003	G
12	809897	chr12:680158	680158	G	A	0.00039	0.99961	0.00039	60.00	228.00	1.0000	82.00	82.00	0.0000	G
12	809929	chr12:680190	680190	A	G	0.00039	0.99961	0.00039	60.00	228.00	1.0000	60.00	60.00	0.0000	A
12	810010	chr12:680271	680271	A	G	0.00091	0.99909	0.00091	59.85	71.42	1.0000	14.97	18.61	0.0052	A
12	810012	chr12:680273	680273	G	C	0.00026	0.99974	0.00026	60.00	31.00	1.0000	18.00	18.00	0.0529	G
12	810051	chr12:680312	680312	G	T	0.00062	0.99938	0.00062	60.00	228.00	1.0000	31.42	37.50	0.0000	G
12	810058	chr12:680319	680319	A	C	0.00053	0.99947	0.00053	60.00	94.00	1.0000	18.33	30.68	0.0549	A
12	810078	chr12:680339	680339	T	A	0.00025	0.99975	0.00025	60.00	228.00	1.0000	51.00	51.00	0.0000	T

12	810163	chr12:680424	680424	T	C	0.00039	0.99961	0.00039	60.00	228.00	1.0000	60.00	60.00	0.0000	T
12	810260	chr12:680521	680521	G	A	0.00135	0.99865	0.00135	60.00	219.94	1.0000	39.94	70.24	0.0002	G
12	810270	chr12:680531	680531	G	A	0.00025	0.99975	0.00025	60.00	228.00	1.0000	61.00	61.00	0.0000	G
12	810283	chr12:680544	680544	T	C	0.00045	0.99955	0.00045	60.00	210.71	1.0000	50.79	53.67	0.0000	T
12	810356	chr12:680617	680617	T	G	0.00044	0.99956	0.00044	60.00	228.00	1.0000	34.00	34.00	0.0000	T
12	810721	chr12:680982	680982	C	T	0.00039	0.99961	0.00039	60.00	228.00	1.0000	17.00	17.00	0.0131	C
12	810722	chr12:680983	680983	G	A	0.00044	0.99956	0.00044	60.00	36.00	1.0000	10.00	10.00	0.0009	A
12	810762	chr12:681023	681023	C	T	0.00025	0.99975	0.00025	60.00	228.00	1.0000	53.00	53.00	0.0005	C
12	810773	chr12:681034	681034	T	G	0.00025	0.99975	0.00025	60.00	228.00	1.0000	53.00	53.00	0.0000	T
12	810804	chr12:681065	681065	C	T	0.00025	0.99975	0.00025	60.00	228.00	1.0000	67.00	67.00	0.0000	C
12	810815	chr12:681076	681076	C	T	0.00025	0.99975	0.00025	60.00	228.00	1.0000	57.00	57.00	0.0000	C
12	810876	chr12:681137	681137	A	G	0.00079	0.99921	0.00079	60.00	228.00	1.0000	35.01	40.03	0.0005	A
12	810984	chr12:681245	681245	A	G	0.00032	0.99968	0.00032	60.00	228.00	1.0000	39.97	39.97	0.0003	A
12	811031	chr12:681292	681292	T	C	0.14177	0.85823	0.14177	60.00	221.60	0.0575	24.22	65.40	0.0027	T
12	811038	chr12:681299	681299	G	A	0.13792	0.86208	0.13792	60.00	220.99	0.0940	26.41	68.68	0.0043	G
12	811063	chr12:681324	681324	G	A	0.00050	0.99950	0.00050	60.00	228.00	1.0000	66.28	76.73	0.0000	G
12	811087	chr12:681348	681348	C	T	0.00039	0.99961	0.00039	60.00	228.00	1.0000	64.00	64.00	0.0000	C
12	811090	chr12:681351	681351	C	T	0.00039	0.99961	0.00039	60.00	228.00	1.0000	35.00	35.00	0.0000	C
12	811104	chr12:681365	681365	A	C	0.00048	0.99952	0.00048	60.00	228.00	1.0000	58.40	60.83	0.0003	A
12	811120	chr12:681381	681381	C	G	0.00050	0.99950	0.00050	60.00	228.00	1.0000	42.35	55.65	0.0000	C
12	811212	chr12:681473	681473	G	A	0.00025	0.99975	0.00025	60.00	228.00	1.0000	75.00	75.00	0.0000	G
12	811296	chr12:681557	681557	G	A	0.00025	0.99975	0.00025	60.00	228.00	1.0000	69.00	69.00	0.0000	G
12	811362	chr12:681623	681623	C	T	0.00039	0.99961	0.00039	60.00	228.00	1.0000	57.00	57.00	0.0000	C
12	811391	chr12:681652	681652	C	T	0.00147	0.99853	0.00147	60.00	228.00	1.0000	48.11	67.95	0.0000	C
12	811400	chr12:681661	681661	G	A	0.00032	0.99968	0.00032	60.00	228.00	1.0000	54.78	54.78	0.0000	G
12	811403	chr12:681664	681664	C	T	0.00039	0.99961	0.00039	60.00	228.00	1.0000	48.00	48.00	0.0000	C
12	811418	chr12:681679	681679	T	C	0.00045	0.99955	0.00045	60.00	228.00	1.0000	56.52	74.98	0.0000	T
12	811452	chr12:681713	681713	G	A	0.00025	0.99975	0.00025	60.00	228.00	1.0000	93.00	93.00	0.0000	G
12	811520	chr12:681781	681781	G	A	0.00025	0.99975	0.00025	60.00	228.00	1.0000	29.00	29.00	0.0000	G
12	811568	chr12:681829	681829	A	G	0.00061	0.99939	0.00061	56.00	123.00	1.0000	10.00	10.00	0.1772	A
12	811796	chr12:682057	682057	T	G	0.00693	0.99307	0.00693	59.92	215.90	1.0000	17.06	38.23	0.0396	T
12	811799	chr12:682060	682060	G	T	0.01167	0.98833	0.01167	59.83	207.48	0.0063	13.50	39.38	0.0795	G
12	811869	chr12:682130	682130	T	G	0.00100	0.99900	0.00100	60.00	112.75	1.0000	28.08	40.33	0.0015	T
12	811896	chr12:682157	682157	T	A	0.00025	0.99975	0.00025	60.00	228.00	1.0000	61.00	61.00	0.0000	T

12	811920	chr12:682181	682181	A	G	0.00025	0.99975	0.00025	60.00	228.00	1.0000	47.00	47.00	0.0000	A
12	811964	chr12:682225	682225	T	C	0.00044	0.99956	0.00044	60.00	228.00	1.0000	67.00	67.00	0.0000	T
12	812022	chr12:682283	682283	G	A	0.00103	0.99897	0.00103	60.00	228.00	1.0000	42.92	54.23	0.0000	G
12	812143	chr12:682404	682404	C	T	0.02257	0.97743	0.02257	60.00	227.56	0.3825	38.27	77.80	0.0000	C
12	812201	chr12:682462	682462	G	A	0.00025	0.99975	0.00025	60.00	228.00	1.0000	71.00	71.00	0.0000	g
12	812227	chr12:682488	682488	T	C	0.00044	0.99956	0.00044	60.00	228.00	1.0000	95.00	95.00	0.0000	T
12	812284	chr12:682545	682545	T	G	0.00025	0.99975	0.00025	60.00	228.00	1.0000	84.00	84.00	0.0000	T
12	812310	chr12:682571	682571	A	G	0.00050	0.99950	0.00050	60.00	228.00	1.0000	69.63	93.38	0.0000	A
12	812343	chr12:682604	682604	C	T	0.00044	0.99956	0.00044	60.00	228.00	1.0000	83.00	83.00	0.0000	C
12	812459	chr12:682720	682720	T	C	0.00260	0.99740	0.00260	60.00	228.00	1.0000	56.53	89.36	0.0000	T
12	812475	chr12:682736	682736	T	C	0.00032	0.99968	0.00032	60.00	228.00	1.0000	51.14	51.14	0.0000	T
12	812760	chr12:683021	683021	G	A	0.00032	0.99968	0.00032	58.38	171.24	1.0000	11.14	11.14	0.0400	G
12	812860	chr12:683121	683121	G	C	0.00044	0.99956	0.00044	60.00	228.00	1.0000	80.00	80.00	0.0000	G
12	812865	chr12:683126	683126	C	G	0.00124	0.99876	0.00124	60.00	228.00	1.0000	58.90	76.45	0.0000	C
12	812905	chr12:683166	683166	A	G	0.00041	0.99959	0.00041	60.00	228.00	1.0000	61.20	61.20	0.0000	A
12	812973	chr12:683234	683234	G	C	0.00032	0.99968	0.00032	60.00	228.00	1.0000	55.94	55.94	0.0000	G
12	813024	chr12:683285	683285	A	C	0.00025	0.99975	0.00025	60.00	228.00	1.0000	55.00	55.00	0.0000	A
12	813045	chr12:683306	683306	A	G	0.00025	0.99975	0.00025	60.00	228.00	1.0000	25.00	25.00	0.0000	A
12	813084	chr12:683345	683345	C	T	0.00185	0.99815	0.00185	60.00	228.00	1.0000	31.64	51.45	0.0000	C
12	813139	chr12:683400	683400	G	T	0.00044	0.99956	0.00044	60.00	228.00	1.0000	98.00	98.00	0.0000	G
12	813158	chr12:683419	683419	G	C	0.00039	0.99961	0.00039	60.00	228.00	1.0000	62.00	62.00	0.0000	G
12	813169	chr12:683430	683430	A	G	0.00044	0.99956	0.00044	60.00	228.00	1.0000	79.00	79.00	0.0000	A
12	813192	chr12:683453	683453	C	T	0.00025	0.99975	0.00025	60.00	228.00	1.0000	69.00	69.00	0.0000	C
12	813197	chr12:683458	683458	A	G	0.00452	0.99548	0.00452	60.00	226.49	1.0000	26.44	76.19	0.0002	A
12	813205	chr12:683466	683466	G	A	0.00039	0.99961	0.00039	60.00	228.00	1.0000	47.00	47.00	0.0000	G
12	813280	chr12:683541	683541	G	A	0.00025	0.99975	0.00025	60.00	228.00	1.0000	69.00	69.00	0.0000	G
12	813293	chr12:683554	683554	A	C	0.00025	0.99975	0.00025	60.00	70.00	1.0000	43.00	43.00	0.0060	A
12	813348	chr12:683609	683609	C	T	0.00124	0.99876	0.00124	60.00	228.00	1.0000	58.74	68.53	0.0000	C
12	813385	chr12:683646	683646	T	G	0.00667	0.99333	0.00667	60.00	227.30	0.0109	35.59	61.19	0.0016	T
12	813411	chr12:683672	683672	A	G	0.16062	0.83938	0.16062	59.57	199.53	0.0001	15.90	37.05	0.1970	G
12	813728	chr12:683989	683989	C	T	0.00039	0.99961	0.00039	60.00	228.00	1.0000	47.00	47.00	0.0000	C
12	813732	chr12:683993	683993	C	T	0.00025	0.99975	0.00025	60.00	228.00	1.0000	60.00	60.00	0.0000	C
12	813752	chr12:684013	684013	C	A	0.00025	0.99975	0.00025	60.00	228.00	1.0000	67.00	67.00	0.0000	C
12	813823	chr12:684084	684084	C	A	0.00044	0.99956	0.00044	60.00	228.00	1.0000	44.00	44.00	0.0000	C

12	813842	chr12:684103	684103	C	T	0.00030	0.99970	0.00030	60.00	228.00	1.0000	44.43	44.43	0.0000	C
12	813866	chr12:684127	684127	G	A	0.00039	0.99961	0.00039	60.00	228.00	1.0000	69.00	69.00	0.0000	G
12	813869	chr12:684130	684130	A	G	0.30591	0.69409	0.30591	60.00	226.29	0.0246	45.79	90.89	0.0139	G
12	813896	chr12:684157	684157	G	A	0.00044	0.99956	0.00044	60.00	228.00	1.0000	68.00	68.00	0.0000	G
12	813909	chr12:684170	684170	G	A	0.00045	0.99955	0.00045	60.00	228.00	1.0000	75.53	79.26	0.0000	G
12	813922	chr12:684183	684183	A	T	0.00056	0.99944	0.00056	60.00	228.00	1.0000	58.52	59.73	0.0000	A
12	813962	chr12:684223	684223	C	G	0.00039	0.99961	0.00039	60.00	228.00	1.0000	50.00	50.00	0.0000	C
12	814030	chr12:684291	684291	G	A	0.00025	0.99975	0.00025	60.00	228.00	1.0000	79.00	79.00	0.0000	G
12	814097	chr12:684358	684358	G	A	0.00025	0.99975	0.00025	60.00	228.00	1.0000	92.00	92.00	0.0000	G
12	814134	chr12:684395	684395	C	T	0.00068	0.99932	0.00068	60.00	228.00	1.0000	82.56	83.94	0.0000	C
12	814141	chr12:684402	684402	A	G	0.00039	0.99961	0.00039	60.00	228.00	1.0000	81.00	81.00	0.0000	A
12	814157	chr12:684418	684418	T	C	0.00039	0.99961	0.00039	60.00	228.00	1.0000	54.00	54.00	0.0000	T
12	814179	chr12:684440	684440	T	C	0.00025	0.99975	0.00025	59.00	228.00	1.0000	33.00	33.00	0.0015	T
12	814187	chr12:684448	684448	T	C	0.00044	0.99956	0.00044	59.00	228.00	1.0000	26.00	26.00	0.0018	t
12	814309	chr12:684570	684570	C	T	0.00039	0.99961	0.00039	60.00	228.00	1.0000	40.00	40.00	0.0000	C
12	814337	chr12:684598	684598	A	G	0.00039	0.99961	0.00039	60.00	228.00	1.0000	41.00	41.00	0.0008	A
12	814829	chr12:685090	685090	T	C	0.00034	0.99966	0.00034	58.45	223.93	1.0000	32.46	32.46	0.0002	T
12	814882	chr12:685143	685143	T	A	0.31032	0.68968	0.31032	60.00	225.16	0.0564	42.12	89.38	0.0029	T
12	814973	chr12:685234	685234	C	T	0.00025	0.99975	0.00025	60.00	228.00	1.0000	75.00	75.00	0.0010	C
12	815021	chr12:685282	685282	T	C	0.00025	0.99975	0.00025	60.00	228.00	1.0000	29.00	29.00	0.0015	T
12	815079	chr12:685340	685340	T	C	0.00040	0.99960	0.00040	57.00	180.00	1.0000	12.00	12.00	0.0285	T
12	815088	chr12:685349	685349	A	G	0.00032	0.99968	0.00032	59.00	228.00	1.0000	38.14	38.14	0.0077	A
12	815091	chr12:685352	685352	A	G	0.00069	0.99931	0.00069	58.40	206.47	1.0000	14.24	20.09	0.0182	A
12	815269	chr12:685530	685530	T	A	0.00030	0.99970	0.00030	60.00	228.00	1.0000	68.36	68.36	0.0000	t
12	815279	chr12:685540	685540	C	A	0.00124	0.99876	0.00124	60.00	228.00	1.0000	71.35	87.74	0.0000	c
12	815428	chr12:685689	685689	A	G	0.00039	0.99961	0.00039	60.00	228.00	1.0000	66.00	66.00	0.0000	a
12	815469	chr12:685730	685730	T	A	0.00124	0.99876	0.00124	59.82	123.43	1.0000	10.15	13.95	0.0054	t
12	815484	chr12:685745	685745	G	T	0.06123	0.93877	0.06123	59.98	217.44	0.3441	13.53	34.82	0.0045	a
12	815610	chr12:685871	685871	T	A	0.00048	0.99952	0.00048	60.00	228.00	1.0000	74.03	83.74	0.0000	T
12	815632	chr12:685893	685893	A	T	0.00039	0.99961	0.00039	60.00	228.00	1.0000	77.00	77.00	0.0000	a
12	815645	chr12:685906	685906	T	C	0.30622	0.69378	0.30622	60.00	228.27	0.0442	46.10	88.43	0.0012	C
12	815695	chr12:685956	685956	G	T	0.00078	0.99922	0.00078	57.80	97.79	1.0000	13.69	15.97	0.0226	g
12	815722	chr12:685983	685983	C	T	0.00025	0.99975	0.00025	60.00	228.00	1.0000	36.00	36.00	0.0030	C
12	816197	chr12:686458	686458	G	A	0.00044	0.99956	0.00044	60.00	228.00	1.0000	38.00	38.00	0.0000	G

12	816382	chr12:686643	686643	G	T	0.00025	0.99975	0.00025	59.00	228.00	1.0000	50.00	50.00	0.0000	G
12	816495	chr12:686756	686756	G	A	0.00088	0.99912	0.00088	60.00	228.00	1.0000	48.23	56.78	0.0000	G
12	816543	chr12:686804	686804	C	T	0.00025	0.99975	0.00025	60.00	228.00	1.0000	67.00	67.00	0.0000	C
12	816601	chr12:686862	686862	A	G	0.00025	0.99975	0.00025	60.00	228.00	1.0000	70.00	70.00	0.0000	A
12	816609	chr12:686870	686870	C	T	0.00088	0.99912	0.00088	60.00	228.00	1.0000	83.18	89.83	0.0000	C
12	816642	chr12:686903	686903	C	T	0.00039	0.99961	0.00039	60.00	228.00	1.0000	65.00	65.00	0.0000	C
12	816662	chr12:686923	686923	T	C	0.00039	0.99961	0.00039	60.00	228.00	1.0000	55.00	55.00	0.0000	T
12	816668	chr12:686929	686929	A	G	0.00025	0.99975	0.00025	60.00	228.00	1.0000	44.00	44.00	0.0000	A
12	816695	chr12:686956	686956	T	C	0.00025	0.99975	0.00025	60.00	72.00	1.0000	13.00	13.00	0.0065	T
12	817488	chr12:687749	687749	C	T	0.25758	0.74242	0.25758	59.97	180.32	0.0000	13.30	33.19	0.1364	C
12	817509	chr12:687770	687770	C	T	0.00044	0.99956	0.00044	60.00	218.00	1.0000	42.00	42.00	0.0000	C
12	817513	chr12:687774	687774	A	G	0.30081	0.69919	0.30081	60.00	211.99	0.0180	29.10	61.76	0.0213	A
12	817521	chr12:687782	687782	G	T	0.00106	0.99894	0.00106	60.00	228.00	1.0000	39.51	62.56	0.0000	G
12	817539	chr12:687800	687800	A	T	0.00039	0.99961	0.00039	60.00	228.00	1.0000	40.00	40.00	0.0000	A
12	817555	chr12:687816	687816	A	T	0.06193	0.93807	0.06193	60.00	225.90	0.4090	31.46	75.11	0.0015	A
12	817560	chr12:687821	687821	T	C	0.30631	0.69369	0.30631	60.00	221.53	0.0564	32.61	79.43	0.0015	T
12	817630	chr12:687891	687891	G	T	0.01253	0.98747	0.01253	60.00	228.00	1.0000	46.50	89.38	0.0000	G
12	817638	chr12:687899	687899	T	C	0.00039	0.99961	0.00039	60.00	228.00	1.0000	74.00	74.00	0.0000	T
12	817732	chr12:687993	687993	G	A	0.00044	0.99956	0.00044	60.00	228.00	1.0000	78.00	78.00	0.0000	G
12	817815	chr12:688076	688076	C	G	0.00025	0.99975	0.00025	59.00	228.00	1.0000	22.00	22.00	0.0005	C
12	817824	chr12:688085	688085	T	C	0.00025	0.99975	0.00025	60.00	139.00	1.0000	10.00	10.00	0.0055	T
12	818415	chr12:688676	688676	C	A	0.00135	0.99865	0.00135	60.00	224.66	1.0000	25.36	44.75	0.0002	C
12	818423	chr12:688684	688684	T	C	0.00044	0.99956	0.00044	60.00	228.00	1.0000	50.00	50.00	0.0009	T
12	818429	chr12:688690	688690	G	A	0.00025	0.99975	0.00025	60.00	228.00	1.0000	46.00	46.00	0.0000	g
12	818485	chr12:688746	688746	A	C	0.00032	0.99968	0.00032	60.00	228.00	1.0000	72.50	72.50	0.0000	A
12	818549	chr12:688810	688810	G	A	0.29423	0.70577	0.29423	60.00	226.66	0.0810	41.94	87.51	0.0025	A
12	818612	chr12:688873	688873	C	T	0.00025	0.99975	0.00025	60.00	228.00	1.0000	50.00	50.00	0.0000	C
12	818704	chr12:688965	688965	A	T	0.00026	0.99974	0.00026	59.00	60.00	1.0000	13.00	13.00	0.0529	A
12	818743	chr12:689004	689004	A	G	0.00096	0.99904	0.00096	59.45	228.00	1.0000	29.46	46.35	0.0010	a
12	818769	chr12:689030	689030	C	T	0.00025	0.99975	0.00025	60.00	228.00	1.0000	70.00	70.00	0.0010	C
12	818807	chr12:689068	689068	A	T	0.00039	0.99961	0.00039	60.00	228.00	1.0000	45.00	45.00	0.0000	A
12	818809	chr12:689070	689070	G	T	0.00044	0.99956	0.00044	60.00	103.00	1.0000	15.00	15.00	0.0000	G
12	818896	chr12:689157	689157	C	T	0.00039	0.99961	0.00039	59.00	228.00	1.0000	58.00	58.00	0.0000	C
12	818904	chr12:689165	689165	G	T	0.00039	0.99961	0.00039	59.00	228.00	1.0000	68.00	68.00	0.0000	G

12	818915	chr12:689176	689176	G	A	0.00025	0.99975	0.00025	59.00	228.00	1.0000	71.00	71.00	0.0000	G
12	818955	chr12:689216	689216	T	C	0.00025	0.99975	0.00025	60.00	228.00	1.0000	69.00	69.00	0.0000	T
12	818961	chr12:689222	689222	G	A	0.00025	0.99975	0.00025	60.00	228.00	1.0000	70.00	70.00	0.0000	G
12	818986	chr12:689247	689247	C	T	0.00025	0.99975	0.00025	60.00	228.00	1.0000	59.00	59.00	0.0000	C
12	818990	chr12:689251	689251	C	T	0.00025	0.99975	0.00025	60.00	228.00	1.0000	44.00	44.00	0.0005	C
12	819051	chr12:689312	689312	A	G	0.00048	0.99952	0.00048	60.00	228.00	1.0000	31.28	45.24	0.0000	A
12	819119	chr12:689380	689380	G	A	0.00025	0.99975	0.00025	60.00	228.00	1.0000	75.00	75.00	0.0000	G
12	820011	chr12:690272	690272	C	A	0.00027	0.99973	0.00027	59.00	78.00	1.0000	13.00	13.00	0.0699	C
12	820023	chr12:690284	690284	G	T	0.00042	0.99958	0.00042	60.00	215.76	1.0000	26.47	26.47	0.0119	G
12	820380	chr12:690641	690641	T	A	0.00049	0.99951	0.00049	59.00	150.00	1.0000	17.00	17.00	0.0936	T
12	820408	chr12:690669	690669	G	A	0.00152	0.99848	0.00152	59.88	223.51	1.0000	32.42	45.61	0.0000	G
12	820500	chr12:690761	690761	C	A	0.00030	0.99970	0.00030	60.00	228.00	1.0000	53.35	53.35	0.0003	C
12	820518	chr12:690779	690779	C	T	0.00025	0.99975	0.00025	60.00	228.00	1.0000	49.00	49.00	0.0005	C
12	820689	chr12:690950	690950	C	A	0.00025	0.99975	0.00025	59.00	228.00	1.0000	56.00	56.00	0.0000	C
12	820700	chr12:690961	690961	T	G	0.00025	0.99975	0.00025	59.00	92.00	1.0000	16.00	16.00	0.0000	T
12	820701	chr12:690962	690962	G	T	0.24437	0.75563	0.24437	59.09	203.03	0.0000	20.43	69.63	0.0569	T
12	820761	chr12:691022	691022	T	G	0.00025	0.99975	0.00025	60.00	228.00	1.0000	34.00	34.00	0.0000	T
12	821165	chr12:691426	691426	T	C	0.00206	0.99794	0.00206	60.00	214.16	1.0000	14.30	33.55	0.0144	T
12	821175	chr12:691436	691436	C	T	0.00145	0.99855	0.00145	60.00	226.29	1.0000	21.02	34.13	0.0025	C
12	821189	chr12:691450	691450	T	C	0.00050	0.99950	0.00050	60.00	174.50	1.0000	20.10	23.90	0.0090	T
12	821198	chr12:691459	691459	A	T	0.00025	0.99975	0.00025	60.00	199.00	1.0000	18.00	18.00	0.0070	A
12	821200	chr12:691461	691461	G	T	0.00025	0.99975	0.00025	60.00	105.00	1.0000	15.00	15.00	0.0055	G
12	821216	chr12:691477	691477	G	A	0.00044	0.99956	0.00044	58.00	228.00	1.0000	13.00	13.00	0.0044	G
12	821225	chr12:691486	691486	T	C	0.00025	0.99975	0.00025	56.00	57.00	1.0000	10.00	10.00	0.0105	T
12	821360	chr12:691621	691621	G	A	0.00116	0.99884	0.00116	60.00	228.00	1.0000	34.50	46.85	0.0000	G
12	821386	chr12:691647	691647	A	G	0.29013	0.70987	0.29013	60.00	219.49	0.0599	31.54	72.43	0.0032	A
12	821406	chr12:691667	691667	G	A	0.00025	0.99975	0.00025	60.00	228.00	1.0000	68.00	68.00	0.0000	G
12	821415	chr12:691676	691676	A	G	0.00039	0.99961	0.00039	60.00	228.00	1.0000	56.00	56.00	0.0000	A
12	821451	chr12:691712	691712	G	A	0.13781	0.86219	0.13781	59.56	227.82	0.1308	48.77	87.89	0.0002	G
12	821454	chr12:691715	691715	C	A	0.00039	0.99961	0.00039	59.00	228.00	1.0000	74.00	74.00	0.0000	C
12	821628	chr12:691889	691889	A	C	0.00047	0.99953	0.00047	60.00	90.85	1.0000	41.21	47.97	0.0349	A
12	821687	chr12:691948	691948	A	G	0.00044	0.99956	0.00044	60.00	228.00	1.0000	53.00	53.00	0.0000	A
12	821760	chr12:692021	692021	G	A	0.00044	0.99956	0.00044	60.00	228.00	1.0000	54.00	54.00	0.0000	G
12	821792	chr12:692053	692053	G	A	0.00044	0.99956	0.00044	60.00	228.00	1.0000	55.00	55.00	0.0000	G

12	821912	chr12:692173	692173	C	G	0.27906	0.72094	0.27906	60.00	220.11	0.0239	31.52	82.10	0.0097	C
12	821914	chr12:692175	692175	G	A	0.00032	0.99968	0.00032	60.00	228.00	1.0000	49.17	49.17	0.0000	G
12	821961	chr12:692222	692222	T	C	0.00025	0.99975	0.00025	60.00	43.00	1.0000	18.00	18.00	0.0010	T
12	821986	chr12:692247	692247	G	A	0.00025	0.99975	0.00025	60.00	214.00	1.0000	37.00	37.00	0.0005	G
12	822344	chr12:692605	692605	G	A	0.00025	0.99975	0.00025	60.00	228.00	1.0000	64.00	64.00	0.0010	G
12	822402	chr12:692663	692663	C	T	0.00039	0.99961	0.00039	60.00	228.00	1.0000	71.00	71.00	0.0000	C
12	822507	chr12:692768	692768	C	G	0.00078	0.99922	0.00078	60.00	183.00	1.0000	10.23	18.78	0.0069	C
12	822508	chr12:692769	692769	A	C	0.00045	0.99955	0.00045	59.00	30.00	1.0000	10.00	10.00	0.0133	A
12	822524	chr12:692785	692785	A	G	0.00039	0.99961	0.00039	59.00	185.00	1.0000	16.00	16.00	0.0131	A
12	822527	chr12:692788	692788	A	C	0.00026	0.99974	0.00026	60.00	105.00	1.0000	15.00	15.00	0.0330	A
12	822533	chr12:692794	692794	A	G	0.00025	0.99975	0.00025	60.00	69.00	1.0000	10.00	10.00	0.0075	A
12	822588	chr12:692849	692849	G	A	0.00025	0.99975	0.00025	60.00	228.00	1.0000	45.00	45.00	0.0000	G
12	822593	chr12:692854	692854	G	A	0.00044	0.99956	0.00044	60.00	228.00	1.0000	40.00	40.00	0.0000	G
12	822601	chr12:692862	692862	G	A	0.00044	0.99956	0.00044	60.00	228.00	1.0000	52.00	52.00	0.0000	G
12	822664	chr12:692925	692925	T	G	0.14671	0.85329	0.14671	60.00	227.92	0.0757	45.98	91.89	0.0009	T
12	822694	chr12:692955	692955	C	T	0.00135	0.99865	0.00135	60.00	228.00	1.0000	41.35	73.81	0.0000	C
12	822710	chr12:692971	692971	G	A	0.00082	0.99918	0.00082	60.00	228.00	1.0000	44.52	66.67	0.0000	G
12	822732	chr12:692993	692993	T	G	0.00025	0.99975	0.00025	60.00	228.00	1.0000	68.00	68.00	0.0000	T
12	822747	chr12:693008	693008	A	G	0.00025	0.99975	0.00025	60.00	228.00	1.0000	52.00	52.00	0.0000	A
12	822906	chr12:693167	693167	A	G	0.00185	0.99815	0.00185	60.00	228.00	1.0000	28.32	56.77	0.0000	G
12	822915	chr12:693176	693176	A	G	0.00185	0.99815	0.00185	60.00	220.54	1.0000	30.75	59.02	0.0000	G
12	822989	chr12:693250	693250	C	T	0.00025	0.99975	0.00025	60.00	228.00	1.0000	64.00	64.00	0.0000	C
12	823035	chr12:693296	693296	C	T	0.00044	0.99956	0.00044	60.00	228.00	1.0000	52.00	52.00	0.0000	C
12	823119	chr12:693380	693380	C	G	0.00044	0.99956	0.00044	60.00	228.00	1.0000	43.00	43.00	0.0000	C
12	823160	chr12:693421	693421	G	A	0.00039	0.99961	0.00039	60.00	228.00	1.0000	46.00	46.00	0.0000	G
12	823185	chr12:693446	693446	C	T	0.00192	0.99808	0.00192	60.00	228.00	1.0000	26.33	62.57	0.0032	T
12	823204	chr12:693465	693465	C	T	0.00025	0.99975	0.00025	60.00	228.00	1.0000	51.00	51.00	0.0010	C
12	823206	chr12:693467	693467	C	T	0.00030	0.99970	0.00030	60.00	228.00	1.0000	49.00	49.00	0.0006	C
12	823230	chr12:693491	693491	G	A	0.00025	0.99975	0.00025	60.00	228.00	1.0000	56.00	56.00	0.0010	G
12	823260	chr12:693521	693521	C	T	0.00044	0.99956	0.00044	60.00	228.00	1.0000	71.00	71.00	0.0000	C
12	823292	chr12:693553	693553	A	G	0.00034	0.99966	0.00034	60.00	68.17	1.0000	44.83	44.83	0.0045	A
12	823293	chr12:693554	693554	A	C	0.00124	0.99876	0.00124	60.00	228.00	1.0000	33.77	56.67	0.0000	A
12	823325	chr12:693586	693586	T	C	0.00135	0.99865	0.00135	60.00	228.00	1.0000	39.14	60.80	0.0000	C
12	823337	chr12:693598	693598	G	T	0.00039	0.99961	0.00039	60.00	228.00	1.0000	47.00	47.00	0.0000	G

12	823906	chr12:694167	694167	G	A	0.00039	0.99961	0.00039	60.00	228.00	1.0000	27.00	27.00	0.0000	G
12	823908	chr12:694169	694169	A	T	0.00025	0.99975	0.00025	60.00	115.00	1.0000	12.00	12.00	0.0065	A
12	823910	chr12:694171	694171	A	T	0.00025	0.99975	0.00025	60.00	95.00	1.0000	12.00	12.00	0.0055	A
12	823922	chr12:694183	694183	G	A	0.00093	0.99907	0.00093	59.50	54.50	1.0000	16.18	22.83	0.0451	G
12	823927	chr12:694188	694188	C	T	0.00039	0.99961	0.00039	60.00	75.00	1.0000	12.00	12.00	0.0000	C
12	823938	chr12:694199	694199	A	G	0.00025	0.99975	0.00025	60.00	198.00	1.0000	33.00	33.00	0.0015	A
12	823959	chr12:694220	694220	G	C	0.00025	0.99975	0.00025	60.00	228.00	1.0000	33.00	33.00	0.0010	G
12	823979	chr12:694240	694240	G	C	0.00112	0.99888	0.00112	60.00	228.00	1.0000	21.31	21.31	0.0003	G
12	824002	chr12:694263	694263	C	T	0.01264	0.98736	0.01264	60.00	225.91	0.1926	26.05	61.66	0.0002	C
12	824012	chr12:694273	694273	C	T	0.00050	0.99950	0.00050	60.00	228.00	1.0000	34.70	61.30	0.0010	C
12	824025	chr12:694286	694286	C	T	0.00239	0.99761	0.00239	60.00	228.00	1.0000	21.68	70.01	0.0003	C
12	824069	chr12:694330	694330	T	C	0.00044	0.99956	0.00044	58.00	211.00	1.0000	34.00	34.00	0.0000	T
12	824074	chr12:694335	694335	G	T	0.00044	0.99956	0.00044	58.00	161.00	1.0000	30.00	30.00	0.0009	G
12	824101	chr12:694362	694362	C	T	0.00039	0.99961	0.00039	57.00	228.00	1.0000	22.00	22.00	0.0000	C
12	824106	chr12:694367	694367	A	G	0.00025	0.99975	0.00025	58.00	213.00	1.0000	13.00	13.00	0.0035	A
12	824110	chr12:694371	694371	T	C	0.00088	0.99912	0.00088	58.50	228.00	1.0000	25.28	35.73	0.0000	T
12	824118	chr12:694379	694379	C	T	0.00025	0.99975	0.00025	60.00	228.00	1.0000	21.00	21.00	0.0005	C
12	824212	chr12:694473	694473	C	A	0.00039	0.99961	0.00039	60.00	228.00	1.0000	73.00	73.00	0.0000	C
12	824276	chr12:694537	694537	C	T	0.00025	0.99975	0.00025	60.00	171.00	1.0000	21.00	21.00	0.0000	C
12	824886	chr12:695147	695147	G	A	0.00025	0.99975	0.00025	59.00	200.00	1.0000	31.00	31.00	0.0055	G
12	824977	chr12:695238	695238	C	T	0.00044	0.99956	0.00044	59.00	228.00	1.0000	42.00	42.00	0.0000	C
12	825019	chr12:695280	695280	T	C	0.00025	0.99975	0.00025	60.00	228.00	1.0000	73.00	73.00	0.0000	T
12	825091	chr12:695352	695352	G	A	0.00056	0.99944	0.00056	60.00	228.00	1.0000	55.18	58.28	0.0002	G
12	825100	chr12:695361	695361	T	A	0.00044	0.99956	0.00044	60.00	228.00	1.0000	57.00	57.00	0.0000	T
12	825150	chr12:695411	695411	A	G	0.00025	0.99975	0.00025	60.00	212.00	1.0000	35.00	35.00	0.0040	A
12	825502	chr12:695763	695763	T	C	0.00025	0.99975	0.00025	60.00	81.00	1.0000	15.00	15.00	0.0005	T
12	825512	chr12:695773	695773	A	G	0.00044	0.99956	0.00044	60.00	228.00	1.0000	50.00	50.00	0.0000	A
12	825591	chr12:695852	695852	T	C	0.00068	0.99932	0.00068	60.00	228.00	1.0000	53.94	63.38	0.0000	T
12	825622	chr12:695883	695883	G	A	0.00044	0.99956	0.00044	60.00	228.00	1.0000	40.00	40.00	0.0000	G
12	825760	chr12:696021	696021	T	G	0.00034	0.99966	0.00034	60.00	228.00	1.0000	63.64	63.64	0.0002	T
12	825782	chr12:696043	696043	T	C	0.28858	0.71142	0.28858	60.00	221.51	0.0611	29.82	79.88	0.0041	C
12	825867	chr12:696128	696128	G	A	0.00044	0.99956	0.00044	60.00	228.00	1.0000	61.00	61.00	0.0000	G
12	825981	chr12:696242	696242	G	A	0.00044	0.99956	0.00044	60.00	228.00	1.0000	39.00	39.00	0.0000	G
12	826001	chr12:696262	696262	A	G	0.00025	0.99975	0.00025	60.00	228.00	1.0000	25.00	25.00	0.0000	A

12	826049	chr12:696310	696310	T	C	0.00436	0.99564	0.00436	60.00	214.73	1.0000	20.18	38.57	0.0160	T
12	827169	chr12:697430	697430	T	G	0.00081	0.99919	0.00081	60.00	87.00	0.0010	21.00	21.00	0.0517	T
12	827185	chr12:697446	697446	G	C	0.00039	0.99961	0.00039	60.00	164.00	1.0000	17.00	17.00	0.0000	G
12	827194	chr12:697455	697455	C	T	0.00044	0.99956	0.00044	60.00	228.00	1.0000	34.00	34.00	0.0044	C
12	827240	chr12:697501	697501	A	G	0.00025	0.99975	0.00025	60.00	228.00	1.0000	73.00	73.00	0.0000	C
12	827248	chr12:697509	697509	A	G	0.00039	0.99961	0.00039	60.00	228.00	1.0000	64.00	64.00	0.0000	A
12	827258	chr12:697519	697519	G	A	0.00025	0.99975	0.00025	60.00	228.00	1.0000	77.00	77.00	0.0000	G
12	827375	chr12:697636	697636	G	A	0.00032	0.99968	0.00032	60.00	228.00	1.0000	47.67	47.67	0.0000	G
12	827401	chr12:697662	697662	A	G	0.00062	0.99938	0.00062	60.00	228.00	1.0000	49.13	51.67	0.0000	A
12	827440	chr12:697701	697701	G	A	0.00044	0.99956	0.00044	60.00	228.00	1.0000	69.00	69.00	0.0000	G
12	827562	chr12:697823	697823	T	G	0.00025	0.99975	0.00025	60.00	228.00	1.0000	80.00	80.00	0.0000	T
12	827608	chr12:697869	697869	G	A	0.00147	0.99853	0.00147	60.00	228.00	1.0000	53.19	76.59	0.0000	G
12	827614	chr12:697875	697875	C	T	0.00044	0.99956	0.00044	60.00	228.00	1.0000	76.00	76.00	0.0000	C
12	827626	chr12:697887	697887	C	T	0.00044	0.99956	0.00044	60.00	228.00	1.0000	59.00	59.00	0.0000	C
12	827633	chr12:697894	697894	A	T	0.00025	0.99975	0.00025	60.00	228.00	1.0000	51.00	51.00	0.0000	A
12	827643	chr12:697904	697904	G	A	0.00044	0.99956	0.00044	60.00	228.00	1.0000	70.00	70.00	0.0000	G
12	827719	chr12:697980	697980	C	G	0.00045	0.99955	0.00045	59.00	169.00	1.0000	26.00	26.00	0.0097	C
12	828078	chr12:698339	698339	T	C	0.03228	0.96772	0.03228	60.00	228.58	0.0007	50.96	92.90	0.0002	T
12	828114	chr12:698375	698375	C	T	0.00025	0.99975	0.00025	60.00	228.00	1.0000	92.00	92.00	0.0000	C
12	828170	chr12:698431	698431	C	G	0.00039	0.99961	0.00039	60.00	228.00	1.0000	84.00	84.00	0.0000	C
12	828228	chr12:698489	698489	T	C	0.99944	0.00056	0.00056	60.00	227.57	1.0000	43.76	90.04	0.0002	T
12	828237	chr12:698498	698498	G	A	0.06321	0.93679	0.06321	60.00	227.95	0.3484	45.81	91.16	0.0002	A
12	828259	chr12:698520	698520	T	A	0.00088	0.99912	0.00088	60.00	228.00	1.0000	33.25	42.75	0.0000	T
12	828303	chr12:698564	698564	C	G	0.00025	0.99975	0.00025	60.00	228.00	1.0000	41.00	41.00	0.0030	C
12	828315	chr12:698576	698576	C	T	0.00102	0.99898	0.00102	60.00	228.00	1.0000	35.32	57.53	0.0016	C
12	828360	chr12:698621	698621	A	G	0.00025	0.99975	0.00025	60.00	228.00	1.0000	33.00	33.00	0.0060	A
12	828383	chr12:698644	698644	C	A	0.00044	0.99956	0.00044	60.00	228.00	1.0000	36.00	36.00	0.0018	C
12	828384	chr12:698645	698645	G	A	0.00039	0.99961	0.00039	60.00	228.00	1.0000	25.00	25.00	0.0000	G
12	828400	chr12:698661	698661	G	A	0.00260	0.99740	0.00260	59.17	206.73	1.0000	17.03	35.72	0.0027	G
12	828494	chr12:698755	698755	T	C	0.00090	0.99910	0.00090	60.00	69.50	1.0000	11.10	14.90	0.0177	T
12	828519	chr12:698780	698780	A	G	0.00080	0.99920	0.00080	59.78	64.39	1.0000	10.03	12.68	0.0179	A
12	828531	chr12:698792	698792	A	G	0.00080	0.99920	0.00080	59.43	207.29	1.0000	16.95	27.58	0.0038	A
12	828631	chr12:698892	698892	A	G	0.00027	0.99973	0.00027	60.00	69.00	1.0000	10.00	10.00	0.0624	A
12	828682	chr12:698943	698943	C	G	0.00025	0.99975	0.00025	59.00	32.00	1.0000	10.00	10.00	0.0155	C

12	828710	chr12:698971	698971	G	C	0.00044	0.99956	0.00044	60.00	228.00	1.0000	21.00	21.00	0.0000	G
12	828737	chr12:698998	698998	A	G	0.00032	0.99968	0.00032	60.00	228.00	1.0000	55.28	55.28	0.0000	A
12	828749	chr12:699010	699010	G	T	0.00039	0.99961	0.00039	60.00	228.00	1.0000	51.00	51.00	0.0000	G
12	828761	chr12:699022	699022	G	C	0.00077	0.99923	0.00077	60.00	219.50	1.0000	56.15	61.85	0.0000	G
12	828778	chr12:699039	699039	G	C	0.00044	0.99956	0.00044	60.00	228.00	1.0000	69.00	69.00	0.0000	G
12	828791	chr12:699052	699052	C	T	0.00025	0.99975	0.00025	60.00	228.00	1.0000	70.00	70.00	0.0000	C
12	828807	chr12:699068	699068	A	G	0.00044	0.99956	0.00044	60.00	65.00	1.0000	25.00	25.00	0.0018	A
12	828813	chr12:699074	699074	C	T	0.00039	0.99961	0.00039	60.00	228.00	1.0000	62.00	62.00	0.0000	C
12	828817	chr12:699078	699078	G	A	0.00044	0.99956	0.00044	60.00	228.00	1.0000	34.00	34.00	0.0000	G
12	828857	chr12:699118	699118	G	C	0.00064	0.99936	0.00064	59.68	228.00	1.0000	35.73	43.33	0.0003	G
12	829256	chr12:699517	699517	G	A	0.00025	0.99975	0.00025	60.00	228.00	1.0000	36.00	36.00	0.0030	g
12	829297	chr12:699558	699558	A	G	0.00039	0.99961	0.00039	60.00	228.00	1.0000	60.00	60.00	0.0000	A
12	829306	chr12:699567	699567	C	T	0.00025	0.99975	0.00025	60.00	228.00	1.0000	31.00	31.00	0.0000	C
12	829334	chr12:699595	699595	T	C	0.45557	0.54443	0.45557	60.00	227.16	0.1493	43.69	90.03	0.0018	C
12	829351	chr12:699612	699612	T	C	0.00044	0.99956	0.00044	60.00	228.00	1.0000	52.00	52.00	0.0000	T
12	829369	chr12:699630	699630	T	C	0.00025	0.99975	0.00025	60.00	228.00	1.0000	76.00	76.00	0.0000	T
12	829377	chr12:699638	699638	T	C	0.00025	0.99975	0.00025	60.00	228.00	1.0000	90.00	90.00	0.0000	T
12	829384	chr12:699645	699645	T	C	0.00044	0.99956	0.00044	60.00	228.00	1.0000	66.00	66.00	0.0000	T
12	829390	chr12:699651	699651	G	A	0.00025	0.99975	0.00025	60.00	228.00	1.0000	92.00	92.00	0.0000	G
12	829476	chr12:699737	699737	T	C	0.00025	0.99975	0.00025	60.00	228.00	1.0000	73.00	73.00	0.0000	T
12	829579	chr12:699840	699840	T	A	0.14707	0.85293	0.14707	60.00	223.32	0.1709	34.50	72.44	0.0018	T
12	830284	chr12:700545	700545	G	A	0.00027	0.99973	0.00027	57.00	107.00	1.0000	11.00	11.00	0.0864	G
12	830332	chr12:700593	700593	T	A	0.00025	0.99975	0.00025	60.00	228.00	1.0000	45.00	45.00	0.0020	T
12	830349	chr12:700610	700610	G	A	0.60311	0.39689	0.39689	60.00	207.92	0.1577	28.66	75.71	0.0063	A
12	830402	chr12:700663	700663	G	A	0.00039	0.99961	0.00039	60.00	228.00	1.0000	82.00	82.00	0.0000	G
12	830495	chr12:700756	700756	G	A	0.00039	0.99961	0.00039	60.00	228.00	1.0000	66.00	66.00	0.0000	G
12	830501	chr12:700762	700762	G	A	0.00044	0.99956	0.00044	60.00	228.00	1.0000	54.00	54.00	0.0000	G
12	830575	chr12:700836	700836	A	G	0.60202	0.39798	0.39798	60.00	214.23	0.2950	28.84	84.72	0.0079	G
12	830584	chr12:700845	700845	T	C	0.60185	0.39815	0.39815	60.00	214.26	0.1603	29.10	84.87	0.0094	C
12	830600	chr12:700861	700861	C	T	0.00044	0.99956	0.00044	60.00	228.00	1.0000	59.00	59.00	0.0000	C
12	830601	chr12:700862	700862	G	A	0.00124	0.99876	0.00124	60.00	228.00	1.0000	49.50	71.99	0.0016	G
12	830617	chr12:700878	700878	G	A	0.00044	0.99956	0.00044	60.00	228.00	1.0000	77.00	77.00	0.0000	G
12	830667	chr12:700928	700928	G	A	0.00090	0.99910	0.00090	60.00	228.00	1.0000	44.28	70.26	0.0005	G
12	830670	chr12:700931	700931	C	T	0.00158	0.99842	0.00158	60.00	228.00	1.0000	40.04	71.65	0.0005	c

12	830722	chr12:700983	700983	T	C	0.00039	0.99961	0.00039	59.00	228.00	1.0000	27.00	27.00	0.0000	T
12	831590	chr12:701851	701851	G	A	0.02384	0.97616	0.02384	60.00	227.47	0.3976	35.81	79.91	0.0011	G
12	831700	chr12:701961	701961	A	G	0.00025	0.99975	0.00025	60.00	53.00	1.0000	20.00	20.00	0.0075	A
12	831735	chr12:701996	701996	A	G	0.00025	0.99975	0.00025	60.00	104.00	1.0000	19.00	19.00	0.0020	A
12	831749	chr12:702010	702010	C	G	0.00032	0.99968	0.00032	60.00	66.30	1.0000	39.11	39.11	0.0026	C
12	831771	chr12:702032	702032	C	T	0.00025	0.99975	0.00025	60.00	228.00	1.0000	61.00	61.00	0.0020	C
12	831784	chr12:702045	702045	C	A	0.00025	0.99975	0.00025	60.00	228.00	1.0000	19.00	19.00	0.0030	C
12	831788	chr12:702049	702049	A	G	0.00044	0.99956	0.00044	60.00	228.00	1.0000	35.00	35.00	0.0000	A
12	831815	chr12:702076	702076	T	G	0.00039	0.99961	0.00039	60.00	85.00	1.0000	13.00	13.00	0.0154	T
12	831818	chr12:702079	702079	G	T	0.00028	0.99972	0.00028	60.00	119.00	1.0000	10.00	10.00	0.0944	G
12	831820	chr12:702081	702081	A	G	0.00291	0.99709	0.00291	60.00	97.04	1.0000	10.09	19.13	0.0647	A
12	831830	chr12:702091	702091	A	G	0.00025	0.99975	0.00025	60.00	228.00	1.0000	20.00	20.00	0.0200	a
12	831883	chr12:702144	702144	C	T	0.14822	0.85178	0.14822	60.00	220.85	0.3434	20.99	64.01	0.0027	C
12	831903	chr12:702164	702164	A	T	0.00025	0.99975	0.00025	60.00	228.00	1.0000	43.00	43.00	0.0025	A
12	831969	chr12:702230	702230	G	A	0.00045	0.99955	0.00045	60.00	228.00	1.0000	47.58	47.96	0.0006	G
12	832046	chr12:702307	702307	T	C	0.00025	0.99975	0.00025	60.00	89.00	1.0000	17.00	17.00	0.0015	T
12	832069	chr12:702330	702330	T	C	0.00026	0.99974	0.00026	60.00	52.00	1.0000	14.00	14.00	0.0295	T
12	832097	chr12:702358	702358	T	C	0.00039	0.99961	0.00039	60.00	201.00	1.0000	15.00	15.00	0.0108	T
12	832225	chr12:702486	702486	T	C	0.44909	0.55091	0.44909	60.00	203.92	0.0821	21.44	63.75	0.0115	T
12	832228	chr12:702489	702489	G	A	0.00032	0.99968	0.00032	60.00	228.00	1.0000	57.48	57.48	0.0013	G
12	832604	chr12:702865	702865	A	G	0.00025	0.99975	0.00025	60.00	228.00	1.0000	43.00	43.00	0.0025	A
12	832636	chr12:702897	702897	C	A	0.00068	0.99932	0.00068	60.00	228.00	1.0000	34.66	55.19	0.0064	C
12	832739	chr12:703000	703000	T	C	0.00039	0.99961	0.00039	60.00	228.00	1.0000	32.00	32.00	0.0000	T
12	832925	chr12:703186	703186	T	C	0.74508	0.25492	0.25492	60.00	189.14	0.2234	26.08	68.66	0.0027	C
12	832928	chr12:703189	703189	G	C	0.00039	0.99961	0.00039	60.00	228.00	1.0000	41.00	41.00	0.0000	G
12	832944	chr12:703205	703205	G	A	0.00039	0.99961	0.00039	60.00	228.00	1.0000	32.00	32.00	0.0000	G
12	833128	chr12:703389	703389	T	C	0.00050	0.99950	0.00050	60.00	228.00	1.0000	33.30	44.70	0.0005	T
12	833186	chr12:703447	703447	A	G	0.00039	0.99961	0.00039	60.00	228.00	1.0000	39.00	39.00	0.0039	A
12	833220	chr12:703481	703481	C	T	0.00147	0.99853	0.00147	60.00	227.47	1.0000	30.75	52.67	0.0005	C
12	833245	chr12:703506	703506	C	T	0.00044	0.99956	0.00044	60.00	228.00	1.0000	53.00	53.00	0.0000	C
12	833256	chr12:703517	703517	T	C	0.43001	0.56999	0.43001	60.00	218.22	0.0658	32.81	79.05	0.0020	T
12	833418	chr12:703679	703679	T	C	0.45578	0.54422	0.45578	60.00	216.45	0.2417	32.75	76.27	0.0023	T
12	833427	chr12:703688	703688	T	C	0.00025	0.99975	0.00025	60.00	228.00	1.0000	60.00	60.00	0.0005	T
12	833437	chr12:703698	703698	G	A	0.00112	0.99888	0.00112	60.00	228.00	1.0000	59.84	70.03	0.0006	G

12	833461	chr12:703722	703722	T	A	0.00025	0.99975	0.00025	60.00	228.00	1.0000	54.00	54.00	0.0005	T
12	833476	chr12:703737	703737	T	C	0.00025	0.99975	0.00025	60.00	51.00	1.0000	13.00	13.00	0.0005	T
12	833479	chr12:703740	703740	G	A	0.00857	0.99143	0.00857	60.00	80.66	1.0000	10.29	22.10	0.0874	G
12	833536	chr12:703797	703797	A	G	0.00039	0.99961	0.00039	60.00	166.00	1.0000	54.00	54.00	0.0000	A
12	833541	chr12:703802	703802	T	C	0.00044	0.99956	0.00044	60.00	228.00	1.0000	37.00	37.00	0.0000	T
12	833563	chr12:703824	703824	A	G	0.00088	0.99912	0.00088	60.00	228.00	1.0000	52.25	61.75	0.0000	A
12	833569	chr12:703830	703830	T	C	0.00039	0.99961	0.00039	60.00	228.00	1.0000	48.00	48.00	0.0000	T
12	833609	chr12:703870	703870	A	G	0.00044	0.99956	0.00044	60.00	228.00	1.0000	63.00	63.00	0.0000	A
12	833614	chr12:703875	703875	C	A	0.00025	0.99975	0.00025	60.00	228.00	1.0000	57.00	57.00	0.0000	C
12	833641	chr12:703902	703902	T	C	0.00044	0.99956	0.00044	60.00	228.00	1.0000	50.00	50.00	0.0000	T
12	833670	chr12:703931	703931	T	C	0.00041	0.99959	0.00041	60.00	228.00	1.0000	69.61	69.61	0.0000	T
12	833675	chr12:703936	703936	G	A	0.00025	0.99975	0.00025	60.00	228.00	1.0000	88.00	88.00	0.0000	G
12	833788	chr12:704049	704049	C	T	0.00025	0.99975	0.00025	60.00	228.00	1.0000	85.00	85.00	0.0000	C
12	833870	chr12:704131	704131	C	T	0.00025	0.99975	0.00025	60.00	228.00	1.0000	52.00	52.00	0.0000	C
12	833930	chr12:704191	704191	C	T	0.45748	0.54252	0.45748	60.00	218.35	0.2414	31.91	78.87	0.0023	C
12	833950	chr12:704211	704211	T	A	0.00039	0.99961	0.00039	60.00	228.00	1.0000	57.00	57.00	0.0000	T
12	833972	chr12:704233	704233	A	G	0.00044	0.99956	0.00044	60.00	228.00	1.0000	32.00	32.00	0.0000	A
12	833995	chr12:704256	704256	C	A	0.00039	0.99961	0.00039	60.00	220.00	1.0000	25.00	25.00	0.0000	C
12	834017	chr12:704278	704278	G	C	0.00039	0.99961	0.00039	60.00	228.00	1.0000	41.00	41.00	0.0000	G
12	834021	chr12:704282	704282	T	G	0.00033	0.99967	0.00033	60.00	89.60	1.0000	20.00	20.00	0.0731	T
12	834062	chr12:704323	704323	G	C	0.00039	0.99961	0.00039	60.00	32.00	1.0000	24.00	24.00	0.0000	G
12	834097	chr12:704358	704358	A	G	0.00025	0.99975	0.00025	60.00	228.00	1.0000	52.00	52.00	0.0000	-
12	834104	chr12:704365	704365	G	C	0.00044	0.99956	0.00044	60.00	228.00	1.0000	67.00	67.00	0.0000	-
12	834126	chr12:704387	704387	G	C	0.29998	0.70002	0.29998	60.00	225.36	0.3218	40.86	88.27	0.0032	G
12	834140	chr12:704401	704401	A	G	0.00044	0.99956	0.00044	60.00	228.00	1.0000	65.00	65.00	0.0000	A
12	834153	chr12:704414	704414	A	G	0.00044	0.99956	0.00044	60.00	228.00	1.0000	76.00	76.00	0.0000	A
12	834182	chr12:704443	704443	A	G	0.00025	0.99975	0.00025	60.00	228.00	1.0000	43.00	43.00	0.0000	A
12	834184	chr12:704445	704445	T	C	0.00039	0.99961	0.00039	60.00	228.00	1.0000	59.00	59.00	0.0008	T
12	834208	chr12:704469	704469	C	T	0.14026	0.85974	0.14026	60.00	225.26	0.1102	28.57	81.12	0.0000	C
12	834284	chr12:704545	704545	A	G	0.00039	0.99961	0.00039	60.00	228.00	1.0000	31.00	31.00	0.0000	A
12	834313	chr12:704574	704574	G	A	0.00025	0.99975	0.00025	60.00	228.00	1.0000	72.00	72.00	0.0000	G
12	834424	chr12:704685	704685	G	A	0.00062	0.99938	0.00062	60.00	223.34	1.0000	45.61	50.68	0.0000	G
12	834462	chr12:704723	704723	G	A	0.00064	0.99936	0.00064	60.00	55.97	1.0000	17.85	20.25	0.0010	G
12	834611	chr12:704872	704872	T	C	0.00025	0.99975	0.00025	60.00	228.00	1.0000	76.00	76.00	0.0000	T

12	834623	chr12:704884	704884	A	G	0.00147	0.99853	0.00147	60.00	228.00	1.0000	57.27	80.18	0.0000	A
12	834790	chr12:705051	705051	T	G	0.00039	0.99961	0.00039	60.00	228.00	1.0000	64.00	64.00	0.0000	T
12	834807	chr12:705068	705068	A	T	0.00025	0.99975	0.00025	60.00	50.00	1.0000	31.00	31.00	0.0005	A
12	834879	chr12:705140	705140	C	G	0.00135	0.99865	0.00135	60.00	228.00	1.0000	33.35	63.16	0.0005	C
12	834881	chr12:705142	705142	A	G	0.00044	0.99956	0.00044	60.00	228.00	1.0000	48.00	48.00	0.0009	A
12	834906	chr12:705167	705167	A	G	0.00136	0.99864	0.00136	60.00	209.93	1.0000	16.68	31.42	0.0011	A
12	834939	chr12:705200	705200	T	C	0.00044	0.99956	0.00044	60.00	228.00	1.0000	38.00	38.00	0.0018	T
12	834955	chr12:705216	705216	C	T	0.00048	0.99952	0.00048	60.00	228.00	1.0000	47.20	56.80	0.0013	C
12	834964	chr12:705225	705225	C	T	0.00025	0.99975	0.00025	60.00	228.00	1.0000	50.00	50.00	0.0025	C
12	834993	chr12:705254	705254	C	T	0.00044	0.99956	0.00044	60.00	228.00	1.0000	25.00	25.00	0.0018	C
12	835013	chr12:705274	705274	C	T	0.00025	0.99975	0.00025	60.00	173.00	1.0000	30.00	30.00	0.0015	C
12	835020	chr12:705281	705281	T	G	0.00025	0.99975	0.00025	60.00	228.00	1.0000	36.00	36.00	0.0015	T
12	835046	chr12:705307	705307	G	A	0.00025	0.99975	0.00025	60.00	228.00	1.0000	44.00	44.00	0.0010	A
12	835074	chr12:705335	705335	G	A	0.13893	0.86107	0.13893	60.00	224.38	0.0429	26.58	74.09	0.0034	G
12	835140	chr12:705401	705401	T	C	0.00025	0.99975	0.00025	60.00	228.00	1.0000	15.00	15.00	0.0015	T
12	835173	chr12:705434	705434	C	A	0.00025	0.99975	0.00025	60.00	228.00	1.0000	40.00	40.00	0.0015	C
12	835220	chr12:705481	705481	C	T	0.00025	0.99975	0.00025	60.00	228.00	1.0000	56.00	56.00	0.0050	C
12	835221	chr12:705482	705482	G	A	0.00025	0.99975	0.00025	60.00	228.00	1.0000	41.00	41.00	0.0040	G
12	835236	chr12:705497	705497	A	C	0.00025	0.99975	0.00025	60.00	228.00	1.0000	46.00	46.00	0.0015	A
12	835247	chr12:705508	705508	G	C	0.00044	0.99956	0.00044	60.00	228.00	1.0000	50.00	50.00	0.0018	G
12	835250	chr12:705511	705511	C	T	0.00025	0.99975	0.00025	60.00	228.00	1.0000	38.00	38.00	0.0020	C
12	835256	chr12:705517	705517	A	T	0.00158	0.99842	0.00158	60.00	228.00	1.0000	40.29	61.54	0.0011	A
12	835317	chr12:705578	705578	C	T	0.60484	0.39516	0.39516	60.00	201.86	0.0631	24.51	70.22	0.0065	C
12	835318	chr12:705579	705579	G	A	0.00339	0.99661	0.00339	60.00	228.00	1.0000	35.12	58.04	0.0007	G
12	835354	chr12:705615	705615	G	A	0.03729	0.96271	0.03729	60.00	224.34	0.1522	26.38	64.82	0.0004	G
12	835359	chr12:705620	705620	A	T	0.00088	0.99912	0.00088	60.00	228.00	1.0000	51.20	58.80	0.0009	A
12	835514	chr12:705775	705775	G	A	0.00025	0.99975	0.00025	60.00	228.00	1.0000	37.00	37.00	0.0010	G
12	835589	chr12:705850	705850	G	A	0.00025	0.99975	0.00025	60.00	228.00	1.0000	58.00	58.00	0.0005	G
12	835659	chr12:705920	705920	C	G	0.00124	0.99876	0.00124	60.00	228.00	1.0000	31.61	57.58	0.0005	C
12	835661	chr12:705922	705922	C	T	0.00039	0.99961	0.00039	60.00	228.00	1.0000	60.00	60.00	0.0000	C
12	835662	chr12:705923	705923	G	A	0.00050	0.99950	0.00050	60.00	228.00	1.0000	31.28	41.73	0.0010	G
12	835671	chr12:705932	705932	A	C	0.00025	0.99975	0.00025	60.00	228.00	1.0000	33.00	33.00	0.0010	A
12	835745	chr12:706006	706006	G	T	0.00025	0.99975	0.00025	60.00	228.00	1.0000	36.00	36.00	0.0010	G
12	835762	chr12:706023	706023	C	T	0.00025	0.99975	0.00025	60.00	228.00	1.0000	52.00	52.00	0.0015	C

12	835776	chr12:706037	706037	C	T	0.00025	0.99975	0.00025	60.00	228.00	1.0000	46.00	46.00	0.0015	C
12	835815	chr12:706076	706076	C	T	0.00062	0.99938	0.00062	60.00	228.00	1.0000	56.17	65.30	0.0004	C
12	835835	chr12:706096	706096	C	A	0.00039	0.99961	0.00039	60.00	228.00	1.0000	50.00	50.00	0.0000	C
12	835836	chr12:706097	706097	C	A	0.00039	0.99961	0.00039	60.00	228.00	1.0000	72.00	72.00	0.0000	C
12	835881	chr12:706142	706142	G	A	0.00025	0.99975	0.00025	60.00	228.00	1.0000	40.00	40.00	0.0005	G
12	835883	chr12:706144	706144	G	A	0.00044	0.99956	0.00044	60.00	228.00	1.0000	76.00	76.00	0.0000	G
12	835909	chr12:706170	706170	A	T	0.01129	0.98871	0.01129	60.00	227.65	1.0000	40.05	79.44	0.0002	A
12	835950	chr12:706211	706211	T	C	0.00025	0.99975	0.00025	60.00	228.00	1.0000	77.00	77.00	0.0005	T
12	835974	chr12:706235	706235	G	A	0.00061	0.99939	0.00061	60.00	228.00	1.0000	68.00	75.47	0.0000	G
12	835975	chr12:706236	706236	A	T	0.00077	0.99923	0.00077	60.00	228.00	1.0000	81.10	84.90	0.0000	A
12	835987	chr12:706248	706248	T	G	0.00293	0.99707	0.00293	60.00	227.22	1.0000	47.81	86.47	0.0000	G
12	836003	chr12:706264	706264	T	C	0.00039	0.99961	0.00039	60.00	228.00	1.0000	63.00	63.00	0.0000	T
12	836100	chr12:706361	706361	C	T	0.00025	0.99975	0.00025	60.00	228.00	1.0000	56.00	56.00	0.0010	C
12	836149	chr12:706410	706410	A	G	0.00025	0.99975	0.00025	60.00	228.00	1.0000	59.00	59.00	0.0005	A
12	836211	chr12:706472	706472	T	C	0.00039	0.99961	0.00039	60.00	228.00	1.0000	19.00	19.00	0.0000	T
12	836237	chr12:706498	706498	A	G	0.00039	0.99961	0.00039	60.00	228.00	1.0000	67.00	67.00	0.0000	a
12	836272	chr12:706533	706533	G	A	0.00079	0.99921	0.00079	60.00	224.42	1.0000	36.33	54.26	0.0002	g
12	836302	chr12:706563	706563	C	T	0.00056	0.99944	0.00056	60.00	228.00	1.0000	47.50	49.70	0.0005	C
12	836332	chr12:706593	706593	G	A	0.00039	0.99961	0.00039	60.00	228.00	1.0000	52.00	52.00	0.0000	G
12	836352	chr12:706613	706613	C	A	0.00025	0.99975	0.00025	60.00	228.00	1.0000	62.00	62.00	0.0010	C
12	836354	chr12:706615	706615	T	A	0.00025	0.99975	0.00025	60.00	228.00	1.0000	62.00	62.00	0.0010	T
12	836480	chr12:706741	706741	C	T	0.00044	0.99956	0.00044	60.00	228.00	1.0000	75.00	75.00	0.0000	C
12	836486	chr12:706747	706747	G	A	0.00025	0.99975	0.00025	60.00	228.00	1.0000	55.00	55.00	0.0005	G
12	836598	chr12:706859	706859	C	T	0.00025	0.99975	0.00025	60.00	228.00	1.0000	43.00	43.00	0.0005	C
12	836601	chr12:706862	706862	T	C	0.00327	0.99673	0.00327	60.00	226.19	1.0000	22.33	60.00	0.0002	C
12	836652	chr12:706913	706913	G	A	0.00025	0.99975	0.00025	60.00	228.00	1.0000	57.00	57.00	0.0005	G
12	836656	chr12:706917	706917	C	A	0.00077	0.99923	0.00077	60.00	228.00	1.0000	38.23	46.78	0.0000	C
12	836731	chr12:706992	706992	A	T	0.00025	0.99975	0.00025	60.00	228.00	1.0000	62.00	62.00	0.0000	A
12	836748	chr12:707009	707009	G	C	0.00039	0.99961	0.00039	60.00	228.00	1.0000	43.00	43.00	0.0000	G
12	836754	chr12:707015	707015	T	C	0.00088	0.99912	0.00088	60.00	96.00	1.0000	20.00	20.00	0.0000	T
12	836771	chr12:707032	707032	T	C	0.00050	0.99950	0.00050	59.00	74.00	1.0000	11.00	11.00	0.1210	T
12	836833	chr12:707094	707094	T	C	0.00044	0.99956	0.00044	60.00	228.00	1.0000	15.00	15.00	0.1235	T
12	836839	chr12:707100	707100	G	A	0.00027	0.99973	0.00027	60.00	228.00	1.0000	97.00	97.00	0.0619	G
12	836848	chr12:707109	707109	A	G	0.00046	0.99954	0.00046	60.00	156.00	1.0000	12.00	12.00	0.1651	A

12	836876	chr12:707137	707137	G	A	0.00025	0.99975	0.00025	59.00	125.00	1.0000	20.00	20.00	0.0205	G
12	837035	chr12:707296	707296	A	G	0.00025	0.99975	0.00025	60.00	92.00	1.0000	14.00	14.00	0.0010	A
12	837113	chr12:707374	707374	T	C	0.00044	0.99956	0.00044	60.00	228.00	1.0000	53.00	53.00	0.0000	T
12	837163	chr12:707424	707424	T	A	0.00044	0.99956	0.00044	60.00	215.00	1.0000	26.00	26.00	0.0000	T
12	837287	chr12:707548	707548	T	A	0.00025	0.99975	0.00025	60.00	228.00	1.0000	71.00	71.00	0.0000	T
12	837294	chr12:707555	707555	G	A	0.00077	0.99923	0.00077	60.00	228.00	1.0000	62.30	73.70	0.0000	G
12	837357	chr12:707618	707618	C	T	0.00025	0.99975	0.00025	60.00	228.00	1.0000	46.00	46.00	0.0005	C
12	837384	chr12:707645	707645	A	T	0.00025	0.99975	0.00025	60.00	228.00	1.0000	55.00	55.00	0.0000	A
12	837385	chr12:707646	707646	T	C	0.00030	0.99970	0.00030	60.00	228.00	1.0000	61.04	61.04	0.0000	T
12	837451	chr12:707712	707712	A	C	0.00025	0.99975	0.00025	60.00	228.00	1.0000	32.00	32.00	0.0000	A
12	837475	chr12:707736	707736	G	C	0.00025	0.99975	0.00025	60.00	228.00	1.0000	61.00	61.00	0.0000	G
12	837494	chr12:707755	707755	T	G	0.02223	0.97777	0.02223	60.00	228.00	1.0000	29.53	67.97	0.0000	T
12	837546	chr12:707807	707807	T	C	0.00039	0.99961	0.00039	60.00	228.00	1.0000	27.00	27.00	0.0000	T
12	837608	chr12:707869	707869	G	A	0.00025	0.99975	0.00025	60.00	228.00	1.0000	60.00	60.00	0.0005	G
12	837619	chr12:707880	707880	G	A	0.00025	0.99975	0.00025	60.00	228.00	1.0000	32.00	32.00	0.0005	G
12	837625	chr12:707886	707886	C	G	0.00048	0.99952	0.00048	60.00	202.45	1.0000	26.04	42.43	0.0003	C
12	837638	chr12:707899	707899	G	A	0.00221	0.99779	0.00221	60.00	207.75	1.0000	34.15	44.70	0.0000	G
12	837648	chr12:707909	707909	C	T	0.00025	0.99975	0.00025	60.00	228.00	1.0000	51.00	51.00	0.0000	C
12	837678	chr12:707939	707939	G	A	0.00039	0.99961	0.00039	60.00	228.00	1.0000	68.00	68.00	0.0000	G
12	837715	chr12:707976	707976	G	A	0.00077	0.99923	0.00077	60.00	128.00	1.0000	34.10	37.90	0.0008	G
12	837827	chr12:708088	708088	T	C	0.00044	0.99956	0.00044	60.00	228.00	1.0000	29.00	29.00	0.0000	T
12	837845	chr12:708106	708106	T	C	0.00044	0.99956	0.00044	60.00	228.00	1.0000	28.00	28.00	0.0009	T
12	837859	chr12:708120	708120	T	C	0.00025	0.99975	0.00025	60.00	228.00	1.0000	96.00	96.00	0.0030	T
12	837906	chr12:708167	708167	G	A	0.00026	0.99974	0.00026	60.00	122.00	1.0000	15.00	15.00	0.0389	G
12	837955	chr12:708216	708216	C	T	0.00028	0.99972	0.00028	60.00	168.00	1.0000	68.00	68.00	0.1033	C
12	838038	chr12:708299	708299	C	T	0.00026	0.99974	0.00026	60.00	228.00	1.0000	93.00	93.00	0.0215	C
12	838042	chr12:708303	708303	A	C	0.00244	0.99756	0.00244	60.00	59.39	1.0000	10.52	13.26	0.0739	A
12	838091	chr12:708352	708352	G	A	0.00044	0.99956	0.00044	60.00	228.00	1.0000	34.00	34.00	0.0000	G
12	838113	chr12:708374	708374	A	T	0.00025	0.99975	0.00025	60.00	228.00	1.0000	49.00	49.00	0.0000	A
12	838114	chr12:708375	708375	C	T	0.00039	0.99961	0.00039	60.00	228.00	1.0000	60.00	60.00	0.0000	C
12	838122	chr12:708383	708383	G	A	0.00025	0.99975	0.00025	60.00	228.00	1.0000	59.00	59.00	0.0000	G
12	838140	chr12:708401	708401	C	T	0.00039	0.99961	0.00039	60.00	228.00	1.0000	43.00	43.00	0.0000	C
12	838266	chr12:708527	708527	A	G	0.00025	0.99975	0.00025	60.00	228.00	1.0000	32.00	32.00	0.0000	A
12	838282	chr12:708543	708543	G	A	0.00044	0.99956	0.00044	60.00	228.00	1.0000	53.00	53.00	0.0000	G

12	838363	chr12:708624	708624	A	G	0.00025	0.99975	0.00025	60.00	228.00	1.0000	43.00	43.00	0.0010	A
12	838380	chr12:708641	708641	C	T	0.00057	0.99943	0.00057	60.00	228.00	1.0000	39.08	41.17	0.0016	C
12	838588	chr12:708849	708849	T	A	0.00039	0.99961	0.00039	60.00	228.00	1.0000	54.00	54.00	0.0000	T
12	838675	chr12:708936	708936	G	A	0.00350	0.99650	0.00350	60.00	228.00	1.0000	29.17	58.57	0.0002	G
12	838713	chr12:708974	708974	G	A	0.00079	0.99921	0.00079	60.00	228.00	1.0000	33.74	45.85	0.0002	G
12	838746	chr12:709007	709007	C	G	0.00044	0.99956	0.00044	60.00	228.00	1.0000	39.00	39.00	0.0000	C
12	838840	chr12:709101	709101	A	T	0.00050	0.99950	0.00050	59.00	130.00	1.0000	20.13	24.88	0.0070	A
12	838879	chr12:709140	709140	G	T	0.01145	0.98855	0.01145	60.00	219.58	1.0000	13.12	38.32	0.0043	G
12	838882	chr12:709143	709143	A	T	0.00039	0.99961	0.00039	60.00	228.00	1.0000	23.00	23.00	0.0031	A
12	838888	chr12:709149	709149	A	C	0.00080	0.99920	0.00080	60.00	186.68	1.0000	13.56	20.13	0.0035	A
12	838908	chr12:709169	709169	T	C	0.00094	0.99906	0.00094	60.00	69.00	0.0023	14.00	14.00	0.0574	T
12	838929	chr12:709190	709190	A	C	0.00046	0.99954	0.00046	60.00	105.00	1.0000	10.00	10.00	0.0353	A
12	838931	chr12:709192	709192	T	C	0.00555	0.99445	0.00555	60.00	225.52	1.0000	13.79	30.49	0.0045	T
12	838942	chr12:709203	709203	C	T	0.00039	0.99961	0.00039	60.00	228.00	1.0000	35.00	35.00	0.0000	C
12	838943	chr12:709204	709204	G	A	0.00048	0.99952	0.00048	60.00	226.74	1.0000	30.83	32.89	0.0000	G
12	839011	chr12:709272	709272	G	A	0.02099	0.97901	0.02099	60.00	226.61	1.0000	33.60	79.79	0.0002	A
12	839027	chr12:709288	709288	T	A	0.00044	0.99956	0.00044	60.00	228.00	1.0000	51.00	51.00	0.0000	T
12	839127	chr12:709388	709388	G	A	0.00039	0.99961	0.00039	60.00	228.00	1.0000	29.00	29.00	0.0000	G
12	839129	chr12:709390	709390	G	A	0.00124	0.99876	0.00124	59.90	222.90	1.0000	20.01	27.32	0.0016	G
12	839419	chr12:709680	709680	G	A	0.00044	0.99956	0.00044	60.00	228.00	1.0000	14.00	14.00	0.0027	G
12	839446	chr12:709707	709707	G	A	0.00039	0.99961	0.00039	60.00	228.00	1.0000	52.00	52.00	0.0000	G
12	839472	chr12:709733	709733	G	A	0.00077	0.99923	0.00077	60.00	228.00	1.0000	51.78	81.23	0.0000	G
12	839552	chr12:709813	709813	G	A	0.00025	0.99975	0.00025	60.00	225.00	1.0000	40.00	40.00	0.0000	G
12	839555	chr12:709816	709816	T	C	0.00044	0.99956	0.00044	60.00	70.00	1.0000	10.00	10.00	0.0035	T
12	839579	chr12:709840	709840	G	C	0.00575	0.99425	0.00575	59.93	216.96	1.0000	20.87	41.73	0.0186	G
12	839974	chr12:710235	710235	T	C	0.00025	0.99975	0.00025	60.00	58.00	1.0000	10.00	10.00	0.0110	T
12	839982	chr12:710243	710243	G	T	0.00068	0.99932	0.00068	59.45	222.55	1.0000	22.84	32.80	0.0067	G
12	840098	chr12:710359	710359	C	T	0.00025	0.99975	0.00025	60.00	228.00	1.0000	97.00	97.00	0.0000	C
12	840106	chr12:710367	710367	T	C	0.00045	0.99955	0.00045	60.00	228.00	1.0000	88.58	90.07	0.0000	T
12	840148	chr12:710409	710409	G	A	0.00044	0.99956	0.00044	60.00	228.00	1.0000	72.00	72.00	0.0000	G
12	840200	chr12:710461	710461	T	C	0.00039	0.99961	0.00039	60.00	228.00	1.0000	80.00	80.00	0.0000	T
12	840235	chr12:710496	710496	G	C	0.00044	0.99956	0.00044	60.00	228.00	1.0000	83.00	83.00	0.0000	G
12	840345	chr12:710606	710606	C	T	0.00025	0.99975	0.00025	60.00	228.00	1.0000	82.00	82.00	0.0000	C
12	840436	chr12:710697	710697	G	T	0.00077	0.99923	0.00077	60.00	228.00	1.0000	54.05	55.95	0.0000	A

12	840466	chr12:710727	710727	G	T	0.00030	0.99970	0.00030	60.00	228.00	1.0000	44.00	44.00	0.0006 G
12	840488	chr12:710749	710749	A	T	0.00153	0.99847	0.00153	59.67	199.67	1.0000	11.13	21.50	0.0230 A
12	840494	chr12:710755	710755	G	A	0.00025	0.99975	0.00025	59.00	176.00	1.0000	17.00	17.00	0.0120 G
12	840734	chr12:710995	710995	C	T	0.00030	0.99970	0.00030	60.00	228.00	1.0000	54.18	54.18	0.0015 C
12	840736	chr12:710997	710997	A	G	0.55768	0.44232	0.44232	60.00	164.69	0.1052	12.72	43.93	0.0197 A
12	840852	chr12:711113	711113	C	G	0.00045	0.99955	0.00045	60.00	228.00	1.0000	51.17	54.18	0.0002 C
12	840881	chr12:711142	711142	G	A	0.00158	0.99842	0.00158	60.00	228.00	1.0000	42.74	63.62	0.0002 G
12	840907	chr12:711168	711168	G	A	0.00025	0.99975	0.00025	60.00	228.00	1.0000	86.00	86.00	0.0005 G
12	840908	chr12:711169	711169	C	A	0.00025	0.99975	0.00025	60.00	228.00	1.0000	80.00	80.00	0.0005 C
12	840930	chr12:711191	711191	C	T	0.02089	0.97911	0.02089	60.00	226.55	1.0000	34.07	77.63	0.0005 C
12	840996	chr12:711257	711257	G	C	0.00034	0.99966	0.00034	60.00	228.00	1.0000	60.80	60.80	0.0000 G
12	841006	chr12:711267	711267	C	T	0.02099	0.97901	0.02099	60.00	225.68	1.0000	29.62	76.68	0.0002 c
12	841092	chr12:711353	711353	G	A	0.00025	0.99975	0.00025	60.00	114.00	1.0000	13.00	13.00	0.0000 G
12	841113	chr12:711374	711374	G	C	0.00845	0.99155	0.00845	60.00	220.31	1.0000	17.50	45.24	0.0004 G
12	841238	chr12:711499	711499	C	T	0.00025	0.99975	0.00025	60.00	228.00	1.0000	100.00	100.00	0.0000 C
12	841267	chr12:711528	711528	A	G	0.00025	0.99975	0.00025	59.00	228.00	1.0000	68.00	68.00	0.0000 A
12	841365	chr12:711626	711626	G	A	0.00025	0.99975	0.00025	60.00	228.00	1.0000	47.00	47.00	0.0000 A
12	841463	chr12:711724	711724	A	G	0.00050	0.99950	0.00050	60.00	228.00	1.0000	58.23	66.78	0.0005 A
12	841484	chr12:711745	711745	A	T	0.00113	0.99887	0.00113	60.00	228.00	1.0000	33.76	47.83	0.0018 A
12	841887	chr12:712148	712148	C	G	0.00237	0.99763	0.00237	60.00	228.00	1.0000	33.05	51.14	0.0005 C
12	841920	chr12:712181	712181	G	T	0.00194	0.99806	0.00194	59.91	228.00	1.0000	24.30	36.47	0.0106 G
12	842127	chr12:712388	712388	C	T	0.00075	0.99925	0.00075	60.00	228.00	1.0000	42.25	49.85	0.0000 c
12	842163	chr12:712424	712424	T	C	0.00039	0.99961	0.00039	60.00	228.00	1.0000	62.00	62.00	0.0039 T
12	842197	chr12:712458	712458	A	G	0.00155	0.99845	0.00155	59.00	228.00	1.0000	30.30	38.93	0.0046 A
12	842204	chr12:712465	712465	C	T	0.00025	0.99975	0.00025	59.00	228.00	1.0000	42.00	42.00	0.0000 C
12	842217	chr12:712478	712478	T	C	0.00044	0.99956	0.00044	60.00	217.00	1.0000	16.00	16.00	0.0009 T
12	842254	chr12:712515	712515	A	C	0.00306	0.99694	0.00306	59.72	219.45	1.0000	27.46	71.28	0.0049 A
12	842279	chr12:712540	712540	C	T	0.00025	0.99975	0.00025	60.00	228.00	1.0000	65.00	65.00	0.0005 C
12	842293	chr12:712554	712554	A	G	0.02027	0.97973	0.02027	59.21	225.30	1.0000	26.56	58.68	0.0036 G
12	842335	chr12:712596	712596	G	A	0.00034	0.99966	0.00034	58.71	212.77	1.0000	28.04	28.04	0.0011 G
12	842337	chr12:712598	712598	G	A	0.00025	0.99975	0.00025	59.00	228.00	1.0000	43.00	43.00	0.0000 G
12	842377	chr12:712638	712638	A	G	0.00039	0.99961	0.00039	60.00	228.00	1.0000	69.00	69.00	0.0000 A
12	842692	chr12:712953	712953	A	G	0.00025	0.99975	0.00025	60.00	114.00	1.0000	17.00	17.00	0.0000 A
12	842705	chr12:712966	712966	A	T	0.00044	0.99956	0.00044	59.00	228.00	1.0000	35.00	35.00	0.0000 A

12	843120	chr12:713381	713381	G	A	0.00067	0.99933	0.00067	59.19	192.33	1.0000	11.30	12.96	0.0434	G
12	843137	chr12:713398	713398	A	T	0.00044	0.99956	0.00044	60.00	67.00	1.0000	10.00	10.00	0.0000	A
12	843202	chr12:713463	713463	C	T	0.06881	0.93119	0.06881	60.00	221.97	0.1110	20.95	55.12	0.0079	C
12	843251	chr12:713512	713512	T	C	0.00025	0.99975	0.00025	60.00	228.00	1.0000	35.00	35.00	0.0005	T
12	843260	chr12:713521	713521	A	G	0.00039	0.99961	0.00039	59.00	228.00	1.0000	23.00	23.00	0.0023	A
12	843273	chr12:713534	713534	G	T	0.00880	0.99120	0.00880	58.44	210.83	1.0000	11.09	23.03	0.0128	G
12	843280	chr12:713541	713541	T	C	0.00025	0.99975	0.00025	57.00	69.00	1.0000	10.00	10.00	0.0025	T
12	843408	chr12:713669	713669	C	T	0.00044	0.99956	0.00044	60.00	228.00	1.0000	33.00	33.00	0.0000	C
12	843411	chr12:713672	713672	G	T	0.00044	0.99956	0.00044	60.00	228.00	1.0000	53.00	53.00	0.0000	G
12	843441	chr12:713702	713702	C	T	0.00025	0.99975	0.00025	60.00	228.00	1.0000	92.00	92.00	0.0000	C
12	843483	chr12:713744	713744	T	C	0.00039	0.99961	0.00039	60.00	228.00	1.0000	83.00	83.00	0.0000	T
12	843486	chr12:713747	713747	G	A	0.00025	0.99975	0.00025	60.00	228.00	1.0000	47.00	47.00	0.0000	G
12	843495	chr12:713756	713756	C	T	0.00025	0.99975	0.00025	60.00	228.00	1.0000	49.00	49.00	0.0000	C
12	843559	chr12:713820	713820	G	C	0.00025	0.99975	0.00025	60.00	228.00	1.0000	48.00	48.00	0.0000	G
12	843579	chr12:713840	713840	C	T	0.00039	0.99961	0.00039	57.00	228.00	1.0000	28.00	28.00	0.0000	C
12	843623	chr12:713884	713884	T	C	0.00025	0.99975	0.00025	60.00	228.00	1.0000	47.00	47.00	0.0005	T
12	843930	chr12:714191	714191	C	T	0.00025	0.99975	0.00025	58.00	214.00	1.0000	12.00	12.00	0.0020	C
12	843934	chr12:714195	714195	C	T	0.01628	0.98372	0.01628	59.31	201.88	1.0000	10.30	25.22	0.0289	C
12	843993	chr12:714254	714254	A	T	0.00044	0.99956	0.00044	60.00	187.00	1.0000	30.00	30.00	0.0000	A
12	844089	chr12:714350	714350	C	T	0.00025	0.99975	0.00025	60.00	228.00	1.0000	63.00	63.00	0.0000	C
12	844109	chr12:714370	714370	G	A	0.00044	0.99956	0.00044	60.00	228.00	1.0000	31.00	31.00	0.0000	G
12	844227	chr12:714488	714488	C	T	0.00113	0.99887	0.00113	60.00	226.64	1.0000	46.00	69.41	0.0002	C
12	844271	chr12:714532	714532	C	T	0.00025	0.99975	0.00025	60.00	228.00	1.0000	63.00	63.00	0.0000	C
12	844395	chr12:714656	714656	C	T	0.00025	0.99975	0.00025	60.00	228.00	1.0000	78.00	78.00	0.0000	C
12	844538	chr12:714799	714799	G	A	0.00113	0.99887	0.00113	60.00	221.71	1.0000	21.25	27.19	0.0023	G
12	844542	chr12:714803	714803	G	A	0.00030	0.99970	0.00030	60.00	228.00	1.0000	21.03	21.03	0.0024	G
12	844918	chr12:715179	715179	A	T	0.00039	0.99961	0.00039	60.00	228.00	1.0000	59.00	59.00	0.0000	A
12	845127	chr12:715388	715388	G	A	0.00039	0.99961	0.00039	60.00	228.00	1.0000	73.00	73.00	0.0000	G
12	845190	chr12:715451	715451	G	A	0.00044	0.99956	0.00044	60.00	228.00	1.0000	34.00	34.00	0.0000	g
12	845214	chr12:715475	715475	T	G	0.00075	0.99925	0.00075	60.00	228.00	1.0000	55.10	63.65	0.0000	T
12	845215	chr12:715476	715476	C	T	0.00039	0.99961	0.00039	60.00	228.00	1.0000	56.00	56.00	0.0000	C
12	845315	chr12:715576	715576	G	A	0.00061	0.99939	0.00061	60.00	228.00	1.0000	38.65	59.98	0.0009	g
12	845333	chr12:715594	715594	C	T	0.00025	0.99975	0.00025	60.00	164.00	1.0000	22.00	22.00	0.0020	C
12	845355	chr12:715616	715616	G	A	0.00025	0.99975	0.00025	60.00	228.00	1.0000	38.00	38.00	0.0050	G

12	845479	chr12:715740	715740	T	C	0.00039	0.99961	0.00039	60.00	228.00	1.0000	87.00	87.00	0.0000	T
12	845489	chr12:715750	715750	G	A	0.00025	0.99975	0.00025	60.00	228.00	1.0000	67.00	67.00	0.0000	G
12	845518	chr12:715779	715779	C	T	0.00044	0.99956	0.00044	60.00	228.00	1.0000	59.00	59.00	0.0000	C
12	845533	chr12:715794	715794	A	G	0.00025	0.99975	0.00025	60.00	228.00	1.0000	38.00	38.00	0.0010	A
12	845562	chr12:715823	715823	T	C	0.00044	0.99956	0.00044	60.00	156.00	1.0000	27.00	27.00	0.0000	T
12	845565	chr12:715826	715826	C	T	0.00025	0.99975	0.00025	60.00	228.00	1.0000	47.00	47.00	0.0005	C
12	845574	chr12:715835	715835	G	C	0.02159	0.97841	0.02159	60.00	225.64	1.0000	30.62	73.64	0.0018	G
12	846146	chr12:716407	716407	G	A	0.01778	0.98222	0.01778	60.00	217.96	1.0000	15.45	30.68	0.0099	G
12	846194	chr12:716455	716455	A	T	0.00039	0.99961	0.00039	60.00	228.00	1.0000	43.00	43.00	0.0000	A
12	846274	chr12:716535	716535	C	T	0.00025	0.99975	0.00025	60.00	228.00	1.0000	98.00	98.00	0.0005	C
12	846280	chr12:716541	716541	A	G	0.00025	0.99975	0.00025	60.00	228.00	1.0000	57.00	57.00	0.0005	A
12	846319	chr12:716580	716580	C	G	0.00025	0.99975	0.00025	60.00	228.00	1.0000	71.00	71.00	0.0010	C
12	846366	chr12:716627	716627	G	A	0.00044	0.99956	0.00044	60.00	173.00	1.0000	13.00	13.00	0.0009	g
12	846389	chr12:716650	716650	C	T	0.00025	0.99975	0.00025	60.00	228.00	1.0000	71.00	71.00	0.0015	C
12	846450	chr12:716711	716711	C	T	0.01163	0.98837	0.01163	60.00	227.84	0.0954	27.18	79.94	0.0005	C
12	846451	chr12:716712	716712	G	T	0.00062	0.99938	0.00062	60.00	228.00	1.0000	56.64	65.50	0.0000	G
12	846576	chr12:716837	716837	C	T	0.00044	0.99956	0.00044	60.00	228.00	1.0000	55.00	55.00	0.0009	C
12	846610	chr12:716871	716871	C	T	0.00039	0.99961	0.00039	60.00	228.00	1.0000	50.00	50.00	0.0000	T
12	846618	chr12:716879	716879	G	C	0.00044	0.99956	0.00044	60.00	228.00	1.0000	60.00	60.00	0.0018	G
12	846662	chr12:716923	716923	T	G	0.00044	0.99956	0.00044	60.00	228.00	1.0000	32.00	32.00	0.0018	T
12	846669	chr12:716930	716930	C	A	0.00136	0.99864	0.00136	60.00	228.00	1.0000	22.41	41.00	0.0036	C
12	846698	chr12:716959	716959	C	A	0.00640	0.99360	0.00640	60.00	222.02	1.0000	16.13	40.09	0.0123	C
12	846725	chr12:716986	716986	G	A	0.00046	0.99954	0.00046	60.00	228.00	1.0000	29.38	39.11	0.0027	G
12	846750	chr12:717011	717011	T	C	0.00032	0.99968	0.00032	60.00	228.00	1.0000	46.44	46.44	0.0029	T
12	846772	chr12:717033	717033	G	C	0.00025	0.99975	0.00025	60.00	228.00	1.0000	34.00	34.00	0.0030	G
12	846793	chr12:717054	717054	G	A	0.00077	0.99923	0.00077	60.00	228.00	1.0000	25.08	27.93	0.0000	G
12	846807	chr12:717068	717068	A	C	0.00052	0.99948	0.00052	60.00	80.50	1.0000	14.13	18.88	0.0459	A
12	846822	chr12:717083	717083	G	A	0.00025	0.99975	0.00025	60.00	210.00	1.0000	10.00	10.00	0.0015	G
12	846921	chr12:717182	717182	C	T	0.00035	0.99965	0.00035	60.00	92.32	1.0000	20.56	20.56	0.0770	C
12	846962	chr12:717223	717223	C	T	0.00044	0.99956	0.00044	60.00	228.00	1.0000	50.00	50.00	0.0018	C
12	847007	chr12:717268	717268	G	A	0.00824	0.99176	0.00824	60.00	227.50	0.0613	30.43	66.45	0.0009	G
12	847018	chr12:717279	717279	G	T	0.00044	0.99956	0.00044	60.00	228.00	1.0000	63.00	63.00	0.0009	G
12	847047	chr12:717308	717308	A	C	0.00025	0.99975	0.00025	60.00	228.00	1.0000	57.00	57.00	0.0010	A
12	847082	chr12:717343	717343	A	T	0.00025	0.99975	0.00025	60.00	228.00	1.0000	74.00	74.00	0.0010	A

12	847092	chr12:717353	717353	C	T	0.01807	0.98193	0.01807	60.00	227.20	1.0000	36.15	76.51	0.0009	T
12	847102	chr12:717363	717363	T	G	0.00039	0.99961	0.00039	60.00	228.00	1.0000	66.00	66.00	0.0000	T
12	847140	chr12:717401	717401	A	G	0.00030	0.99970	0.00030	60.00	228.00	1.0000	49.54	49.54	0.0006	A
12	847186	chr12:717447	717447	C	T	0.00030	0.99970	0.00030	60.00	228.00	1.0000	54.75	54.75	0.0006	C
12	847190	chr12:717451	717451	C	T	0.00044	0.99956	0.00044	60.00	228.00	1.0000	46.00	46.00	0.0000	C
12	847380	chr12:717641	717641	G	C	0.00032	0.99968	0.00032	60.00	228.00	1.0000	25.08	25.08	0.0061	G
12	847580	chr12:717841	717841	G	A	0.00025	0.99975	0.00025	60.00	228.00	1.0000	23.00	23.00	0.0040	G
12	847585	chr12:717846	717846	G	A	0.00044	0.99956	0.00044	60.00	228.00	1.0000	30.00	30.00	0.0009	G
12	847599	chr12:717860	717860	T	C	0.09382	0.90618	0.09382	60.00	224.08	0.4120	22.85	56.58	0.0029	T
12	847704	chr12:717965	717965	G	A	0.00044	0.99956	0.00044	60.00	123.00	1.0000	15.00	15.00	0.0035	G
12	847707	chr12:717968	717968	G	A	0.00046	0.99954	0.00046	60.00	167.86	1.0000	17.07	21.11	0.0015	G
12	847771	chr12:718032	718032	G	A	0.00025	0.99975	0.00025	60.00	228.00	1.0000	50.00	50.00	0.0010	G
12	847796	chr12:718057	718057	T	C	0.00039	0.99961	0.00039	60.00	228.00	1.0000	47.00	47.00	0.0000	T
12	847841	chr12:718102	718102	G	T	0.13887	0.86113	0.13887	60.00	222.58	0.7175	26.45	67.38	0.0014	G
12	847896	chr12:718157	718157	G	T	0.00044	0.99956	0.00044	60.00	182.00	1.0000	59.00	59.00	0.0000	G
12	848022	chr12:718283	718283	C	T	0.00025	0.99975	0.00025	60.00	228.00	1.0000	45.00	45.00	0.0005	C
12	848148	chr12:718409	718409	A	G	0.00025	0.99975	0.00025	60.00	58.00	1.0000	10.00	10.00	0.0000	A
12	848195	chr12:718456	718456	C	A	0.00025	0.99975	0.00025	60.00	228.00	1.0000	47.00	47.00	0.0000	C
12	848213	chr12:718474	718474	C	G	0.00025	0.99975	0.00025	60.00	228.00	1.0000	59.00	59.00	0.0000	C
12	848255	chr12:718516	718516	T	C	0.00025	0.99975	0.00025	60.00	228.00	1.0000	64.00	64.00	0.0000	T
12	848332	chr12:718593	718593	G	A	0.00025	0.99975	0.00025	60.00	228.00	1.0000	56.00	56.00	0.0000	G
12	848352	chr12:718613	718613	G	A	0.00025	0.99975	0.00025	60.00	228.00	1.0000	64.00	64.00	0.0000	G
12	848412	chr12:718673	718673	A	G	0.00025	0.99975	0.00025	60.00	228.00	1.0000	64.00	64.00	0.0000	A
12	848418	chr12:718679	718679	G	A	0.00050	0.99950	0.00050	60.00	228.00	1.0000	69.18	75.83	0.0000	G
12	848427	chr12:718688	718688	C	T	0.00044	0.99956	0.00044	60.00	228.00	1.0000	74.00	74.00	0.0000	C
12	848428	chr12:718689	718689	G	A	0.00044	0.99956	0.00044	60.00	228.00	1.0000	59.00	59.00	0.0000	G
12	848449	chr12:718710	718710	A	G	0.00044	0.99956	0.00044	60.00	91.00	1.0000	49.00	49.00	0.0000	A
12	848453	chr12:718714	718714	C	T	0.00044	0.99956	0.00044	60.00	228.00	1.0000	68.00	68.00	0.0000	T
12	848497	chr12:718758	718758	G	A	0.00044	0.99956	0.00044	60.00	228.00	1.0000	63.00	63.00	0.0000	G
12	848507	chr12:718768	718768	A	G	0.00050	0.99950	0.00050	60.00	228.00	1.0000	47.18	53.83	0.0000	A
12	848872	chr12:719133	719133	C	T	0.00025	0.99975	0.00025	60.00	47.00	1.0000	10.00	10.00	0.0090	C
12	848874	chr12:719135	719135	C	T	0.00039	0.99961	0.00039	59.00	228.00	1.0000	20.00	20.00	0.0000	C
12	848887	chr12:719148	719148	A	C	0.00025	0.99975	0.00025	59.00	52.00	1.0000	11.00	11.00	0.0130	A
12	848891	chr12:719152	719152	G	A	0.06134	0.93866	0.06134	59.95	222.20	0.6366	21.00	52.18	0.0029	G

12	848940	chr12:719201	719201	G	A	0.08322	0.91678	0.08322	59.98	222.27	0.3771	25.06	55.17	0.0020	A
12	849250	chr12:719511	719511	A	G	0.00044	0.99956	0.00044	60.00	228.00	1.0000	29.00	29.00	0.0000	A
12	849308	chr12:719569	719569	G	C	0.00039	0.99961	0.00039	60.00	209.00	1.0000	30.00	30.00	0.0000	G
12	849310	chr12:719571	719571	G	A	0.00045	0.99955	0.00045	59.80	228.00	1.0000	33.97	36.96	0.0000	G
12	849324	chr12:719585	719585	G	A	0.00025	0.99975	0.00025	59.00	112.00	1.0000	18.00	18.00	0.0015	g
12	849328	chr12:719589	719589	G	A	0.00044	0.99956	0.00044	58.00	155.00	1.0000	17.00	17.00	0.0009	g
12	849682	chr12:719943	719943	C	T	0.00187	0.99813	0.00187	59.56	227.63	1.0000	14.40	25.18	0.0319	C
12	849755	chr12:720016	720016	G	C	0.00025	0.99975	0.00025	60.00	228.00	1.0000	55.00	55.00	0.0000	G
12	849905	chr12:720166	720166	C	T	0.00044	0.99956	0.00044	60.00	228.00	1.0000	68.00	68.00	0.0000	C
12	849960	chr12:720221	720221	A	G	0.00025	0.99975	0.00025	60.00	228.00	1.0000	74.00	74.00	0.0000	A
12	849962	chr12:720223	720223	T	C	0.00025	0.99975	0.00025	60.00	228.00	1.0000	83.00	83.00	0.0000	T
12	849977	chr12:720238	720238	C	T	0.00316	0.99684	0.00316	60.00	228.00	1.0000	61.35	83.56	0.0000	C
12	849997	chr12:720258	720258	C	T	0.00025	0.99975	0.00025	60.00	228.00	1.0000	78.00	78.00	0.0000	C
12	849999	chr12:720260	720260	A	G	0.00025	0.99975	0.00025	60.00	228.00	1.0000	76.00	76.00	0.0000	A
12	850033	chr12:720294	720294	T	C	0.00050	0.99950	0.00050	60.00	228.00	1.0000	32.73	60.28	0.0000	T
12	850060	chr12:720321	720321	T	C	0.00025	0.99975	0.00025	60.00	228.00	1.0000	80.00	80.00	0.0000	T
12	850065	chr12:720326	720326	G	A	0.00044	0.99956	0.00044	60.00	228.00	1.0000	81.00	81.00	0.0000	G
12	850076	chr12:720337	720337	G	A	0.00039	0.99961	0.00039	60.00	228.00	1.0000	58.00	58.00	0.0000	g
12	850082	chr12:720343	720343	G	A	0.00025	0.99975	0.00025	60.00	228.00	1.0000	67.00	67.00	0.0000	G
12	850089	chr12:720350	720350	C	T	0.00508	0.99492	0.00508	60.00	227.87	1.0000	48.35	78.98	0.0000	C
12	850094	chr12:720355	720355	C	T	0.00056	0.99944	0.00056	60.00	228.00	1.0000	68.86	71.87	0.0000	C
12	850122	chr12:720383	720383	C	T	0.01807	0.98193	0.01807	60.00	227.64	0.1289	30.30	71.07	0.0007	c
12	850126	chr12:720387	720387	C	T	0.00077	0.99923	0.00077	60.00	96.00	1.0000	21.05	22.95	0.0000	C
12	850133	chr12:720394	720394	C	T	0.01648	0.98352	0.01648	60.00	224.83	1.0000	23.53	65.27	0.0005	C
12	850170	chr12:720431	720431	G	A	0.00025	0.99975	0.00025	60.00	228.00	1.0000	53.00	53.00	0.0005	G
12	850201	chr12:720462	720462	C	T	0.00044	0.99956	0.00044	60.00	228.00	1.0000	62.00	62.00	0.0000	C
12	850211	chr12:720472	720472	C	T	0.00025	0.99975	0.00025	60.00	228.00	1.0000	61.00	61.00	0.0000	C
12	850288	chr12:720549	720549	C	T	0.44043	0.55957	0.44043	60.00	223.00	0.0832	38.57	85.35	0.0018	C
12	850296	chr12:720557	720557	T	G	0.00044	0.99956	0.00044	60.00	228.00	1.0000	66.00	66.00	0.0000	T
12	850352	chr12:720613	720613	A	G	0.00025	0.99975	0.00025	60.00	228.00	1.0000	57.00	57.00	0.0005	A
12	850382	chr12:720643	720643	C	T	0.00025	0.99975	0.00025	60.00	228.00	1.0000	61.00	61.00	0.0005	C
12	850386	chr12:720647	720647	C	G	0.00025	0.99975	0.00025	60.00	58.00	1.0000	27.00	27.00	0.0005	C
12	850387	chr12:720648	720648	A	T	0.00044	0.99956	0.00044	60.00	228.00	1.0000	67.00	67.00	0.0000	A
12	850392	chr12:720653	720653	C	T	0.00061	0.99939	0.00061	60.00	228.00	1.0000	34.21	38.31	0.0000	C

12	850397	chr12:720658	720658	T	A	0.00025	0.99975	0.00025	60.00	228.00	1.0000	45.00	45.00	0.0000	T
12	850419	chr12:720680	720680	T	C	0.06716	0.93284	0.06716	60.00	227.98	0.4892	44.64	84.06	0.0002	T
12	850442	chr12:720703	720703	C	T	0.00032	0.99968	0.00032	60.00	228.00	1.0000	82.81	82.81	0.0000	C
12	850472	chr12:720733	720733	A	G	0.00056	0.99944	0.00056	60.00	228.00	1.0000	77.95	82.77	0.0000	A
12	850490	chr12:720751	720751	C	G	0.00025	0.99975	0.00025	60.00	228.00	1.0000	68.00	68.00	0.0000	C
12	850613	chr12:720874	720874	T	C	0.01762	0.98238	0.01762	60.00	224.72	1.0000	21.51	55.23	0.0009	T
12	850626	chr12:720887	720887	T	C	0.00025	0.99975	0.00025	60.00	180.00	1.0000	28.00	28.00	0.0005	T
12	850632	chr12:720893	720893	C	A	0.00044	0.99956	0.00044	60.00	228.00	1.0000	32.00	32.00	0.0000	C
12	850638	chr12:720899	720899	C	G	0.00025	0.99975	0.00025	60.00	53.00	1.0000	16.00	16.00	0.0010	C
12	850689	chr12:720950	720950	A	G	0.01560	0.98440	0.01560	60.00	223.80	1.0000	22.83	62.87	0.0018	A
12	850818	chr12:721079	721079	G	A	0.00044	0.99956	0.00044	60.00	228.00	1.0000	70.00	70.00	0.0000	G
12	850866	chr12:721127	721127	C	T	0.00025	0.99975	0.00025	60.00	228.00	1.0000	62.00	62.00	0.0005	C
12	850907	chr12:721168	721168	C	A	0.00025	0.99975	0.00025	60.00	228.00	1.0000	43.00	43.00	0.0000	C
12	850936	chr12:721197	721197	C	A	0.00056	0.99944	0.00056	60.00	228.00	1.0000	40.76	46.33	0.0005	C
12	850945	chr12:721206	721206	T	G	0.00044	0.99956	0.00044	60.00	228.00	1.0000	32.00	32.00	0.0000	T
12	851252	chr12:721513	721513	G	A	0.00088	0.99912	0.00088	60.00	212.50	1.0000	11.58	33.43	0.0000	G
12	851278	chr12:721539	721539	A	G	0.00039	0.99961	0.00039	60.00	228.00	1.0000	59.00	59.00	0.0000	A
12	851299	chr12:721560	721560	C	G	0.00271	0.99729	0.00271	60.00	228.00	1.0000	52.03	83.56	0.0002	C
12	851324	chr12:721585	721585	T	C	0.00025	0.99975	0.00025	60.00	228.00	1.0000	68.00	68.00	0.0000	C
12	851356	chr12:721617	721617	C	T	0.00025	0.99975	0.00025	60.00	228.00	1.0000	49.00	49.00	0.0000	C
12	851372	chr12:721633	721633	A	T	0.00025	0.99975	0.00025	60.00	228.00	1.0000	72.00	72.00	0.0000	A
12	851384	chr12:721645	721645	A	C	0.00039	0.99961	0.00039	60.00	228.00	1.0000	45.00	45.00	0.0000	A
12	851385	chr12:721646	721646	T	C	0.00044	0.99956	0.00044	60.00	228.00	1.0000	73.00	73.00	0.0000	T
12	851393	chr12:721654	721654	A	G	0.00039	0.99961	0.00039	60.00	228.00	1.0000	70.00	70.00	0.0000	A
12	851408	chr12:721669	721669	G	T	0.00025	0.99975	0.00025	60.00	228.00	1.0000	85.00	85.00	0.0000	G
12	851416	chr12:721677	721677	C	T	0.99932	0.00068	0.00068	60.00	235.02	1.0000	43.54	91.67	0.0002	T
12	851482	chr12:721743	721743	C	T	0.00025	0.99975	0.00025	60.00	228.00	1.0000	51.00	51.00	0.0005	C
12	851489	chr12:721750	721750	G	A	0.00025	0.99975	0.00025	60.00	228.00	1.0000	74.00	74.00	0.0005	G
12	851511	chr12:721772	721772	A	G	0.00039	0.99961	0.00039	60.00	228.00	1.0000	70.00	70.00	0.0000	A
12	851553	chr12:721814	721814	C	T	0.00173	0.99827	0.00173	59.76	219.55	1.0000	17.90	25.45	0.0237	C
12	851849	chr12:722110	722110	A	G	0.00026	0.99974	0.00026	60.00	44.00	1.0000	10.00	10.00	0.0524	A
12	851893	chr12:722154	722154	A	G	0.00135	0.99865	0.00135	60.00	228.00	1.0000	49.17	68.39	0.0000	A
12	851920	chr12:722181	722181	T	C	0.00039	0.99961	0.00039	60.00	228.00	1.0000	75.00	75.00	0.0000	T
12	851972	chr12:722233	722233	A	G	0.00039	0.99961	0.00039	60.00	228.00	1.0000	84.00	84.00	0.0000	A

12	851989	chr12:722250	722250	T	A	0.00044	0.99956	0.00044	60.00	228.00	1.0000	82.00	82.00	0.0000	T
12	852029	chr12:722290	722290	G	A	0.00068	0.99932	0.00068	60.00	228.00	1.0000	43.51	52.24	0.0000	G
12	852051	chr12:722312	722312	C	T	0.00039	0.99961	0.00039	60.00	228.00	1.0000	68.00	68.00	0.0000	C
12	852111	chr12:722372	722372	G	T	0.00039	0.99961	0.00039	60.00	228.00	1.0000	68.00	68.00	0.0023	G
12	852139	chr12:722400	722400	G	T	0.00039	0.99961	0.00039	60.00	228.00	1.0000	57.00	57.00	0.0031	G
12	852146	chr12:722407	722407	T	G	0.00048	0.99952	0.00048	60.00	228.00	1.0000	66.67	73.19	0.0003	T
12	852243	chr12:722504	722504	T	G	0.00044	0.99956	0.00044	60.00	228.00	1.0000	69.00	69.00	0.0000	T
12	852251	chr12:722512	722512	C	T	0.00025	0.99975	0.00025	60.00	179.00	1.0000	12.00	12.00	0.0030	C
12	852637	chr12:722898	722898	G	C	0.00039	0.99961	0.00039	60.00	228.00	1.0000	42.00	42.00	0.0000	G
12	852730	chr12:722991	722991	C	T	0.00044	0.99956	0.00044	60.00	228.00	1.0000	53.00	53.00	0.0000	C
12	852738	chr12:722999	722999	C	T	0.00025	0.99975	0.00025	60.00	228.00	1.0000	43.00	43.00	0.0015	C
12	852790	chr12:723051	723051	A	G	0.00082	0.99918	0.00082	60.00	226.14	1.0000	23.50	30.60	0.0004	A
12	852814	chr12:723075	723075	T	C	0.00039	0.99961	0.00039	60.00	228.00	1.0000	27.00	27.00	0.0008	T
12	852881	chr12:723142	723142	G	A	0.00039	0.99961	0.00039	60.00	228.00	1.0000	73.00	73.00	0.0000	G
12	852961	chr12:723222	723222	C	T	0.00076	0.99924	0.00076	59.80	228.00	1.0000	60.36	71.76	0.0003	C
12	852962	chr12:723223	723223	G	A	0.00025	0.99975	0.00025	60.00	228.00	1.0000	50.00	50.00	0.0005	G
12	852991	chr12:723252	723252	G	A	0.00044	0.99956	0.00044	60.00	228.00	1.0000	90.00	90.00	0.0000	G
12	853118	chr12:723379	723379	A	G	0.00044	0.99956	0.00044	60.00	228.00	1.0000	58.00	58.00	0.0000	A
12	853154	chr12:723415	723415	T	C	0.00034	0.99966	0.00034	60.00	228.00	1.0000	51.79	51.79	0.0000	T
12	853156	chr12:723417	723417	C	G	0.00039	0.99961	0.00039	60.00	228.00	1.0000	50.00	50.00	0.0000	C
12	853160	chr12:723421	723421	C	G	0.00044	0.99956	0.00044	60.00	228.00	1.0000	63.00	63.00	0.0000	C
12	853176	chr12:723437	723437	G	C	0.00044	0.99956	0.00044	60.00	228.00	1.0000	38.00	38.00	0.0000	G
12	853191	chr12:723452	723452	A	G	0.00077	0.99923	0.00077	60.00	228.00	1.0000	31.35	44.65	0.0000	A
12	853205	chr12:723466	723466	C	G	0.00039	0.99961	0.00039	60.00	228.00	1.0000	47.00	47.00	0.0000	C
12	853228	chr12:723489	723489	G	A	0.00135	0.99865	0.00135	60.00	228.00	1.0000	54.92	75.72	0.0000	g
12	853318	chr12:723579	723579	T	C	0.00044	0.99956	0.00044	60.00	228.00	1.0000	50.00	50.00	0.0000	T
12	853358	chr12:723619	723619	G	A	0.00025	0.99975	0.00025	60.00	228.00	1.0000	52.00	52.00	0.0005	G
12	853411	chr12:723672	723672	T	C	0.00025	0.99975	0.00025	60.00	228.00	1.0000	48.00	48.00	0.0005	T
12	853434	chr12:723695	723695	T	G	0.00025	0.99975	0.00025	60.00	146.00	1.0000	16.00	16.00	0.0010	T
12	853443	chr12:723704	723704	T	C	0.00025	0.99975	0.00025	60.00	228.00	1.0000	38.00	38.00	0.0010	T
12	853656	chr12:723917	723917	A	G	0.00025	0.99975	0.00025	60.00	228.00	1.0000	37.00	37.00	0.0045	A
12	853692	chr12:723953	723953	C	T	0.00025	0.99975	0.00025	59.00	228.00	1.0000	39.00	39.00	0.0050	C
12	854270	chr12:724531	724531	G	T	0.00993	0.99007	0.00993	60.00	228.00	1.0000	59.07	93.05	0.0000	G
12	854275	chr12:724536	724536	G	A	0.00025	0.99975	0.00025	60.00	228.00	1.0000	85.00	85.00	0.0000	G

12	854331	chr12:724592	724592	T	C	0.48947	0.51053	0.48947	60.00	233.10	0.0894	50.28	94.75	0.0029	C
12	854353	chr12:724614	724614	T	C	0.06467	0.93533	0.06467	60.00	228.44	0.5721	55.67	96.02	0.0002	T
12	854492	chr12:724753	724753	T	C	0.99936	0.00064	0.00064	60.00	237.55	1.0000	45.92	93.28	0.0000	C
12	854522	chr12:724783	724783	A	C	0.00079	0.99921	0.00079	60.00	228.00	1.0000	50.41	74.63	0.0002	A
12	854535	chr12:724796	724796	C	T	0.00025	0.99975	0.00025	60.00	228.00	1.0000	59.00	59.00	0.0000	C
12	854640	chr12:724901	724901	T	G	0.00039	0.99961	0.00039	60.00	228.00	1.0000	22.00	22.00	0.0008	T
12	854645	chr12:724906	724906	A	C	0.00039	0.99961	0.00039	60.00	228.00	1.0000	20.00	20.00	0.0015	A
12	854956	chr12:725217	725217	G	A	0.00060	0.99940	0.00060	60.00	95.00	1.0000	14.03	14.98	0.1618	G
12	854977	chr12:725238	725238	T	C	0.00057	0.99943	0.00057	60.00	228.00	1.0000	37.03	40.03	0.0020	T
12	854996	chr12:725257	725257	T	C	0.00192	0.99808	0.00192	60.00	228.00	1.0000	46.39	71.66	0.0000	T
12	855088	chr12:725349	725349	A	T	0.00025	0.99975	0.00025	60.00	228.00	1.0000	35.00	35.00	0.0000	A
12	855168	chr12:725429	725429	G	T	0.00039	0.99961	0.00039	60.00	228.00	1.0000	43.00	43.00	0.0116	G
12	855370	chr12:725631	725631	C	T	0.00032	0.99968	0.00032	60.00	220.78	1.0000	44.22	44.22	0.0000	C
12	855468	chr12:725729	725729	T	A	0.00039	0.99961	0.00039	60.00	228.00	1.0000	37.00	37.00	0.0023	T
12	855598	chr12:725859	725859	T	G	0.00039	0.99961	0.00039	60.00	228.00	1.0000	39.00	39.00	0.0154	T
12	855599	chr12:725860	725860	T	C	0.00025	0.99975	0.00025	60.00	228.00	1.0000	36.00	36.00	0.0045	T
12	855600	chr12:725861	725861	C	G	0.00171	0.99829	0.00171	60.00	222.35	1.0000	14.28	32.15	0.0092	C
12	855625	chr12:725886	725886	G	A	0.00025	0.99975	0.00025	60.00	228.00	1.0000	12.00	12.00	0.0025	G
12	855654	chr12:725915	725915	A	C	0.00045	0.99955	0.00045	60.00	102.00	1.0000	10.00	10.00	0.0212	A
12	855670	chr12:725931	725931	G	A	0.00044	0.99956	0.00044	60.00	94.00	1.0000	29.00	29.00	0.0018	G
12	855702	chr12:725963	725963	G	A	0.00039	0.99961	0.00039	60.00	228.00	1.0000	65.00	65.00	0.0008	G
12	855721	chr12:725982	725982	C	T	0.00025	0.99975	0.00025	60.00	228.00	1.0000	75.00	75.00	0.0000	C
12	855755	chr12:726016	726016	G	A	0.00039	0.99961	0.00039	60.00	228.00	1.0000	55.00	55.00	0.0000	G
12	855765	chr12:726026	726026	G	A	0.00048	0.99952	0.00048	60.00	228.00	1.0000	59.56	63.80	0.0000	G
12	855836	chr12:726097	726097	C	G	0.00185	0.99815	0.00185	60.00	228.00	1.0000	43.89	54.26	0.0000	C
12	855865	chr12:726126	726126	A	C	0.00044	0.99956	0.00044	60.00	228.00	1.0000	42.00	42.00	0.0000	A
12	855869	chr12:726130	726130	C	T	0.00044	0.99956	0.00044	60.00	228.00	1.0000	32.00	32.00	0.0000	C
12	855872	chr12:726133	726133	T	C	0.00030	0.99970	0.00030	60.00	228.00	1.0000	45.43	45.43	0.0003	T
12	855884	chr12:726145	726145	G	A	0.00044	0.99956	0.00044	60.00	228.00	1.0000	42.00	42.00	0.0000	G
12	855885	chr12:726146	726146	T	A	0.00025	0.99975	0.00025	60.00	228.00	1.0000	63.00	63.00	0.0000	T
12	855895	chr12:726156	726156	A	G	0.00039	0.99961	0.00039	60.00	228.00	1.0000	24.00	24.00	0.0023	A
12	855896	chr12:726157	726157	C	A	0.00039	0.99961	0.00039	60.00	228.00	1.0000	22.00	22.00	0.0023	C
12	855905	chr12:726166	726166	C	T	0.00075	0.99925	0.00075	60.00	228.00	1.0000	43.15	69.75	0.0000	C
12	855995	chr12:726256	726256	G	A	0.00044	0.99956	0.00044	60.00	228.00	1.0000	39.00	39.00	0.0000	G

12	856031	chr12:726292	726292	G	A	0.00068	0.99932	0.00068	60.00	228.00	1.0000	53.84	64.29	0.0000	G
12	856041	chr12:726302	726302	T	C	0.00033	0.99967	0.00033	60.00	99.60	1.0000	41.47	41.47	0.0207	T
12	856053	chr12:726314	726314	G	A	0.00025	0.99975	0.00025	60.00	228.00	1.0000	81.00	81.00	0.0000	G
12	856079	chr12:726340	726340	T	C	0.00025	0.99975	0.00025	60.00	228.00	1.0000	79.00	79.00	0.0000	T
12	856132	chr12:726393	726393	G	A	0.00025	0.99975	0.00025	59.00	228.00	1.0000	41.00	41.00	0.0005	G
12	856301	chr12:726562	726562	T	C	0.00031	0.99969	0.00031	60.00	34.00	1.0000	10.00	10.00	0.1857	T
12	856307	chr12:726568	726568	T	C	0.00041	0.99959	0.00041	60.00	30.00	1.0000	10.00	10.00	0.0648	T
12	856309	chr12:726570	726570	A	T	0.00048	0.99952	0.00048	60.00	228.00	1.0000	17.00	17.00	0.0892	A
12	856311	chr12:726572	726572	A	G	0.00046	0.99954	0.00046	59.61	48.93	1.0000	19.62	22.96	0.0121	A
12	856342	chr12:726603	726603	C	T	0.00044	0.99956	0.00044	60.00	228.00	1.0000	48.00	48.00	0.0000	C
12	856454	chr12:726715	726715	T	A	0.00096	0.99904	0.00096	60.00	228.00	1.0000	52.76	66.08	0.0000	T
12	856508	chr12:726769	726769	A	G	0.00039	0.99961	0.00039	60.00	228.00	1.0000	77.00	77.00	0.0000	A
12	856526	chr12:726787	726787	C	A	0.00025	0.99975	0.00025	60.00	228.00	1.0000	55.00	55.00	0.0000	C
12	856529	chr12:726790	726790	A	C	0.00030	0.99970	0.00030	60.00	228.00	1.0000	65.25	65.25	0.0000	A
12	856552	chr12:726813	726813	C	T	0.00039	0.99961	0.00039	60.00	228.00	1.0000	61.00	61.00	0.0000	C
12	856583	chr12:726844	726844	T	C	0.00116	0.99884	0.00116	60.00	228.00	1.0000	76.05	83.65	0.0000	T
12	857151	chr12:727412	727412	G	T	0.00044	0.99956	0.00044	60.00	228.00	1.0000	36.00	36.00	0.0000	G
12	857192	chr12:727453	727453	A	G	0.00039	0.99961	0.00039	60.00	228.00	1.0000	54.00	54.00	0.0000	A
12	857203	chr12:727464	727464	C	T	0.01343	0.98657	0.01343	60.00	227.77	0.0132	34.63	85.25	0.0000	C
12	857206	chr12:727467	727467	T	C	0.00372	0.99628	0.00372	60.00	227.09	1.0000	46.93	79.29	0.0000	T
12	857224	chr12:727485	727485	A	G	0.53456	0.46544	0.46544	60.00	224.33	0.1354	40.23	86.10	0.0009	A
12	857248	chr12:727509	727509	A	G	0.00045	0.99955	0.00045	60.00	228.00	1.0000	81.23	85.84	0.0000	A
12	857269	chr12:727530	727530	C	T	0.00030	0.99970	0.00030	60.00	228.00	1.0000	69.11	69.11	0.0000	C
12	857273	chr12:727534	727534	T	C	0.00048	0.99952	0.00048	60.00	228.00	1.0000	56.13	60.93	0.0000	T
12	857285	chr12:727546	727546	C	T	0.00045	0.99955	0.00045	60.00	228.00	1.0000	65.63	67.94	0.0000	C
12	857301	chr12:727562	727562	C	T	0.00937	0.99063	0.00937	60.00	228.00	1.0000	35.72	79.33	0.0002	T
12	857351	chr12:727612	727612	T	G	0.00025	0.99975	0.00025	60.00	228.00	1.0000	19.00	19.00	0.0010	T
12	857356	chr12:727617	727617	A	G	0.00039	0.99961	0.00039	60.00	92.00	1.0000	14.00	14.00	0.0201	A
12	857707	chr12:727968	727968	A	C	0.00039	0.99961	0.00039	60.00	228.00	1.0000	21.00	21.00	0.0131	A
12	857719	chr12:727980	727980	T	C	0.00090	0.99910	0.00090	60.00	228.00	1.0000	41.21	44.76	0.0005	T
12	857734	chr12:727995	727995	C	A	0.00025	0.99975	0.00025	60.00	228.00	1.0000	67.00	67.00	0.0000	C
12	857762	chr12:728023	728023	A	C	0.00025	0.99975	0.00025	60.00	228.00	1.0000	53.00	53.00	0.0000	A
12	858065	chr12:728326	728326	T	C	0.00025	0.99975	0.00025	57.00	185.00	1.0000	23.00	23.00	0.0000	T
12	858101	chr12:728362	728362	C	T	0.00044	0.99956	0.00044	60.00	204.00	1.0000	65.00	65.00	0.0000	c

12	858179	chr12:728440	728440	T	C	0.00039	0.99961	0.00039	60.00	228.00	1.0000	69.00	69.00	0.0000	T
12	858251	chr12:728512	728512	T	C	0.00032	0.99968	0.00032	60.00	228.00	1.0000	73.81	73.81	0.0000	T
12	858321	chr12:728582	728582	T	C	0.00044	0.99956	0.00044	60.00	228.00	1.0000	39.00	39.00	0.0000	T
12	858462	chr12:728723	728723	T	C	0.00044	0.99956	0.00044	60.00	228.00	1.0000	21.00	21.00	0.0000	T
12	858465	chr12:728726	728726	A	G	0.00077	0.99923	0.00077	60.00	228.00	1.0000	52.43	68.58	0.0008	A
12	858494	chr12:728755	728755	T	C	0.00025	0.99975	0.00025	60.00	228.00	1.0000	67.00	67.00	0.0000	T
12	858536	chr12:728797	728797	C	T	0.00030	0.99970	0.00030	60.00	228.00	1.0000	69.71	69.71	0.0006	C
12	858623	chr12:728884	728884	G	T	0.00025	0.99975	0.00025	60.00	228.00	1.0000	46.00	46.00	0.0005	G
12	858629	chr12:728890	728890	T	C	0.00025	0.99975	0.00025	60.00	228.00	1.0000	46.00	46.00	0.0010	T
12	858654	chr12:728915	728915	C	T	0.00039	0.99961	0.00039	60.00	228.00	1.0000	54.00	54.00	0.0031	C
12	858662	chr12:728923	728923	C	T	0.00039	0.99961	0.00039	60.00	228.00	1.0000	45.00	45.00	0.0046	C
12	858759	chr12:729020	729020	C	T	0.00044	0.99956	0.00044	60.00	228.00	1.0000	13.00	13.00	0.0044	T
12	858821	chr12:729082	729082	A	C	0.01459	0.98541	0.01459	59.90	213.03	1.0000	11.51	28.07	0.0315	A
12	858831	chr12:729092	729092	A	G	0.00052	0.99948	0.00052	60.00	65.00	1.0000	10.00	10.00	0.0320	A
12	858834	chr12:729095	729095	A	C	0.00026	0.99974	0.00026	60.00	60.00	1.0000	10.00	10.00	0.0300	A
12	858841	chr12:729102	729102	A	G	0.00129	0.99871	0.00129	60.00	74.20	1.0000	10.00	14.70	0.0339	A
12	858844	chr12:729105	729105	A	G	0.00025	0.99975	0.00025	59.00	30.00	1.0000	10.00	10.00	0.0115	A
12	859000	chr12:729261	729261	C	G	0.00025	0.99975	0.00025	60.00	228.00	1.0000	57.00	57.00	0.0000	C
12	859062	chr12:729323	729323	A	G	0.00044	0.99956	0.00044	60.00	228.00	1.0000	46.00	46.00	0.0009	A
12	859147	chr12:729408	729408	T	C	0.00045	0.99955	0.00045	60.00	228.00	1.0000	57.96	57.96	0.0000	T
12	859166	chr12:729427	729427	G	A	0.00100	0.99900	0.00100	60.00	228.00	1.0000	45.13	62.78	0.0000	G
12	859250	chr12:729511	729511	G	C	0.03859	0.96141	0.03859	60.00	227.49	0.2368	46.72	91.03	0.0000	G
12	859259	chr12:729520	729520	G	C	0.00226	0.99774	0.00226	60.00	228.13	1.0000	56.81	84.56	0.0000	G
12	859274	chr12:729535	729535	C	T	0.00025	0.99975	0.00025	60.00	228.00	1.0000	80.00	80.00	0.0000	C
12	859293	chr12:729554	729554	G	C	0.00034	0.99966	0.00034	60.00	228.00	1.0000	67.15	67.15	0.0000	G
12	859302	chr12:729563	729563	A	G	0.00044	0.99956	0.00044	60.00	228.00	1.0000	47.00	47.00	0.0000	A
12	859416	chr12:729677	729677	C	A	0.00048	0.99952	0.00048	57.65	228.00	1.0000	21.44	23.28	0.0107	C
12	859451	chr12:729712	729712	C	T	0.28366	0.71634	0.28366	58.63	190.71	0.0054	11.21	32.89	0.0355	C
12	859466	chr12:729727	729727	A	C	0.00039	0.99961	0.00039	60.00	228.00	1.0000	17.00	17.00	0.0062	A
12	859474	chr12:729735	729735	A	C	0.04281	0.95719	0.04281	59.97	219.79	0.3940	12.80	39.10	0.0166	A
12	859622	chr12:729883	729883	G	T	0.00045	0.99955	0.00045	60.00	228.00	1.0000	55.25	56.75	0.0000	G
12	859717	chr12:729978	729978	T	G	0.00048	0.99952	0.00048	60.00	228.00	1.0000	66.24	68.06	0.0000	T
12	859736	chr12:729997	729997	T	A	0.00039	0.99961	0.00039	60.00	228.00	1.0000	22.00	22.00	0.0000	T
12	859754	chr12:730015	730015	T	C	0.00025	0.99975	0.00025	60.00	228.00	1.0000	60.00	60.00	0.0000	T

12	859756	chr12:730017	730017	A	T	0.00045	0.99955	0.00045	60.00	228.00	1.0000	53.72	54.10	0.0000	A
12	859825	chr12:730086	730086	T	G	0.00039	0.99961	0.00039	60.00	228.00	1.0000	58.00	58.00	0.0000	T
12	859837	chr12:730098	730098	C	A	0.03733	0.96267	0.03733	60.00	222.70	0.4790	26.33	67.64	0.0016	C
12	859843	chr12:730104	730104	G	C	0.00041	0.99959	0.00041	60.00	228.00	1.0000	56.41	56.41	0.0000	G
12	859874	chr12:730135	730135	A	G	0.00025	0.99975	0.00025	60.00	228.00	1.0000	74.00	74.00	0.0000	A
12	859937	chr12:730198	730198	C	G	0.00039	0.99961	0.00039	60.00	228.00	1.0000	50.00	50.00	0.0031	C
12	860020	chr12:730281	730281	C	T	0.00039	0.99961	0.00039	60.00	228.00	1.0000	42.00	42.00	0.0054	C
12	860033	chr12:730294	730294	A	C	0.00136	0.99864	0.00136	60.00	228.00	1.0000	28.67	48.37	0.0018	A
12	860119	chr12:730380	730380	A	G	0.00032	0.99968	0.00032	60.00	228.00	1.0000	54.72	54.72	0.0003	A
12	860156	chr12:730417	730417	G	A	0.00045	0.99955	0.00045	60.00	63.56	1.0000	17.83	19.12	0.0029	G
12	860172	chr12:730433	730433	C	G	0.00039	0.99961	0.00039	60.00	67.00	1.0000	20.00	20.00	0.0008	C
12	860190	chr12:730451	730451	T	G	0.00039	0.99961	0.00039	60.00	228.00	1.0000	36.00	36.00	0.0000	T
12	860226	chr12:730487	730487	A	T	0.00025	0.99975	0.00025	60.00	228.00	1.0000	33.00	33.00	0.0015	A
12	860242	chr12:730503	730503	C	T	0.00158	0.99842	0.00158	60.00	228.00	1.0000	32.28	47.71	0.0020	C
12	860265	chr12:730526	730526	A	T	0.00045	0.99955	0.00045	60.00	192.00	1.0000	24.00	24.00	0.0088	A
12	860279	chr12:730540	730540	T	C	0.00050	0.99950	0.00050	60.00	228.00	1.0000	33.13	37.88	0.0020	T
12	860293	chr12:730554	730554	T	C	0.00068	0.99932	0.00068	60.00	228.00	1.0000	18.82	35.38	0.0029	T
12	860301	chr12:730562	730562	C	G	0.00044	0.99956	0.00044	60.00	228.00	1.0000	26.00	26.00	0.0044	C
12	860531	chr12:730792	730792	G	A	0.00026	0.99974	0.00026	60.00	228.00	1.0000	38.00	38.00	0.0265	G
12	860594	chr12:730855	730855	C	A	0.00026	0.99974	0.00026	60.00	80.00	1.0000	10.00	10.00	0.0379	C
12	860596	chr12:730857	730857	C	A	0.00496	0.99504	0.00496	60.00	80.29	1.0000	13.01	33.73	0.0785	C
12	860644	chr12:730905	730905	G	T	0.00047	0.99953	0.00047	60.00	31.00	1.0000	10.00	10.00	0.0521	G
12	860653	chr12:730914	730914	C	A	0.00026	0.99974	0.00026	60.00	228.00	1.0000	25.00	25.00	0.0220	C
12	860684	chr12:730945	730945	A	G	0.00039	0.99961	0.00039	60.00	228.00	1.0000	32.00	32.00	0.0008	A
12	860734	chr12:730995	730995	G	C	0.00039	0.99961	0.00039	60.00	228.00	1.0000	21.00	21.00	0.0000	G
12	860748	chr12:731009	731009	A	T	0.00039	0.99961	0.00039	60.00	228.00	1.0000	47.00	47.00	0.0000	A
12	860765	chr12:731026	731026	A	G	0.00025	0.99975	0.00025	60.00	228.00	1.0000	44.00	44.00	0.0020	A
12	860850	chr12:731111	731111	C	T	0.00025	0.99975	0.00025	60.00	228.00	1.0000	27.00	27.00	0.0000	C
12	860875	chr12:731136	731136	A	T	0.00025	0.99975	0.00025	60.00	228.00	1.0000	52.00	52.00	0.0000	A
12	860882	chr12:731143	731143	G	A	0.00045	0.99955	0.00045	60.00	93.39	1.0000	24.55	30.56	0.0025	G
12	860930	chr12:731191	731191	C	T	0.00025	0.99975	0.00025	60.00	228.00	1.0000	53.00	53.00	0.0010	C
12	860938	chr12:731199	731199	A	G	0.00044	0.99956	0.00044	60.00	228.00	1.0000	29.00	29.00	0.0044	A
12	860992	chr12:731253	731253	C	T	0.00044	0.99956	0.00044	60.00	96.00	1.0000	12.00	12.00	0.0027	C
12	861002	chr12:731263	731263	T	C	0.00039	0.99961	0.00039	60.00	228.00	1.0000	67.00	67.00	0.0000	T

12	861003	chr12:731264	731264	T	C	0.00065	0.99935	0.00065	60.00	78.54	1.0000	38.60	45.45	0.0198	T
12	861028	chr12:731289	731289	C	A	0.00050	0.99950	0.00050	60.00	228.00	1.0000	35.35	48.65	0.0005	C
12	861054	chr12:731315	731315	G	T	0.00025	0.99975	0.00025	60.00	228.00	1.0000	18.00	18.00	0.0010	G
12	861063	chr12:731324	731324	A	C	0.00039	0.99961	0.00039	60.00	228.00	1.0000	42.00	42.00	0.0000	A
12	861096	chr12:731357	731357	C	T	0.00025	0.99975	0.00025	60.00	228.00	1.0000	37.00	37.00	0.0005	C
12	861105	chr12:731366	731366	G	A	0.07997	0.92003	0.07997	60.00	223.13	0.0258	24.99	67.06	0.0038	A
12	861177	chr12:731438	731438	C	G	0.03606	0.96394	0.03606	60.00	223.93	0.6219	20.39	59.33	0.0078	C
12	861188	chr12:731449	731449	C	A	0.00045	0.99955	0.00045	60.00	228.00	1.0000	27.00	27.00	0.0186	C
12	861190	chr12:731451	731451	G	A	0.00025	0.99975	0.00025	60.00	228.00	1.0000	49.00	49.00	0.0060	G
12	861195	chr12:731456	731456	G	A	0.00025	0.99975	0.00025	60.00	228.00	1.0000	52.00	52.00	0.0055	G
12	861227	chr12:731488	731488	C	T	0.00025	0.99975	0.00025	60.00	228.00	1.0000	11.00	11.00	0.0050	C
12	861254	chr12:731515	731515	T	C	0.00025	0.99975	0.00025	60.00	112.00	1.0000	36.00	36.00	0.0110	T
12	861340	chr12:731601	731601	C	T	0.00129	0.99871	0.00129	60.00	124.50	0.0011	11.05	12.95	0.1057	C
12	861448	chr12:731709	731709	G	A	0.00030	0.99970	0.00030	60.00	228.00	1.0000	14.00	14.00	0.1687	G
12	861528	chr12:731789	731789	A	G	0.00039	0.99961	0.00039	60.00	228.00	1.0000	45.00	45.00	0.0000	A
12	861536	chr12:731797	731797	C	T	0.00226	0.99774	0.00226	60.00	224.12	1.0000	19.72	38.19	0.0504	C
12	861581	chr12:731842	731842	G	A	0.00049	0.99951	0.00049	60.00	98.00	1.0000	11.00	11.00	0.1069	G
12	861619	chr12:731880	731880	C	G	0.00054	0.99946	0.00054	60.00	228.00	1.0000	12.00	12.00	0.1758	C
12	861841	chr12:732102	732102	T	C	0.00156	0.99844	0.00156	60.00	102.25	1.0000	13.00	14.93	0.0108	T
12	861889	chr12:732150	732150	C	T	0.00077	0.99923	0.00077	60.00	85.00	1.0000	17.30	28.70	0.0031	C
12	861907	chr12:732168	732168	C	T	0.00039	0.99961	0.00039	60.00	228.00	1.0000	27.00	27.00	0.0193	C
12	863415	chr12:733676	733676	C	T	0.00047	0.99953	0.00047	60.00	228.00	1.0000	37.00	37.00	0.0636	C
12	863505	chr12:733766	733766	A	C	0.01537	0.98463	0.01537	60.00	221.13	1.0000	18.33	53.12	0.0300	A
12	863510	chr12:733771	733771	A	C	0.00026	0.99974	0.00026	60.00	228.00	1.0000	28.00	28.00	0.0454	A
12	863517	chr12:733778	733778	G	A	0.69211	0.30789	0.30789	60.00	170.92	0.4147	17.27	53.77	0.0641	G
12	863537	chr12:733798	733798	T	C	0.00042	0.99958	0.00042	60.00	91.03	1.0000	28.70	28.70	0.0189	T
12	863538	chr12:733799	733799	G	A	0.00027	0.99973	0.00027	60.00	46.00	1.0000	10.00	10.00	0.0659	G
12	863591	chr12:733852	733852	G	A	0.00357	0.99643	0.00357	60.00	226.98	1.0000	19.67	53.58	0.0503	G
12	863594	chr12:733855	733855	T	A	0.00047	0.99953	0.00047	60.00	228.00	1.0000	24.00	24.00	0.0689	T
12	863614	chr12:733875	733875	C	A	0.00028	0.99972	0.00028	60.00	63.00	1.0000	10.00	10.00	0.0989	C
12	863639	chr12:733900	733900	G	A	0.00025	0.99975	0.00025	60.00	228.00	1.0000	45.00	45.00	0.0095	G
12	863646	chr12:733907	733907	G	A	0.00039	0.99961	0.00039	60.00	228.00	1.0000	76.00	76.00	0.0000	G
12	863649	chr12:733910	733910	G	A	0.00089	0.99911	0.00089	60.00	228.00	1.0000	30.23	38.78	0.0124	G
12	863680	chr12:733941	733941	G	C	0.00039	0.99961	0.00039	60.00	228.00	1.0000	68.00	68.00	0.0000	G

12	863696	chr12:733957	733957	G	C	0.00045	0.99955	0.00045	60.00	228.00	1.0000	49.00	49.00	0.0124	G
12	863725	chr12:733986	733986	A	G	0.00045	0.99955	0.00045	60.00	35.00	1.0000	10.00	10.00	0.0106	A
12	863752	chr12:734013	734013	C	A	0.00077	0.99923	0.00077	60.00	228.00	1.0000	69.10	72.90	0.0000	C
12	863789	chr12:734050	734050	T	C	0.00050	0.99950	0.00050	60.00	228.00	1.0000	26.50	45.50	0.0050	T
12	863814	chr12:734075	734075	T	G	0.00050	0.99950	0.00050	60.00	228.00	1.0000	41.13	45.88	0.0050	T
12	863833	chr12:734094	734094	A	G	0.70085	0.29915	0.29915	60.00	189.51	0.6963	20.20	64.98	0.0184	A
12	863863	chr12:734124	734124	G	A	0.00077	0.99923	0.00077	60.00	228.00	1.0000	62.18	68.83	0.0000	G
12	863902	chr12:734163	734163	G	C	0.00025	0.99975	0.00025	60.00	228.00	1.0000	61.00	61.00	0.0030	G
12	863990	chr12:734251	734251	A	T	0.00033	0.99967	0.00033	60.00	187.37	1.0000	15.11	15.11	0.0384	A
12	864001	chr12:734262	734262	C	T	0.00045	0.99955	0.00045	60.00	228.00	1.0000	20.00	20.00	0.0239	C
12	864010	chr12:734271	734271	C	T	0.00025	0.99975	0.00025	60.00	228.00	1.0000	20.00	20.00	0.0200	C
12	864028	chr12:734289	734289	G	A	0.00025	0.99975	0.00025	60.00	207.00	1.0000	38.00	38.00	0.0105	G
12	864042	chr12:734303	734303	T	C	0.00025	0.99975	0.00025	60.00	228.00	1.0000	32.00	32.00	0.0045	T
12	864116	chr12:734377	734377	C	T	0.00039	0.99961	0.00039	60.00	228.00	1.0000	64.00	64.00	0.0000	C
12	864119	chr12:734380	734380	C	A	0.00044	0.99956	0.00044	60.00	228.00	1.0000	30.00	30.00	0.0071	C
12	864148	chr12:734409	734409	T	G	0.00044	0.99956	0.00044	60.00	46.00	1.0000	11.00	11.00	0.0035	T
12	864181	chr12:734442	734442	A	G	0.00046	0.99954	0.00046	60.00	146.40	1.0000	16.87	17.62	0.0018	A
12	864183	chr12:734444	734444	T	C	0.00125	0.99875	0.00125	60.00	160.41	1.0000	13.39	29.46	0.0090	T
12	864316	chr12:734577	734577	A	G	0.00039	0.99961	0.00039	60.00	228.00	1.0000	67.00	67.00	0.0000	A
12	864376	chr12:734637	734637	C	A	0.00057	0.99943	0.00057	60.00	228.00	1.0000	34.91	37.81	0.0041	C
12	864384	chr12:734645	734645	G	A	0.00045	0.99955	0.00045	60.00	228.00	1.0000	51.00	51.00	0.0106	G
12	864436	chr12:734697	734697	T	G	0.00048	0.99952	0.00048	60.00	228.00	1.0000	15.00	15.00	0.0769	T
12	864447	chr12:734708	734708	A	G	0.00145	0.99855	0.00145	60.00	104.96	1.0000	11.18	13.75	0.0568	A
12	864460	chr12:734721	734721	A	T	0.00039	0.99961	0.00039	60.00	43.00	1.0000	10.00	10.00	0.0054	A
12	864477	chr12:734738	734738	T	C	0.14042	0.85958	0.14042	60.00	210.19	0.3594	13.33	39.60	0.0316	-
12	864489	chr12:734750	734750	C	A	0.00025	0.99975	0.00025	60.00	228.00	1.0000	39.00	39.00	0.0035	C
12	864520	chr12:734781	734781	G	A	0.03316	0.96684	0.03316	60.00	220.16	0.2989	19.35	60.04	0.0063	G
12	864590	chr12:734851	734851	G	A	0.00025	0.99975	0.00025	60.00	228.00	1.0000	48.00	48.00	0.0020	G
12	864627	chr12:734888	734888	A	C	0.00068	0.99932	0.00068	60.00	228.00	1.0000	40.89	43.47	0.0059	A
12	864633	chr12:734894	734894	C	T	0.00045	0.99955	0.00045	60.00	228.00	1.0000	34.00	34.00	0.0097	C
12	864669	chr12:734930	734930	T	G	0.00025	0.99975	0.00025	60.00	228.00	1.0000	22.00	22.00	0.0055	T
12	864729	chr12:734990	734990	G	A	0.00025	0.99975	0.00025	60.00	228.00	1.0000	27.00	27.00	0.0185	G
12	864793	chr12:735054	735054	A	T	0.00027	0.99973	0.00027	60.00	39.00	1.0000	11.00	11.00	0.0739	A
12	864808	chr12:735069	735069	C	A	0.00026	0.99974	0.00026	60.00	228.00	1.0000	25.00	25.00	0.0220	C

12	865148	chr12:735409	735409	A	G	0.00076	0.99924	0.00076	59.00	148.50	0.0005	13.13	17.88	0.0090	A
12	865164	chr12:735425	735425	C	A	0.00914	0.99086	0.00914	59.52	129.56	0.0443	11.65	27.74	0.0505	C
12	865235	chr12:735496	735496	A	G	0.00169	0.99831	0.00169	60.00	228.00	1.0000	54.26	74.98	0.0000	A
12	865341	chr12:735602	735602	T	C	0.00044	0.99956	0.00044	60.00	228.00	1.0000	60.00	60.00	0.0000	T
12	865354	chr12:735615	735615	C	T	0.00221	0.99779	0.00221	60.00	197.25	1.0000	25.15	39.33	0.0000	C
12	865412	chr12:735673	735673	C	A	0.00025	0.99975	0.00025	60.00	228.00	1.0000	42.00	42.00	0.0005	C
12	865426	chr12:735687	735687	C	G	0.00192	0.99808	0.00192	60.00	228.00	1.0000	31.74	78.75	0.0002	C
12	865497	chr12:735758	735758	A	G	0.00039	0.99961	0.00039	60.00	228.00	1.0000	48.00	48.00	0.0023	A
12	865506	chr12:735767	735767	A	C	0.00044	0.99956	0.00044	60.00	228.00	1.0000	29.00	29.00	0.0027	A
12	865615	chr12:735876	735876	T	C	0.00090	0.99910	0.00090	60.00	228.00	1.0000	43.19	57.74	0.0005	T
12	865633	chr12:735894	735894	T	C	0.00068	0.99932	0.00068	60.00	228.00	1.0000	42.82	47.91	0.0009	T
12	865719	chr12:735980	735980	A	C	0.00044	0.99956	0.00044	60.00	228.00	1.0000	50.00	50.00	0.0009	A
12	865789	chr12:736050	736050	G	A	0.00039	0.99961	0.00039	60.00	228.00	1.0000	65.00	65.00	0.0000	G
12	865869	chr12:736130	736130	T	C	0.00039	0.99961	0.00039	60.00	228.00	1.0000	52.00	52.00	0.0000	T
12	865876	chr12:736137	736137	G	A	0.00044	0.99956	0.00044	60.00	228.00	1.0000	22.00	22.00	0.0000	G
12	865894	chr12:736155	736155	T	G	0.00056	0.99944	0.00056	60.00	228.00	1.0000	38.12	39.51	0.0007	T
12	865900	chr12:736161	736161	T	C	0.00044	0.99956	0.00044	60.00	228.00	1.0000	30.00	30.00	0.0009	T
12	865925	chr12:736186	736186	A	G	0.00050	0.99950	0.00050	60.00	228.00	1.0000	37.50	56.50	0.0010	A
12	865927	chr12:736188	736188	G	A	0.00039	0.99961	0.00039	60.00	228.00	1.0000	48.00	48.00	0.0000	G
12	865948	chr12:736209	736209	G	C	0.00075	0.99925	0.00075	60.00	228.00	1.0000	24.15	59.30	0.0010	G
12	865963	chr12:736224	736224	G	A	0.00238	0.99762	0.00238	60.00	227.46	1.0000	21.33	43.62	0.0036	G
12	865970	chr12:736231	736231	C	G	0.76373	0.23627	0.23627	60.00	171.53	0.0007	16.91	59.99	0.0099	G
12	865989	chr12:736250	736250	A	G	0.00044	0.99956	0.00044	60.00	228.00	1.0000	35.00	35.00	0.0027	A
12	866084	chr12:736345	736345	C	A	0.00044	0.99956	0.00044	60.00	162.00	1.0000	14.00	14.00	0.0044	C
12	866089	chr12:736350	736350	A	G	0.00025	0.99975	0.00025	60.00	102.00	1.0000	15.00	15.00	0.0005	A
12	866090	chr12:736351	736351	T	C	0.00025	0.99975	0.00025	60.00	81.00	1.0000	20.00	20.00	0.0135	T
12	866103	chr12:736364	736364	T	C	0.00025	0.99975	0.00025	60.00	228.00	1.0000	44.00	44.00	0.0005	T
12	866107	chr12:736368	736368	G	A	0.00025	0.99975	0.00025	60.00	184.00	1.0000	34.00	34.00	0.0005	G
12	866129	chr12:736390	736390	T	C	0.00025	0.99975	0.00025	60.00	228.00	1.0000	62.00	62.00	0.0000	T
12	866208	chr12:736469	736469	C	T	0.06448	0.93552	0.06448	60.00	226.58	0.2601	43.30	85.55	0.0007	C
12	866236	chr12:736497	736497	A	G	0.00090	0.99910	0.00090	60.00	228.00	1.0000	60.28	67.05	0.0000	A
12	866289	chr12:736550	736550	C	T	0.00039	0.99961	0.00039	60.00	228.00	1.0000	81.00	81.00	0.0000	c
12	866381	chr12:736642	736642	G	A	0.00025	0.99975	0.00025	60.00	228.00	1.0000	73.00	73.00	0.0000	G
12	866399	chr12:736660	736660	A	G	0.00044	0.99956	0.00044	60.00	228.00	1.0000	35.00	35.00	0.0000	A

12	866407	chr12:736668	736668	A	C	0.00025	0.99975	0.00025	60.00	228.00	1.0000	80.00	80.00	0.0000	A
12	866429	chr12:736690	736690	T	A	0.00025	0.99975	0.00025	60.00	54.00	1.0000	18.00	18.00	0.0010	T
12	866637	chr12:736898	736898	G	A	0.00028	0.99972	0.00028	60.00	228.00	1.0000	17.00	17.00	0.0949	G
12	866665	chr12:736926	736926	G	A	0.00039	0.99961	0.00039	60.00	228.00	1.0000	40.00	40.00	0.0000	G
12	866682	chr12:736943	736943	A	G	0.00260	0.99740	0.00260	60.00	227.97	1.0000	31.21	61.85	0.0005	A
12	866765	chr12:737026	737026	T	C	0.00032	0.99968	0.00032	60.00	228.00	1.0000	41.64	41.64	0.0000	T
12	866791	chr12:737052	737052	T	A	0.00025	0.99975	0.00025	60.00	34.00	1.0000	16.00	16.00	0.0005	T
12	866792	chr12:737053	737053	T	A	0.00387	0.99613	0.00387	60.00	79.52	1.0000	19.80	30.19	0.0074	T
12	866817	chr12:737078	737078	A	G	0.00050	0.99950	0.00050	60.00	247.00	0.0005	74.00	74.00	0.0110	A
12	866927	chr12:737188	737188	G	A	0.00079	0.99921	0.00079	60.00	99.00	0.0010	24.00	24.00	0.0247	G
12	867314	chr12:737575	737575	G	A	0.00045	0.99955	0.00045	60.00	228.00	1.0000	96.00	96.00	0.0177	G
12	867337	chr12:737598	737598	C	A	0.00025	0.99975	0.00025	60.00	228.00	1.0000	72.00	72.00	0.0150	C
12	867364	chr12:737625	737625	A	G	0.00039	0.99961	0.00039	60.00	228.00	1.0000	54.00	54.00	0.0216	A
12	867403	chr12:737664	737664	A	G	0.00025	0.99975	0.00025	60.00	228.00	1.0000	80.00	80.00	0.0160	A
12	867755	chr12:738016	738016	T	A	0.00026	0.99974	0.00026	60.00	139.00	1.0000	20.00	20.00	0.0290	-
12	867764	chr12:738025	738025	A	G	0.00051	0.99949	0.00051	59.00	96.00	0.0005	23.00	23.00	0.0165	-
12	867866	chr12:738127	738127	C	G	0.00025	0.99975	0.00025	60.00	228.00	1.0000	84.00	84.00	0.0160	C
12	867891	chr12:738152	738152	C	T	0.03235	0.96765	0.03235	60.00	219.98	0.0001	29.97	89.40	0.0198	C
12	867901	chr12:738162	738162	C	T	0.00025	0.99975	0.00025	60.00	228.00	1.0000	97.00	97.00	0.0160	C
12	867915	chr12:738176	738176	G	T	0.00689	0.99311	0.00689	60.00	222.51	0.0001	38.16	87.89	0.0181	G
12	867931	chr12:738192	738192	C	T	0.00025	0.99975	0.00025	60.00	228.00	1.0000	69.00	69.00	0.0150	C
12	867951	chr12:738212	738212	G	A	0.00045	0.99955	0.00045	60.00	228.00	1.0000	54.00	54.00	0.0177	G
12	867985	chr12:738246	738246	T	C	0.00045	0.99955	0.00045	60.00	60.00	1.0000	10.00	10.00	0.0194	T
12	868007	chr12:738268	738268	C	T	0.00139	0.99861	0.00139	60.00	219.91	0.0053	49.68	77.20	0.0182	C
12	868012	chr12:738273	738273	T	G	0.00025	0.99975	0.00025	60.00	228.00	1.0000	86.00	86.00	0.0155	T
12	868040	chr12:738301	738301	G	A	0.00045	0.99955	0.00045	60.00	228.00	1.0000	36.00	36.00	0.0186	G
12	868089	chr12:738350	738350	A	G	0.00025	0.99975	0.00025	60.00	228.00	1.0000	76.00	76.00	0.0160	A
12	868093	chr12:738354	738354	A	G	0.00025	0.99975	0.00025	60.00	228.00	1.0000	58.00	58.00	0.0160	A
12	868097	chr12:738358	738358	C	T	0.00090	0.99910	0.00090	60.00	181.50	1.0000	24.28	34.73	0.0239	C
12	868225	chr12:738486	738486	T	A	0.00046	0.99954	0.00046	60.00	218.00	1.0000	34.00	34.00	0.0486	T
12	868246	chr12:738507	738507	A	T	0.00025	0.99975	0.00025	60.00	228.00	1.0000	53.00	53.00	0.0160	A
12	868292	chr12:738553	738553	T	G	0.00040	0.99960	0.00040	60.00	228.00	1.0000	44.00	44.00	0.0262	T
12	868294	chr12:738555	738555	G	T	0.00040	0.99960	0.00040	60.00	228.00	1.0000	67.00	67.00	0.0262	G
12	868316	chr12:738577	738577	T	A	0.00253	0.99747	0.00253	60.00	204.84	0.0000	31.39	63.52	0.0194	T

12	868322	chr12:738583	738583	T	A	0.04505	0.95495	0.04505	60.00	200.78	0.0002	16.00	65.00	0.0194	T
12	868384	chr12:738645	738645	G	A	0.00033	0.99967	0.00033	60.00	228.00	1.0000	36.75	36.75	0.0291	G
12	868828	chr12:739089	739089	A	G	0.01668	0.98332	0.01668	60.00	215.75	0.0058	22.65	69.62	0.0192	A
12	868853	chr12:739114	739114	G	C	0.03784	0.96216	0.03784	60.00	218.16	0.0046	21.00	76.20	0.0194	G
12	868937	chr12:739198	739198	A	G	0.00092	0.99908	0.00092	60.00	208.22	0.0022	48.31	65.19	0.0185	A
12	869003	chr12:739264	739264	A	G	0.00050	0.99950	0.00050	60.00	60.00	1.0000	10.00	10.00	0.1228	A
12	869015	chr12:739276	739276	T	C	0.00040	0.99960	0.00040	60.00	38.00	1.0000	15.00	15.00	0.0324	T
12	869019	chr12:739280	739280	A	T	0.00047	0.99953	0.00047	60.00	91.00	1.0000	24.00	24.00	0.0627	A
12	869024	chr12:739285	739285	T	C	0.00046	0.99954	0.00046	60.00	228.00	1.0000	34.17	40.53	0.0191	T
12	869082	chr12:739343	739343	A	C	0.00039	0.99961	0.00039	60.00	228.00	1.0000	60.00	60.00	0.0216	A
12	869092	chr12:739353	739353	T	C	0.00045	0.99955	0.00045	60.00	66.00	1.0000	12.00	12.00	0.0177	T
12	869103	chr12:739364	739364	C	T	0.00025	0.99975	0.00025	60.00	228.00	1.0000	74.00	74.00	0.0160	C
12	869243	chr12:739504	739504	G	A	0.00039	0.99961	0.00039	60.00	228.00	1.0000	74.00	74.00	0.0208	G
12	869314	chr12:739575	739575	A	C	0.00025	0.99975	0.00025	60.00	228.00	1.0000	83.00	83.00	0.0160	A
12	869342	chr12:739603	739603	T	C	0.00090	0.99910	0.00090	60.00	84.00	1.0000	18.00	18.00	0.0177	T
12	869365	chr12:739626	739626	T	C	0.00045	0.99955	0.00045	60.00	228.00	1.0000	29.00	29.00	0.0194	T
12	869372	chr12:739633	739633	G	A	0.00049	0.99951	0.00049	60.00	200.97	0.0022	51.04	51.04	0.0175	G
12	869411	chr12:739672	739672	A	G	0.00081	0.99919	0.00081	60.00	228.00	1.0000	49.05	50.95	0.0448	A
12	869491	chr12:739752	739752	A	G	0.00076	0.99924	0.00076	60.00	145.50	1.0000	13.15	56.85	0.0175	A
12	869524	chr12:739785	739785	G	A	0.00025	0.99975	0.00025	60.00	228.00	1.0000	66.00	66.00	0.0170	G
12	869556	chr12:739817	739817	C	T	0.00079	0.99921	0.00079	60.00	90.00	0.0010	21.00	21.00	0.0262	C
12	869651	chr12:739912	739912	A	G	0.00040	0.99960	0.00040	60.00	40.00	1.0000	10.00	10.00	0.0270	A
12	869674	chr12:739935	739935	A	C	0.00093	0.99907	0.00093	60.00	66.00	1.0000	13.00	13.00	0.0495	A
12	869689	chr12:739950	739950	A	G	0.00102	0.99898	0.00102	60.00	156.59	0.0005	30.58	31.84	0.0568	A
12	869777	chr12:740038	740038	A	G	0.00110	0.99890	0.00110	60.00	228.00	1.0000	19.15	35.40	0.0909	A
12	869794	chr12:740055	740055	C	T	0.00056	0.99944	0.00056	60.00	57.00	1.0000	10.00	10.00	0.1143	C
